# SVPG: a pangenome-based structural variant detection approach and rapid augmentation of pangenome graphs with new samples

**DOI:** 10.1038/s41592-026-03219-2

**Published:** 2026-09-21

**Authors:** Tao Jiang, Heng Hu, Runtian Gao, Shuqi Cao, Zhongjun Jiang, Murong Zhou, Wentao Gao, Shengming Zhou, Guohua Wang

**Affiliations:** 1 Faculty of Computing, Harbin Institute of Technology, Harbin, China; 2 College of Life Sciences, Northeast Forestry University, Harbin, China; 3 College of Artificial Intelligence, Zhejiang Agriculture and Forestry University, Hangzhou, China

**Keywords:** Software, Structural variation, Genome informatics

## Abstract

Breakthrough advances in long-read sequencing have opened unprecedented opportunities to study genetic variations through pangenome analysis, yet tools that effectively leverage such frameworks for structural variant (SV) detection remain limited. In addition, efficient construction of pangenome graphs becomes increasingly challenging with the acquisition of larger numbers of samples. Here we present SVPG, an approach that leverages haplotype-resolved pangenome reference for accurate SV detection and rapid pangenome graph augmentation from long-read sequencing data. Compared with state-of-the-art SV callers, SVPG maintained superior overall performance across different sequencing technologies and coverages. SVPG also achieved notable improvements in calling individual-specific SVs, including rare and somatic SVs. Furthermore, in a benchmark involving 20 samples, SVPG accelerated pangenome graph augmentation by nearly tenfold compared with traditional augmentation strategies. These results indicate that SVPG has the potential to improve SV detection and serve as an effective tool, offering new possibilities for advancing pangenomic research.

## Abstract (web summary)

SVPG performs accurate pangenome-facilitated structural variant detection and accelerates pangenome graph augmentation using long-read sequencing data.

## Main

Structural variants (SVs), defined as DNA sequence alterations larger than 50 base pairs (bp), encompass insertions, deletions, inversions, translocations and duplications^1,2^. Compared with single nucleotide variants, SVs are fewer in number; however, their broader genomic impact often leads to more important phenotypic changes^3,4^. In clinical medicine, SVs are closely associated with numerous genetic diseases, cancers and complex disorders, emerging as crucial molecular markers for disease diagnosis and treatment^5,6^. Simultaneously, SVs play an irreplaceable role in revealing genome evolution and analyzing population genetic diversity, providing key insights into species evolution and population characteristics^7^.

SV detection has primarily relied on single reference genome sequences as analytical benchmarks. However, traditional methods such as Sniffles2^8^ and cuteSV^9^ face a fundamental limitation: the single reference genome cannot comprehensively reflect the rich genetic variation within species. This issue is pronounced in highly polymorphic regions or population-specific sequences, often leading to reference bias^10–12^. This bias affects the accurate mapping of sample-specific sequences, thereby reducing SV detection reliability. The emergence of pangenomes offers a new solution to this challenge. By integrating genomic information from multiple representative individuals, pangenomes create a complete genetic variation map encompassing both shared sequences and population-specific variants^13–15^. This comprehensive genetic background significantly improves genome alignment accuracy, offering unique advantages in SV detection, particularly in complex variant regions. Recently, the Human Pangenome Reference Consortium (HPRC) successfully constructed the first human pangenome reference draft through sequencing and assembly of diverse global populations, establishing a solid foundation for improving SV detection accuracy and understanding human genetic diversity^14^.

Beyond improving detection of known variants, de novo variant discovery within the pangenome makes capturing individual-specific variants more flexible and pangenome refinement more scalable. By using pangenomes as backgrounds of genetic diversity, researchers can precisely identify rare variants absent from population-level data within a single genome. This fundamentally accelerates the discovery of rare SVs, especially cancer-specific SVs. However, as genomic studies expand in scale, pangenome construction and maintenance face substantial computational challenges. When integrating new samples into existing pangenomes, traditional approaches typically require re-execution of expensive de novo assembly and complex graph construction, with computational burden scaling superlinearly with sample numbers. In this context, rapidly and accurately discovering sample-specific SVs from new samples and dynamically integrating them into existing pangenomes without complete reconstruction will significantly reduce computational costs and accelerate iterative pangenome updates.

Although the utility of pangenomes for SV detection has been widely recognized, there is a lack of effective tools to fully leverage this advanced resource; existing tools have notable limitations. Early attempts, such as the pangenome-guided SV detection tool PanSVR^16^ and several short-read-based genotypers^12,17–19^ demonstrate the potential of leveraging pangenomes for accurate SV detection and genotyping. However, these tools were primarily designed for short-read sequencing data, which inherently limits their ability to resolve large SVs. Recently developed pangenome-based tools such as SVarp^20^ and miniSV^21^ represent significant advances in de novo SV discovery from pangenome. SVarp can generate SV-related contigs through local assembly, while miniSV provides direct SV calling based on the pangenome graph. However, both tools are limited to reporting variants outside the pangenome graph, and their performance in diverse genomic contexts has yet to be evaluated comprehensively.

Here we present SVPG, a tool designed for SV detection and pangenome graph construction. Leveraging the pangenome as a reference background, SVPG accurately filters false-positive calls in germline SV detection and refines SV breakpoint coordinates, improving overall SV detection performance. In addition, SVPG supports identification of de novo SVs using pangenome graphs and captures low-frequency variants associated with rare disease or cancer-related loci, providing support for rare disease etiology research and personalized cancer therapeutics. Finally, new SVs called by SVPG can be seamlessly integrated back into the pangenome framework, enabling efficient enhancement and continuous iteration of the graph structure, thereby establishing a reciprocal feedback mechanism between variant detection and graph evolution.

## Results

### Overview of SVPG

**Fig. 1:**
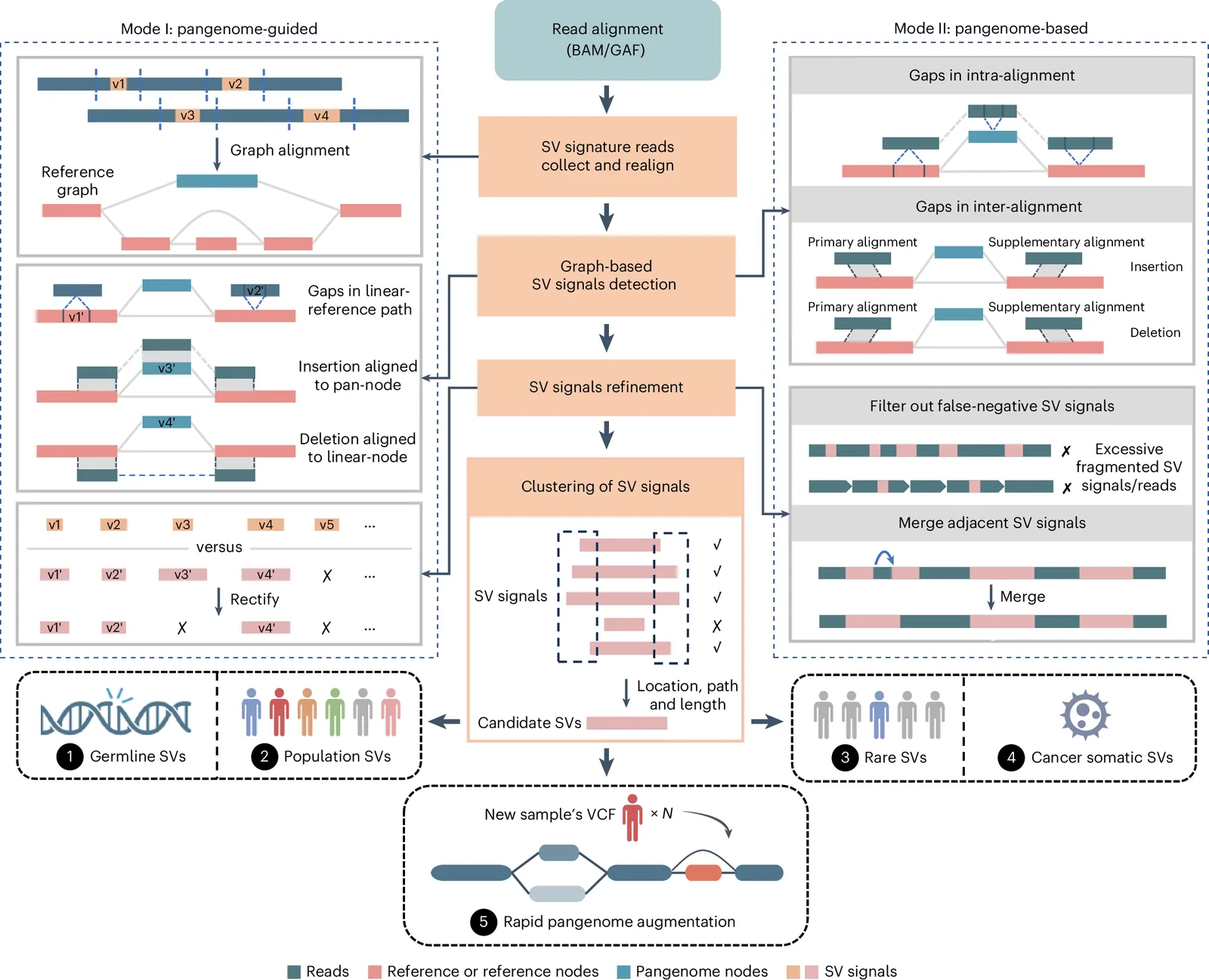
Overview of SVPG. SVPG supports two modes of SV detection, processing either BAM or GAF alignments, which correspond to pangenome-guided and pangenome-based SV detection, respectively. In the BAM-input mode (left), SVPG collects SV signature reads and aligns them to the pangenome reference, followed by graph-aware SV signal extraction and refinement. The GAF-input mode (right) enables direct extraction and refinement of de novo SV signals from sequence-graph alignments. Both modes use a common cluster-based refinement step, ultimately generating standard VCF output.

SVPG integrates two SV detection modes: pangenome-guided and pangenome-based SV detection (Fig. 1). In the pangenome-guided mode, SVPG first converts collected SV signals from a BAM file into signature reads and realigns these reads to the pangenome reference. By thoroughly analyzing the topological features and path transition patterns of SV signature reads in the pangenome graph, SVPG achieves high-precision SV breakpoint localization while optimizing the quality of original feature signals. In the pangenome-based mode, SVPG directly analyzes the alignment characteristics and patterns of raw read sequences within the pangenome graph structure without relying on the linear-reference genome, accurately detecting de novo SVs within haplotype paths of graph (see Methods for details). This dual-mode design provides SVPG with broader applicability: the first mode supports conservative and highly accurate germline SV calling for whole-genome and population-level analyses; the second mode is suited for detecting sample-specific events, including rare SVs absent from the pangenome graph and cancer-associated somatic SVs. Beyond the above-mentioned SV detection functions, SVPG integrates a graph augmentation pipeline, allowing researchers to rapidly call pangenome-based SVs from population-scale new samples and implementing graph augmentation functionality in conjunction with pangenome construction tools.

### SVPG enables high performance SV calling on GIAB

**Fig. 2:**
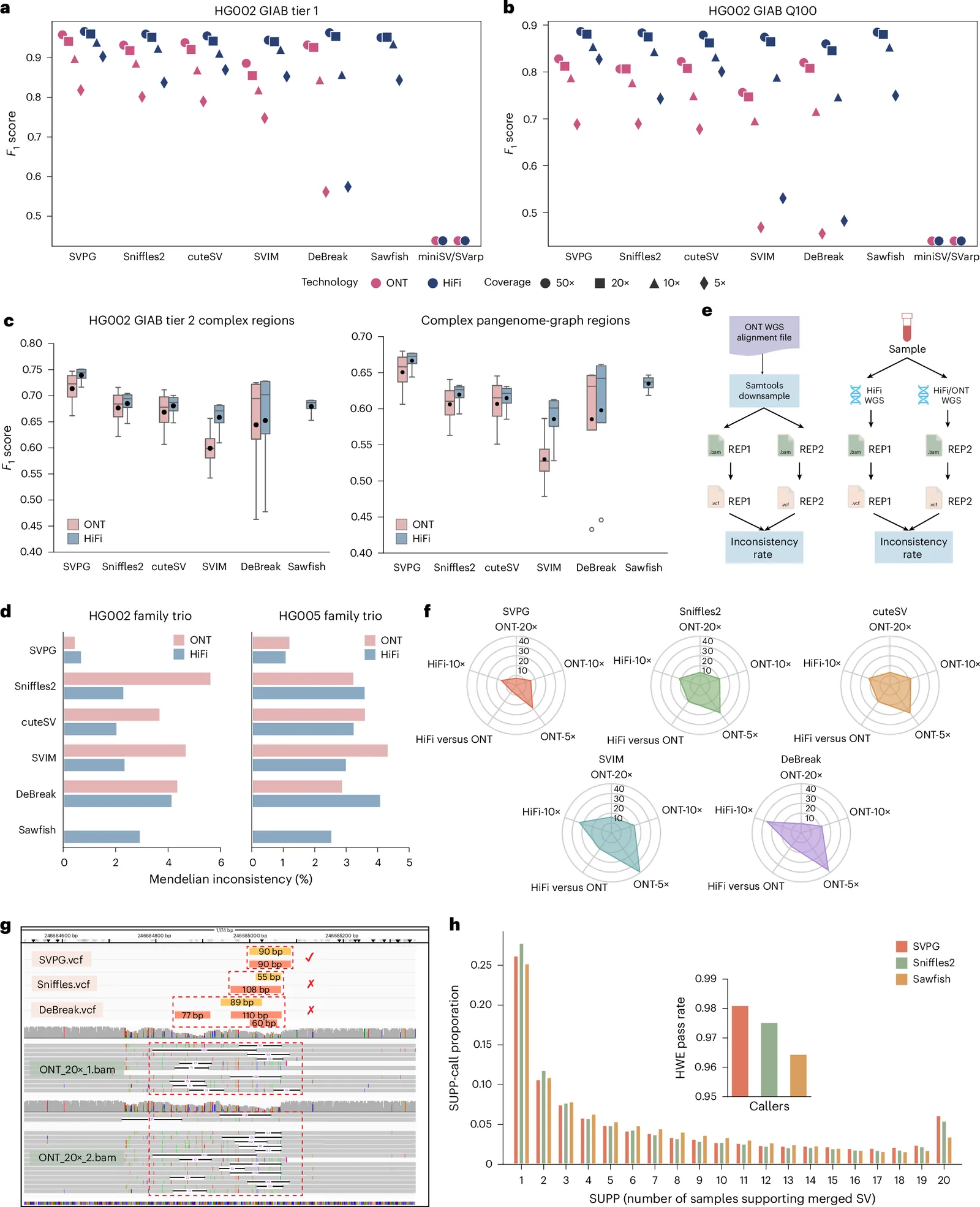
Performance assessment of SVPG in pangenome-guided SV calling. **a**,**b**, Comparison of SV calling *F*_1_ score using the GIAB-tier 1 (**a**) and GIAB-Q100 HG002 (**b**) benchmark across sequencing platform (ONT and HiFi) and coverage; symbols on the *x* axis indicate *F*_1_ < 0.5. **c**, Comparison of SV calling *F*_1_ score across four independent downsampling coverage levels (5×, 10×, 20× and 50×) in two complex genomic regions on HG002. The box plots show the median (Q2, 50th percentile), first (Q1, 25th percentile) and third quartile (Q3, 75th percentile), and the whiskers encompass observations within 1.5× the interquartile range from the first and third quartiles. The black dots indicate the mean values. **d**, Mendelian inconsistency rates across sequencing platform in HG002 and HG005 trio family. **e**, Schematic representation of three replicate-based experiments. **f**, Replicate inconsistency rates across five experimental groups: three ONT downsampling replicates (HG002), one cross-platform comparison (HG002 HiFi versus ONT) and one HiFi 10× replicate group (HG005). **g**, An example of inconsistent SV calls in the ONT-20× downsampling group. **h**, Multi-sample merging analyses across 20 HPRC samples. The *x* axis represents the sample support count (SUPP) of the merged SV loci, and the *y* axis shows the proportion of SVs at each SUPP level relative to the total number of calls produced by each caller. The inset figure shows comparison of the proportion of SVs that pass the HWE test (score ≥0.01). WGS, whole-genome sequencing. Source data

We first assessed pangenome-guided mode’s SV calling performance on HG002 sample’s Tier1_ v.0.6 ground truth set^22^, from the Genome in a Bottle (GIAB) consortium. We compared the SVPG with five leading long-read linear-reference-based SV callers, including Sniffles2^8^, cuteSV^9^, SVIM^23^, DeBreak^24^ and a recent published from PacBio specific HiFi SV calling tool, Sawfish^25^. In addition, two pangenome-based callers, miniSV and SVarp, were included in the comparison. In the high-confidence SV regions, SVPG was among the top performers with *F*_1_ scores of 0.958 and 0.966 for Oxford Nanopore Technologies (ONT) and HiFi platforms (see Fig. 2a and Supplementary Figs. 1 and 2 for more details). In subsequent downsampling datasets, SVPG maintained *F*_1_ scores above 0.90 even at low-coverage conditions (ONT-10× and HiFi-5× coverage), consistently demonstrating superior detection robustness.

We also evaluated all tools using the newly released GIAB HG002-Q100 benchmark set^26^, which provides a next-generation variant reference derived from a fully gapless, telomere-to-telomere genome assembly. On HiFi data, SVPG delivered a leading *F*_1_ score of 0.886, performing on par with Sawfish and outperforming all other methods (Fig. 2b). All tools showed reduced performance on ONT data, likely due to newly added low-complexity regions in the Q100 benchmark that challenge higher-error-rate technologies. Despite this, SVPG achieved the highest *F*_1_ score at 0.828. Analyses of downsampled datasets from both platforms further support the above observations. Notably, miniSV and SVarp performed poorly on the HG002 benchmark set primarily because they can only detect SVs outside the pangenome, we excluded both tools from subsequent HG002 benchmark evaluations.

Next, we benchmarked performance on representative challenging genome regions: GIAB-released HG002 tier2 regions (containing approximately 6,000 high-confidence SVs in complex or nonconsensus sequences), complex graph regions based on the pangenome reference (characterized by high-frequency multi-allelic variants; see Supplementary Figs. 3 and 4 for distribution and Methods for construction details), and clinically relevant regions defined in the Challenging Medical Relevant Genes^27^ (CMRG) (encompassing SVs in 273 medically significant genes). SVPG demonstrated significant performance advantage over other calling tools when analysis expanded from high-confidence to these complex regions. Particularly, SVPG achieved average *F*_1_ scores of 0.714, 0.651 and 0.881 across the three challenging regions using ONT dataset with multiple coverages, surpassing the others by 4–12%. For HiFi datasets, SVPG also achieved the highest average *F*_1_ scores (Fig. 2c and Supplementary Table 1). In addition, we applied all callsets to the Q100-defined hard regions to assess the consistency of SVPG’s performance (see Methods for details on region construction). Under this evaluation, SVPG outperformed all other approaches, achieving average *F*_1_ scores of 0.702 and 0.810 on ONT and HiFi data, respectively (Supplementary Table 2). Overall, SVPG consistently maintained stable, high-precision performance under various testing conditions, facilitating the reliable discovery of SVs in complex regions.

We further conducted Mendelian consistency analysis using two representative GIAB-released trio family datasets (Ashkenazim and Chinese Trio). SVPG achieved the lowest inconsistency levels in both families (ONT 0.5–1.2%, HiFi 0.7–1.1%; Fig. 2d). Reduction of SVs showing Mendelian inconsistency represents a crucial step in rapidly identifying de novo SVs, as manual screening of all inconsistent variants is typically required when searching for true de novo events. We selected a subset of de novo SVs called by SVPG and Sniffles2, in high-confidence regions of HG002 sample for further validation using Integrative Genomics Viewer (IGV)^28^ (Supplementary Note 1). In chromosomes (chr.) 1–3, results revealed that two out of five de novo SVs called by SVPG were confirmed as high-confidence variants. In contrast, of the 13 de novo SVs called by Sniffles2, 11 were either false positives or located in noisy regions with split alignments, while the remaining 2 genuine events were successfully identified by SVPG. These findings confirm that pangenome effectively eliminates Mendelian inconsistencies in SV calling.

### Benchmarking SV call consistency across replicates and samples

Benchmarking based solely on GIAB gold standards may constrains evaluation comprehensiveness and introduces systematic biases. We therefore adopted a replicate-based strategy, in which genuine SVs should be consistently detected across replicates, whereas false positives arising from technical artifacts (for example, library preparation or sequencing errors) typically appear in single samples^29–31^. To avoid sensitivity biases caused by uneven sequencing depth in actual repeat sequencing, we simulated replicate sample pairs with the same coverage by downsampling BAM files of the same sample, using different random seeds (Fig. 2e). We ran SVPG’s pangenome-guided mode and other calling tools on replicate sample pairs of HG002 at 20×, 10× and 5× coverage from ONT, comparing the inconsistency of the SV sets detected. The results showed that SVPG exhibited the lowest inconsistency in all three downsampling replicate experiments (Fig. 2f; Fig. 2g shows an example), significantly outperforming other tools. As sequencing coverage decreased, the inconsistency rate of all tools increased significantly, consistent with the expectation that reduced read support at low coverage leads to more false positives. We also validated the inconsistency using a set of 10× coverage raw HiFi data from HG005, similar results were obtained, further confirming the stability advantage of SVPG.

We further assessed the consistency of SV calls across different sequencing platforms using of HG002 HiFi and ONT data. Using high-accuracy HiFi data as the control, SVPG showed only an 7.4% discrepancy between platforms, substantially outperforming other tools (for example, 17.1% of the second-best DeBreak). This exceptional cross-platform consistency of SVPG, despite similar *F*_1_ scores among tools on the same platform, highlights the ability to reduce false positives by leveraging pangenome-guided refinement and thereby minimize technology-specific biases.

To validate SVPG’s cross-sample consistency at the population level, we next performed SVPG calling on each of the 20 HPRC samples (Supplementary Table 3) and merged the results, alongside parallel workflows using Sniffles2 and Sawfish. We used the number of supporting samples per merged site as a proxy for population frequency and quantified the proportion of SVs falling into different support intervals for each method. SVPG exhibited a markedly higher fraction of high-support (high-frequency) variants (Fig. 2h), indicating superior sensitivity to recurrent, reliable SVs shared across individuals. Consistent with this, Hardy–Weinberg equilibrium (HWE) analysis further revealed that SVPG produced the highest proportion of loci (98.1%; HWE test scores ≥0.01 were considered pass) conforming to expected allele-frequency distributions. This closer agreement with the theoretical HWE model highlights SVPG’s improved genotyping consistency and reduced bias. Overall, these results highlight SVPG’s advantage in population-scale analyses: leveraging the pangenome framework, SVPG enforces unified representations of the same SV across samples, thereby suppressing sample-specific noise and enhancing cross-sample consistency and comparability.

### Pangenome-based rare SV calling performance assessment

Rare SVs predominantly represent recent or de novo structural mutations that reveal potential genomic mutation mechanisms. The 1,000 Genomes Project demonstrated that 96–99% of variant alleles in the human genome are shared within populations^32^, and this information has now been systematically incorporated into pangenome references. Leveraging this characteristic, we developed a pangenome-based SV calling mode, which established a reliable approach for sample’s rare SV identification by detecting de novo SVs that were absent in the pangenome. To assess the approach, we curated 66,197 rare SVs of varying types and sizes from an existing database as the gold standard benchmark set^33^, and generated two 20× coverage simulated datasets (ONT and HiFi) containing these representative rare variants. By using the ‘bedtools jaccard’^34^ command, we compared these SVs with the bubble regions in the pangenome reference, and found that only 2.6% of the regions overlapped, further confirming the rarity of these SVs (see Methods for benchmark set construction details).

**Fig. 3:**
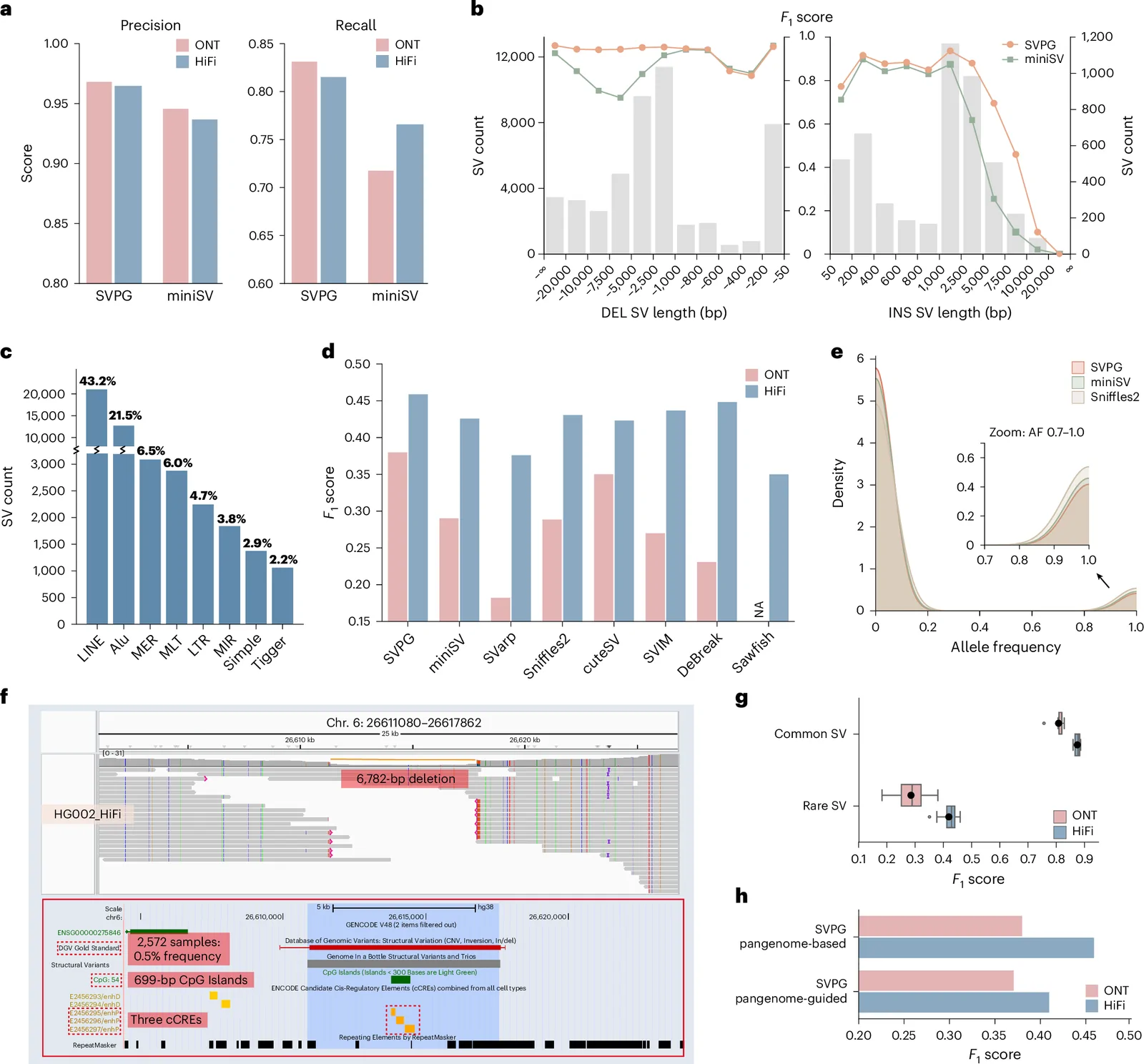
Performance evaluation of SVPG in pangenome-based SV calling. **a**, Comparison of pangenome-based de novo SV calling performance on simulated rare SV datasets. **b**, Performance across SV types and length ranges on simulated ONT data; lines show *F*_1_ scores and bars indicate SV counts called by SVPG. **c**, Overlap of SVPG true positive rare SVs from the ONT dataset with common repeat family. **d**, Performance comparison of pangenome-based (top three) and linear-reference-based tools in detecting HG002-Q100 rare SVs. **e**, Allele-frequency (AF) distribution of true positive rare SVs detected by SVPG, miniSV and Sniffles2 from HiFi data; inset shows AF = 0.7–1.0. **f**, A 6,782-bp rare large deletion (chr. 6: 26,611,080) called by SVPG in HG002. The bottom panel of UCSC Genome Browser snapshot indicates the SV supported by the Database of Genomic Variants (DGV; marked in red, ~0.54% frequency), confirming its rarity. The deletion overlaps a 699-bp CpG island and three candidate *cis*-regulatory elements (cCREs, yellow), suggesting potential disruption to local regulatory activity. **g**, Performance comparison for calling common SVs from six tools and rare SVs from eight tools in the HG002-Q100 benchmark set. The box plots show the median (Q2, 50th percentile), first (Q1, 25th percentile) and third quartile (Q3, 75th percentile), and the whiskers encompass observations within 1.5× the interquartile range from the first and third quartiles. The black dots indicate the mean values. **h**, Performance comparison of SVPG in pangenome-based versus pangenome-guided modes for HG002-Q100 rare SV detection. Source data

We input the simulated read alignments against the pangenome reference into SVPG’s pangenome-based calling mode, and compared its performance with that of miniSV (SVarp was excluded because it failed to finish after 5 days of runtime). SVPG achieved *F*_1_ scores of 0.898 and 0.888 on ONT and HiFi data, respectively, surpassing that of miniSV by 8.1% and 4.5%, respectively. SVPG maintained low false-positive rates while successfully recalling more accurate rare SVs (Fig. 3a and Supplementary Fig. 5). This performance advantage was consistent across different SV size ranges and SV type, with particularly notable improvements in regions associated with transposable element activity, such as around 300 bp, 2 kilobases (kb) and 6 kb (Fig. 3b). In addition, the size distribution of rare SVs exhibited distinct abundance at these intervals, implying that many of these rare SVs may be influenced by the activity of transposable elements (Supplementary Fig. 6). This observation is further supported by our overlap analysis between rare SVs and main classes of repeat elements (Fig. 3c). These results highlight the widespread impact of repeat elements (Alu, LINE1, SVAs) in shaping human genomic diversity, and high-level similarities between our SV catalog and other studies^1^.

We further benchmarked SVPG on HG002 to analyze the performance to detect rare SVs in a real sample. Our evaluation was based on two key assumptions: (1) the existing pangenome reference already covered the most common SVs, so de novo SVs called from the pangenome graph are likely to represented individual-specific rare SVs; and (2) an individual’s rare SVs should be a subset of their complete SV spectrum, with benchmark-validated SVs considered as true positive. Specifically, we stratified the GIAB-Q100 SVs using the pangenome reference: variants not overlapping with pangenome bubbles were labeled as rare SV ground truth, while the remaining were labeled as common SVs. We then aligned HG002 sequencing data to the pangenome reference and performed pangenome-based SV calling (that is, SVPG, miniSV and SVarp) and linear-reference-based SV calling (that is, Sniffles2, cuteSV, SVIM, DeBreak, Sawfish). Overall, SVPG achieved 3.0–19.8% and 1.0–10.8% more *F*_1_ scores than other callers on ONT and HiFi data from the Q100 rare SVs (Fig. 3d), respectively. These SVs were further confirmed as genuinely rare through verification against the Human Genome Structural Variation Consortium dataset of 65 individuals (Fig. 3e). Figure 3f shows an example of a rare deletion called by SVPG, with the UCSC Genome Browser screenshot suggesting that this variant may be pathogenic.

Moreover, we found that rare SVs are substantially more difficult to detect than common ones (Fig. 3g). Even so, the pangenome-based strategy remains highly effective: compared with the pangenome-guided mode, the full pangenome-based mode enables SVPG to detect rare SVs with markedly higher accuracy (Fig. 3h). It is owing that pangenome-based mode is more sensitive to sequences absent in the pangenome, while pangenome-guided mode inherits biases from the linear reference, limiting its ability in the fully rare SV detection.

### Pangenome-based somatic SV calling performance assessment

**Fig. 4:**
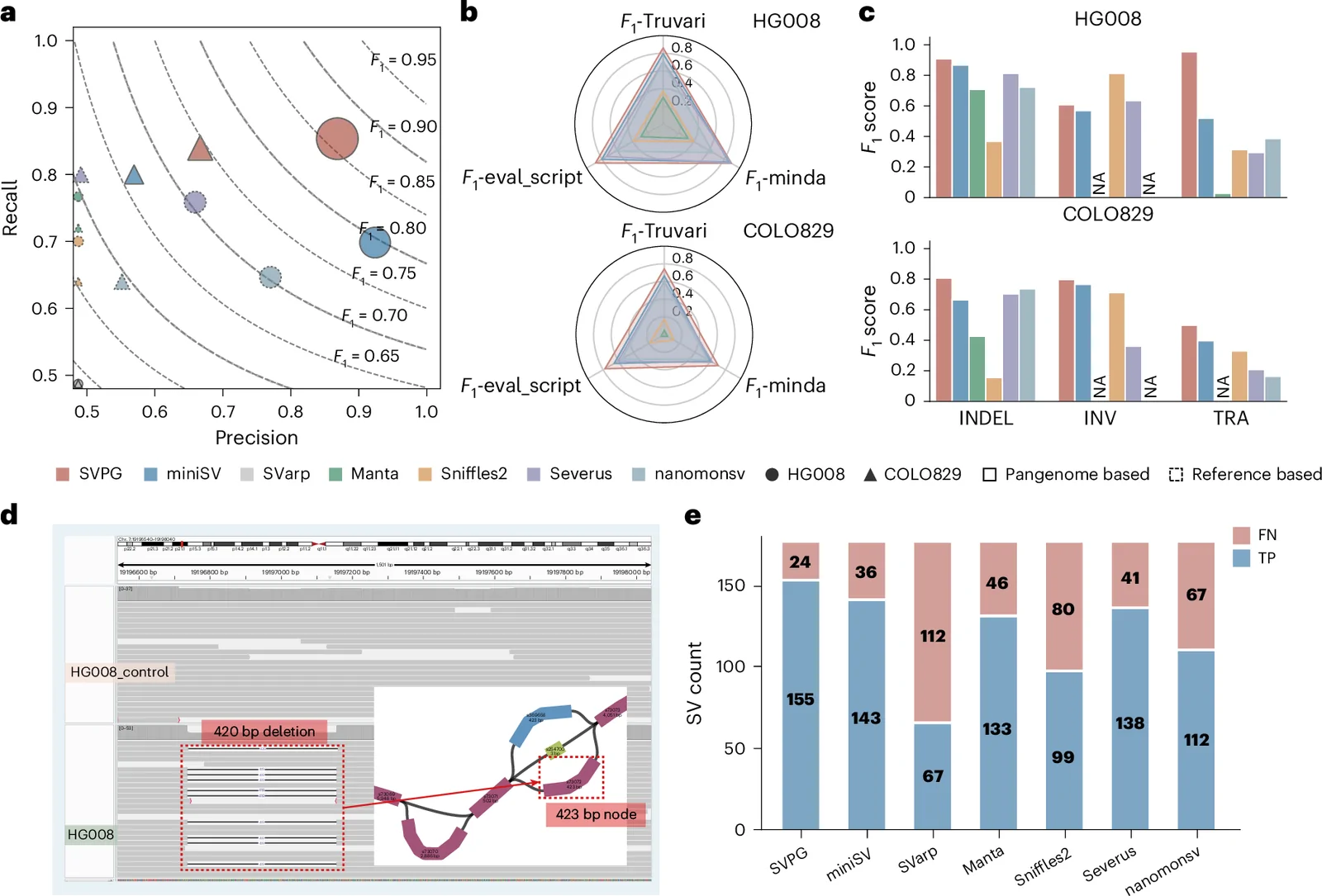
Performance evaluation of SVPG in somatic SV calling. **a**, Precision–recall bubble plot of somatic SVs called from HG008 and COLO829. Solid lines represent pangenome-based methods and dashed lines represent linear-reference-based methods. Symbols positioned on the axis indicate the score below 0.5. **b**, *F*_1_ scores of somatic SV calling on the HG008 and COLO829 assessed using Truvari, minda and custom evaluation scripts. **c**, *F*_1_ score comparison across SV types using custom evaluation scripts. Duplications are treated as insertions and included within the INDEL category. **d**, A missed somatic SV by SVPG in HG008. IGV screenshot shows a 419-bp deletion at chr. 7: 19196736 in HG008, previously reported by GIAB as cancer-specific. Bandage visualization indicates that this deletion is already represented in the pangenome graph, leading to its omission by SVPG. **e**, True positive (TP) and false-positive (FP) counts of somatic SV calling on the HCC1395 biologically validated benchmark. Source data

To evaluate the potential of pangenomes for cancer-specific SV detection, we applied the SVPG’s pangenome-based mode into a somatic SV discovery pipeline. We expected that aligning reads from both tumor and adjacent normal tissues to the pangenome, rather than to the single linear-reference genome, may help reduce false positives caused by reference bias. We assessed SVPG, miniSV, SVarp and five linear-reference-based callers using recently GIAB-released HiFi datasets from the HG008 cancer cell line and its matched control^35^, as well as the widely used COLO829^36^ tumor–normal pair (see Methods for benchmark details). Following the GIAB-recommended benchmarking, SVPG achieved *F*_1_ scores of 0.861 on HG008 and 0.743 on COLO829 using Truvari, substantially outperforming other methods (Fig. 4a). Compared with traditional linear-reference-based callers (such as nanomonsv^37^ and Severus^38^), the pangenome-based SVPG and miniSV both exhibited significantly lower numbers of false-positive calls. We further evaluated all methods using minda (an independent somatic SV assessment tool developed for Severus), as well as our custom evaluation script specifically designed for SV-type-based assessment of cancer-associated SVs. Figure 4b shows that SVPG exhibited highly consistent performance across these distinct evaluation tools. In addition, SVPG maintains strong and well-balanced performance across multiple SV types using evaluation scrip (Fig. 4c). Furthermore, a closer inspection of HG008 dataset the five insertions and deletions (INDELs) missed by SVPG revealed that four were already represented in the pangenome graph, suggesting that they are likely somatic SVs overlapping with common germline SVs (Fig. 4d show one case; see Supplementary Note 2 for all). Overall, we attribute the improvement and low false-positive rate results to SVPG effectively leverages pangenome characteristics to enable precise inference cancer-specific SV.

We also analyzed an earlier yet widely studied HCC1395 breast cancer cell line^39^, and its matched normal HCC1395BL. This benchmark was established through the biological validation of a curated subset of SVs derived from consensus germline callsets. Because the dataset lacks rigorously defined high-confidence genomic regions necessary for an accurate assessment of false positives, we prioritized recall as the primary metric for our performance evaluation. As shown Fig. 4e, SVPG achieved the highest sensitivity, identifying 155 out of 179 validated SVs, followed closely by the pangenome-based method miniSV. We further performed the same evaluation on the consensus benchmark set comprising 1,268 SVs identified by at least two germline SV callers. We found that 951 SVs were not reported by SVPG. Notably, most of these SVs were also missed by nanomonsv (949) and Severus (899), indicating that these events may be broadly challenging for current somatic SV callers. Among the 951 SVs not detected by SVPG, cross-validation using ‘bedtools intersect’ showed that 829 overlap with node regions in the pangenome reference, indicating that these hard-to-call somatic SVs occur in regions enriched for common germline SV (Supplementary Table 4). In contrast, only 25 of the 285 SVs detected by SVPG overlap with pangenome nodes, which is significantly lower than the overlap observed in the unrecalled group (Fisher’s exact test, *P* < 0.001). These regions expose a fundamental limitation of current somatic SV evaluation frameworks, where independent somatic events may overlap common germline variation in highly polymorphic regions. This ambiguity affects SVPG as well as other state-of-the-art callers, underscoring the need for more refined benchmarking strategies that explicitly account for overlapping germline and somatic variation.

### Rapid pangenome graph augmentation from SVPG’s graph-called SVs

**Fig. 5:**
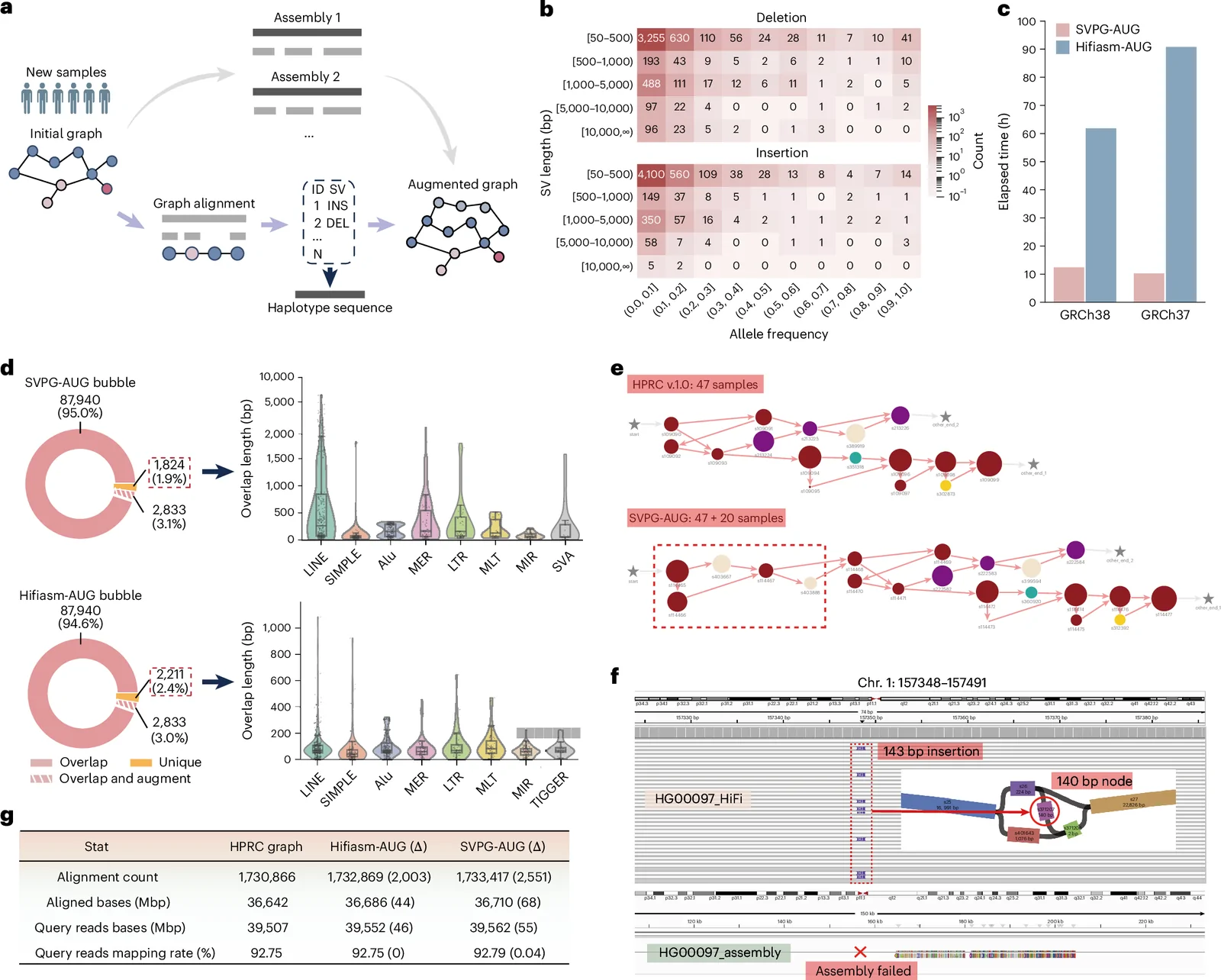
SVPG pangenome graph augmentation results. **a**, Comparison of two augmentation strategies: iterative graph augmentation via de novo assemblies (hifiasm-AUG, upper) versus integration of population-scale variant calls from graph alignments (SVPG-AUG, lower). **b**, Distribution of SVs called by SVPG across 20 HPRC samples by length and allele frequency. **c**, Comparison of computational time required for constructing SVPG-AUG and hifiasm-AUG using 20 HPRC samples. **d**, Composition of bubble regions in GRCh38-based SVPG-AUG and hifiasm-AUG. ‘Overlap’ indicates nonaugmented bubbles that are shared between the two augmented graphs (original graph bubbles); ‘Overlap and augment’ represents newly added bubbles compared with the original graph and ‘Unique’ indicates graph-specific bubbles. The violin plot shows the overlap (1,824 in SVPG-AUG and 2,211 in hifiasm-AUG) between unique bubbles and repetitive elements, displaying the top eight repeat families, and SV lengths >1,000 bp are log-transformed for SVPG-AUG. The box plots show the median (Q2, 50th percentile), first (Q1, 25th percentile) and third quartile (Q3, 75th percentile), and the whiskers encompass observations within 1.5× the interquartile range from the first and third quartiles. The black dots indicate the mean values. **e**, Local graph topology at the *MUC6* locus in HPRC GRCh38-based pangenome graph (upper, 17 nodes), and SVPG-AUG (lower, 22 nodes); newly introduced nodes are highlighted in red box. **f**, An SVPG**-**AUG-specific node example. The upper IGV panel shows a 143-bp insertion at chr. 1: 157348 in HG00097 sample, called by SVPG and successfully integrated into the graph; the lower IGV panel indicates that hifiasm failed to assemble this region. **g**, Summary of alignment metrics obtained by mapping HG002 HiFi reads to the HPRC GRCh38-based pangenome graph and its two corresponding augmented versions; delta (Δ) denotes the increase compared with the original graph. Source data

Traditional pangenome augmentation, exemplified by HPRC efforts, typically relies on complex and time-consuming de novo assembly processes that require high-quality sequencing data and multiple refined assembly steps. We propose a rapid graph augmentation strategy based on de novo SVs identified by SVPG from graph alignments. By using existing high-quality pangenome reference graphs for initial SV calling and merging, the strategy directly incorporates these variants into the original pangenome, thereby circumventing elaborate assembly procedures. Figure 5a illustrates the key differences between the two graph augmentation strategies. We selected 20 HPRC samples’ HiFi data and applied SVPG’s graph augmentation mode, using the HPRC-released original pangenome reference graphs as the foundation for generating augmented versions (Supplementary Fig. 7). Analysis of population-level SVs called by SVPG revealed that these de novo SVs, predominantly ranging from 50 bp to 500 bp in length, existed at allele frequencies of approximately 0.1, further indicating the rarity (Fig. 5b). Subsequently, we constructed an augmented graph based on de novo assembly using the hifiasm^40^ assembler, following the standard pipeline of HPRC. Given our primary focus on SVs exceeding 50 bp, all graphs were constructed using minigraph^41^, which is optimized for capturing large-scale genomic rearrangements. Overall, SVPG completed this process within 0.5 day, while for the assembly strategy even the initial hifiasm assembly step took up to 3 days, with substantially longer timeframes when including additional refined assembly procedures (Fig. 5c).

We denote the graph-alignment-based augmented graph as SVPG-AUG and the assembly-based augmented graph as hifiasm-AUG. Compared with the original graph, both augmentation graphs exhibited a substantial increase in nodes and edges (left panel of Fig. 5d), indicating enhanced the expressive capacity of the graph representations. Analyzing bubble regions that represent variant sites within the graphs, we observed remarkable similarity between the two augmented graphs. Using GRCh38-based augmented graph as an example, we found that 98% of the 92,597 bubbles identified in SVPG-AUG overlapped with bubbles in hifiasm-AUG. We further analyzed the subset of bubble regions unique to each augmented graph with respect to annotated repetitive elements. Among the 1,824 SVPG-AUG–specific bubbles, 1,322 (72.5%) overlapped with known repeats, while 1,689 (76.4%) of the 2,211 hifiasm-AUG–specific bubbles overlapped with repeats. Among the eight most abundant repeat families ranked by overlap count (right panel of Fig. 5d), SVPG-AUG exhibited overall greater overlap lengths, particularly with long interspersed elements such as LINEs and Alus. In contrast, hifiasm-AUG displayed a greater number of overlapping regions, especially with shorter, high-copy elements such as simple repeats. These results indicate that, despite differing methodologies, both augmentation strategies yielded highly consistent variant representations, while also capturing distinct repeat-associated variation.

To compare how pangenomes constructed from complete haplotypes capture specific biologically relevant loci, we focused on the *MUC6* gene region, a locus known for its extensive polymorphism and strong clinical relevance^42^ (see UniProt entry https://www.uniprot.org/uniprotkb/Q6W4X9/entry). We extracted the subgraph corresponding to *MUC6* regions (chr. 11: 1012823–1036718), allowing us to directly inspect how structural diversity is represented across different graph. As shown in Fig. 5e (PanGraphViewer^43^ for graph visualization), SVPG-AUG subdivides an original segment into five distinct nodes, capturing sample-specific structural rearrangements contributed by HG00140 and HG00323 samples. In contrast, hifiasm-AUG maintains a topology similar to the original graph (Supplementary Fig. 8), indicating a more conservative representation of variation in this region. As another case study, we examined 881 genomic regions in the HG00097 sample where hifiasm failed to assemble long-read sequences (all exceeding the 15-kilobase pairs average HiFi read length). Within these regions, SVPG identified 25 de novo SVs. Figure 5f presents an example where an SVPG-called SV was successfully incorporated to augment the graph: a 143-bp insertion called by SVPG at the assembly-failed region in the HG00097 sample and encoded in the augmented graph, by decomposing the normal path to create a corresponding bubble structure (s371207). Another example is presented in Supplementary Fig. 9. In conclusion, these findings strongly demonstrate that SVPG can effectively augment pangenome structures in assembly-challenging regions, providing more comprehensive genomic characterization for SV analysis.

Finally, we aligned HG002 HiFi data to the pangenome graphs to assess the impact of augmented graphs on downstream applications. Minigraph alignment showed that both augmented graphs improved mapping over the original graph. SVPG-AUG yielded 2,551 more alignment records and 68 megabase pairs of additional aligned bases, with slightly higher than hifiasm-AUG (Fig. 5g). Further, we substituted the original graph with augmented graphs to examine their influence on SV calling under the SVPG pangenome-guided mode. Results indicated that the SVPG’s calling performance remained largely consistent across both augmented graphs. In the CMRG regions, SVPG-AUG exhibited modest improvements (Supplementary Table 5). These findings indicate that current pangenome references already capture substantial SV information, and augmented graphs have yet to show marked improvements in common germline SV calling within current benchmarks. However, with advancing technologies and expanding datasets, optimized pangenomes may offer greater benefits, particularly in complex variant regions or diverse population analyses.

## Discussion

This study introduces SVPG, a method to incorporate pangenome information into SV detection of long-read sequencing. Unlike traditional long-read SV calling methods that rely on linear reference, SVPG leverages pangenome-guided priors to force-call and annotate widespread common SVs while retaining high sensitivity for sample-specific events. We showed that SVPG consistently outperforms existing state-of-the-art methods, and both unbiased replicate experiments and cross-sample analyses further highlighted its substantially enhanced call consistency on the population scale and markedly reduced false negatives. These findings not only validate the potential importance of pangenome resource in SV detection but also provide a promising direction for future developments in this field.

Furthermore, the SVPG pangenome-based SV calling mode enables de novo SV detection from pangenome graph. This offers a new perspective on rare SV discovery, which traditionally relies on large-scale population sequencing data analysis: an approach that is both resource intensive and prone to missing individual-specific rare variants, particularly without adequate genetic background references. SVPG pangenome-based detection leverages population genetic variation information embedded in pangenomes to achieve accurate rare SV detection at the single-sample level with high sensitivity, eliminating the need for population-scale data support. This capability has significant implications for elucidating disease-associated variants and individual-specific phenotypic mechanisms.

Notably, SVPG demonstrates that pangenome significantly enhances the detection of somatic SVs, the specialized and distinct class of rare SVs. By incorporating pangenome-based background information, SVPG outperforms linear-reference-based methods with higher precision. Building on this advantage, SVPG could be further extended to integrate personalized or population-specific pangenome references, more precisely identifying cancer-specific variants through a more comprehensive and sample-relevant genomic background. Further analysis revealed that some false negative SVs unreported by SVPG are actually visible in the germline-derived pangenome, suggesting that current published cancer SV benchmark sets may contain nongenuine cancer-driving mutations. This finding emphasizes that pangenome background is an essential factor when constructing cancer-specific SV benchmark sets.

Our exploration of SVPG application in pangenome graph augmentation also yielded promising results. Using graph SVs called from 20 human samples, we implemented a graph-alignment-based augmentation strategy that integrates SVPG-called graph SVs directly into existing pangenomes. The resulting augmented graphs showed high concordance with those generated from assembly-based approaches, while reducing the computational time required for graph construction by nearly tenfold across 20 samples, and this efficiency is expected to increase further with larger sample sizes. Notably, while we present several assembly-failure cases, these examples are based on preliminary native assemblies, we acknowledge that with sufficiently accurate and high-coverage reads, together with appropriate postprocessing (for example, trio-binning, polishing, scaffolding), de novo assembly can resolve most complex regions, particularly longer segmental duplications or satellite. Nevertheless, when data quality or cost constraints limit assembly success, SVPG provides a cost-effective and scalable alternative for augmenting pangenome graphs, offering substantial practical advantages for large-scale, population-level analyses.

Although this study represents an attempt at leveraging pangenomes for SV detection, it still faces several unresolved challenges. First, precise SV detection and reliable assessment in complex genomic regions remain challenging. SVPG performance is intrinsically contingent on the quality of pangenome resources and the accuracy of graph alignment (Supplementary Table 6 shows the impact of different graph-alignment tools on SVPG’s performance). As observed when using tools such as minigraph, multi-allelic variable number tandem repeats pose particular challenges for current graph construction frameworks, preventing an optimal graph representation and alignment. Besides introducing more specific heuristics for complex regions, improving graph-based algorithms remains crucial for advancing pangenome-based SV detection. Second, while alignment-based graph augmentation approaches offer efficient and cost-effective solutions, they rely on an intricate pipeline of multiple tools, including graph alignment, variant calling and merging, and graph construction. This multi-tool dependency introduces compound error sources that are particularly pronounced in multi-allelic regions, where sequence similarities and structural complexities can cascade through each analytical step. In the future, incorporating more fine-grained local assembly may be beneficial for improving sequence accuracy.

As initiatives such as HPRC progress and reference graphs continue to evolve, pangenome references are expected to rapidly expand to thousands of haplotypes in the coming years. In this context, the high accuracy and versatility of SVPG position it advantageously for both SV detection and graph augmentation. We anticipate that pangenome-based SV detection methods such as SVPG will play an increasingly important role in whole-genome analysis pipelines.

## Methods

### SVPG methodology

#### SV signature reads collection and pangenome realignment

In the pangenome-guided mode, SVPG takes a linear-reference genome alignment result as the input and extracts SV signatures through two heuristic rules, according to the signal source type: (1) gap sequences in the CIGAR string of primary alignments exceeding predefined thresholds, indicating potential insertions or deletions, and (2) abnormal distances between primary and supplementary alignments from split-read mapping, with sequence lengths exceeding predefined thresholds, usually indicating more complex or larger SVs.

Each detected SV signal is instantiated as an independent object with key attributes: breakpoint coordinates in both reference genome and reads, complete read sequence, read name and signal source type. SVPG then scans each SV signal object to generate SV signature reads based on their attributes. In the standard pipeline, SVPG extracts an additional 2,000-bp window sequence on both sides of the breakpoints of the SV signal as alignment anchors. For signals from split alignments, the complete original read sequences are used directly. Notably, when SV breakpoints are less than 2,000 bp from read ends, SVPG supplements missing sequences from the reference genome based on the SV signal’s reference breakpoint position, to ensure sequence window completeness. These flanking sequences are concatenated with SV signal sequences to form complete signature read sequences, with key SV signal attributes recorded in the read description line, for subsequent refinement steps.

SVPG employs minigraph for rapid realignment of SV signature reads to the pangenome reference graph. Parameters are configured according to sequencing data types: ‘-x asm’ for HiFi data and ‘-x lr’ for ONT data. The ‘-vc’ parameter enables output of unstable sequence coordinates to support standard GFA graph format.

#### Graph-aware SV signal detection and refinement

Unlike BAM files using linear-reference coordinates, GAF data structure describes sequence-to-graph-alignment information through graph paths and path coordinates. Graph paths comprise ordered nodes and edges, with each node’s start and end uniquely corresponding to linear-reference coordinates. SVPG first converts the pangenome graph structure into a dictionary for efficient node querying and graph traversal, with the node name as keys and their properties (sequence length, coordinate information and topological relationships) as values.

Graph-aware SV signal detection handles two scenarios: (1) alignment paths containing only linear-reference nodes. Here path discontinuities typically indicate deletions. SVPG accesses the graph dictionary by the missing node names to obtain node coordinates and spans, which accurately represent the SV breakpoints. In addition, gaps in path CIGAR strings further localize SV breakpoints based on path-start nodes and alignment-start coordinates in the path, thereby capturing variants that are not explicitly encoded in the graph. (2) Alignment paths including nonreference haplotype nodes. SVPG identifies insertions based on haplotype nodes, with variant spans equal to the total span of all haplotype nodes between linear-reference nodes. For CIGAR string gaps, processing depends on gap location. Gaps in linear-reference node paths are preserved; gaps in haplotype node paths adjust the insertion span represented by that haplotype node. Specifically, insertion-induced gaps increase the original insertion span by the gap length, while deletion-induced gaps decrease it. Finally, for complex paths containing both haplotype and discontinuous nodes, SVPG preserves deletion signals represented by missing nodes and subtracts haplotype node length from signal span.

The complex topology of pangenome graphs is more prone to alignment artifacts, particularly in highly variable or repetitive regions. To mitigate these effects, SVPG implements stringent signal refinement: all graph-called SV signals within the same signature read are compared against the original SV signature signal for filtering. Specifically, SVPG selects graph-called SVs with breakpoints closest to the raw SV signal. If the graph SV signal span does not meet consistency principles with the raw signal span, all graph SV signals from that signature read are filtered out. The consistency principle determines the reliability of an SV signal based on the span of the called SV (span__graph_) and raw SV signal (span__raw_); if $\frac{\min \left({\mathrm{span}}_{\mathrm{graph}},{\mathrm{span}}_{\mathrm{raw}}\right)}{\max \left({\mathrm{span}}_{\mathrm{graph}},{\mathrm{span}}_{\mathrm{raw}}\right)} > 0.5$, the SV signal is considered highly reliable.

#### Clustering of SV signal

SVPG uses a two-stage clustering strategy to transform scattered graph SV signals into candidate SVs. The first stage performs coarse-grained clustering based on spatial similarity, grouping SV signals of the same type into initial clusters, if their breakpoint distances are within a predefined threshold (default 1,000 bp). This spatial proximity-based clustering broadly captures multiple signals originating from the same SV event.

The second stage focuses on SV signal feature similarity analysis, refining the initial clusters through three feature dimensions: variant span, breakpoint coordinates and graph path. Span similarity undergoes difference-based normalization, while breakpoint coordinate similarity is normalized using span and parameter *σ*, where *σ* is determined by both local cluster SV signal abundance (local*N*) and global SV signal abundance (global*N*): ${\rm{\sigma }}=\frac{\left|{{\mathrm{local}}N}-{{\mathrm{global}}N}\right|}{\max \left({{\mathrm{local}}N},{{\mathrm{global}}N}\right)}+{{\mathrm{global}}N}$. Graph path similarity employs Jaccard distance with a penalty *μ* (default 0.05). The overall similarity between any two SV signals is expressed as: $S=\frac{1}{\max ({\mathrm{span}}_{1},{\mathrm{span}}_{2})}(|{\mathrm{span}}_{1}-{\mathrm{span}}_{2}|+\frac{|{\mathrm{loc}}_{1}-{\mathrm{loc}}_{2}|}{\sigma })+\mu \times (1-\frac{{\mathrm{path}}_{1}\cap {\mathrm{path}}_{2}}{{\mathrm{path}}_{1}\cup {\mathrm{path}}_{2}})$. Based on multi-dimensional feature similarity analysis, initial clusters undergo hierarchical clustering (default threshold 0.3) to form highly homogeneous subclusters, each representing potential SV alleles in local genomic regions.

#### SV call and genotyping

Before finalizing candidate SVs, SVPG implements a quality control strategy based on minimum supported reads and alignment path consistency. For each SV cluster, variants sharing identical graph traversal paths are subject to a relaxed read support threshold, effectively recovering high-quality SV signals that may otherwise be missed due to insufficient sequencing depth. This pangenome-based alignment path consistency filtering mechanism effectively balances the sensitivity and specificity of detection.

Clusters passing quality control proceed to final SV calling and genotyping. SVPG uses the median of all SV signal breakpoint coordinates within a cluster as the final candidate SV breakpoint coordinates. In addition, the SVPG genotyping module assigns optimal genotypes (0/0, 0/1 and 1/1) to each candidate SV through maximum likelihood estimation based on sequence coverage statistics in variant flanking regions.

#### Graph-aware de novo SV signal detection and refinement

The SVPG pangenome-based SV detection mode processes a GAF format that graphs alignments, converting gap information from each alignment path’s CIGAR string into corresponding SV signals. Besides internal graph-alignment information, SVPG encodes SV signals from external graph alignments. Given the lack of explicit supplementary alignment markers in the GAF format compared with the BAM format, SVPG sorts multiple alignment records from the same read by genomic position, designating the upstream alignment as primary and determines SV breakpoint coordinates through primary–supplementary alignment relationships. Subsequently, SVPG infers the SV types (include larger INDELs, inversions, duplications and translocations) based on the orientation and clipping location of the two segments involved.

Subsequently, SVPG implements postprocessing steps to enhance SV signal quality, including: (1) filtering SV signals from reads exceeding thresholds for fragmented SV signals or split operations during alignment; (2) refining SV breakpoints in repetitive regions by identifying locally redundant signals within same genomic windows, and removing inconsistent or spurious SV signals arising from ambiguous alignments in repetitive contexts and (3) merging same-type SV signals’ heterogeneous breakpoints from the same alignments record when their distances fall below a preset threshold (default 500 bp). All SV breakpoints are normalized to a unified coordinate system based on the linear reference, by adjusting for the orientation of their originating graph nodes. Finally, collected SV signals undergo clustering and calling steps identical to the pangenome-guided mode, with results output in VCF format.

#### Graph augment module

SVPG integrates multiple tools to achieve rapid graph augment (Supplementary Fig. 10). Users can provide input through either an organized workspace containing sample sequencing files or a comprehensive file list documenting paths to all sequencing data. For each sample, the workflow begins with minigraph alignment of raw sequencing data to the original pangenome, followed by implementation of the SVPG pangenome-based mode to call de novo SVs. Population-level SVs undergo systematic integration through a multi-step process. First, multi-sample VCFs are merged using the ‘bcftools merge’^44^, and redundant variants are eliminated through ‘Truvari collapse’. Subsequently, these SVs are incorporated into the linear-reference genome (the initial haplotype used to construct the original pangenome) using ‘bcftools consensus’, generating a haplotype sequence. Before augmentation, uncertain nucleotides (‘N’) in the sequence are removed to improve alignment efficiency. The resulting haplotype sequence, serving as a carrier for all de novo SVs, is ultimately integrated into the original pangenome graph via ‘minigraph -cxggs’, completing the graph augment process.

### Benchmarking methodology

#### Benchmarking SV callers on GIAB ground truths and complex regions

Based on the latest GIAB release, we downloaded HG002 sample raw sequencing reads (HiFi and ONT) alignment data based on the GRCh37 reference genome, and applied SVPG (v.1.4.1), Sniffles2 (v.2.3.3), cuteSV (v.2.1.1), SVIM (v.1.4.1), DeBreak (v.1.2), Sawfish (v.2.0.3), miniSV (v.r29) and SVarp (v.1.0) to perform SV calling. SVPG, miniSV and SVarp adopt the GRCh37-91c.r559 version pangenome reference release by HPRC as input. SVarp outputs ‘svtigs’ of local assemblies of SV alleles; we aligned these svtigs to the GRCh37 reference using minimap2 and subsequently applied SVIM-asm^45^ to generate a VCF file with the SV breakpoints. Except for Sniffles2, DeBreak and Sawfish, other tools used the same supporting read threshold for low-quality call filtering (Supplementary Note 3 for command line details).

SV calling results for the HG002 sample were uniformly evaluated using Truvari^46^ (v.5.3), against three GIAB-released ground truth sets (HG002_SVs_Tier1_v0.6, HG002_GRCh37_CMRG_SV_v1.00 and GRCh37_HG2-T2TQ100-V1.1), with the ‘--includebed’ parameter specifying high-confidence regions and complex genomic regions.

Complex graph regions were created using custom scripts. The script traverses the pangenome reference graph along linear-reference genome coordinates with a 5,000-bp window size, counting the number of nodes in each window. If the node count >3, the window-covered region is defined as a complex graph region. Q100-hard regions were constructed by subtracting the SV v.0.6 tier 1 regions from the Q100 confident regions using the ‘bedtools subtract’ command. The remaining genomic intervals, which are not covered by v.0.6 tier 1 but still within the Q100 confident regions, were designated as harder evaluation regions.

#### Mendelian inconsistency analysis

We selected two family datasets, Ashkenazim and Chinese family trio datasets for Mendelian inconsistency analysis. The Ashkenazim trio sample data directly used GIAB-released HiFi alignment data. Chinese trio samples lacked GIAB-provided HiFi platform alignment results; therefore, we used minimap2 (parameters -ax map-hifi) and pbmm2 (Sawfish strictly requires pbmm2 result as input) to align raw HiFi reads to the GRCh37 reference genome. In the evaluation, we selected all SV records >50 bp from the VCF file of each family for consistency verification. Specifically, for the same SV record within the family (with a breakpoint distance <1,000 bp), we extracted genotype trios (offspring, father and mother) and verified their consistency according to Mendelian inheritance rules. For example, when the genotypes of both parents are 0/0, the genotype of the offspring must be 0/0; when one parent is 0/0 and the other is 1/1, the genotype of the offspring must be 0/1. Finally, we calculated the Mendelian inconsistency rate by dividing the number of inconsistent records by the total number of records.

#### Benchmarking the performance of SV caller using replicates

We assessed the inconsistency of replicate experiments using multiple datasets from HG002 and HG005 samples. For the 48× ONT alignment data of HG002, we performed downsampling using ‘samtools view’ with default and 99 random seeds to construct paired datasets at 20×, 10× and 5× coverage. For the HG005 sample, we selected two independent 10× coverage HiFi sequencing datasets and aligned the raw reads to the GRCh37 reference genome using minimap2. In the reproducibility assessment, we used scripts similar to those used in Mendelian consistency analysis, extracting genotypes from control and case groups for each identical SV record and checking their concordance. It is important to note that inconsistency does not always imply incorrect SV calls. Different callers may report the same true SV with slightly shifted breakpoints or alternative representations, especially in repetitive or microhomology regions. To reduce the risk of misclassifying such cases as false positives, we allowed a breakpoint tolerance of 500 bp and required a size concordance ratio (min–max) of at least 0.7 when comparing SVs across replicates. Only when both conditions were violated did we classify events as inconsistent. Finally, the inconsistency rate was calculated by dividing the number of discordant records by the total number of records.

In addition, we incorporated SURVIVOR^47^, a third-party SV merging tool, to calculate Jaccard similarity for further validation of our assessment accuracy. Specifically, we merged VCF files from two replicate experiments using parameters ‘1000 2 1 1 0 50’ and ‘1000 1 1 1 0 50’, then computed Jaccard similarity (A/B) based on the intersection (A) and union (B) of SV records from both files.

#### Benchmarking SV call consistency across 20 samples

HPRC has released raw sequencing data for more than 300 samples. From this dataset, we selected 20 samples that were not included in the draft pangenome reference. The HiFi raw reads were aligned to the GRCh38 reference genome using minimap2 and then processed with SVPG in pangenome-guided mode and Sniffles for SV calling; for Sawfish, we used pbmm2 alignments to meet the tool’s specific requirements. SV call results across the 20 samples were subsequently merged using Jasmine^48^. For each merged site, the number of supporting samples was extracted from the SUPP (sample support count) field of the merged VCF, and the proportion of calls for each SUPP value (SUPP_call_count/total_call_count) was calculated. As for the proportion of HWE pass-rate, it was calculated using ‘bcftools fill-tags’.

#### Rare SV detection benchmarking in simulated data

To assess rare SV detection performance, we designed a pipeline to extract 66,197 rare SVs^33^ from the National Human Genome Research Institute repository to simulate a ‘super-variant’. Initially, we filtered 95,000 rare INDELs (duplications were treated as insertions) from the original SV callset using two criteria: NSAMP and NFAM = 1, identifying SVs unique to single individuals or families among 14,623 individuals. Subsequently, we removed overlapping SVs, randomly added base sequences of corresponding variant lengths to <ALT> fields of insertion, and for deletions, retrieved base sequences from reference genome breakpoint positions for the <REF> fields. Following this preprocessing, we established 66,197 independent rare SVs as our validation benchmark. We then integrated these SVs into the GRCh38 reference genome using ‘bcftools consensus’ to construct the ‘super-variant’. Based on this variant reference sequence, we simulated 20× coverage ONT and HiFi raw sequencing reads using PBSIM3^49^, aligned them to the pangenome reference (GRCh38-90c.r518 version) using minigraph, generating graph-alignment results. Finally, these alignments were input to SVPG, miniSV and SVarp for SV calling; evaluation result from the Truvari summary output files.

#### Somatic SV detection benchmarking

We obtained high-coverage HiFi sequencing data for HG008 and its matched control from GIAB and aligned them to the GRCh38-90c.r518 version pangenome reference using minigraph. The resulting graph-alignment files underwent independent SV calling through SVPG. Subsequently, we identified cancer-specific SVs through subtracted the normal sample SV calls from the tumor sample calls via our custom script, which filtered VCF files according to defined overlap criteria: SVs in the cancer sample were classified as cancer-specific if their breakpoint did not overlap with that of a normal sample SV within a 1,000 bp window, otherwise it was considered nonspecific. For miniSV, we followed the ‘tumor-normal pairs’ mode as documented on GitHub to identify specific SVs. For SVarp and Sniffles2, we used the same SV subtraction procedure as SVPG. Manta^50^, Severus and nanomonsv directly generated tumor-specific SVs from paired tumor–normal alignment files. Overall performance assessment employed Truvari and minda (commit 182fbcf), while SV-type-based evaluation was performed using a custom evaluation script. The script categorizes variants according to the SVTYPE field in the VCF and currently evaluates three primary SV classes: INDELs (with duplications mapped to insertions), inversions and translocations. Matching criteria and thresholds are defined to be consistent with those used by Truvari. Supplementary Fig. 11 shows the custom script shows high concordance with Truvari-based evaluations. Before assessment, VCFs with double-entry breakends were preprocessed to convert them to single representation. For the COLO829 and HCC1395 samples, the calling workflow was performed in the same pipeline as HG008.

#### Graph augmentation benchmarking in 20 HPRC samples

We used the HiFi raw reads from the 20 HPRC samples mentioned above and input them into the SVPG graph augment mode. For de novo assembly-based graph augment, an automated pipeline was used: hifiasm was used to assemble haplotype-resolved sequences for the 20 samples, which were then iteratively integrated into the original pangenome using minigraph. Basic statistics for graphs were compiled using ‘gfatools stat’, with bubble overlap counts determined using ‘bedtools intersect’ (with ‘gfatools bubble’ converting graph bubbles into BED format). In addition, HG002 HiFi sequencing data were aligned to both original and augmented graphs, and alignment statistics were generated using custom script.

### Reporting summary

Further information on research design is available in the Nature Portfolio Reporting Summary linked to this article.

## Online content

Any methods, additional references, Nature Portfolio reporting summaries, source data, extended data, supplementary information, acknowledgements, peer review information; details of author contributions and competing interests; and statements of data and code availability are available at https://doi.org/10.1038/s41592-026-03219-2.

## Supplementary information

Supplementary Information Supplementary Figs. 1–11 and Notes 1–3.

Reporting Summary

Peer Review File

Supplementary Tables Supplementary Tables 1–7.

## Source data

Source Data Fig. 2 Statistical source data.

Source Data Fig. 3 Statistical source data.

Source Data Fig. 4 Statistical source data.

Source Data Fig. 5 Statistical source data.

## Data Availability

Sequencing and alignment data are as follows: GIAB HG002 HiFi data are publicly available via the National Center for Biotechnology Information (NCBI) at https://ftp-trace.ncbi.nlm.nih.gov/giab/ftp/data/AshkenazimTrio/HG002_NA24385_son/PacBio_HiFi-Revio_20231031/; HG002 ONT available via NCBI at https://ftp-trace.ncbi.nlm.nih.gov/giab/ftp/data/AshkenazimTrio/HG002_NA24385_son/UCSC_Ultralong_OxfordNanopore_Promethion/; Ashkenazim trio available via NCBI at https://ftp-trace.ncbi.nlm.nih.gov/giab/ftp/data/AshkenazimTrio/; Chinese trio available via NCBI at https://ftp-trace.ncbi.nlm.nih.gov/giab/ftp/data/ChineseTrio/; HG008 HiFi available via NCBI at https://ftp-trace.ncbi.nlm.nih.gov/giab/ftp/data_somatic/HG008/Liss_lab/PacBio_Revio_20240125/; HG008 Illumina available via NCBI at https://ftp-trace.ncbi.nlm.nih.gov/giab/ftp/data_somatic/HG008/Liss_lab/BCM_Illumina-WGS_20240313/; HCC1395 and COLO829 available via PacBio at https://www.pacb.com/connect/datasets/ and 20 HPRC samples available via Amazon AWS at https://s3-us-west-2.amazonaws.com/human-pangenomics/index.html?prefix=working/HPRC/. Benchmark sets are available as follows: HG002 genome wide available via NCBI at https://ftp-trace.ncbi.nlm.nih.gov/ReferenceSamples/giab/release/AshkenazimTrio/HG002_NA24385_son/NIST_SV_v0.6/ and https://ftp-trace.ncbi.nlm.nih.gov/ReferenceSamples/giab/data/AshkenazimTrio/analysis/NIST_HG002_DraftBenchmark_defrabbV0.019-20241113/; HG002 medical regions available via NCBI at https://ftp-trace.ncbi.nlm.nih.gov/ReferenceSamples/giab/release/AshkenazimTrio/HG002_NA24385_son/CMRG_v1.00/; rare SVs available via GitHub at https://github.com/hall-lab/sv_paper_042020/blob/master/Supplementary_File_1.zip; repeats region available via USCS at https://genome.ucsc.edu/cgi-bin/hgTables; Human Genome Structural Variation Consortium 3 available via 1000 Genomes at https://ftp.1000genomes.ebi.ac.uk/vol1/ftp/data_collections/HGSVC3/release/Variant_Calls/1.0/GRCh38/; HG008 available via NCBI at https://ftp-trace.ncbi.nlm.nih.gov/ReferenceSamples/giab/data_somatic/HG008/Liss_lab/analysis/NIST_HG008-T_somatic-stvar_DraftBenchmark_V0.3-20250220/; COLO829 available via Zenodo at https://doi.org/10.5281/zenodo.13917379 (ref. ^51^); consensus benchmark set of HCC1395 available via Springer at https://static-content.springer.com/esm/art%3A10.1186%2Fs13059-022-02816-6/MediaObjects/13059_2022_2816_MOESM4_ESM.xlsx. and the validation benchmark set of HCC1395 available via Springer at https://static-content.springer.com/esm/art%3A10.1186%2Fs13059-022-02816-6/MediaObjects/13059_2022_2816_MOESM5_ESM.xlsx. Reference genome are available as follows: GRCh38 available via 1000 Genomes at http://ftp.1000genomes.ebi.ac.uk/vol1/ftp/technical/reference/GRCh38_reference_genome/; GRCh37 available via 1000 Genomes at http://ftp-trace.ncbi.nih.gov/1000genomes/ftp/technical/reference/phase2_reference_assembly_sequence/ and GRCh38-90c.r518 and GRCh37-91c.r559 available via Zenodo at https://zenodo.org/records/10693675 (ref. ^52^). The VCF outputs of all tools, augment pangenome graph and benchmark scripts are available via Zenodo at https://doi.org/10.5281/zenodo.18456502 (ref. ^53^). Source data are provided with this paper.

## Code availability

Source code for SVPG is available via GitHub at https://github.com/coopsor/SVPG and via Zenodo at https://doi.org/10.5281/zenodo.19754620 (ref. ^54^).
