# Supplementary material for: SVPG: a pangenome-based structural variant detection approach and rapid augmentation of pangenome graphs with new samples
Source: Nat Methods. 2026 Sep 21;23(10):1984–93. doi: 10.1038/s41592-026-03219-2 (PMC13645633; doi:10.1038/s41592-026-03219-2)
Supplement: Supplementary file 1 [file 41592_2026_3219_MOESM1_ESM.pdf]

nature methods

Supplementary information https://doi.org/10.1038/s41592-026-03219-2

SVPG: a pangenome-based structural variant detection approach and rapid augmentation of pangenome graphs with new samples

In the format provided by the authors and unedited

SVPG: A pangenome-based structural variant detection approach and rapid augmentation of pangenome graphs with new samples

Supplementary Figures

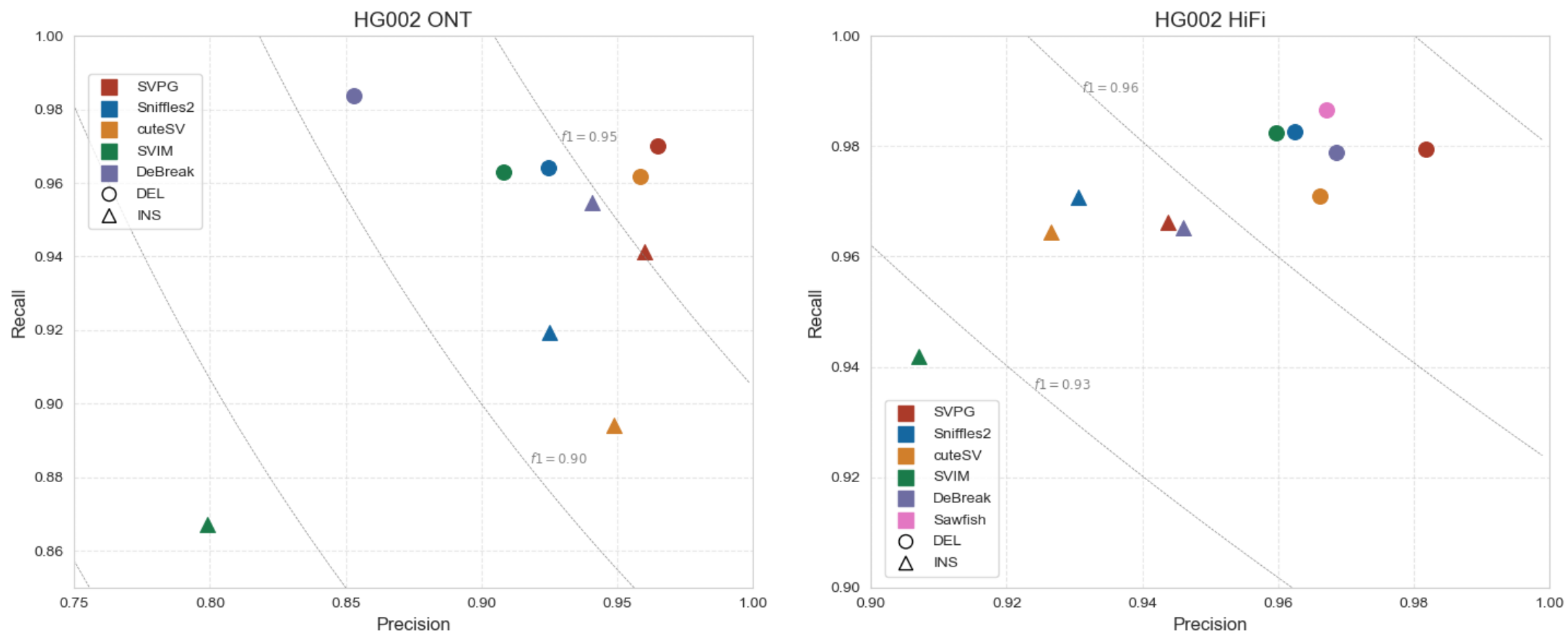

Supplementary Fig 1. SV calling performance across different SV types on HG002 47× ONT and 48× HiFi datasets. Measured by using the Truvari for the evaluation of each caller on the GIAB-tier1 ground truth. Sawfish only supports HiFi data, while miniSV and SVarp were excluded due to their poor performance in the GIAB benchmarking.

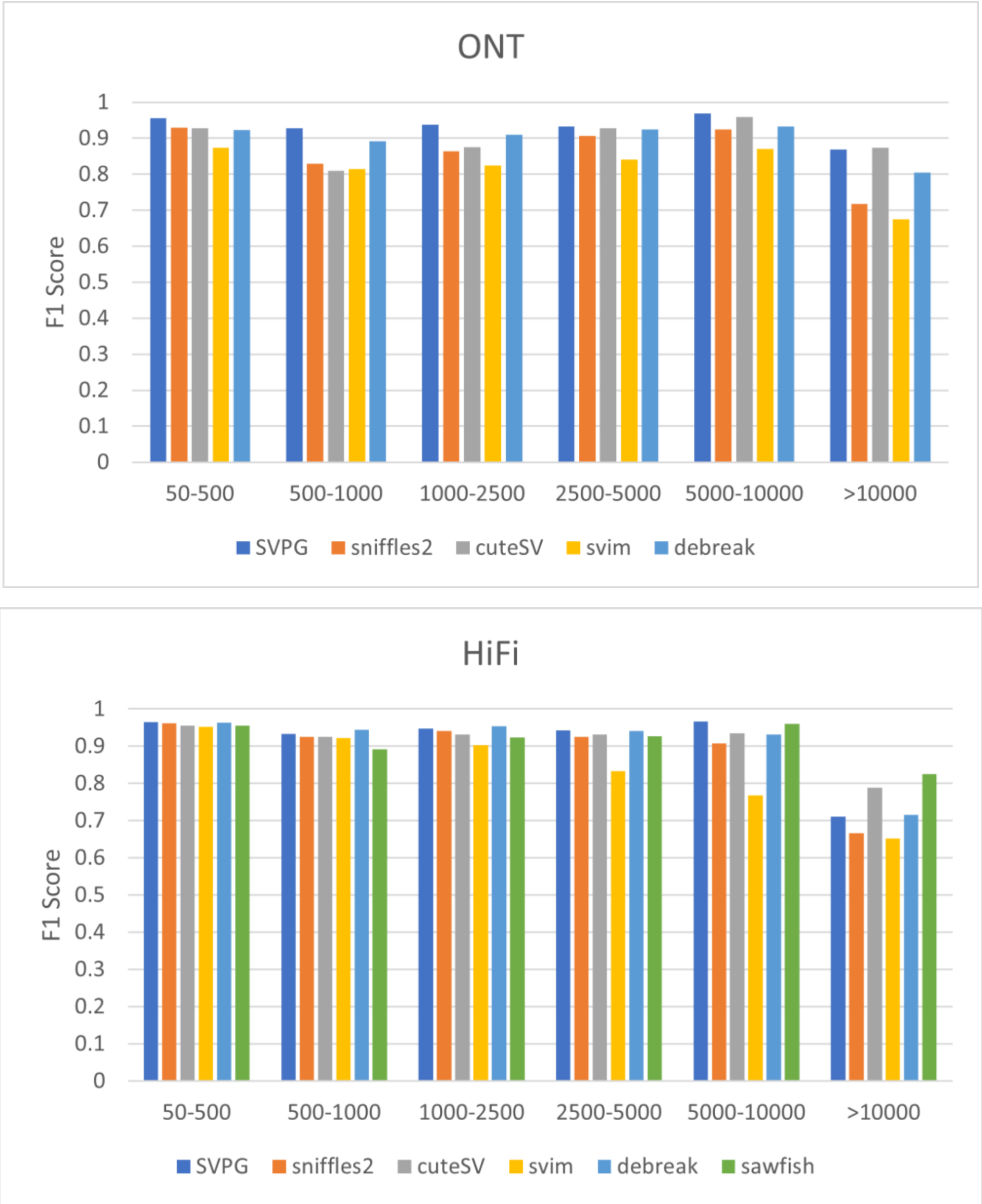

Supplementary Fig 2. F1 measure across different SV length on HG002 47× ONT and 48× HiFi datasets. Measured by using the Truvari for the evaluation of each caller on the GIAB-tier1 ground truth. Sawfish only supports HiFi data, while miniSV and SVarp were excluded due to their poor performance in the GIAB benchmarking

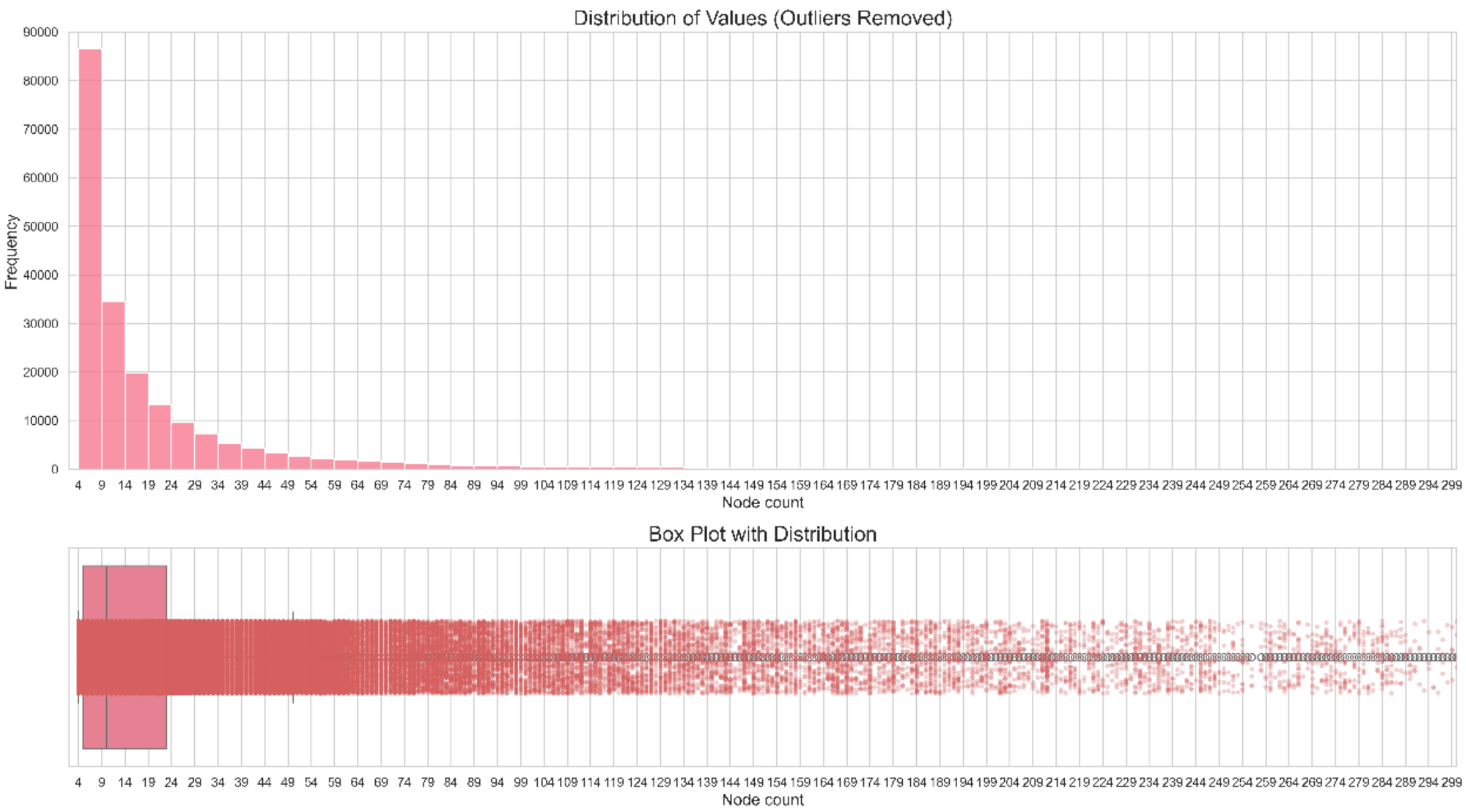

Supplementary Fig 3. The distribution of complex pangenome graph regions (node count >3) defined by the GRCh37 reference pangenome coordinates. The upper panel shows a histogram of node count distribution with a bin size of 4, while the lower panel presents the corresponding box plot. Statistical analysis excluded outliers above the 0.97 quantile. The box plots show the median (Q2, 50th percentile), first (Q1, 25th percentile) and third quartile (Q3, 75th percentile), and the whiskers encompass observations within 1.5× the interquartile range from the first and third quartiles.

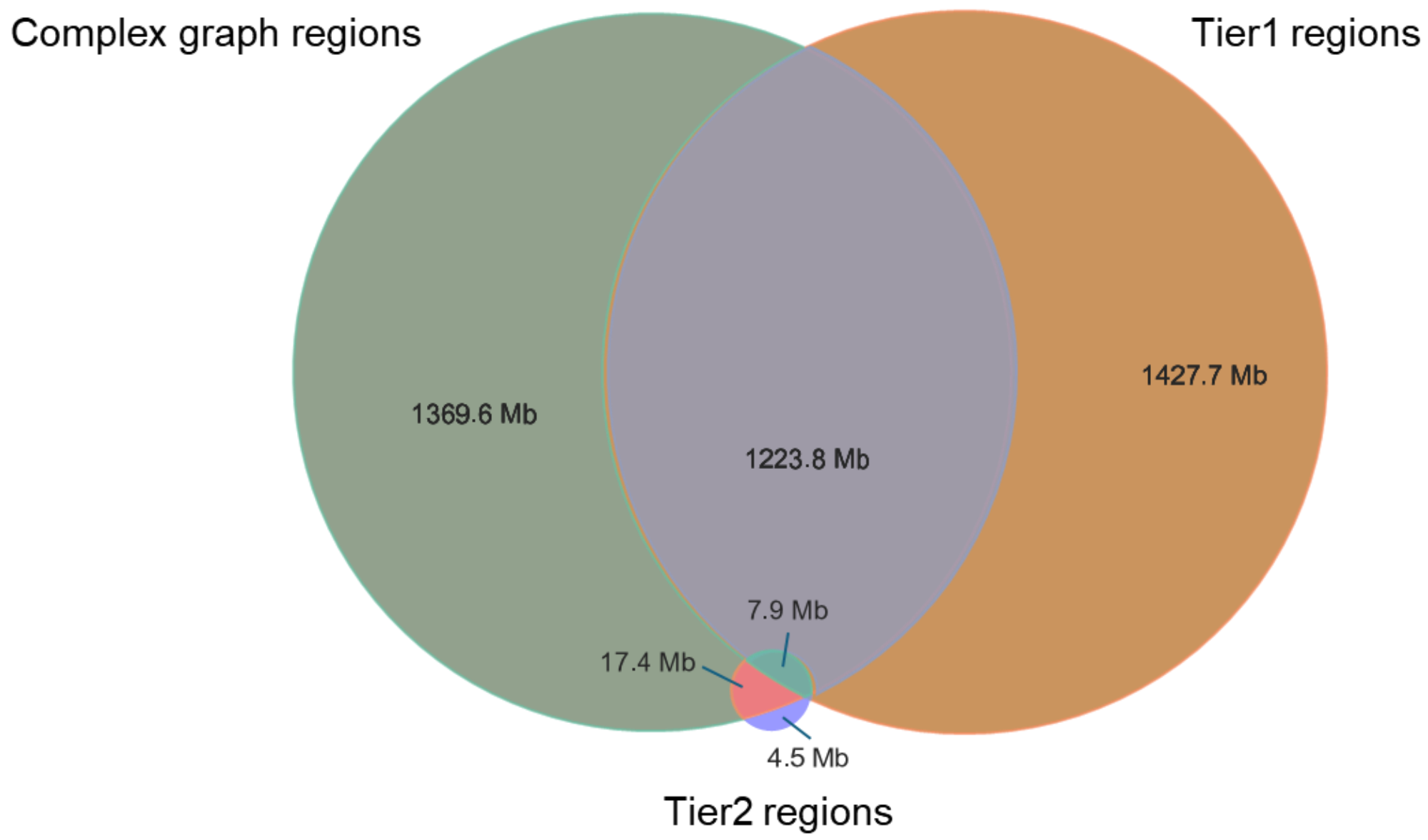

Supplementary Fig 4. The intersection analysis between GIAB high-confidence regions (Tier1 regions) and two types of complex regions: complex graph regions and GIAB-defined complex genomic regions (Tier2 regions). The intersection statistics were generated using bedtools intersect, with results reported in megabase pairs (Mb).

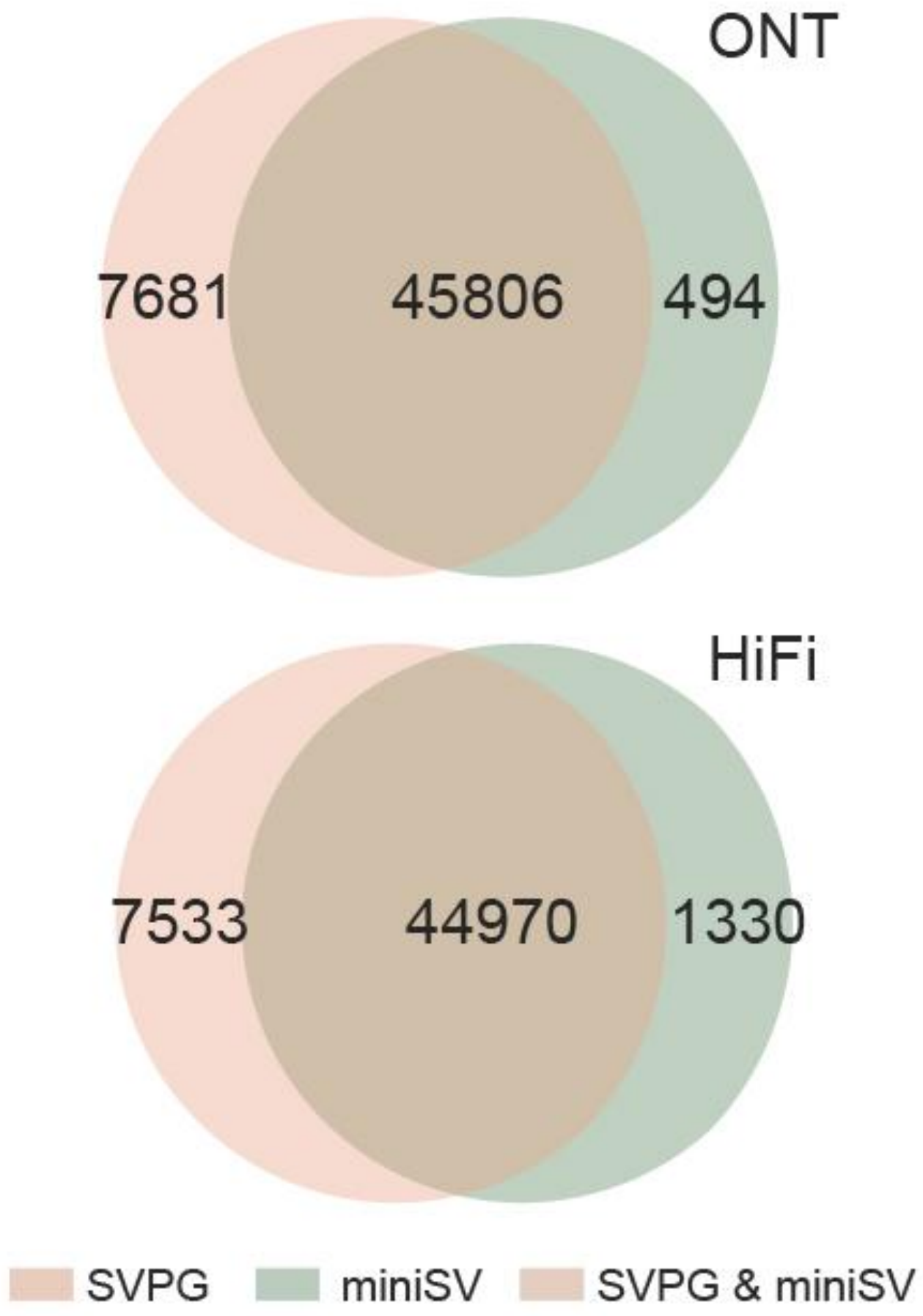

Supplementary Fig 5. Venn diagram of rare true positive SVs detected by SVPG and miniSV on ONT and HiFi simulated rare SVs dataset.

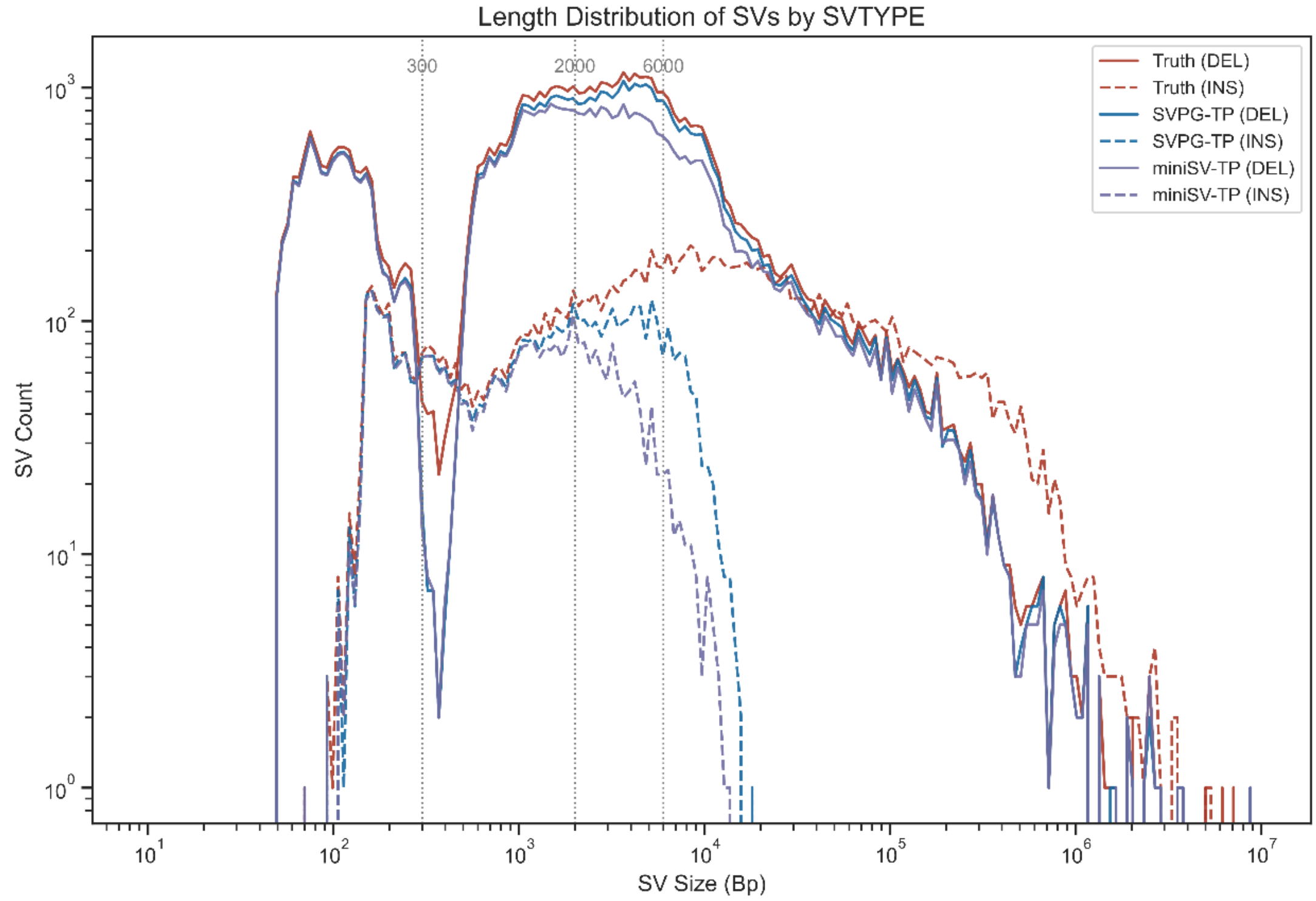

Supplementary Fig 6. Length distribution of rare SV truthset and true positive (TP) SV callsets from SVPG and miniSV. The TP SV callsets from Truvari evaluation result based on ONT dataset. Vertical lines at 300 bp, 2000 bp, and 6000 bp mark commonly observed SV lengths associated with mobile elements. The 300 bp and 6 kb insertions corresponded to Alu and LINE1 elements respectively, the two most abundant classes of transposable elements in the human genome (~11% and ~17% of the genome). The 2 kb SVs represent composite SVA (SINE, Variable Number Tandem Repeat, and Alu) transposons.

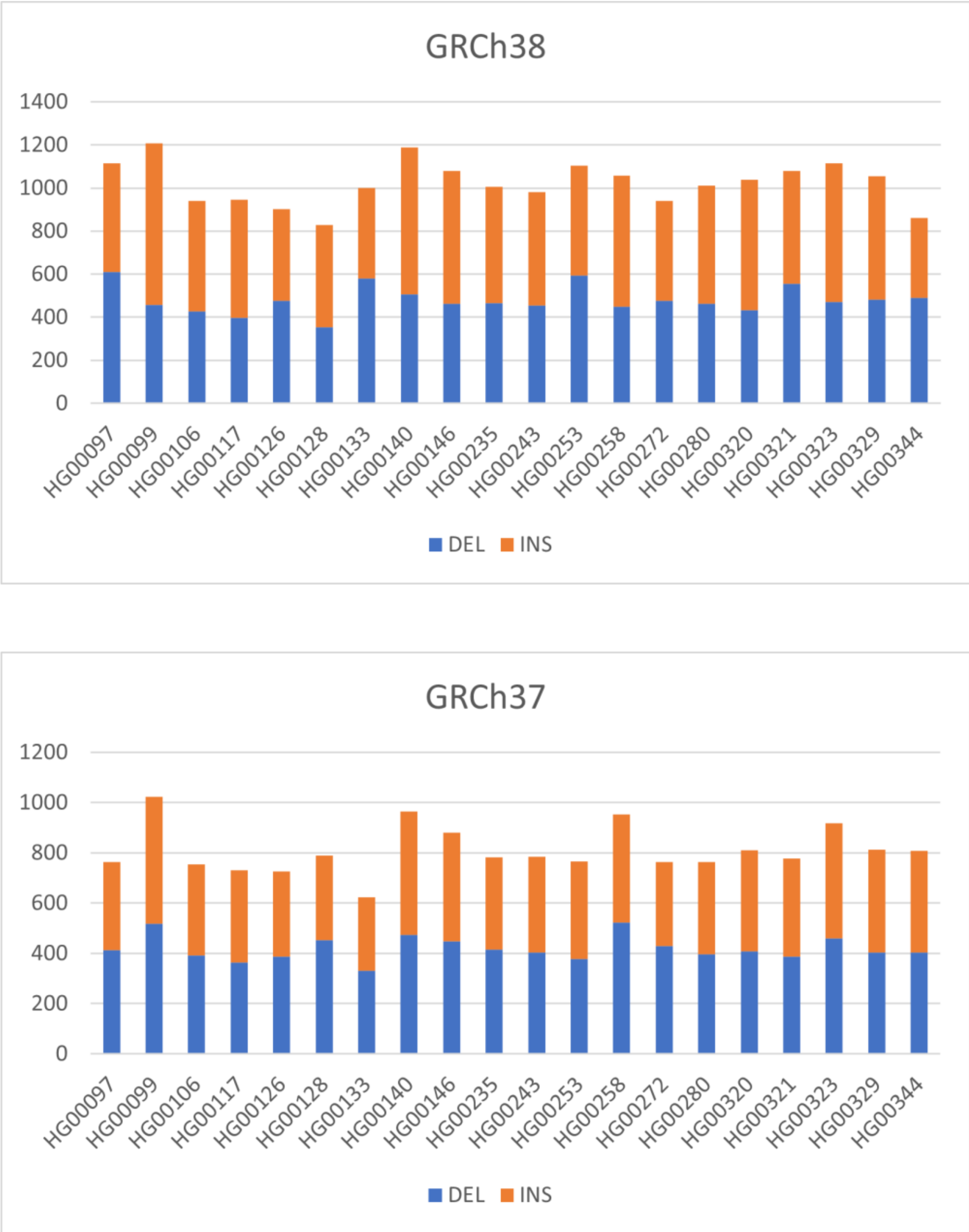

Supplementary Fig 7. Statistics of SV count detected by SVPG across 20 HPRC samples.

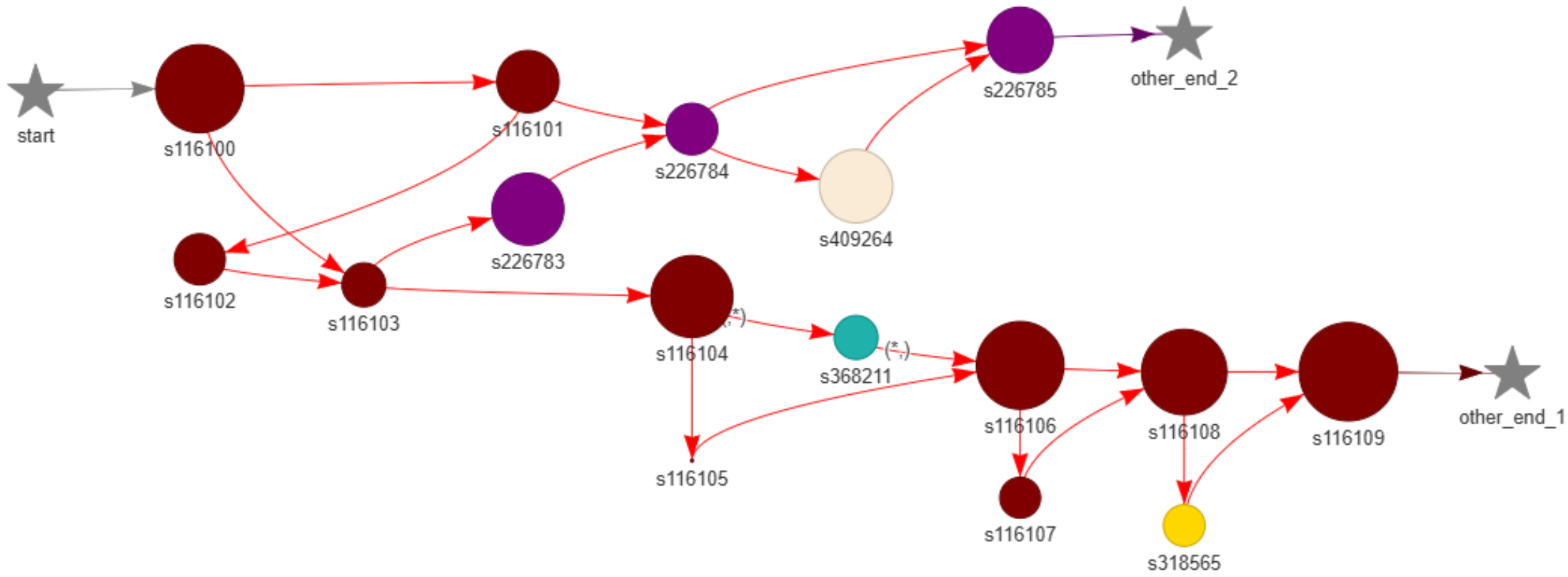

Supplementary Fig 8. Local graph topology at the *MUC6* locus of hifiasm-AUG (17 nodes).

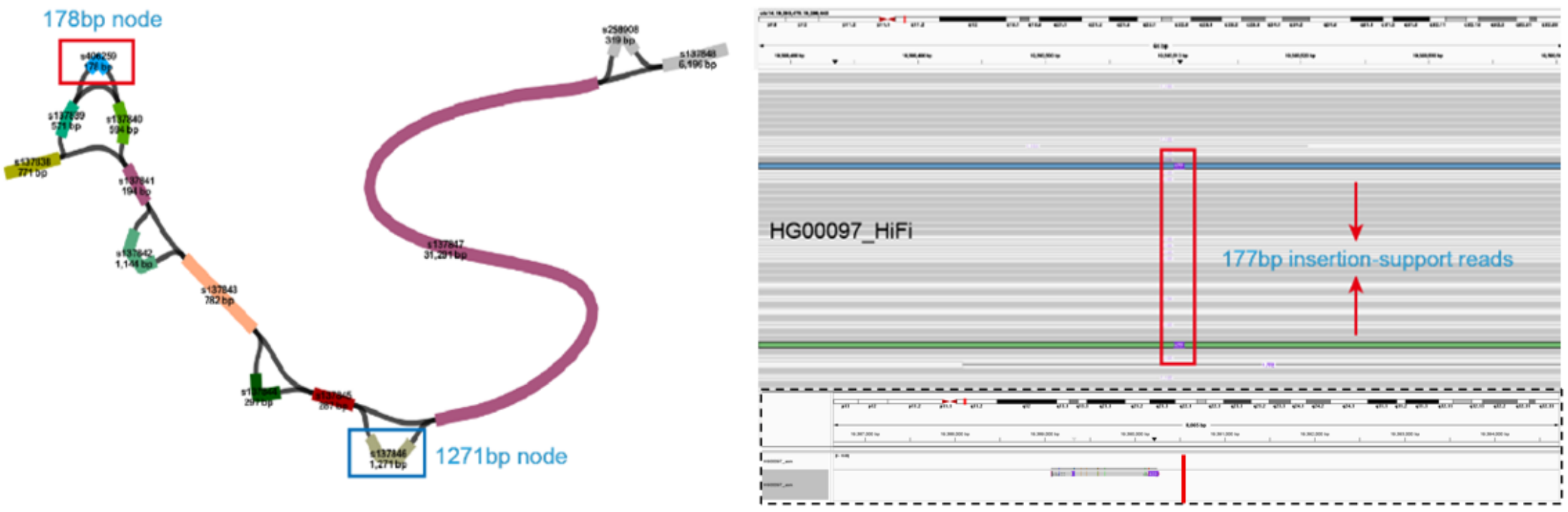

Supplementary Fig 9. Two SVPG-AUG-specific node examples. The Bandage view (left) shows a 178bp insertion at chr14:19,390,510 in HG00097 was identified by SVPG and represented as a bubble structure (labeled red box, node s406359). In an adjacent region, a new 1,271bp node (labeled blue box, s137847) of SVPG-AUG compared to hifiasm-AUG for the same region. By examining the VCF files of 20 new samples called by SVPG, we confirmed this variant originated from the HG00117 sample (chr14:19,393,631-19,394,902). The IGV view (top right) shows read support for the 178bp insertion in HG00097 at the chr14:19,390,510 position, whereas the bottom panel indicates a local assembly failure by hifiasm in this region.

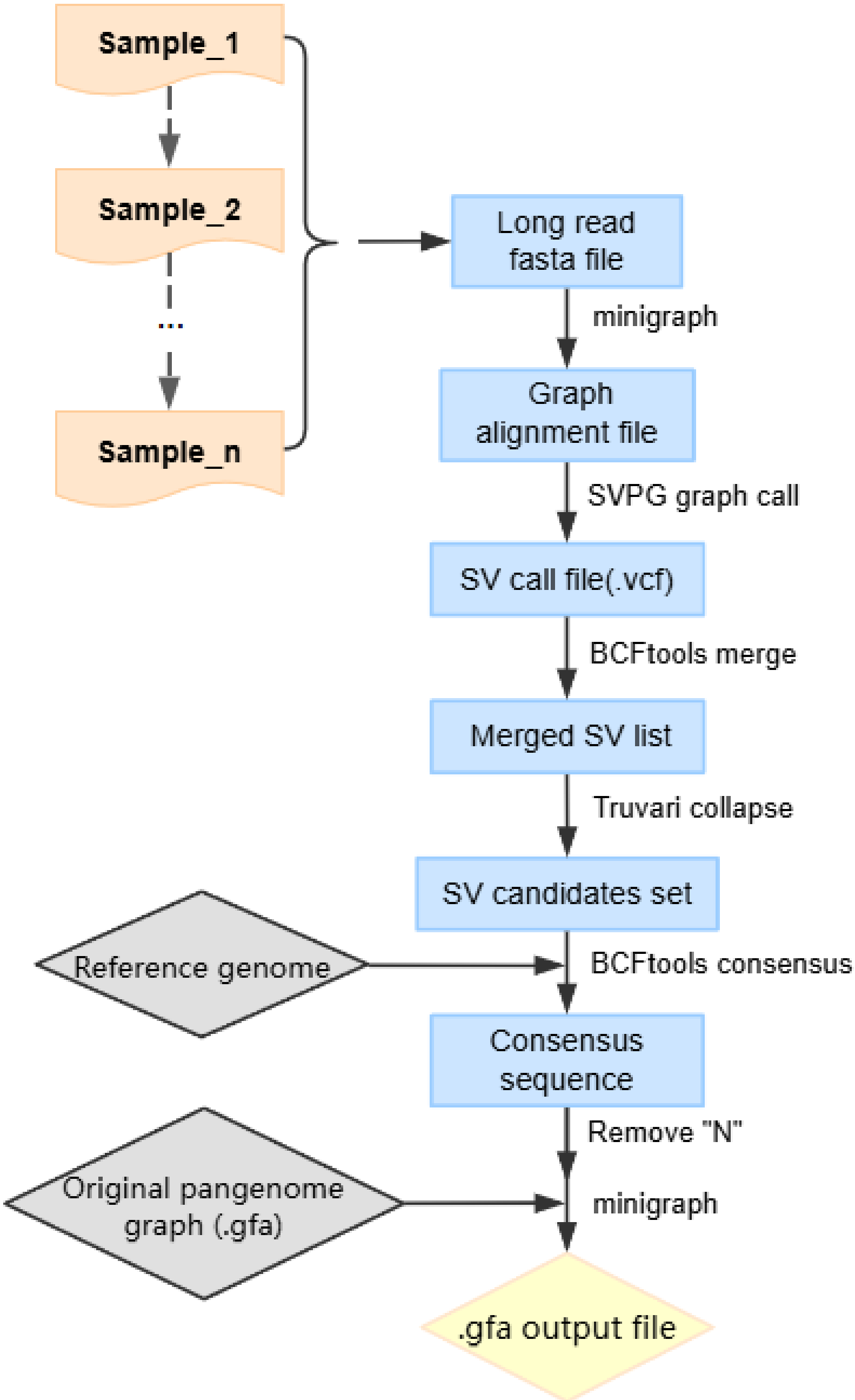

Supplementary Fig 10. Flowchart of the graph augmentation mode in SVPG.

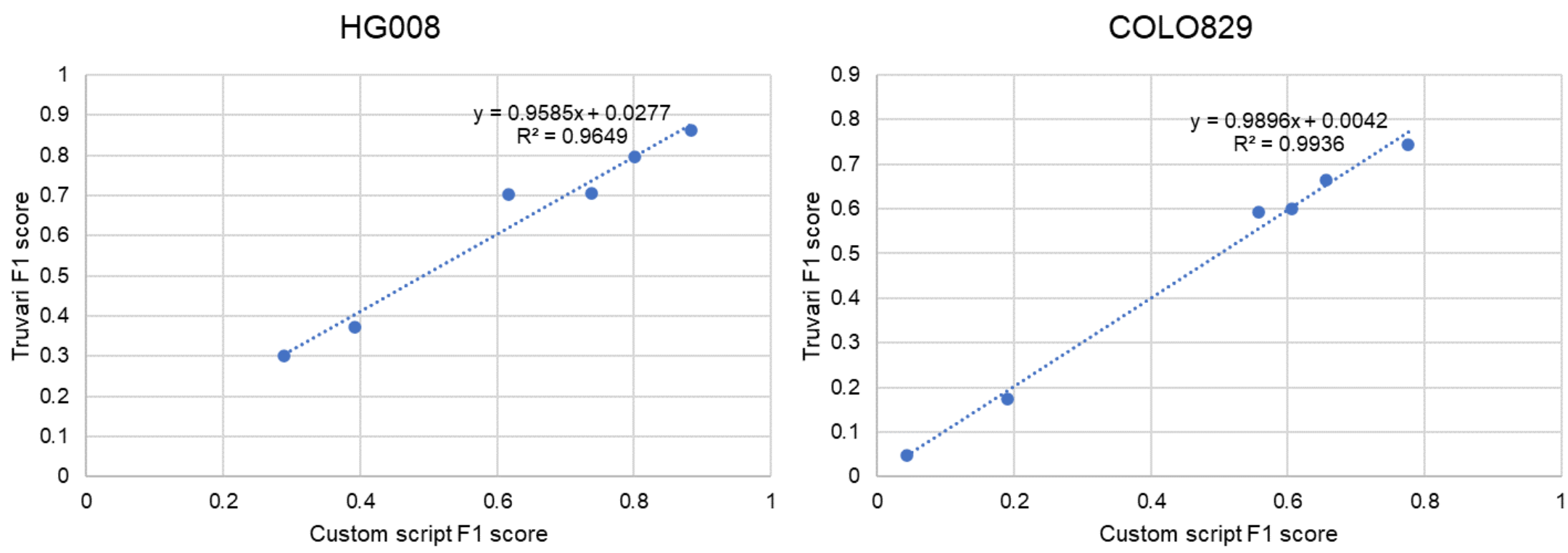

Supplementary Fig 11. Correlation between custom script-derived F1 scores and Truvari F1 scores in benchmark datasets. Scatter plots showing the concordance between F1 scores calculated using the custom evaluation script and those generated by Truvari for the HG008 and COLO829 datasets. Each point represents the performance of a variant call set under different evaluation conditions. Dashed lines indicate linear regression fits, with the corresponding regression equations and coefficients of determination (R^2) shown in each panel.

Supplementary Notes

Supplementary Note 1. Validation of the five *de novo* SVs of SVPG and the 13 *de novo* SVs of Sniffles2

Validation of discordant SVs called by SVPG and Sniffles2 in HG002 sample across chromosomes 1-3, using IGV screenshots and GIAB benchmark set (high-confidence regions). Each IGV snapshot spans the region covering the start and end positions of a *de novo* SV called in HG002, extended by 1000 bp on both sides. At the top of the snapshot, the nearest SV record from the GIAB benchmark set is shown; if no record is found within 1000 bp of the *de novo* SV, it is labeled as "NA". On the left side of the snapshot, SVs called by different tools are displayed for HG002, HG003, and HG004 within the same extended region. For HG003 and HG004, only the closest SV to the *de novo* SV position is displayed; if this SV is more than 1000 bp away, it is also labeled as "NA".

GIAB: Chr1:18599939-18599993 54bp DEL HG003_GT=0/1; HG003_GT=0/0; HG004_GT=0/1

SVPG

Chr1:18599931-18599993

62bp

DEL

0/1

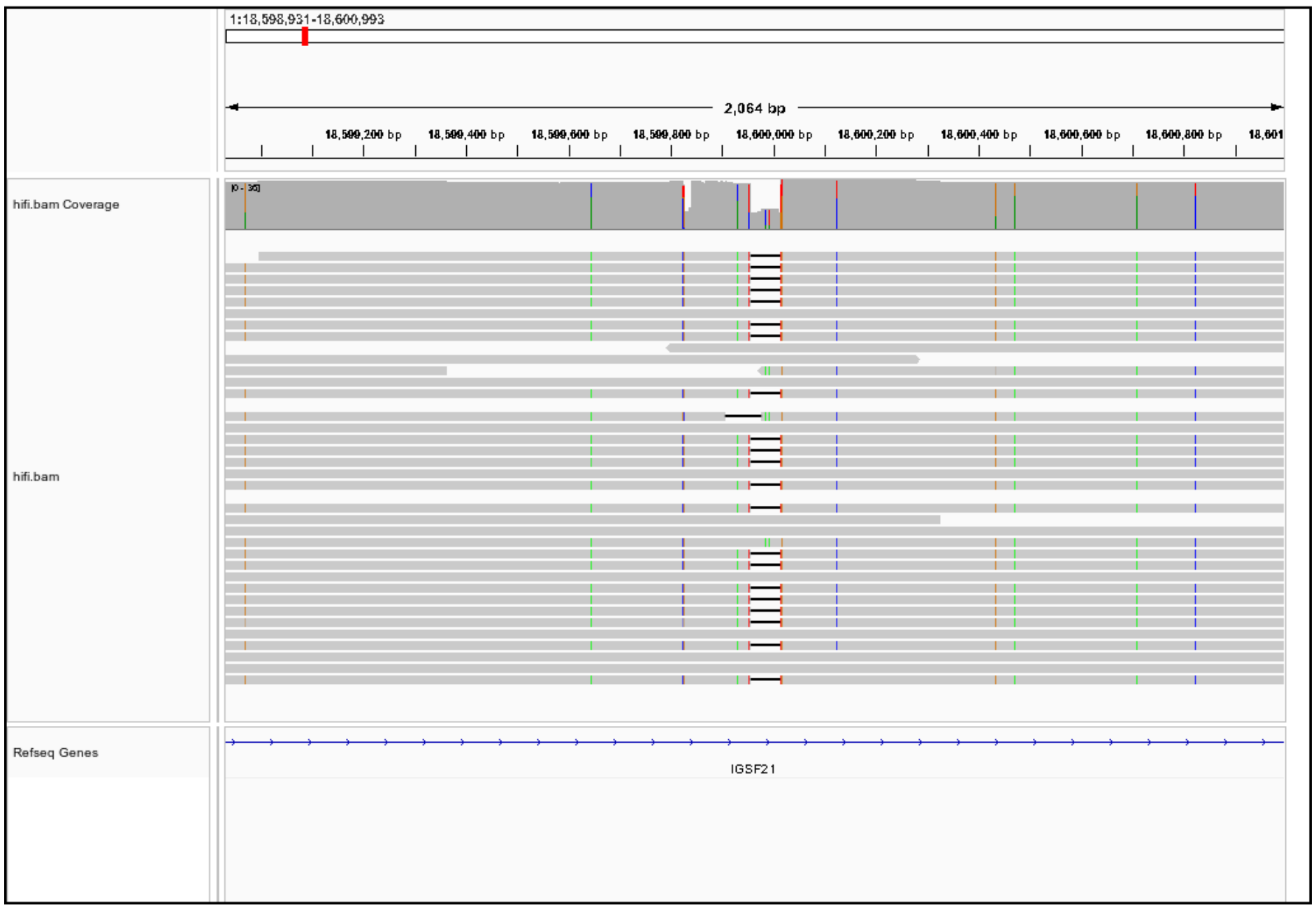

NA

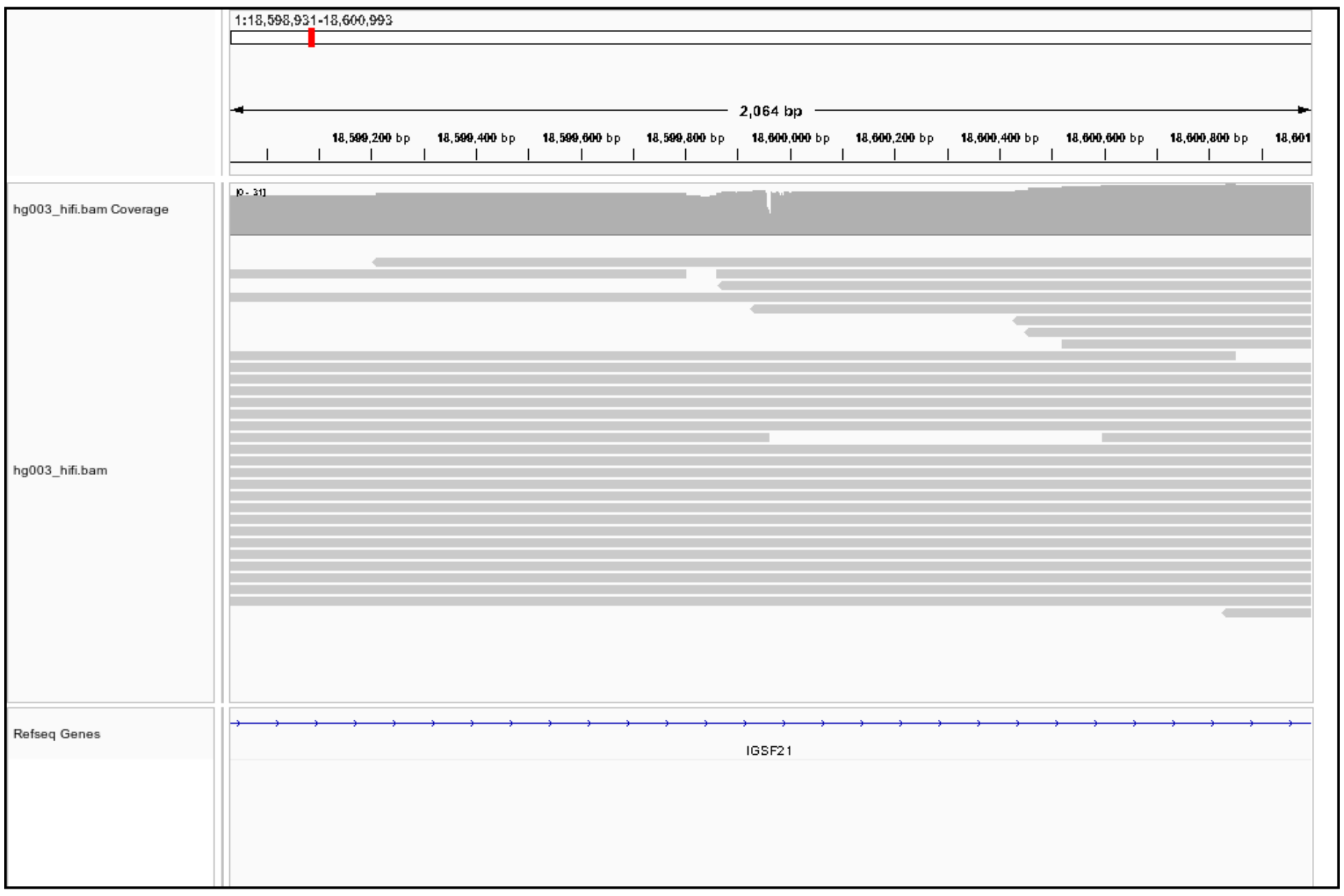

NA

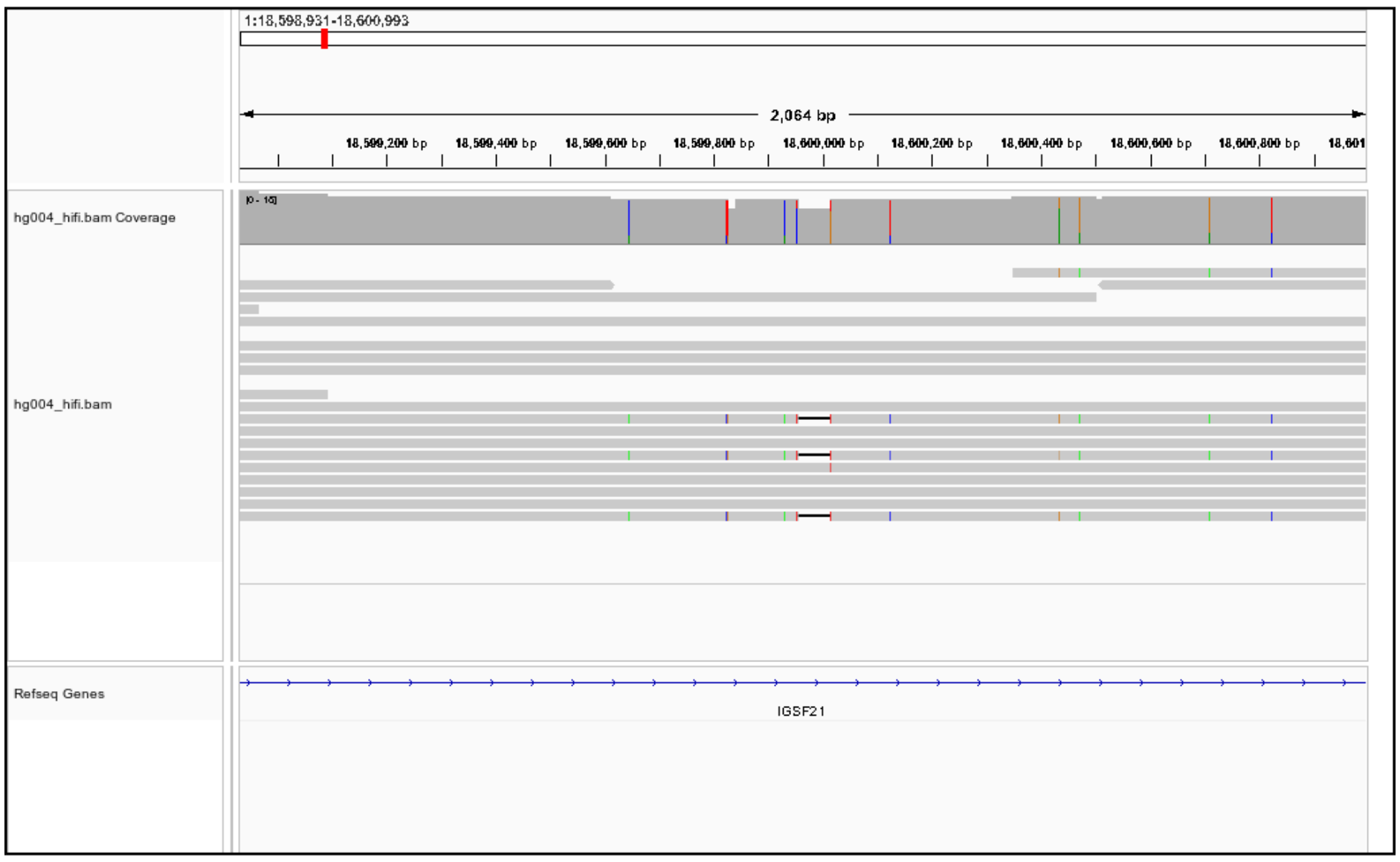

GIAB: Chr2: 232734665 1895bp INS HG002_GT=0/1; HG003_GT=1/1; HG004_GT=1/1

SVPG

Chr2:232734675

2021bp

INS

1/1

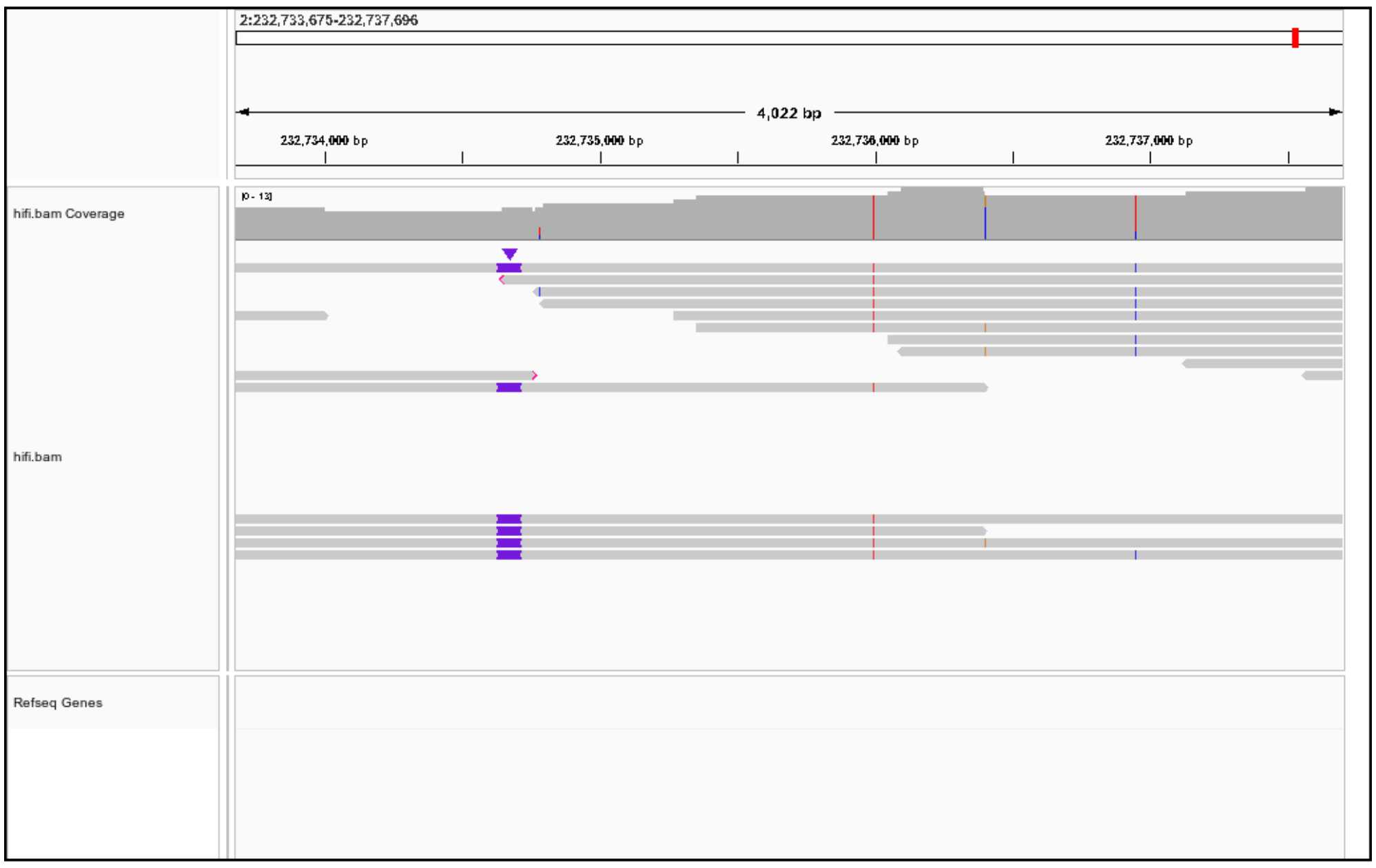

NA

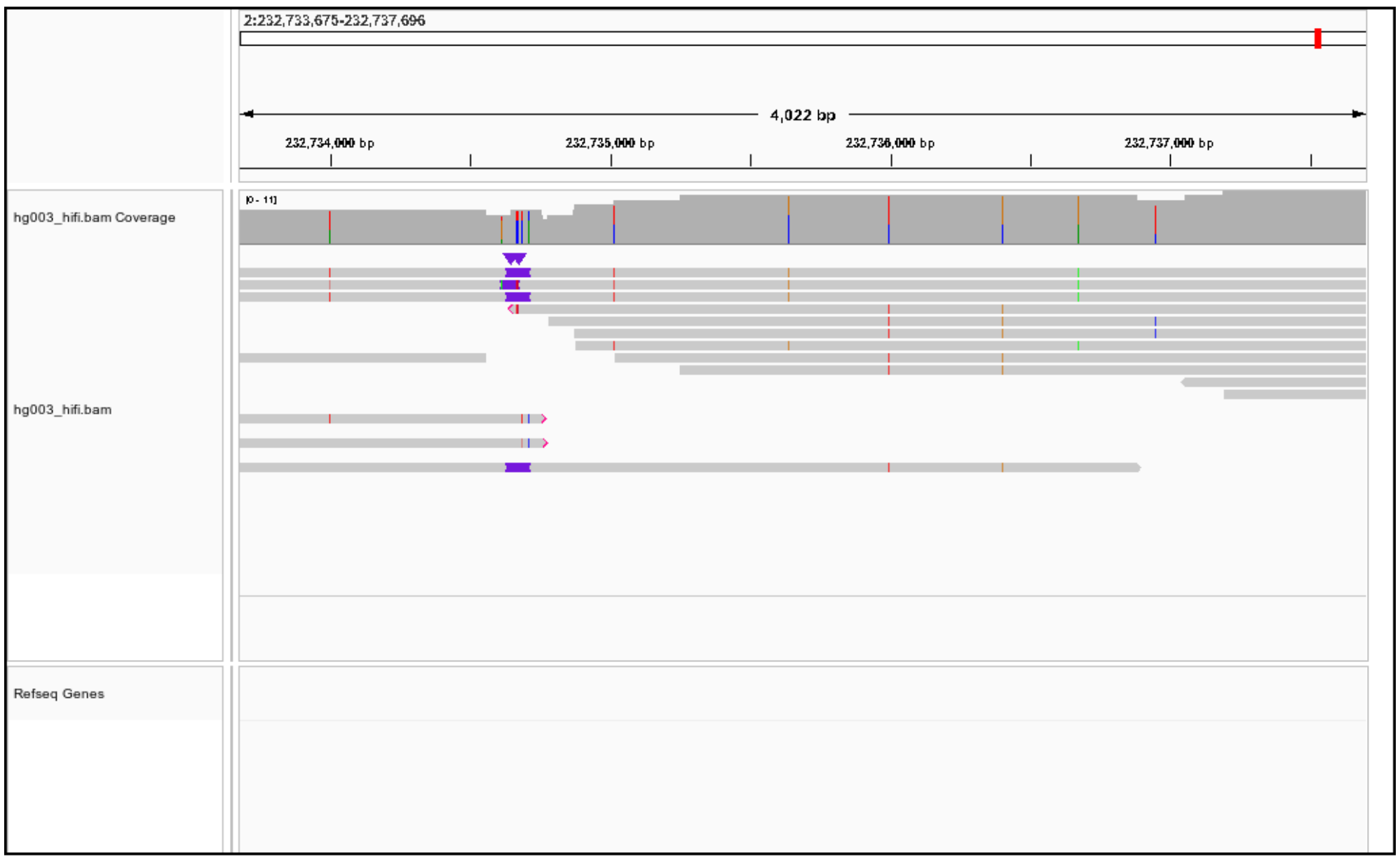

NA

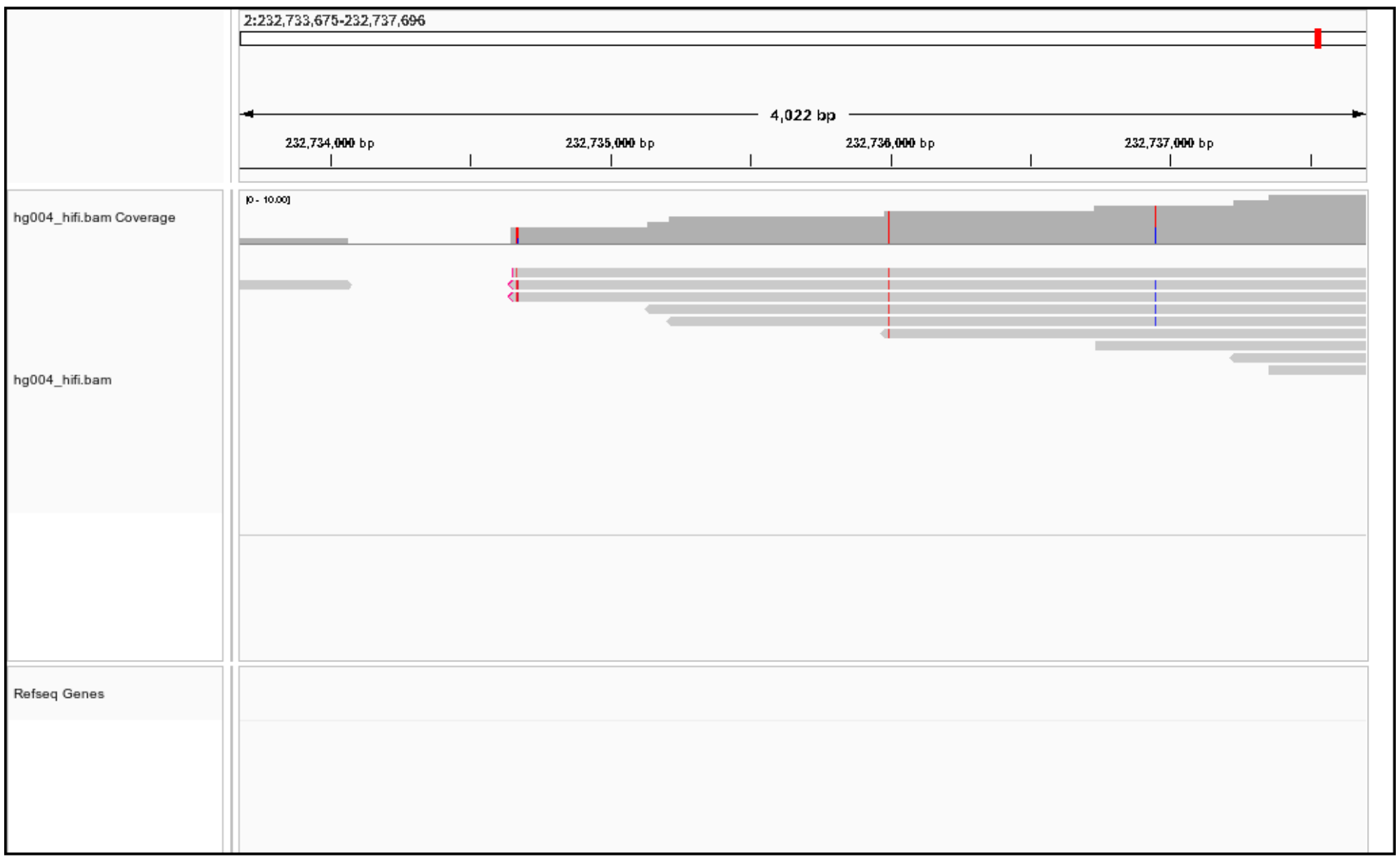

GIAB: NA

SVPG

Chr3:85601516

62bp

INS

0/1

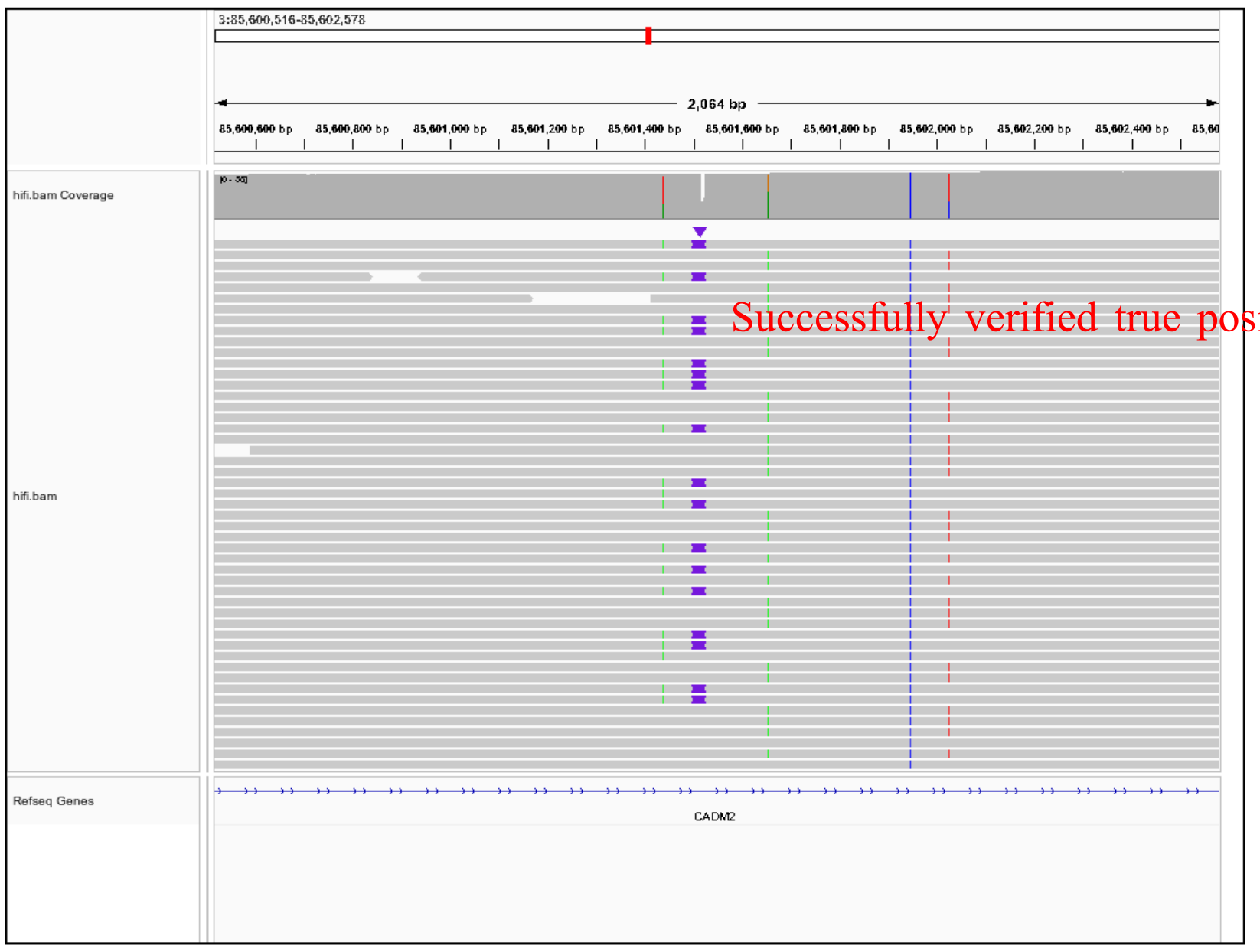

Successfully verified true positive *de novo* SV

NA

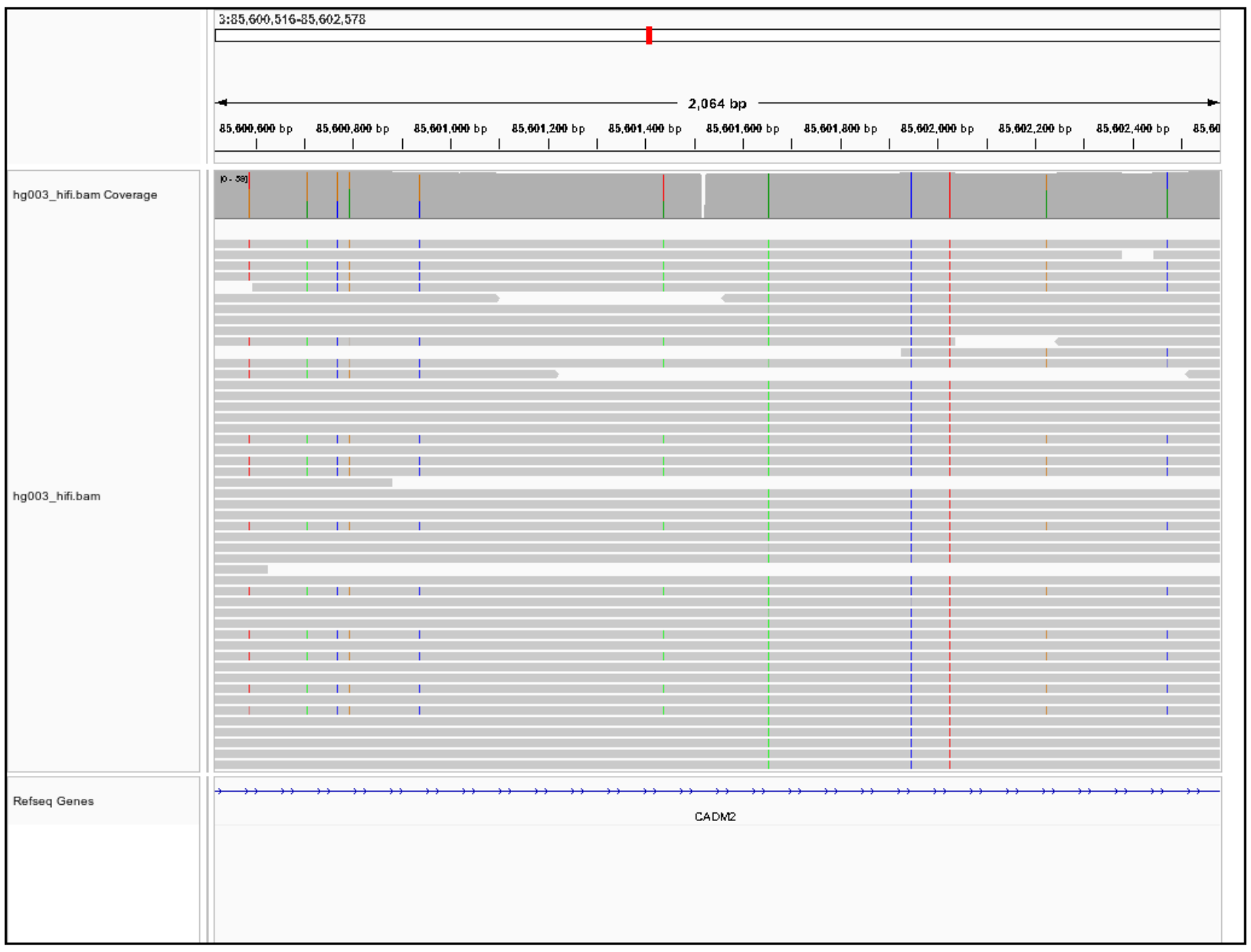

NA

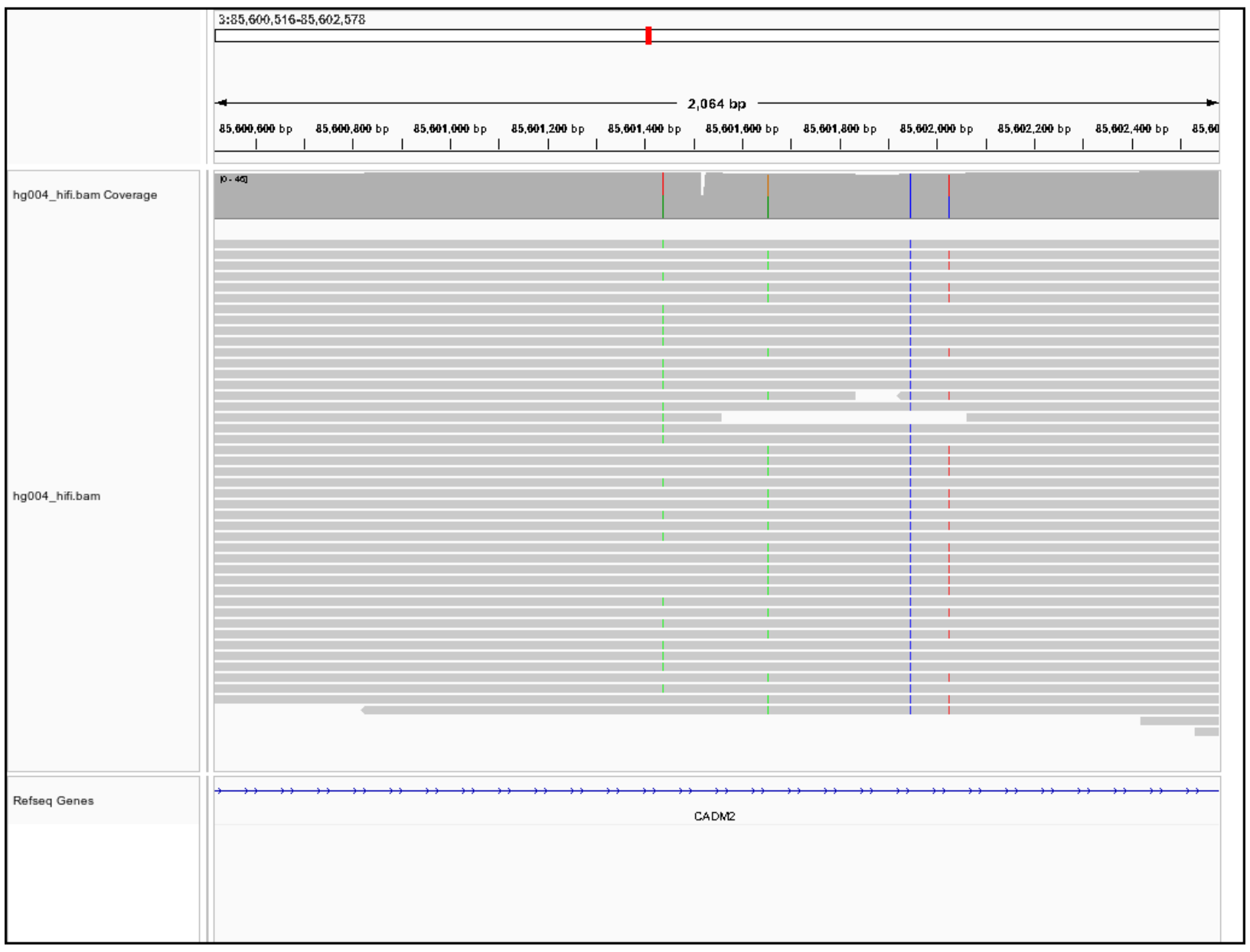

GIAB: Chr3: 127859142 7043bp INS HG002_GT=0/1; HG003_GT=0/0; HG004_GT=0/1

SVPG

Chr3:127859142

7043bp

INS

0/1

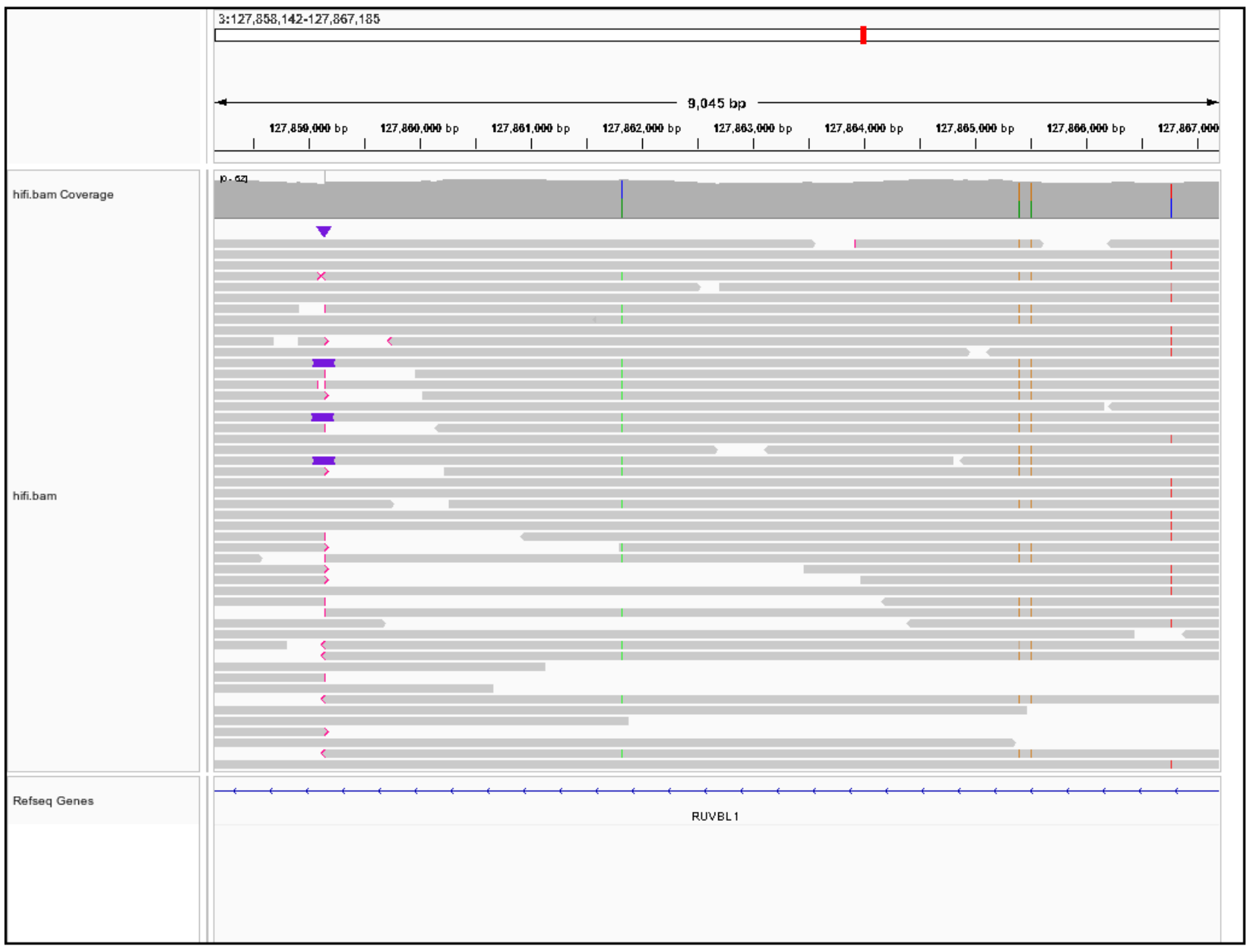

NA

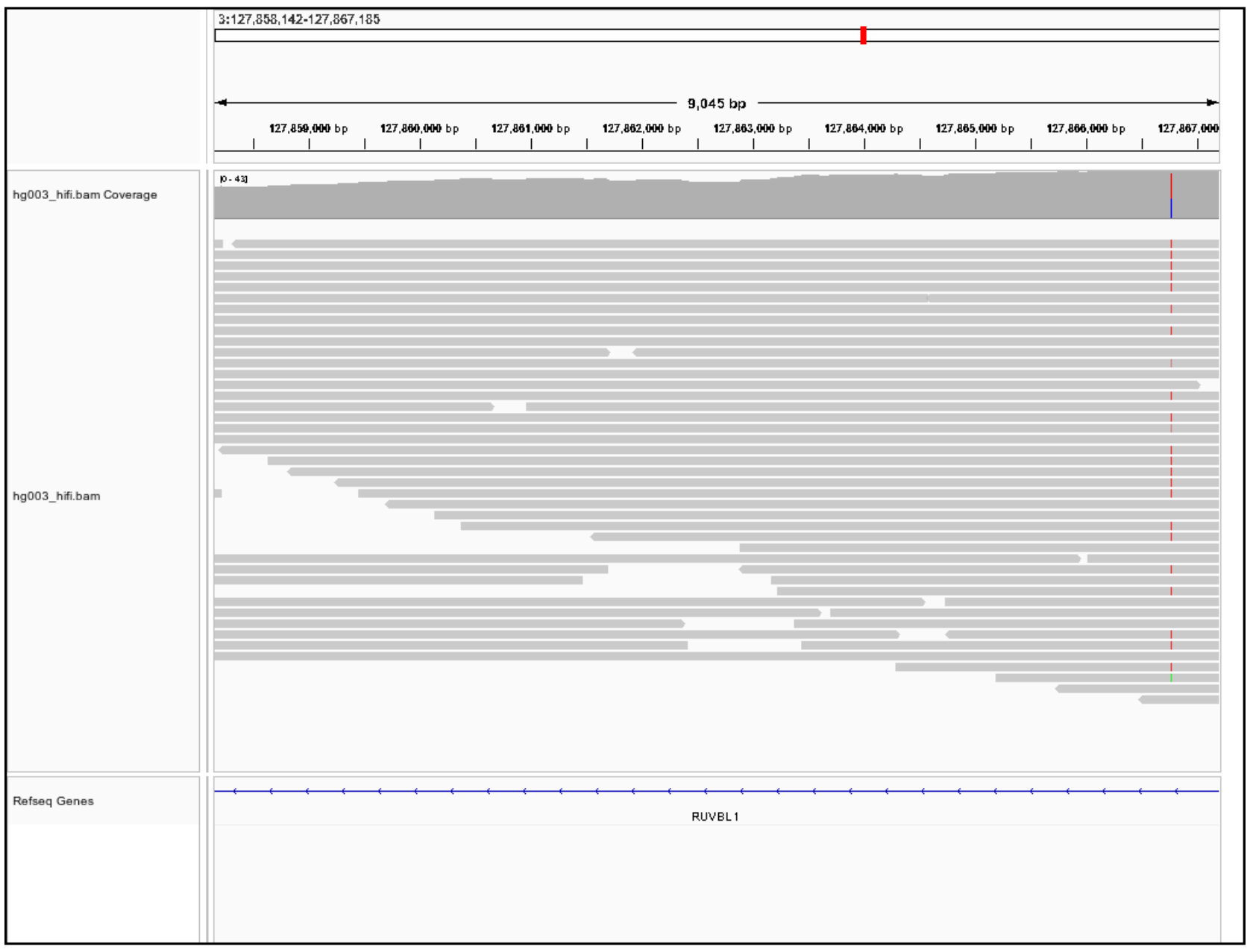

NA

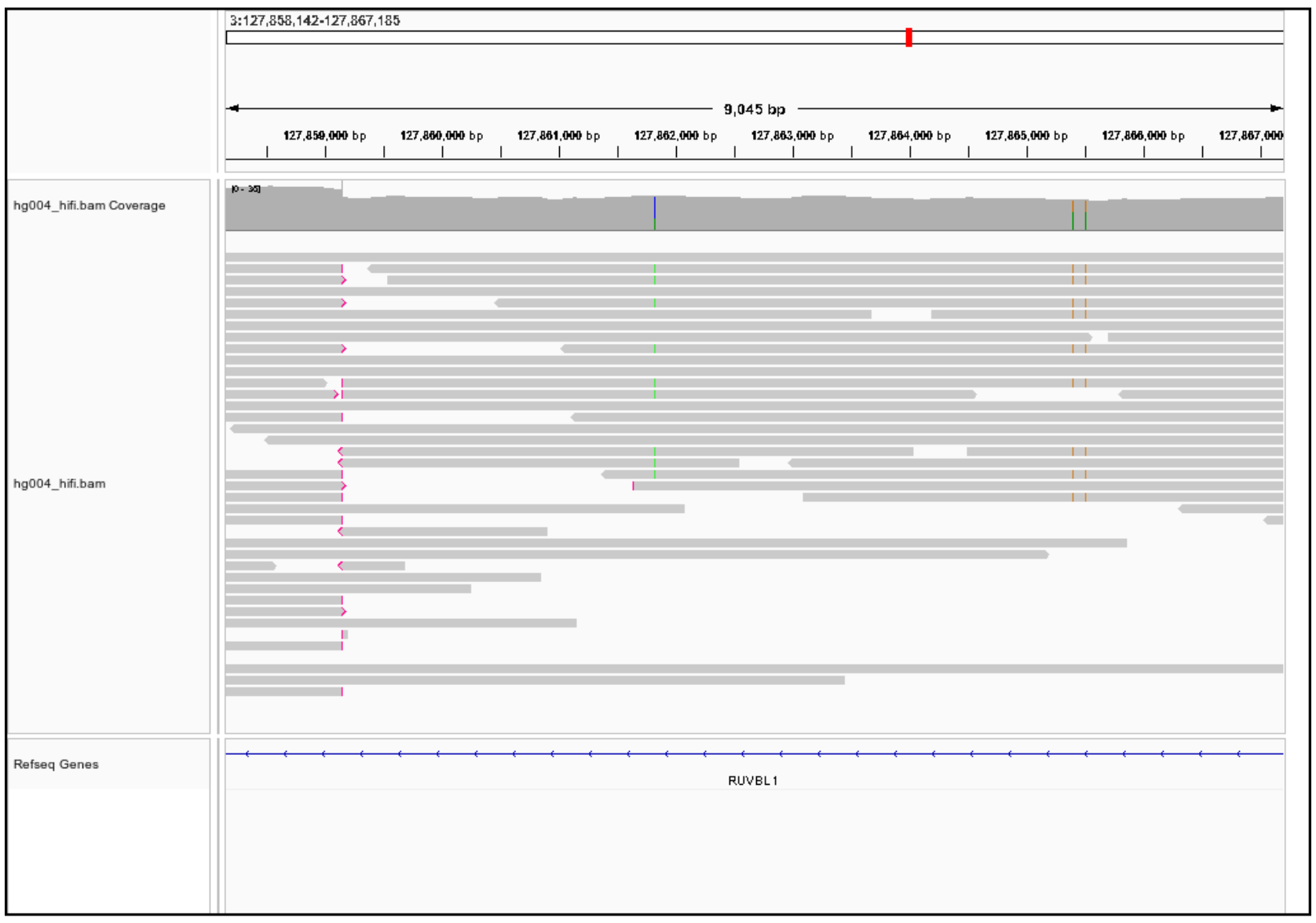

GIAB: NA

SVPG

Chr3:130355765

50bp

INS

0/1

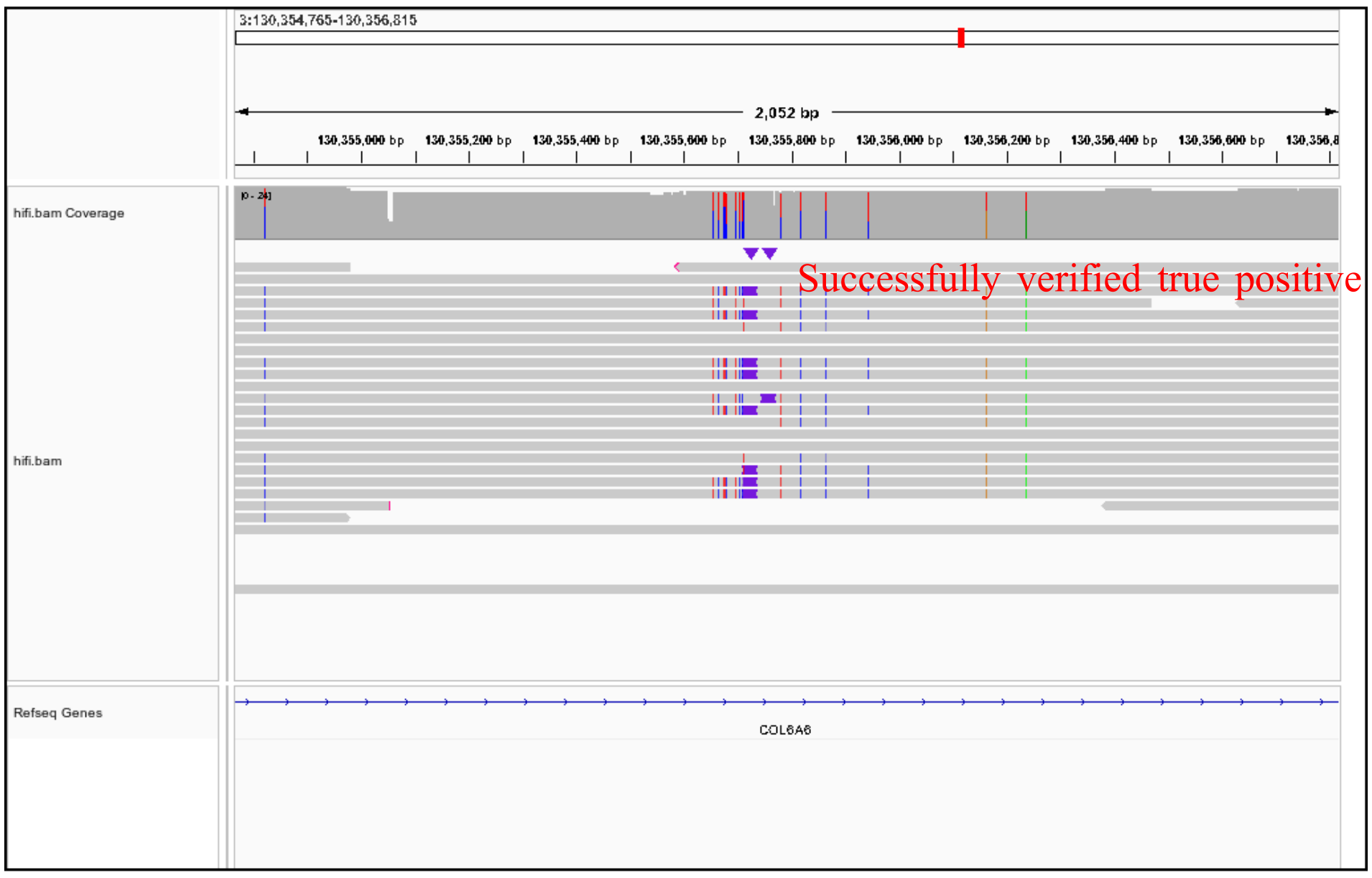

Successfully verified true positive *de novo* SV

NA

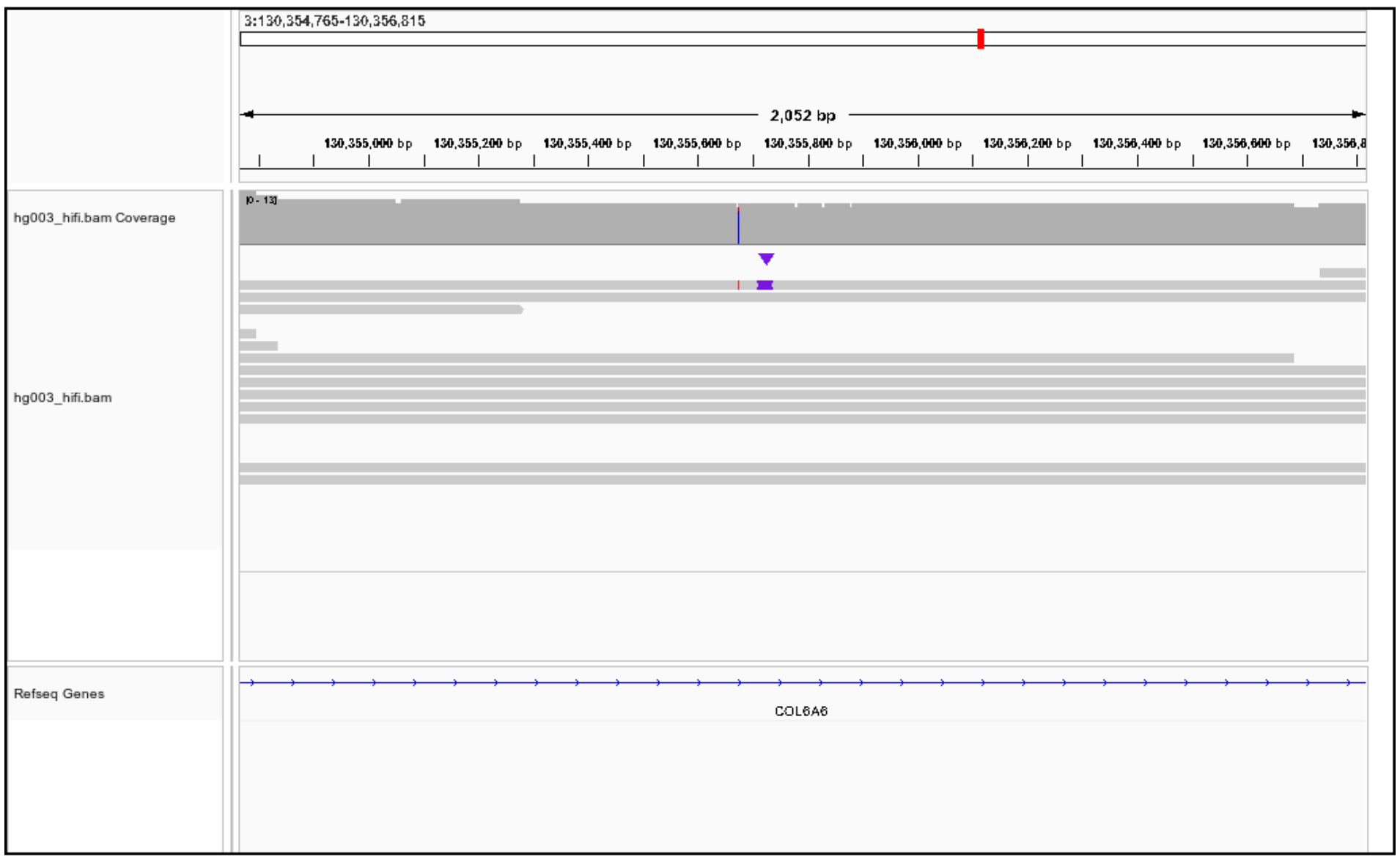

NA

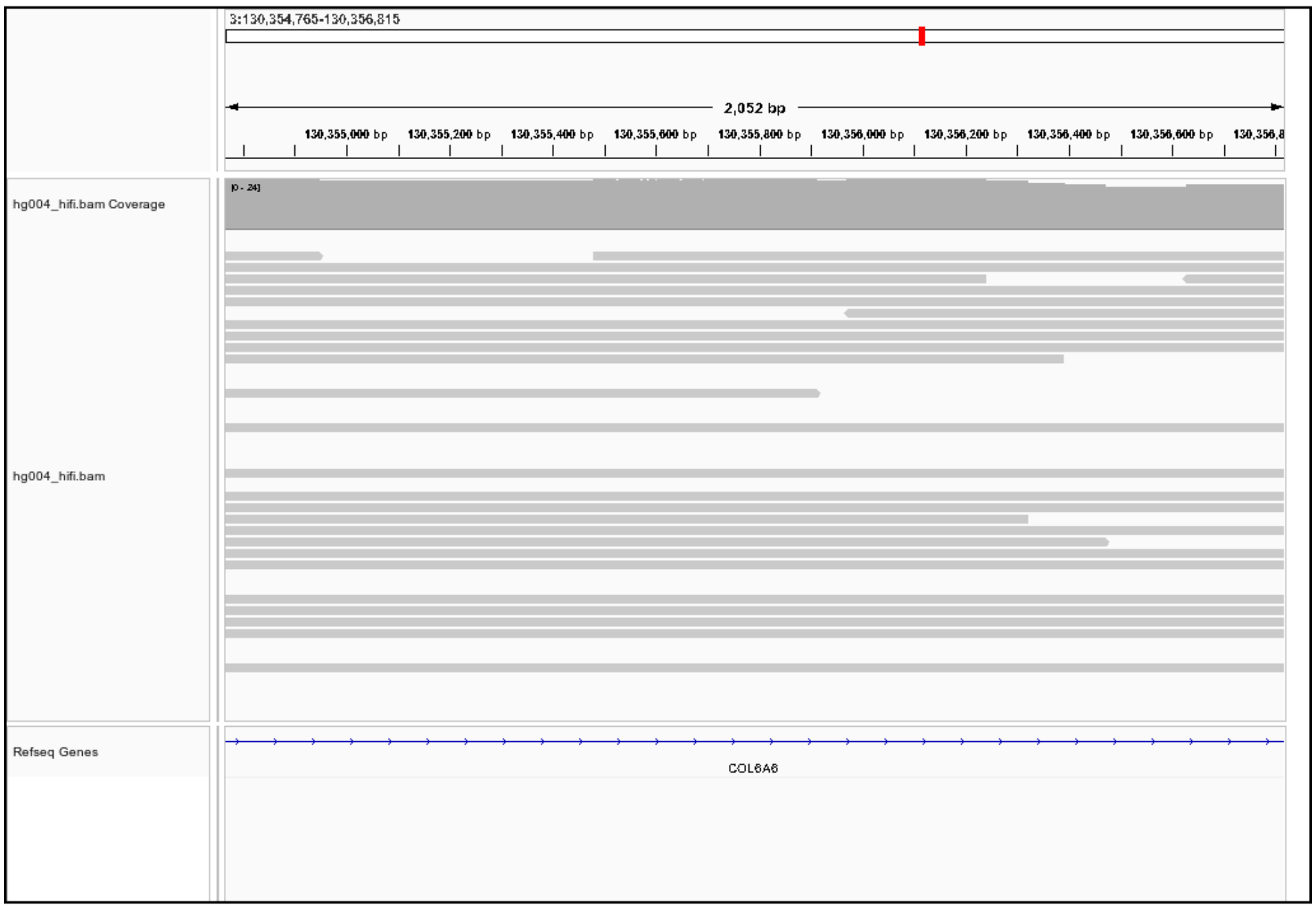

GIAB: Chr1: 167404234 99bp INS HG002_GT=0/1; HG003_GT=0/0; HG004_GT=0/1

Sniffles2

Chr1: 167404307

73bp

INS

0/1

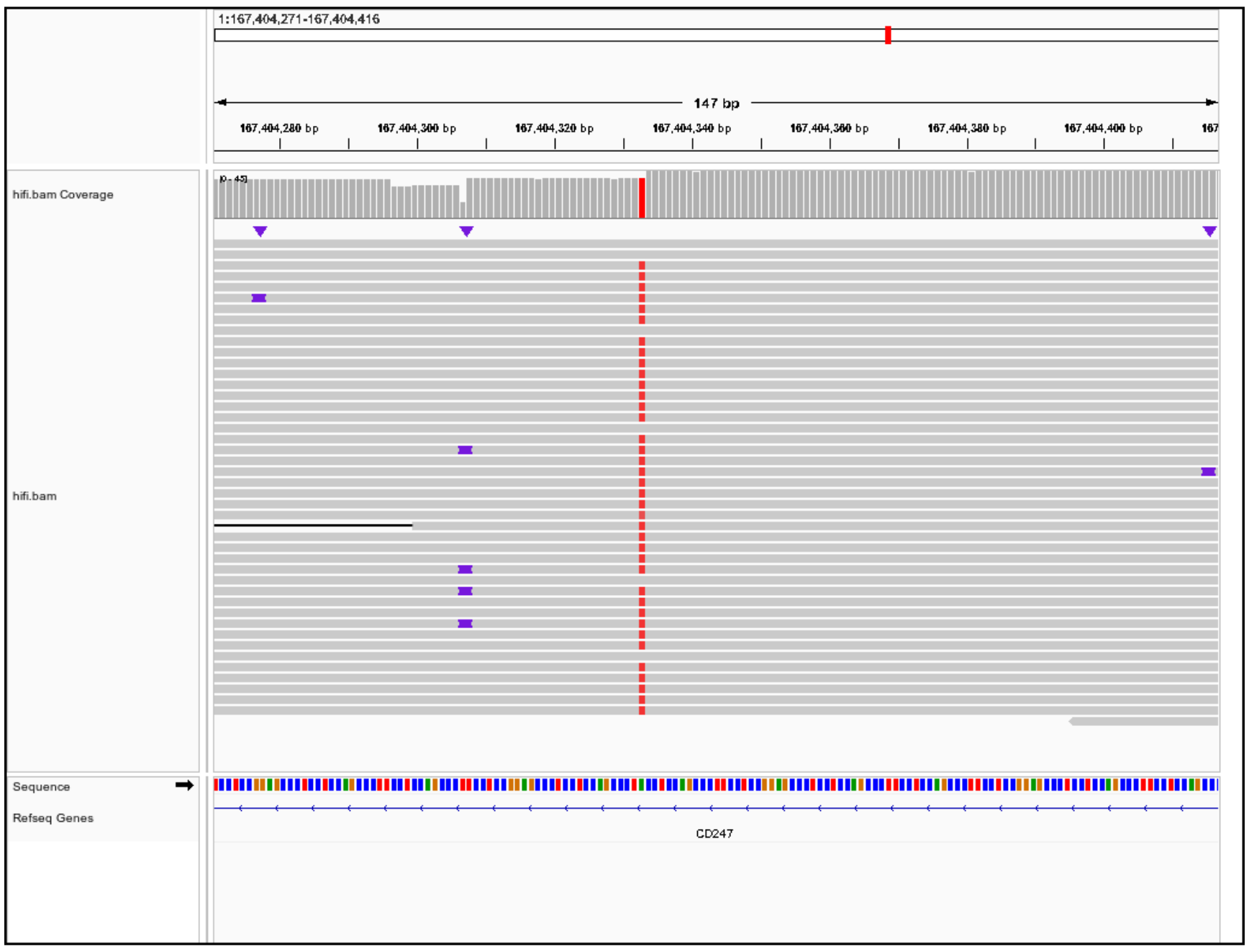

Chr1: 167404320

197bp

INS

1/1

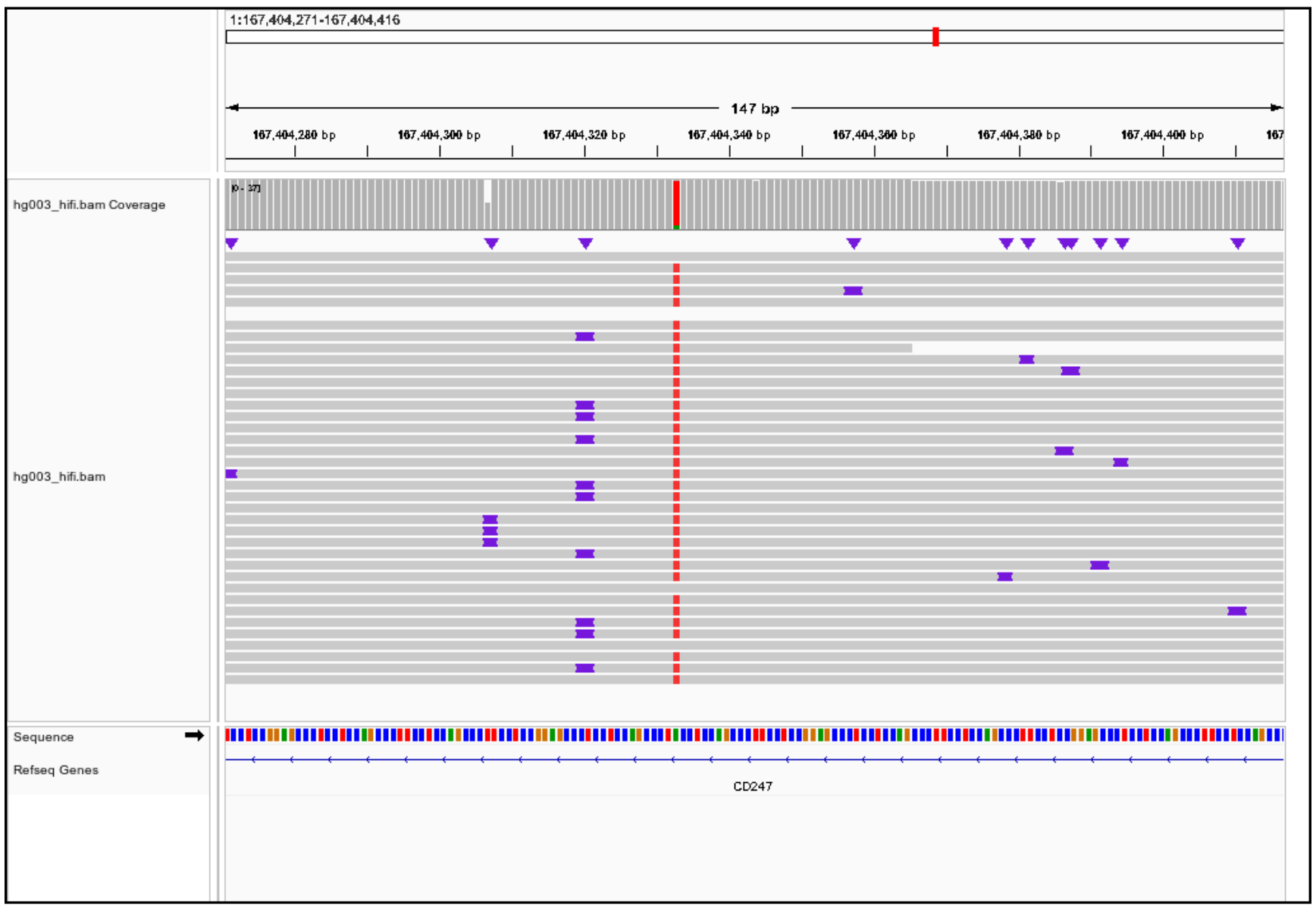

Chr1: 167404177

62bp

DEL

0/1

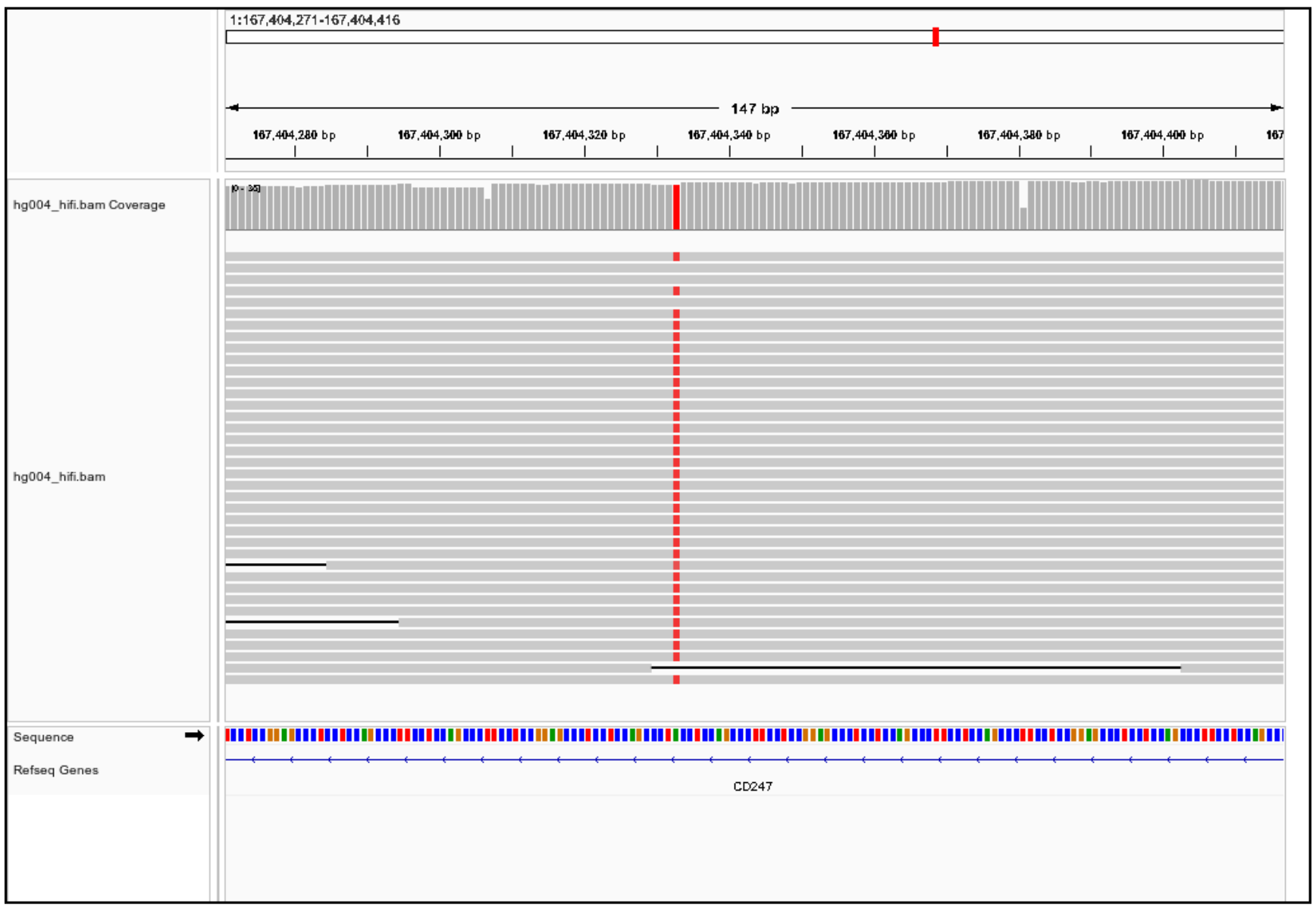

Sniffles2

Chr1: 202174162

64bp

INS

0/1

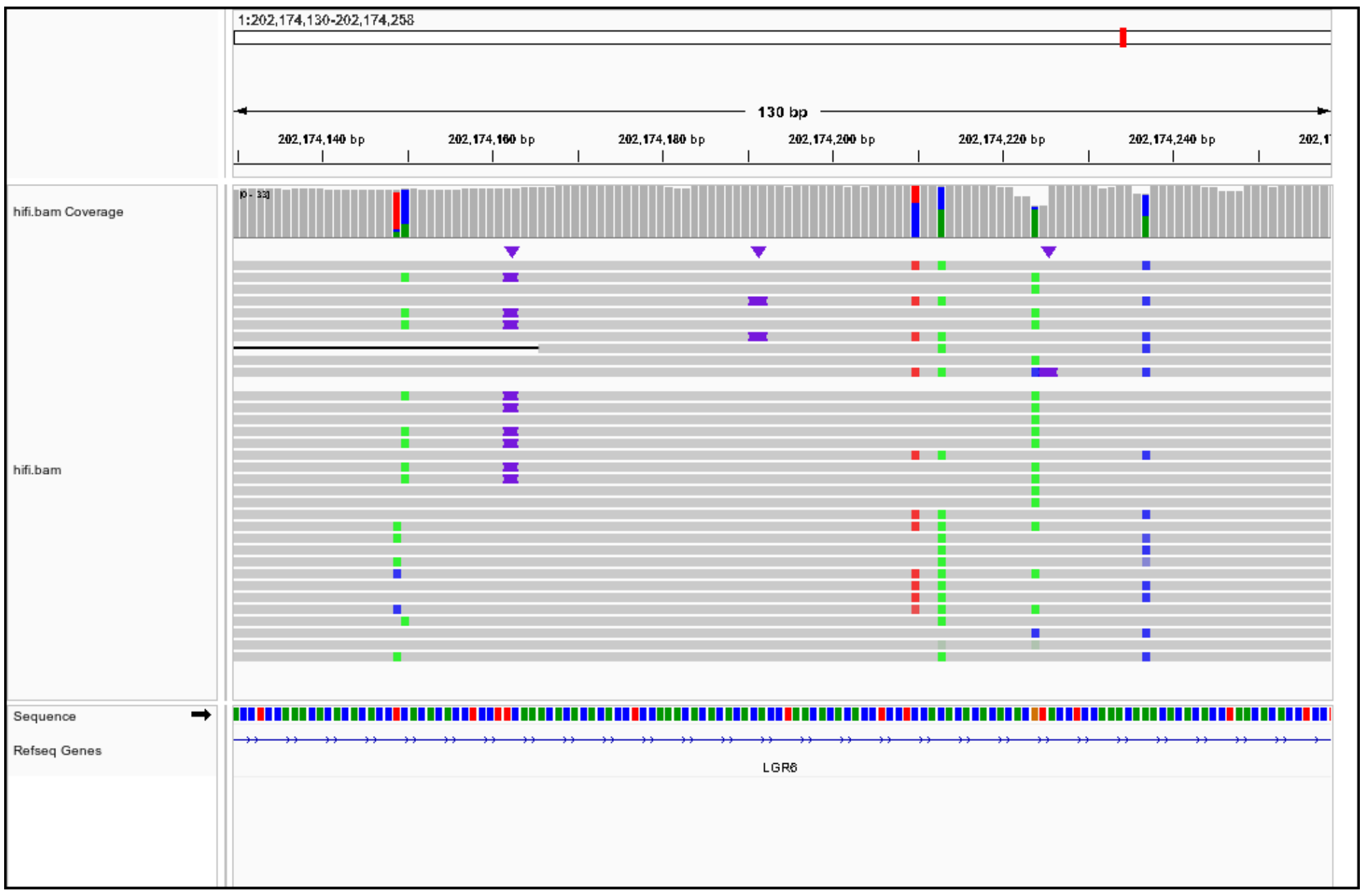

Chr1: 202174339

2425bp

INS

0/1

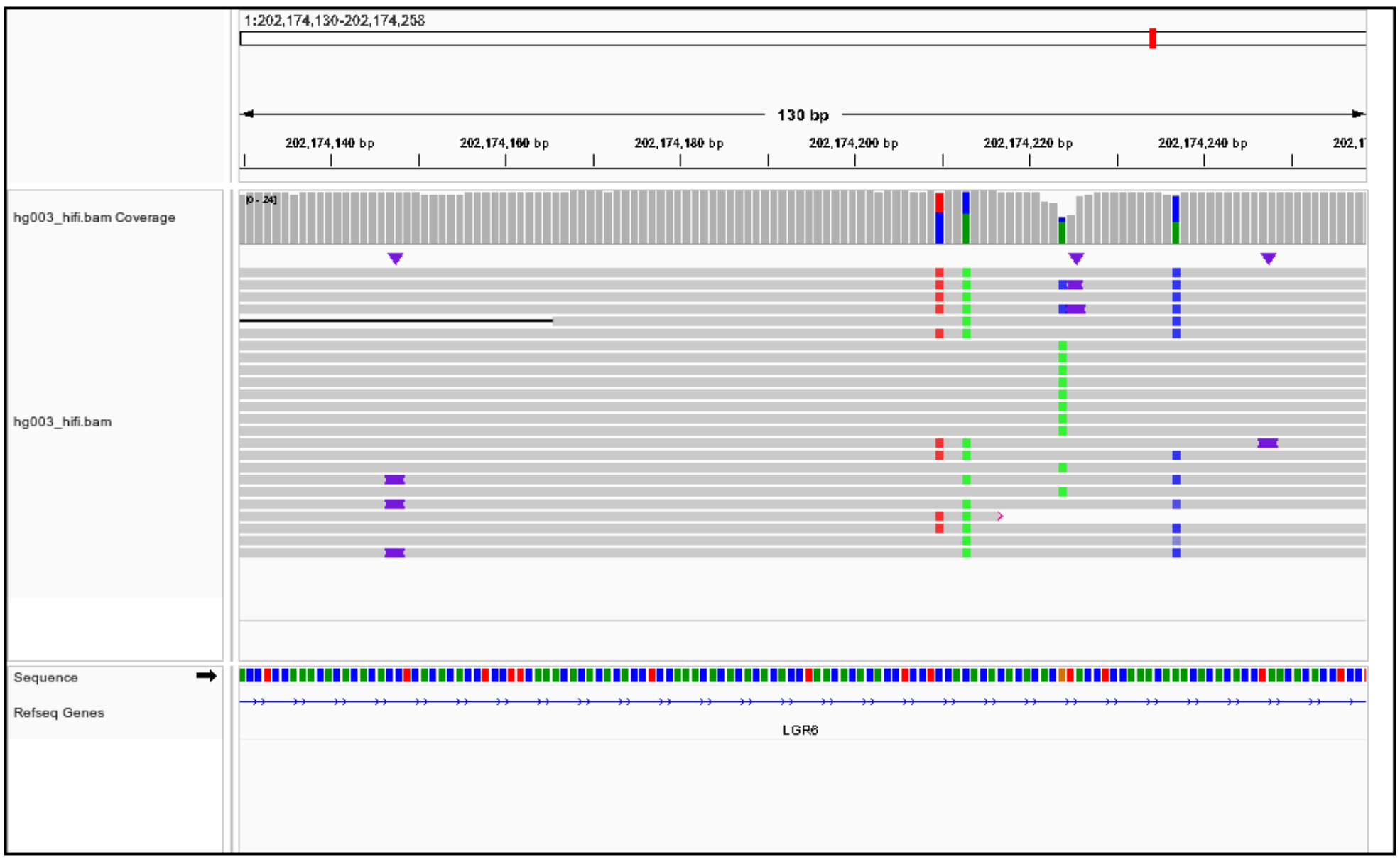

Chr1: 202174538

2111bp

INS

0/1

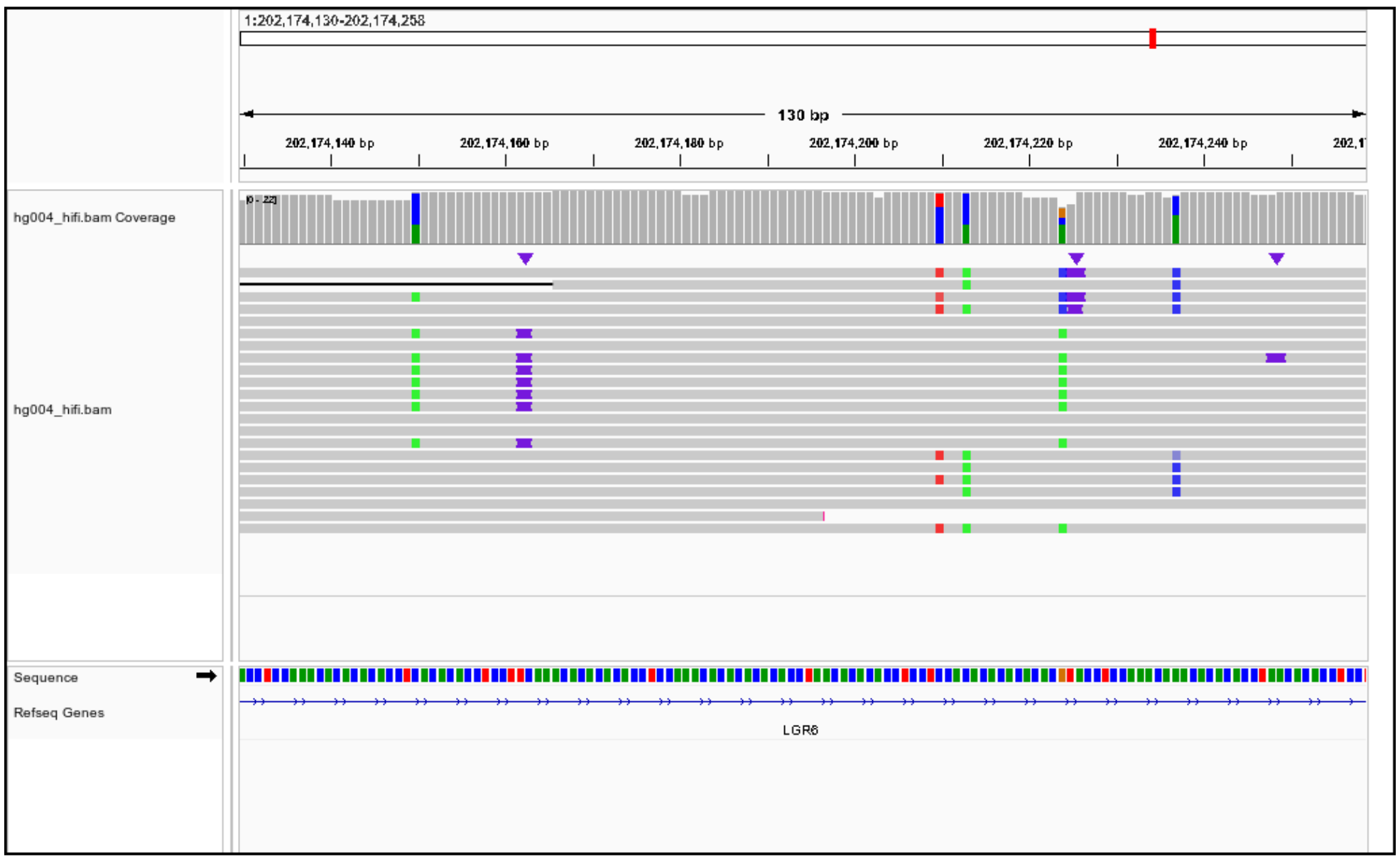

Sniffles2

Chr1: 202174358

304bp

INS

0/1

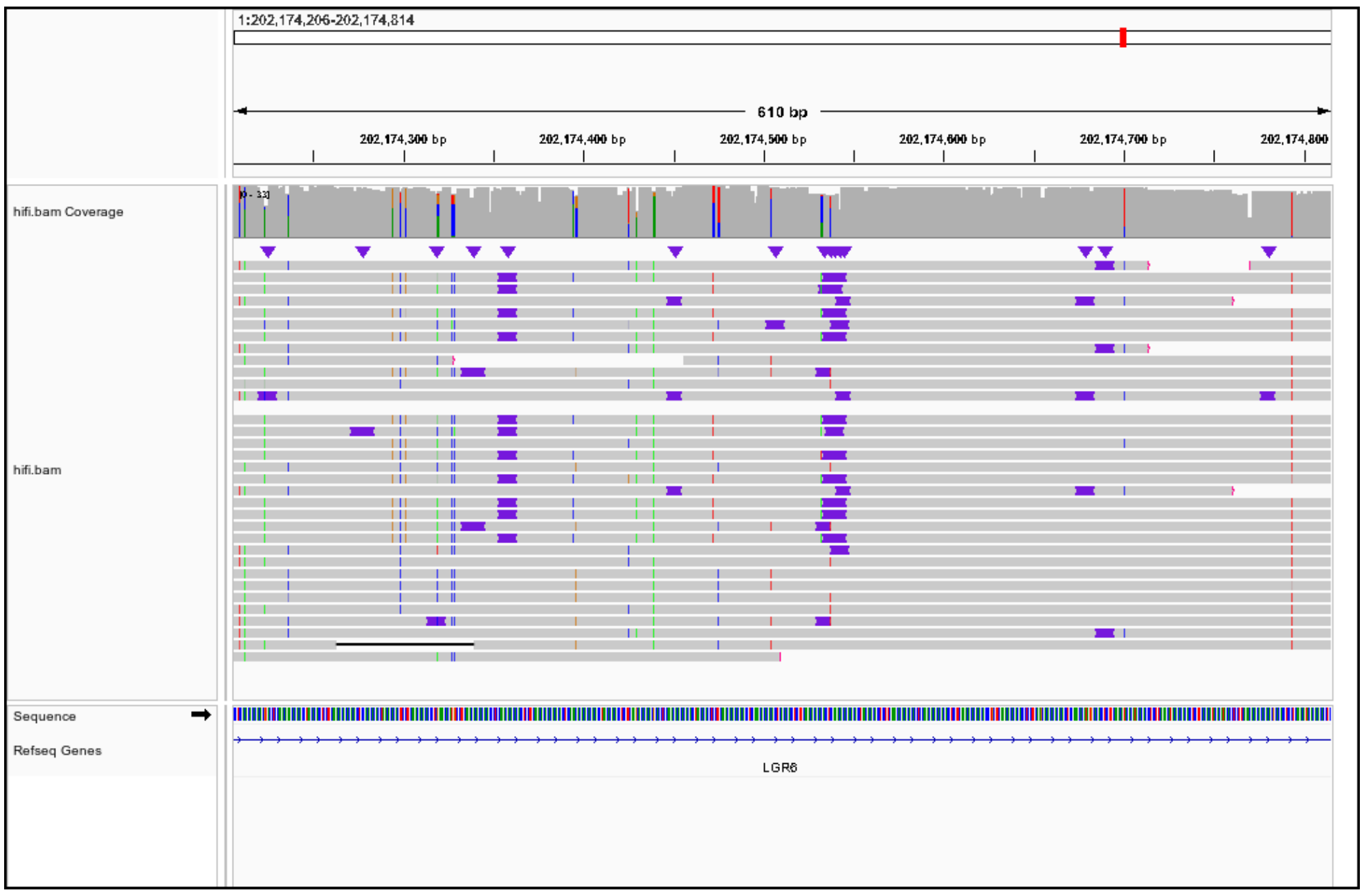

Chr1: 202174339

2425bp

INS

0/1

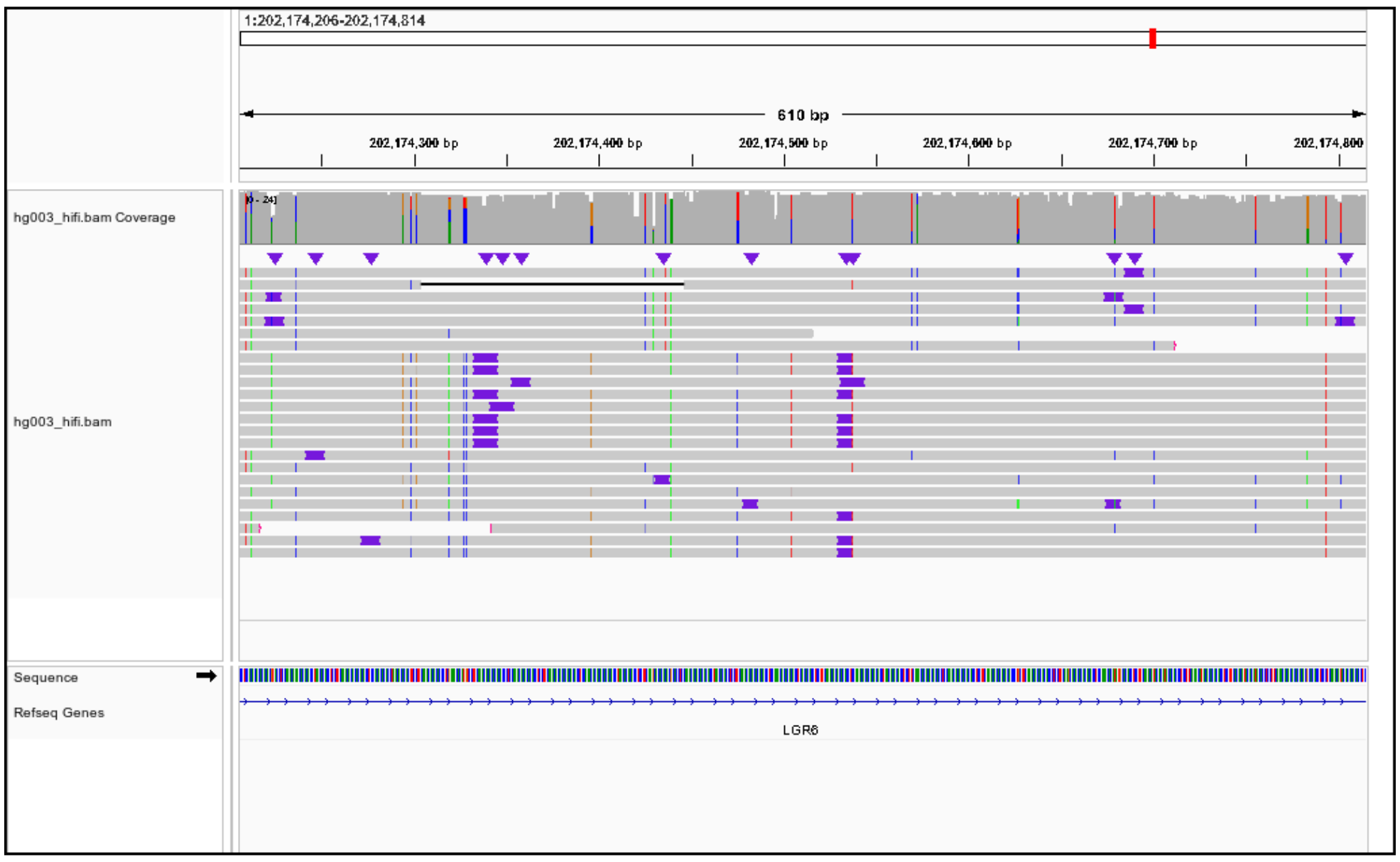

Chr1: 202174538

2111bp

INS

0/1

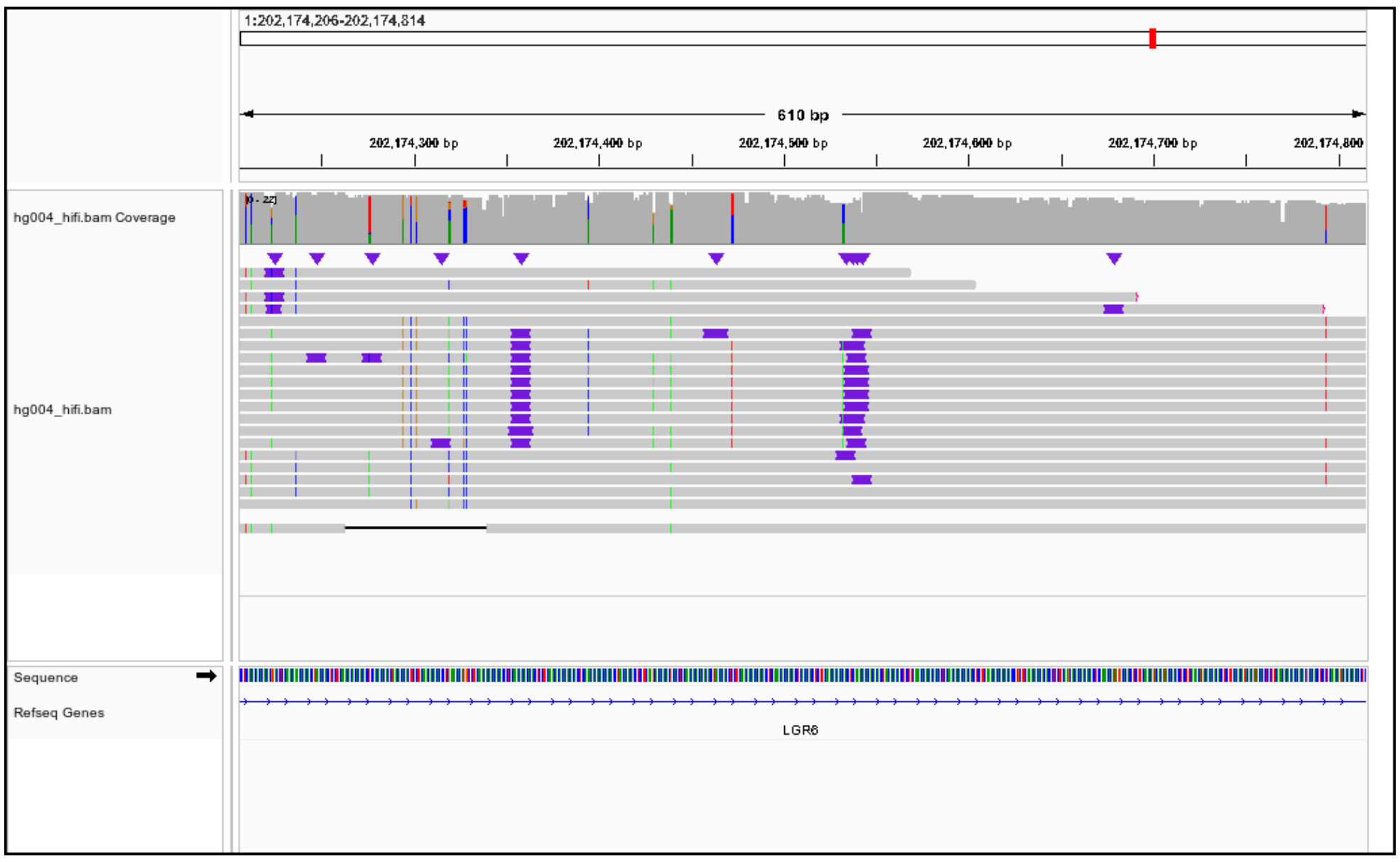

GIAB: Chr2: 845231 333bp INS HG002_GT=0/1; HG003_GT=1/1; HG004_GT=0/0

Sniffles2

Chr2: 845359

143bp

INS

0/1

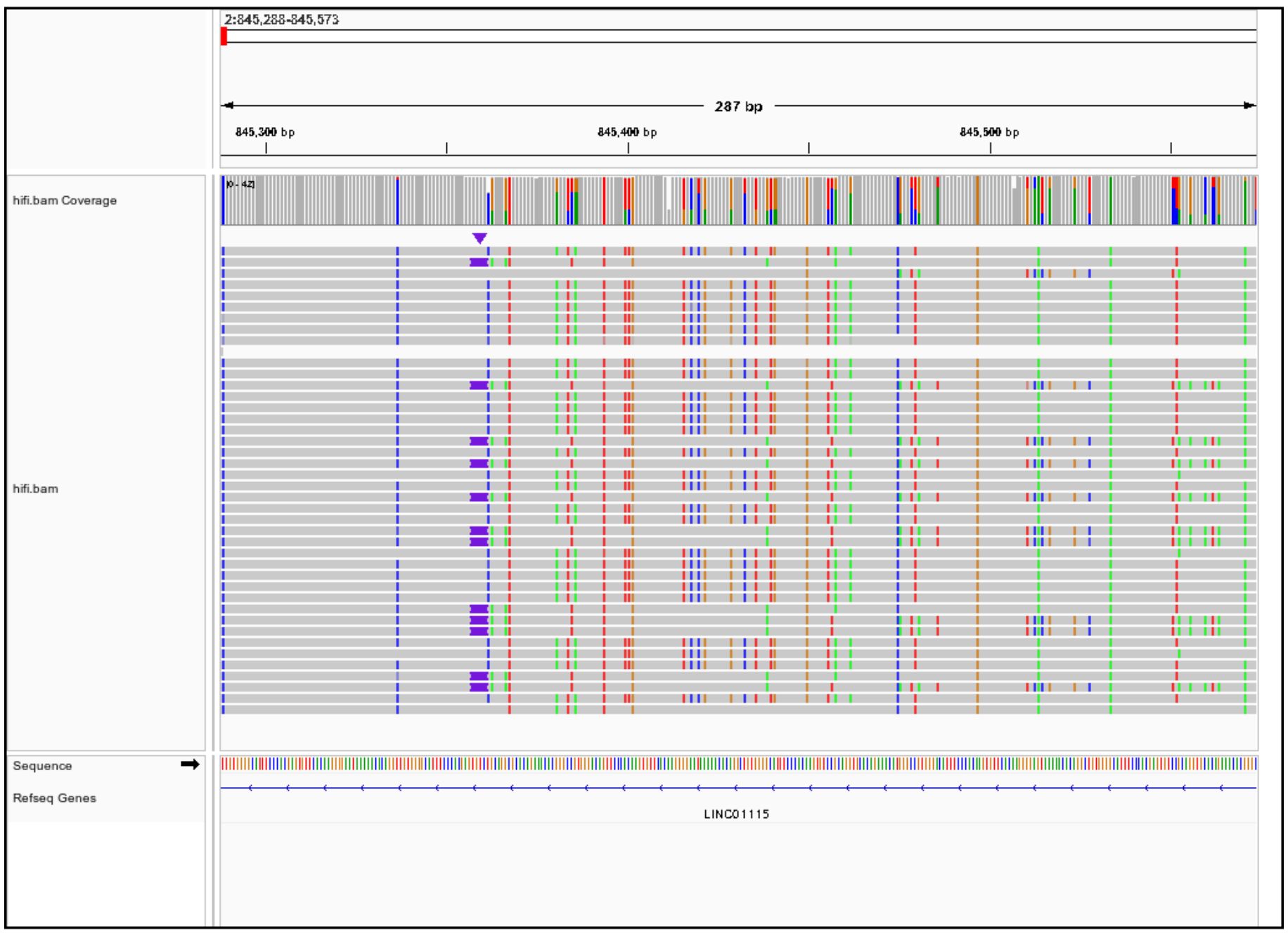

Chr2: 845781

379bp

INS

0/1

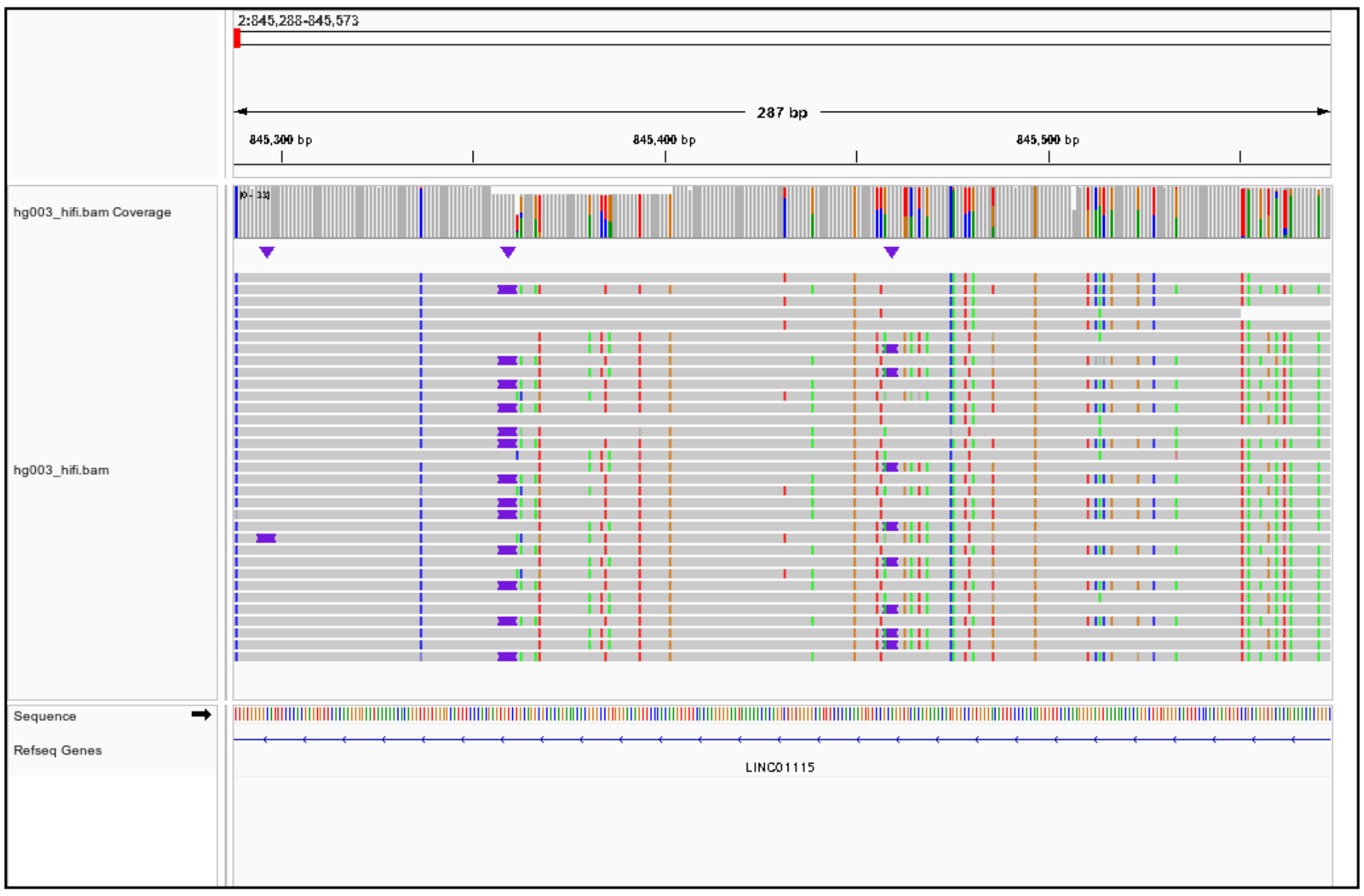

NA

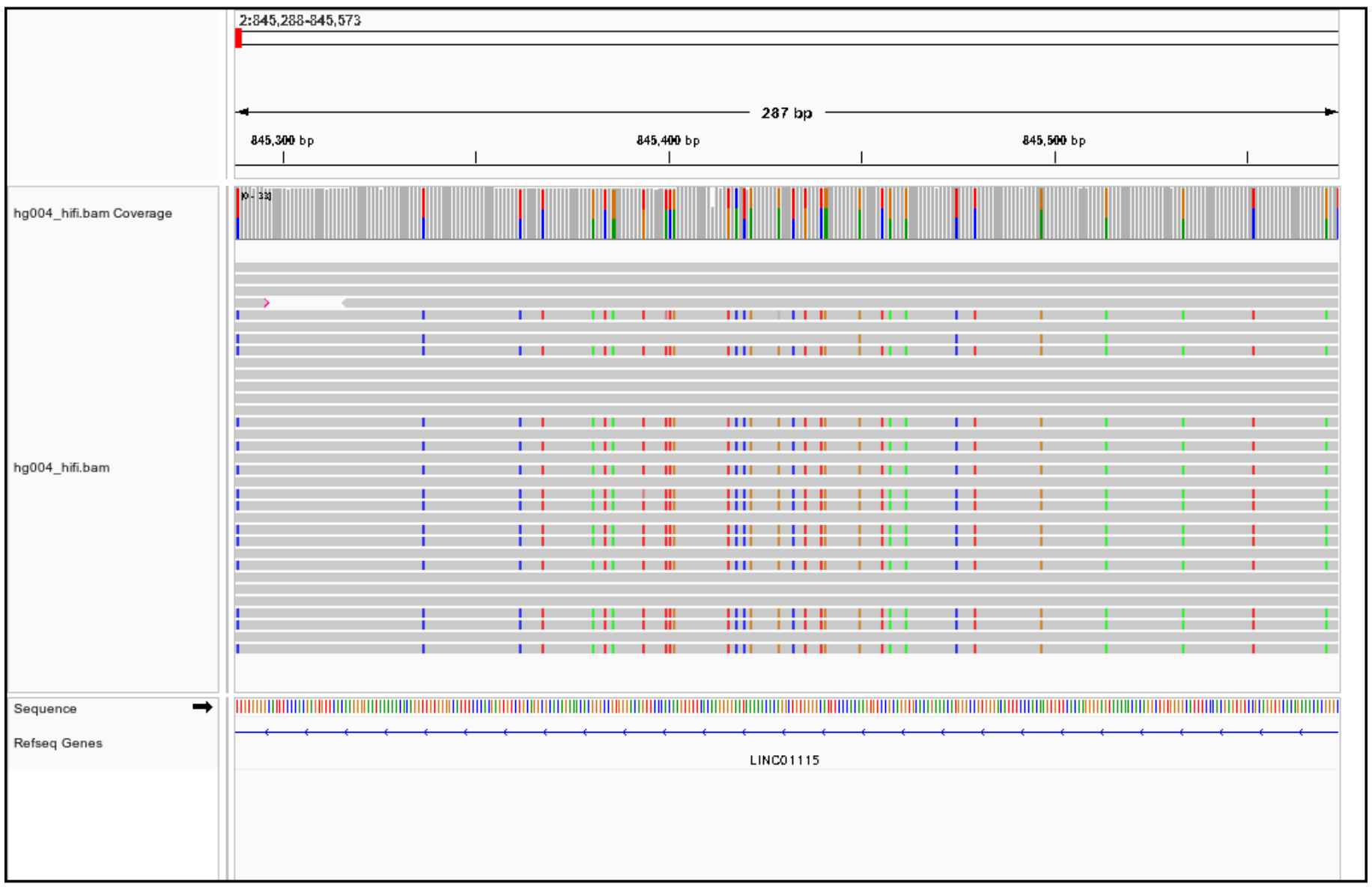

GIAB: Chr2: 845231 333bp INS HG002_GT= 0/1; HG003_GT=1/1; HG004_GT=0/0

Sniffles2

Chr2: 845955

141bp

INS

0/1

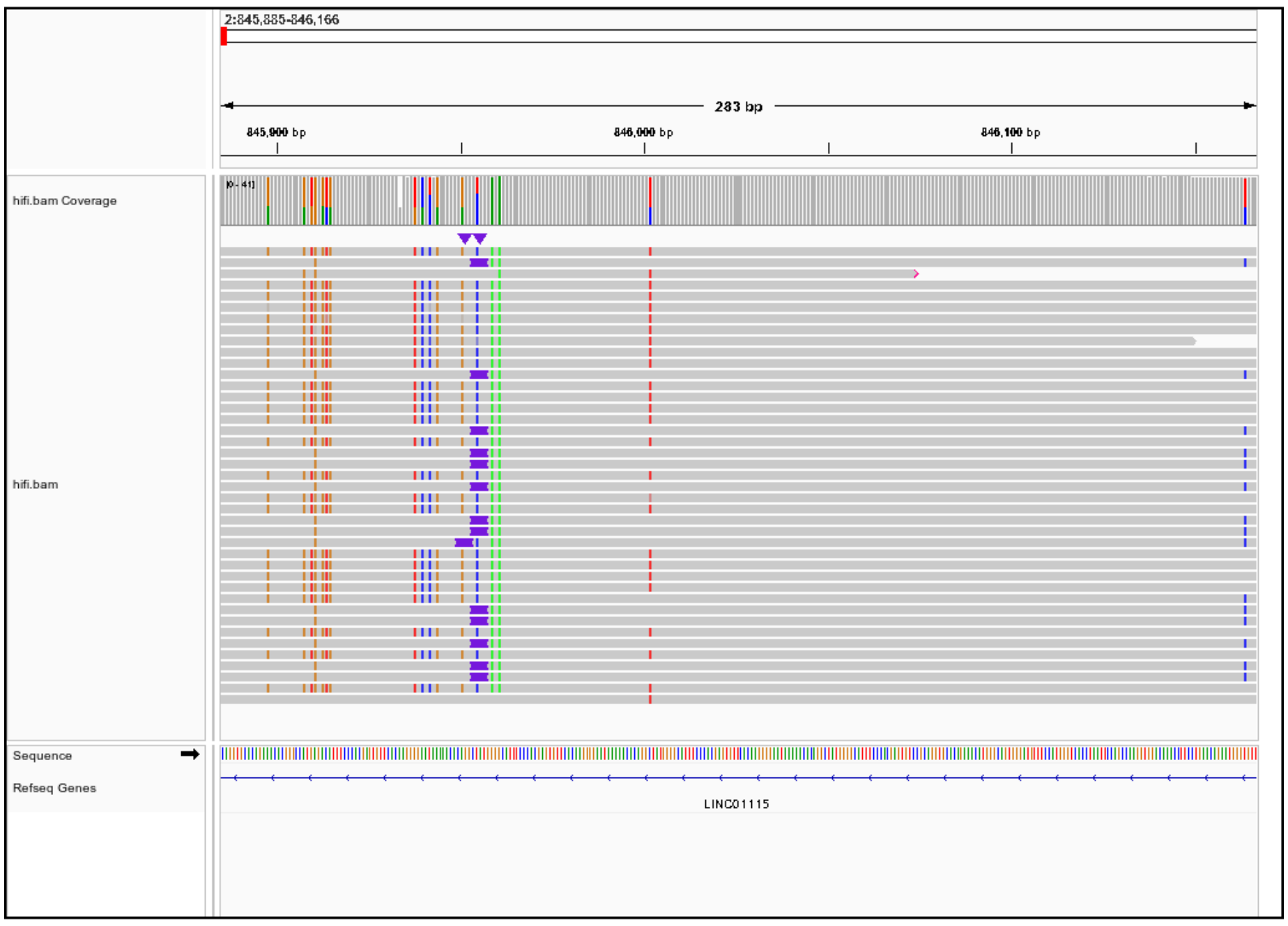

Chr2: 845964

567bp

INS

0/0

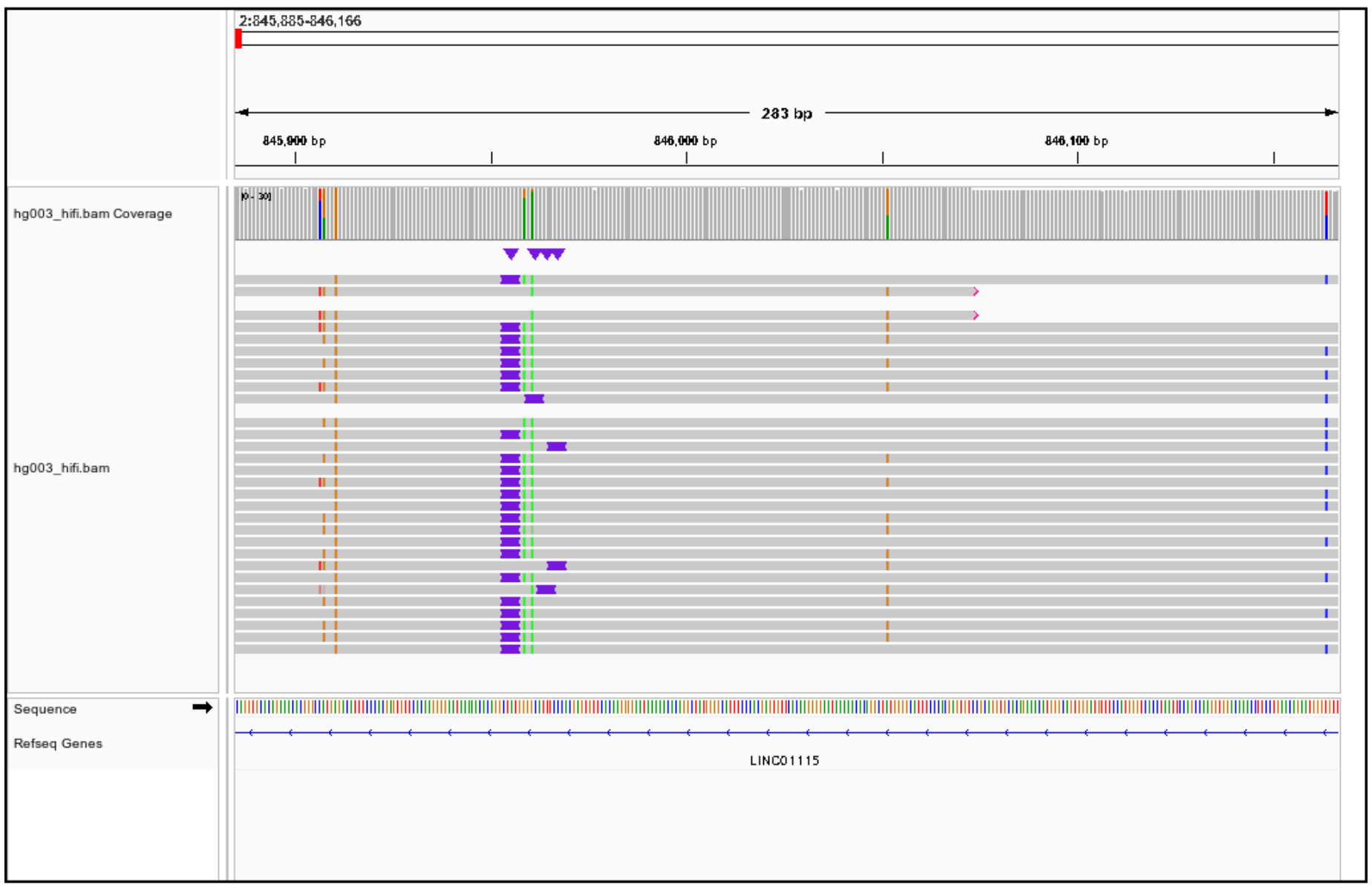

NA

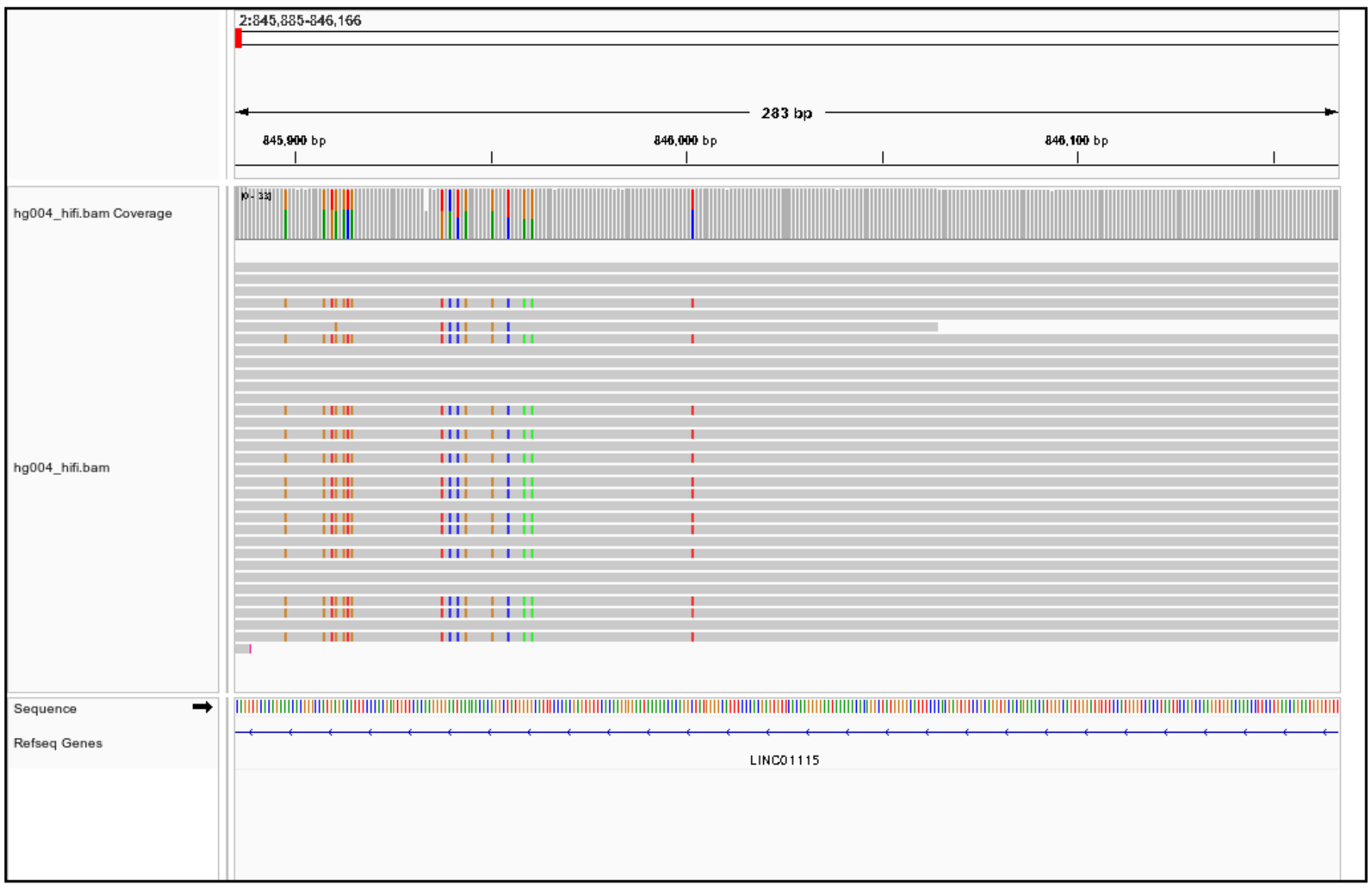

GIAB: Chr2: 34695830 40730bp DEL HG002_GT= 0/1; HG003_GT=1/1; HG004_GT=0/0

Sniffles2

Chr2: 34695829

40738bp

DEL

1/1

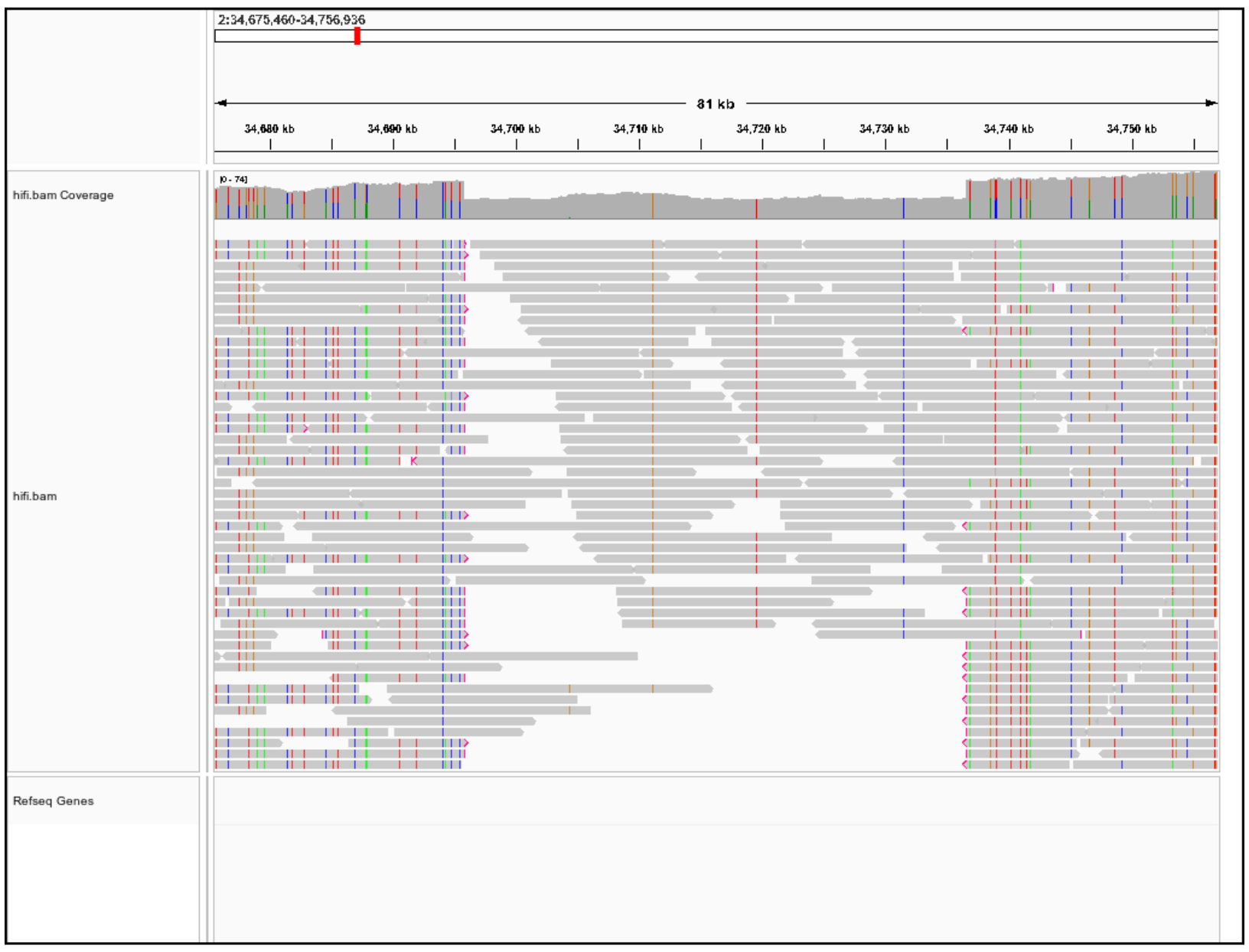

NA

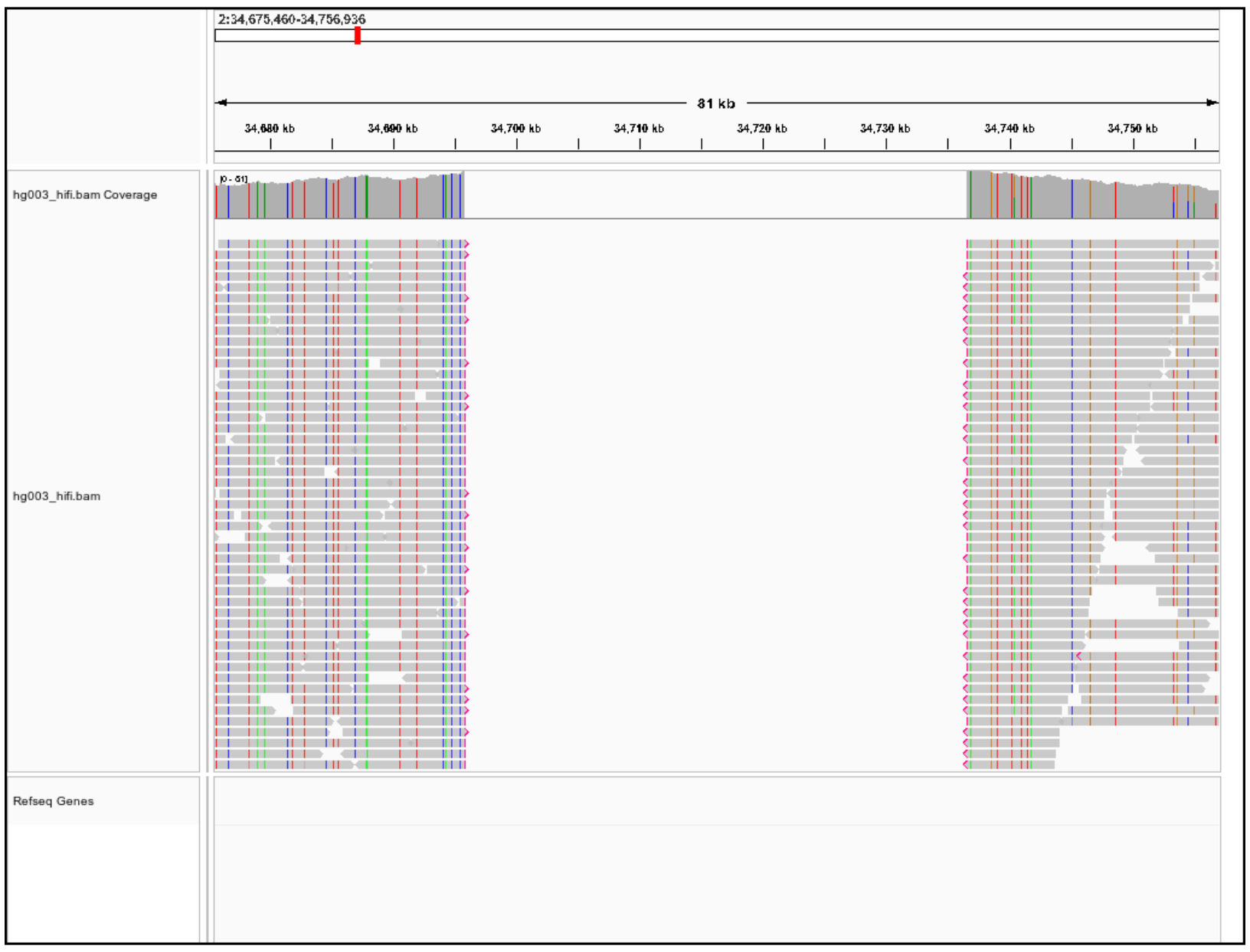

NA

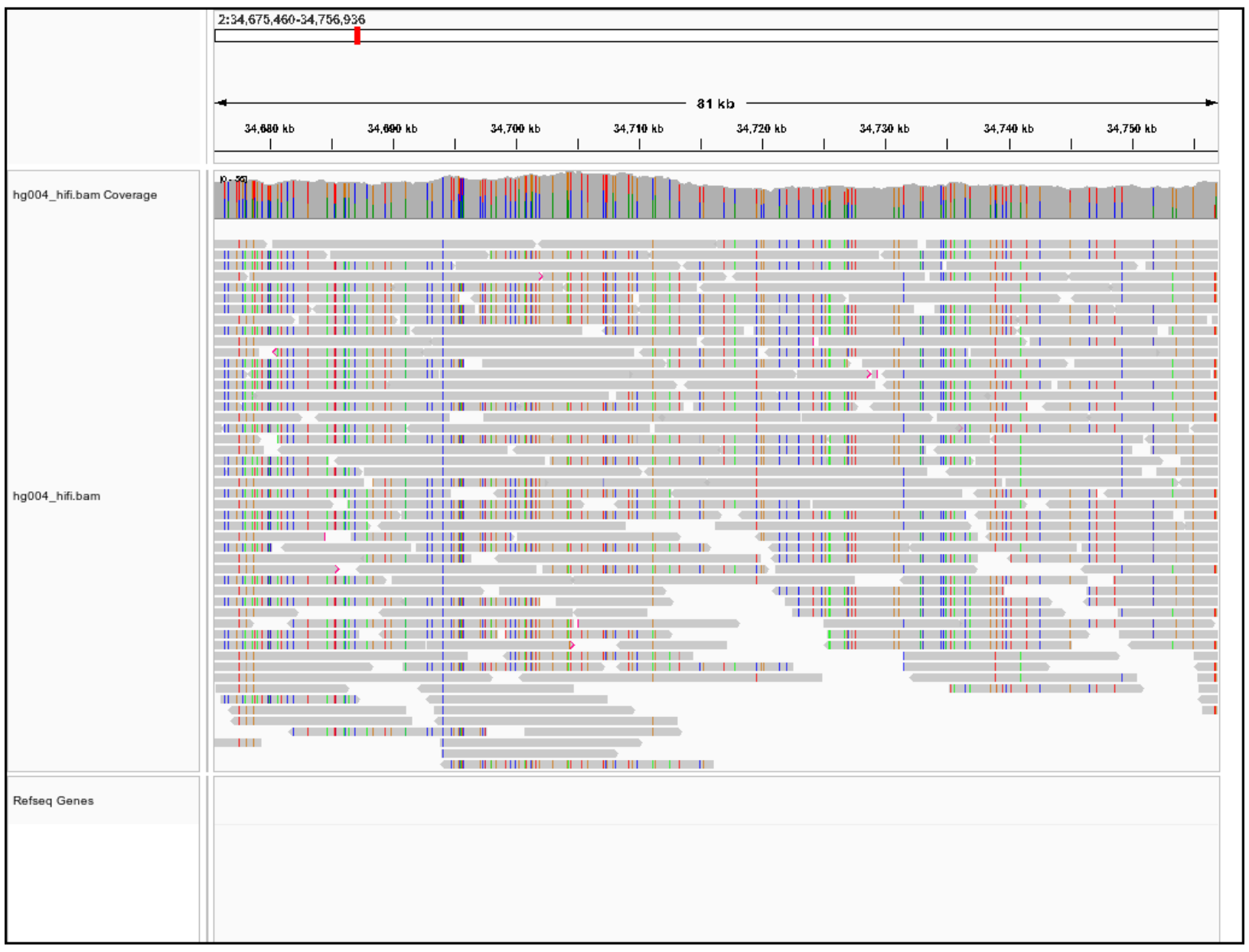

GIAB: Chr2: 232734665 1895bp DEL HG002_GT= 0/1; HG003_GT=1/1; HG004_GT=1/1

Sniffles2

Chr2: 232734675

3175bp

INS

1/1

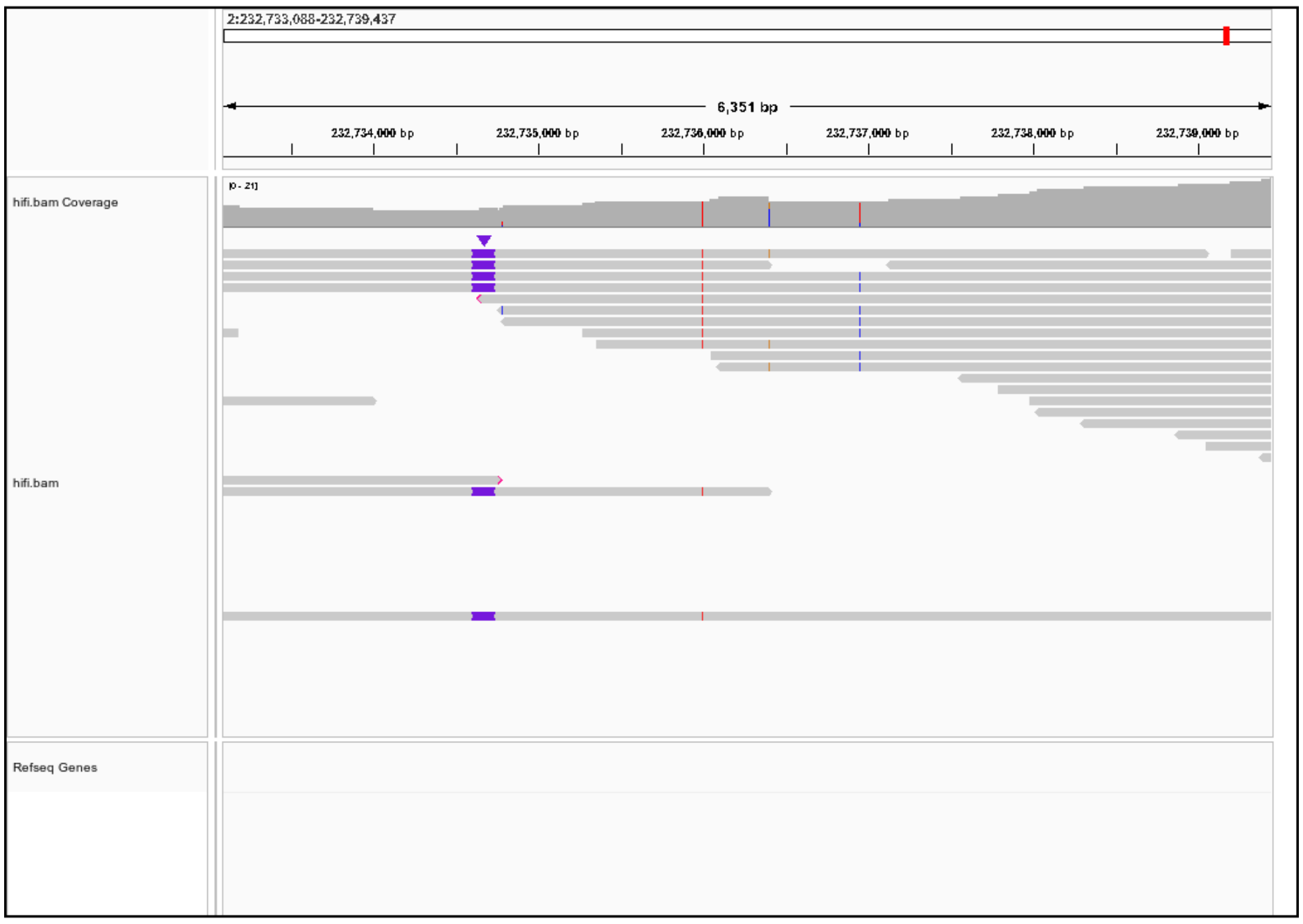

NA

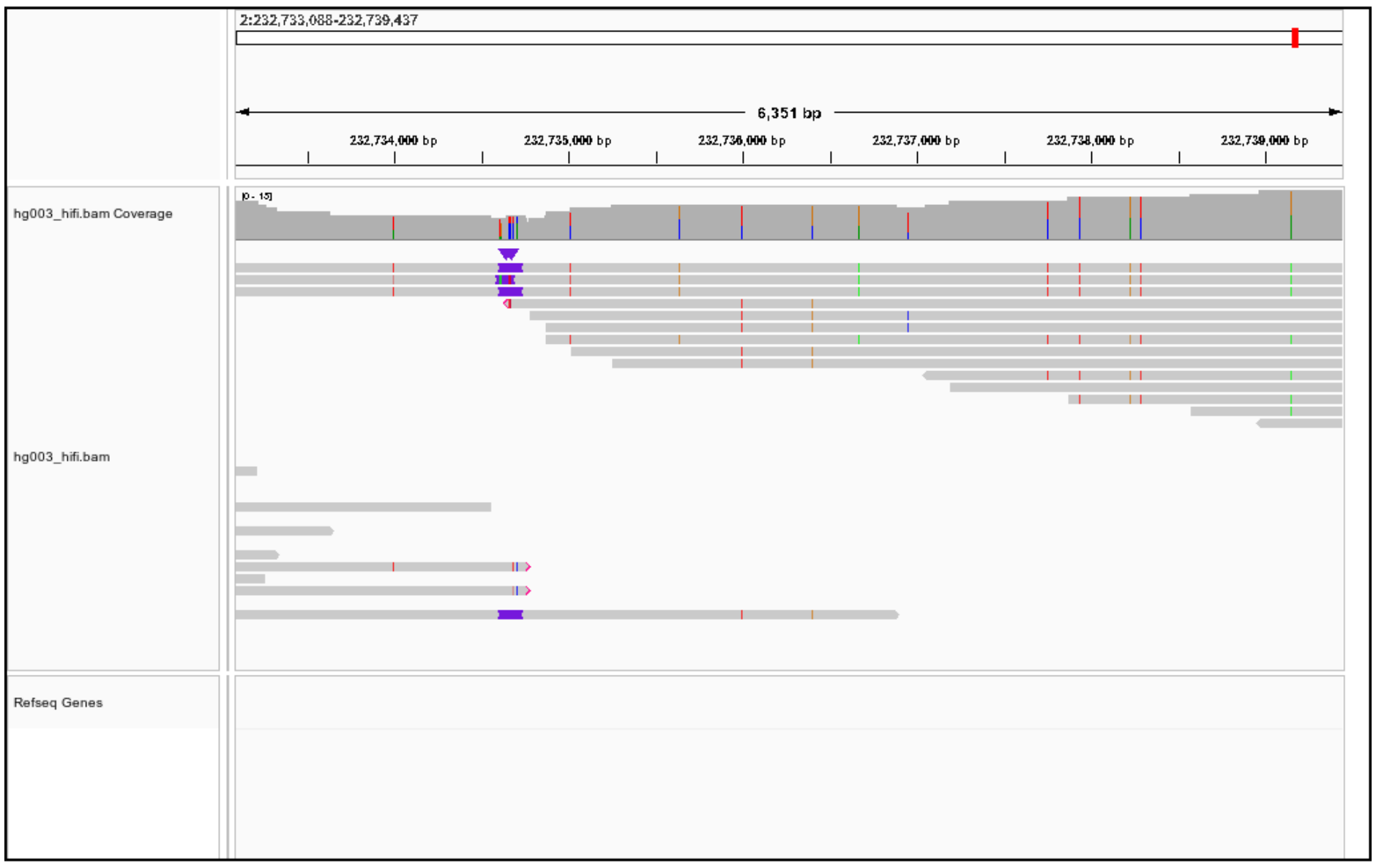

NA

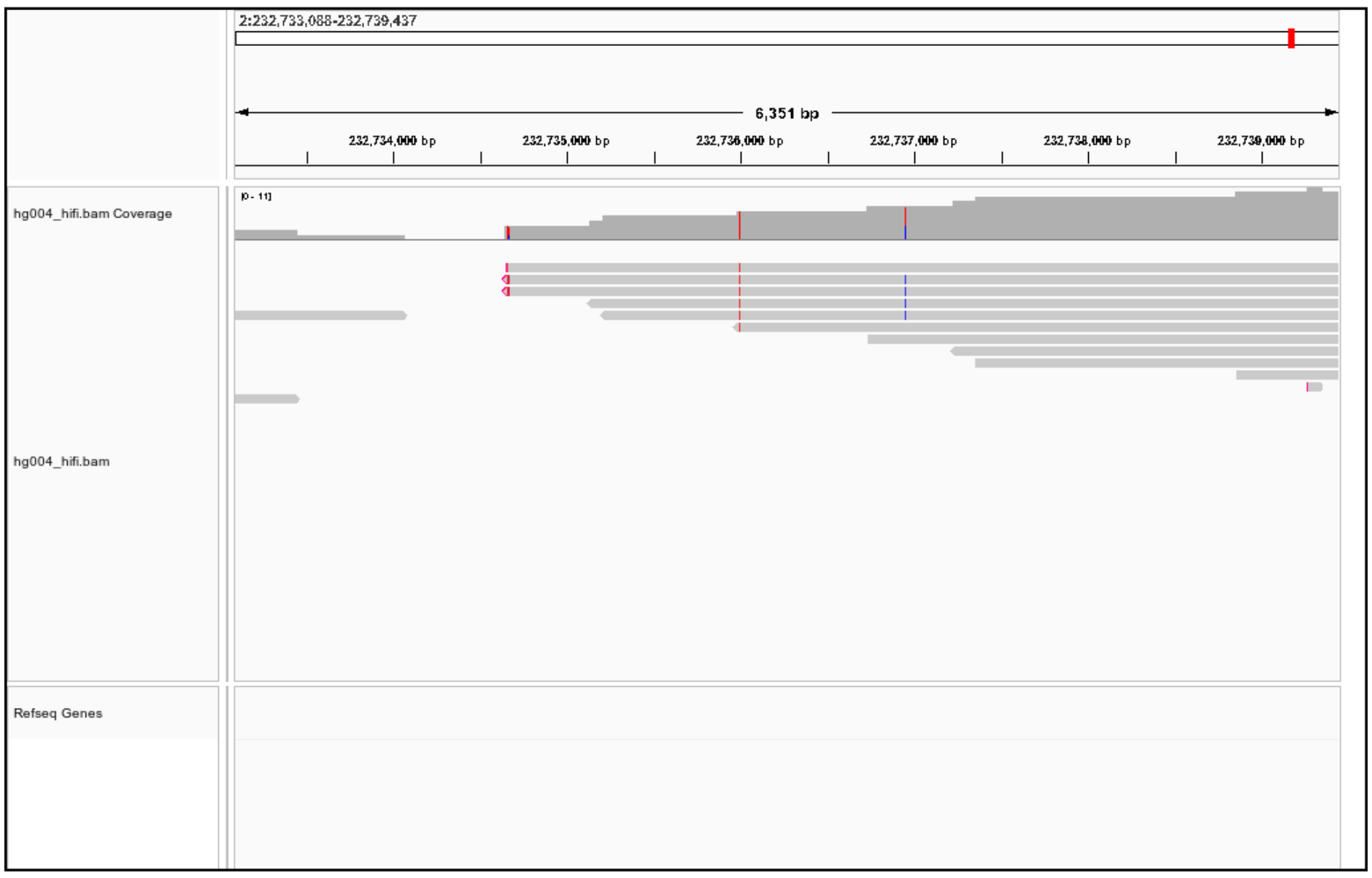

GIAB: Chr2: 232734665 1895bp DEL HG002_GT= 0/1; HG003_GT=1/1; HG004_GT=1/1

Sniffles2

Chr2: 232734678

2017bp

INS

0/1

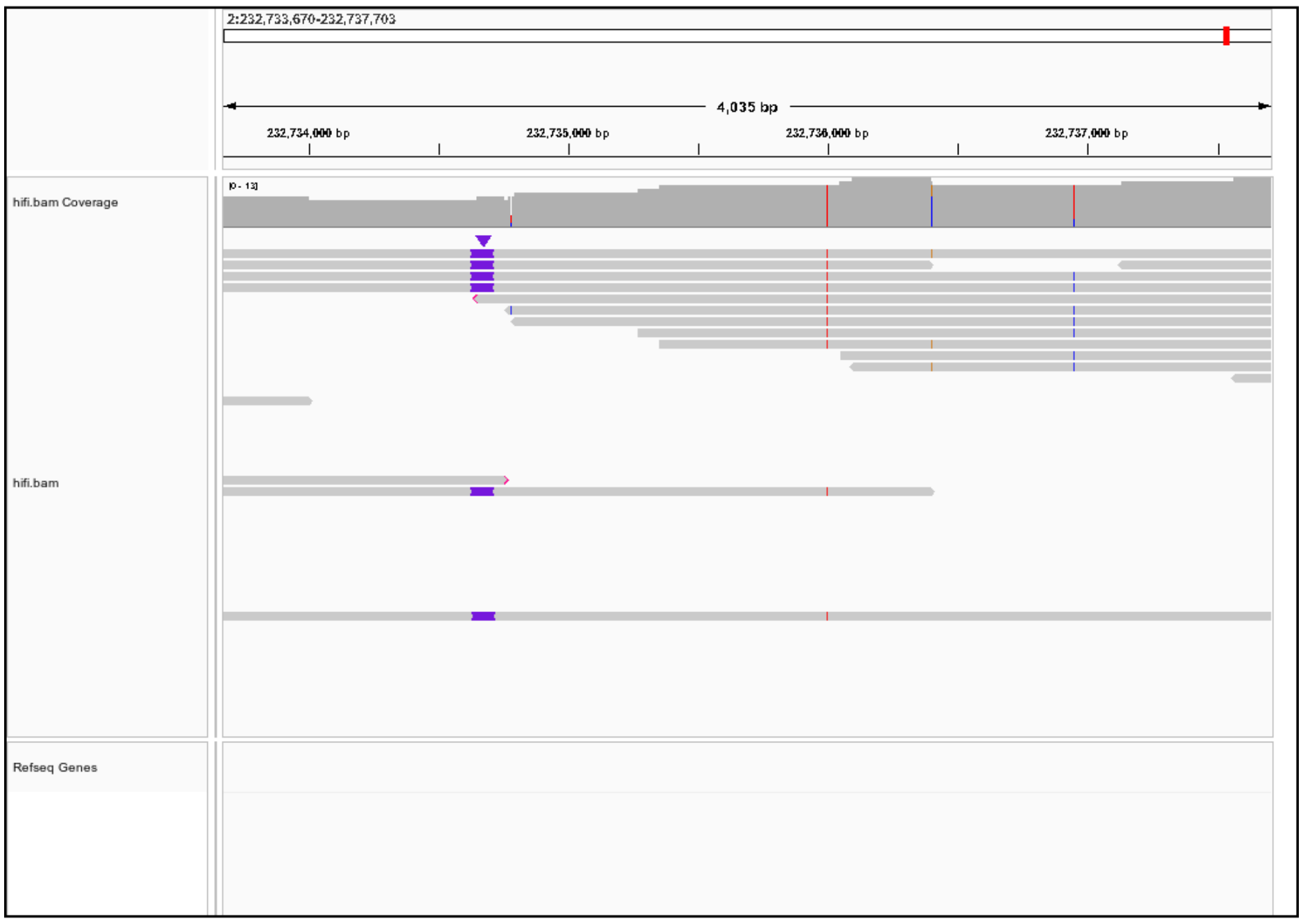

NA

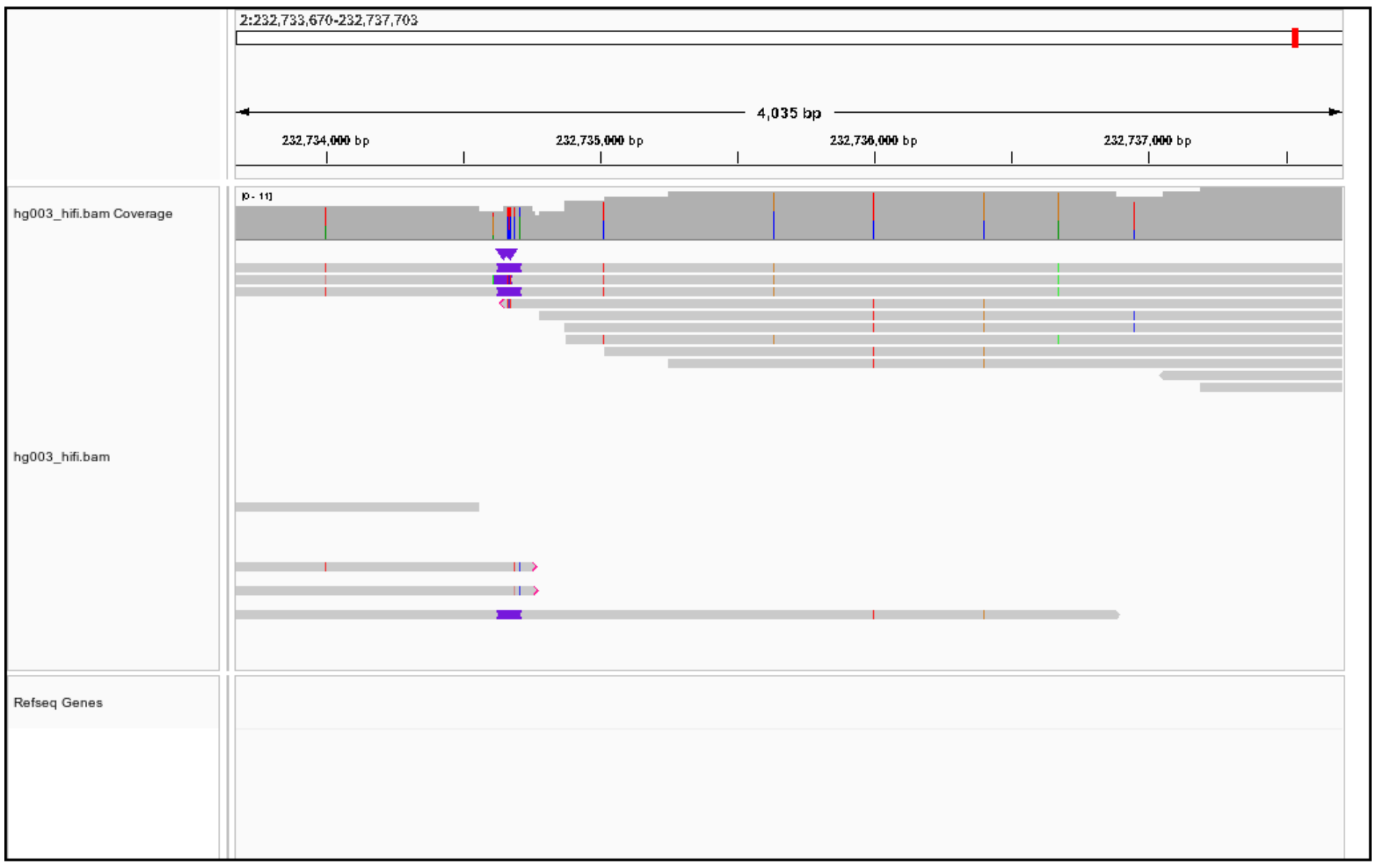

NA

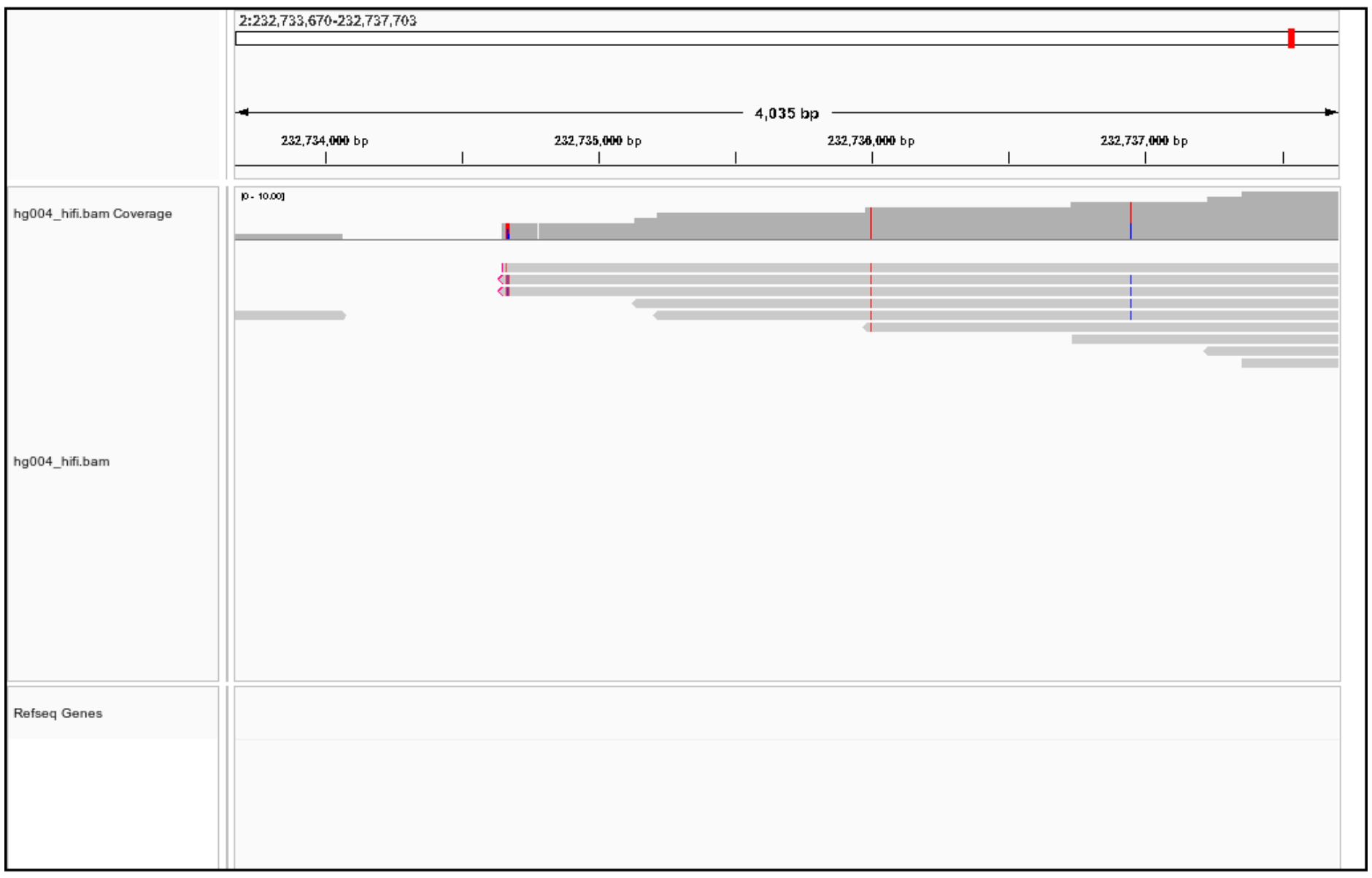

GIAB: NA

Sniffles2

Chr3: 85601516

62bp

INS

0/1

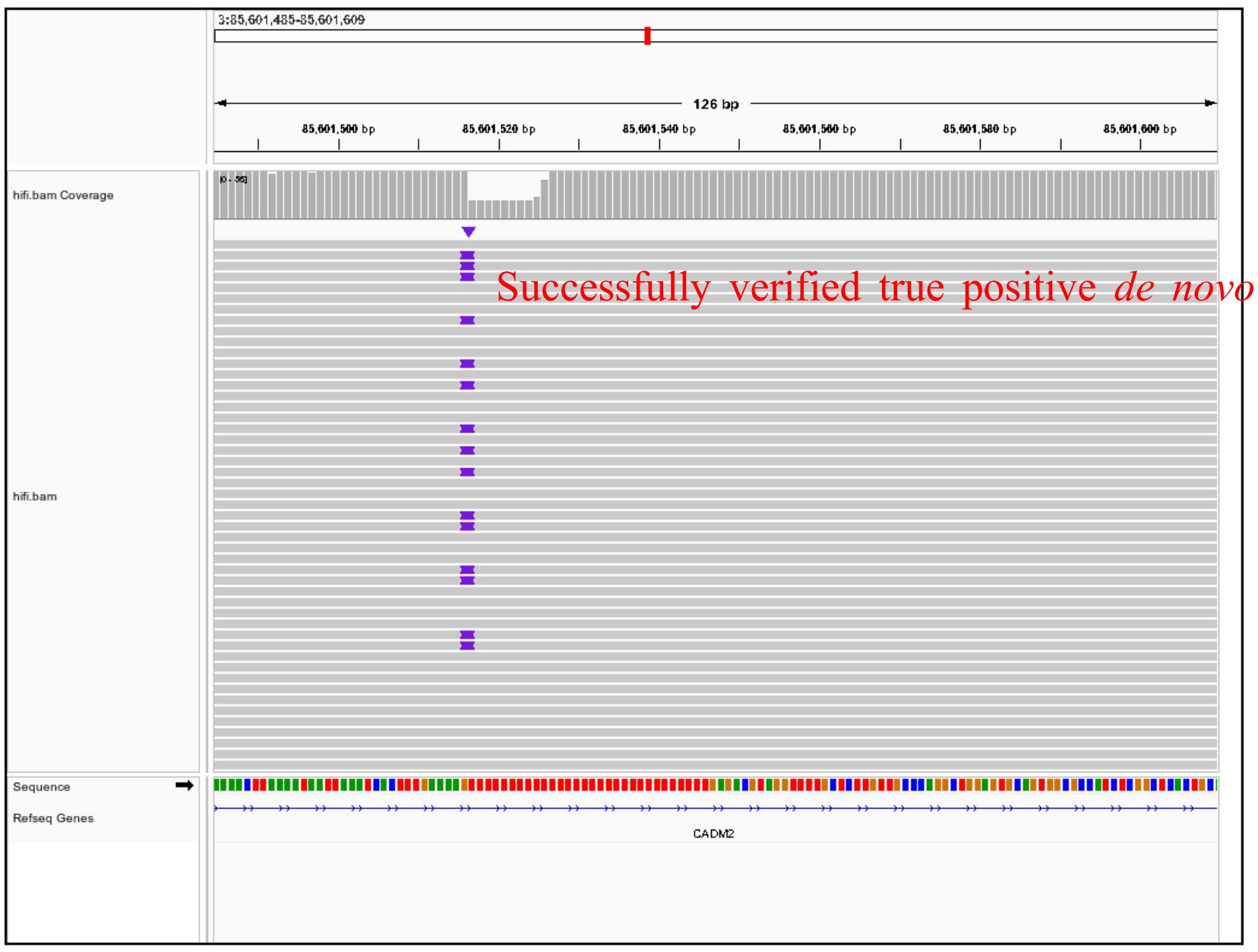

Successfully verified true positive *de novo* SV

NA

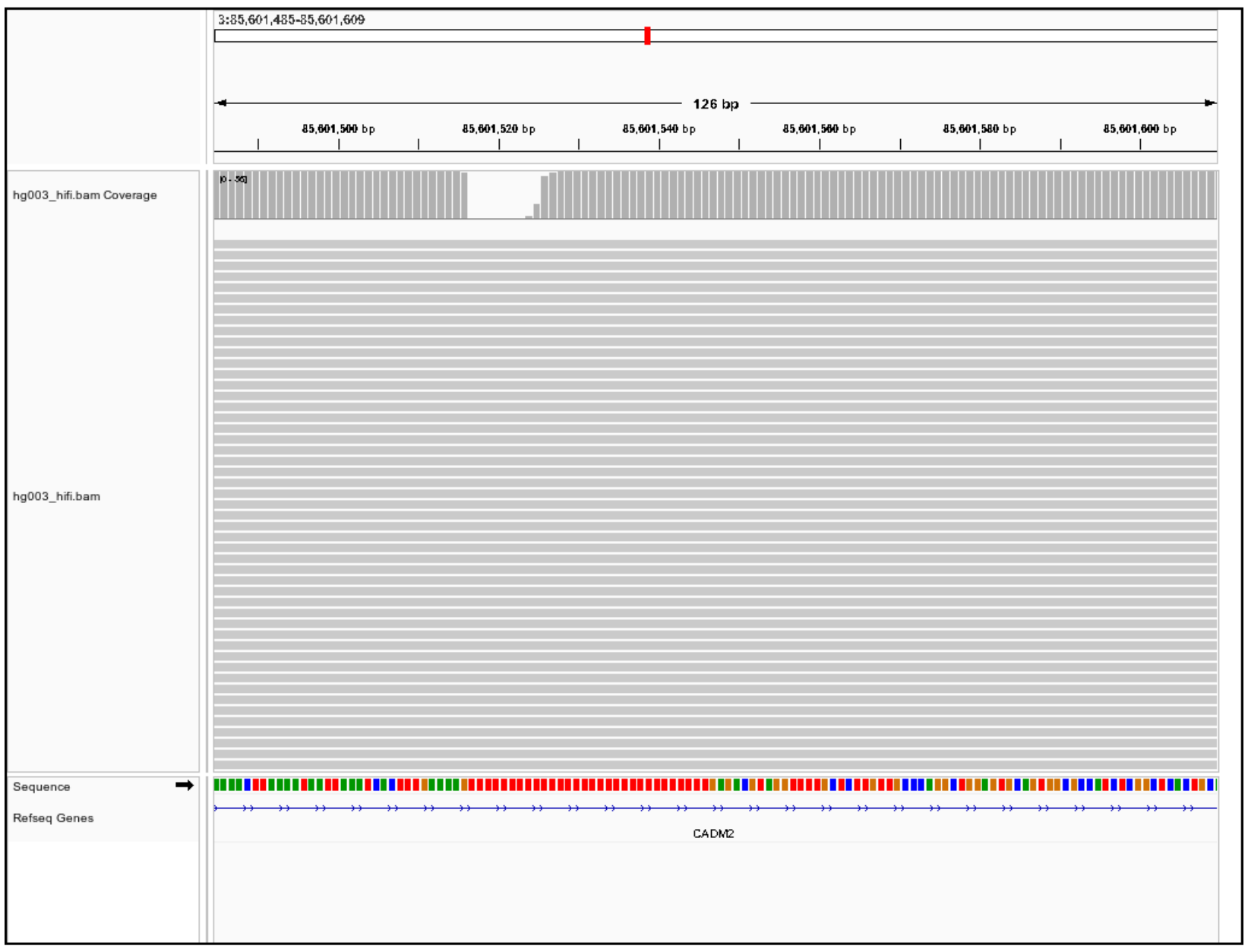

NA

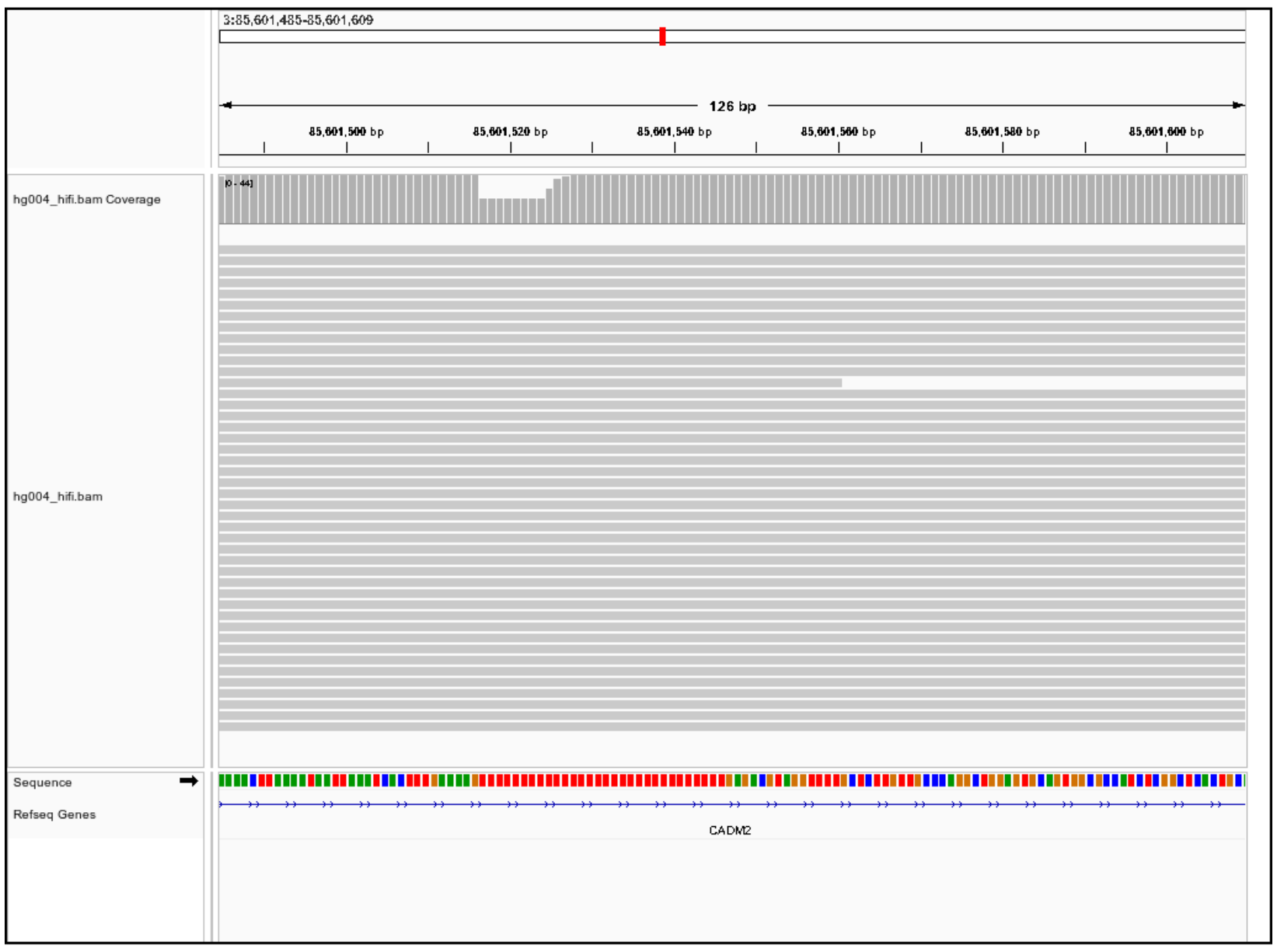

GIAB: Chr3: 120591471 50bp DEL HG002_GT= 0/1; HG003_GT= 0/1; HG004_GT=1/1

Sniffles2

Chr3: 120591471

50bp

DEL

0/1

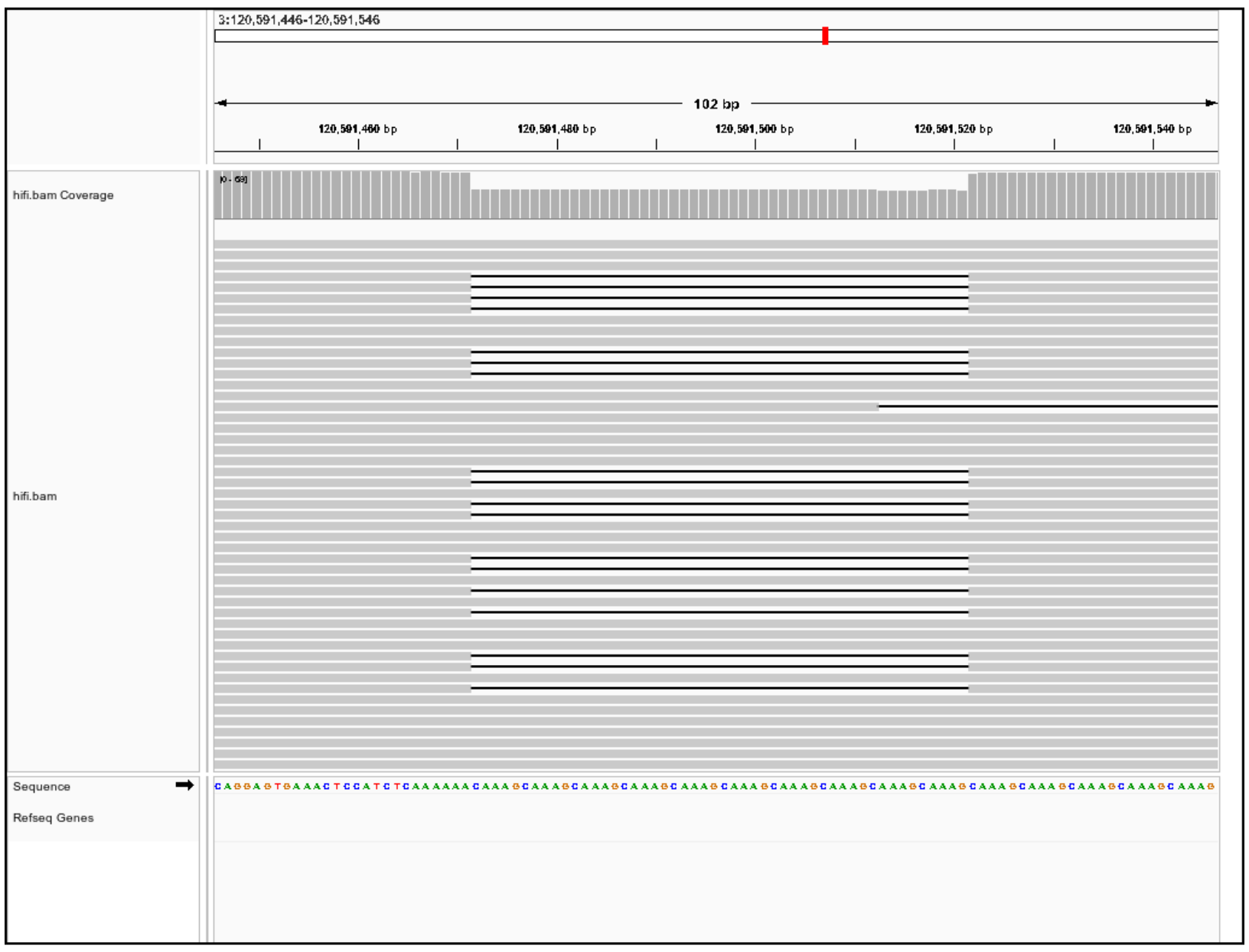

NA

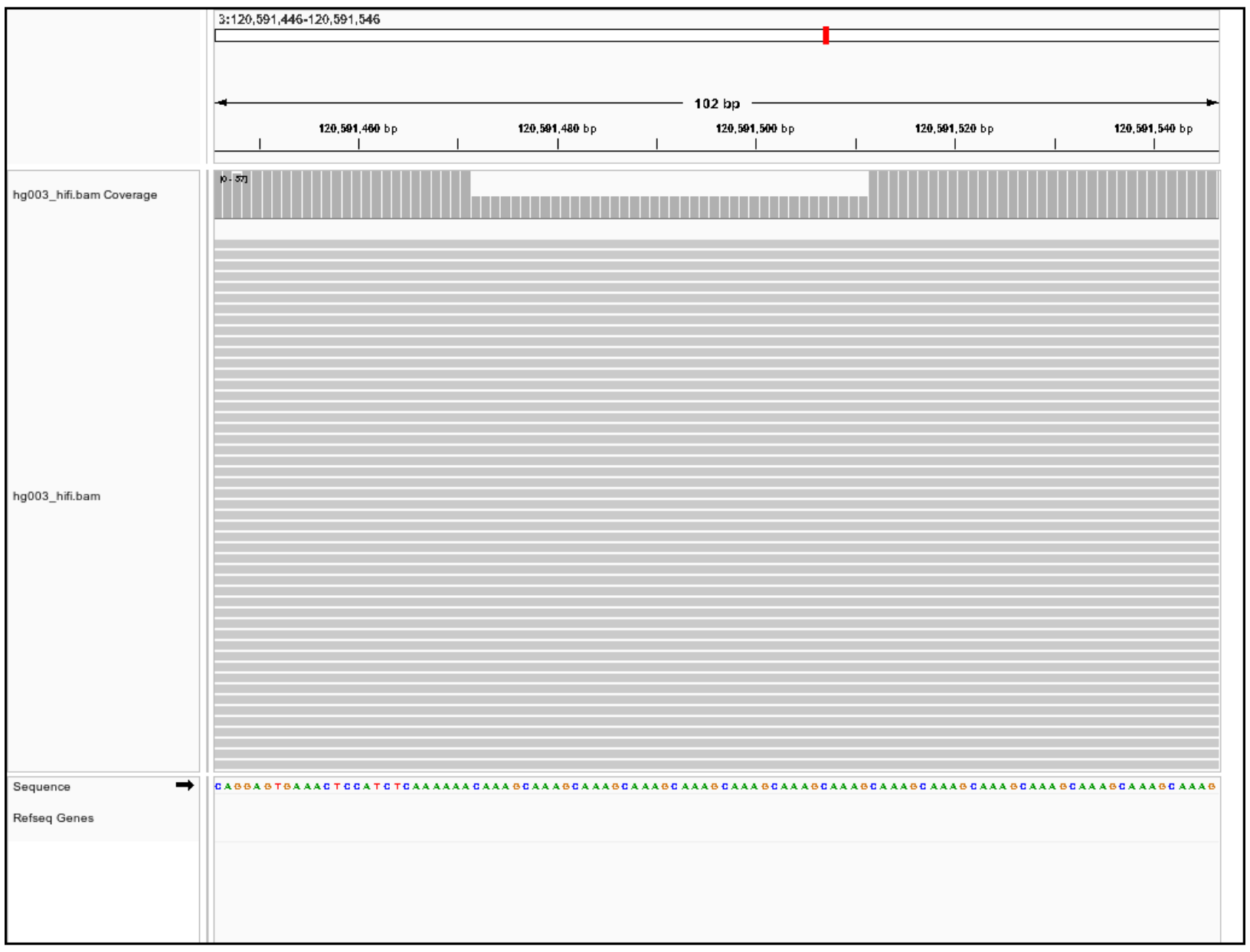

NA

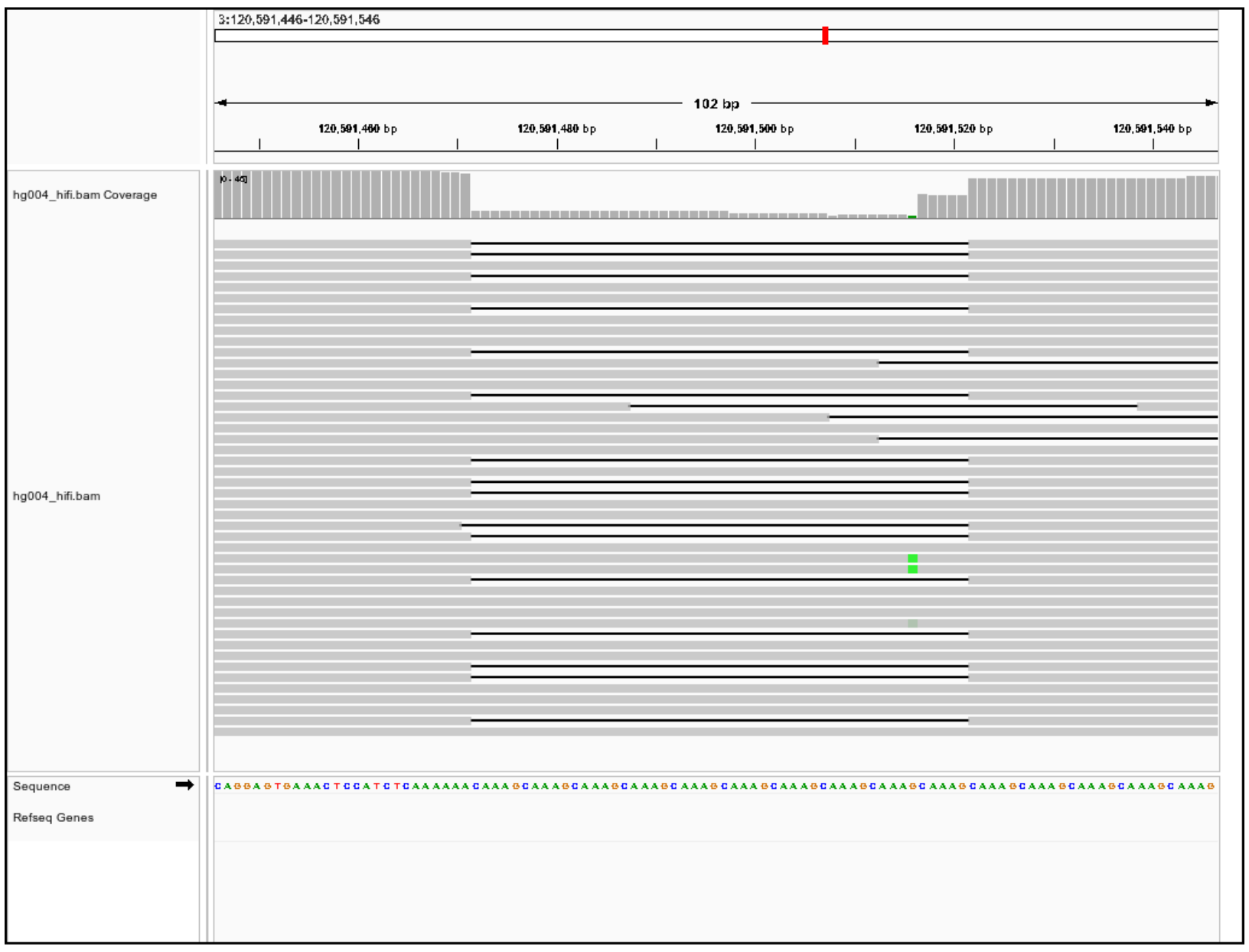

Sniffles2

Chr3: 130355726

68bp

INS

0/1

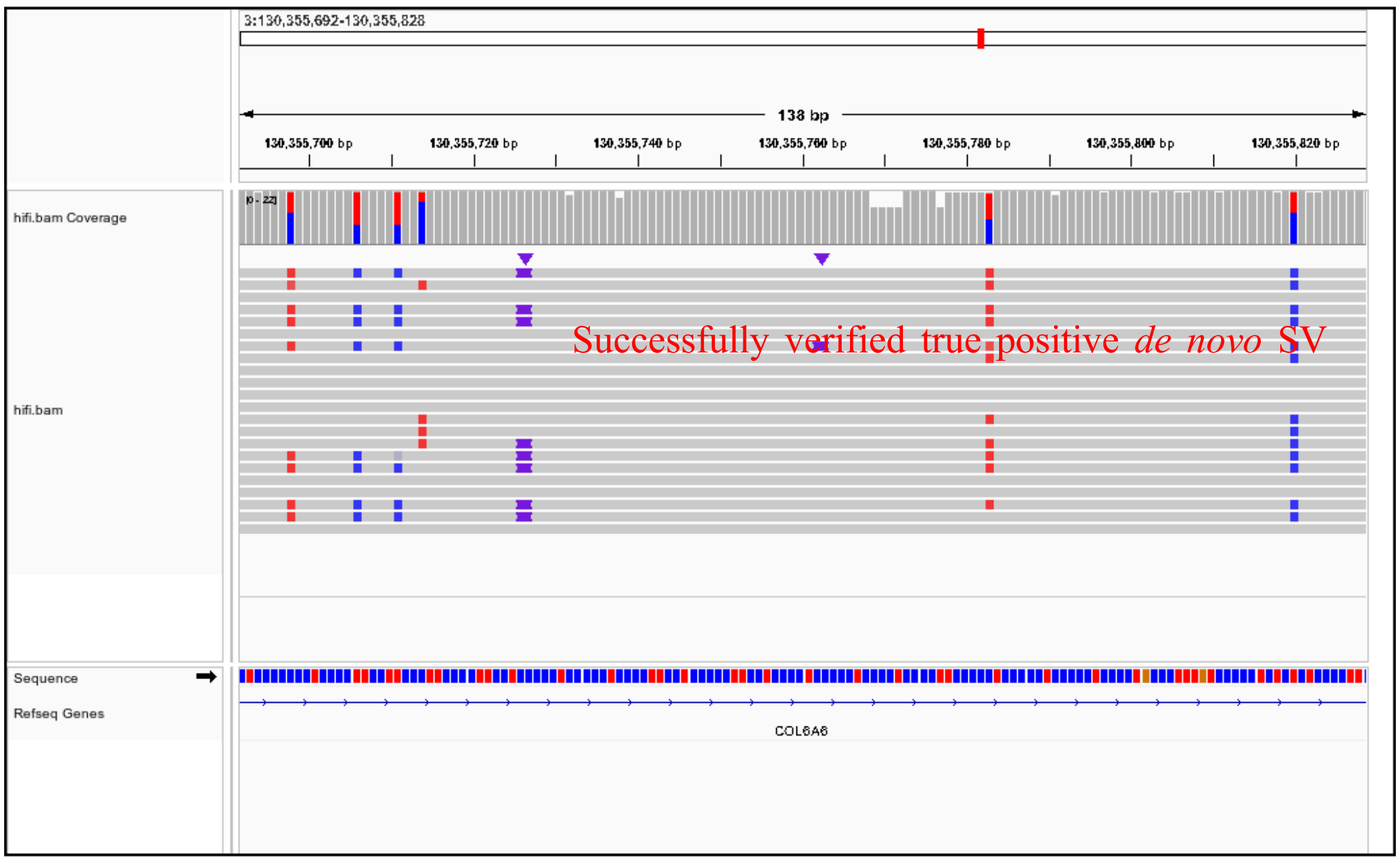

NA

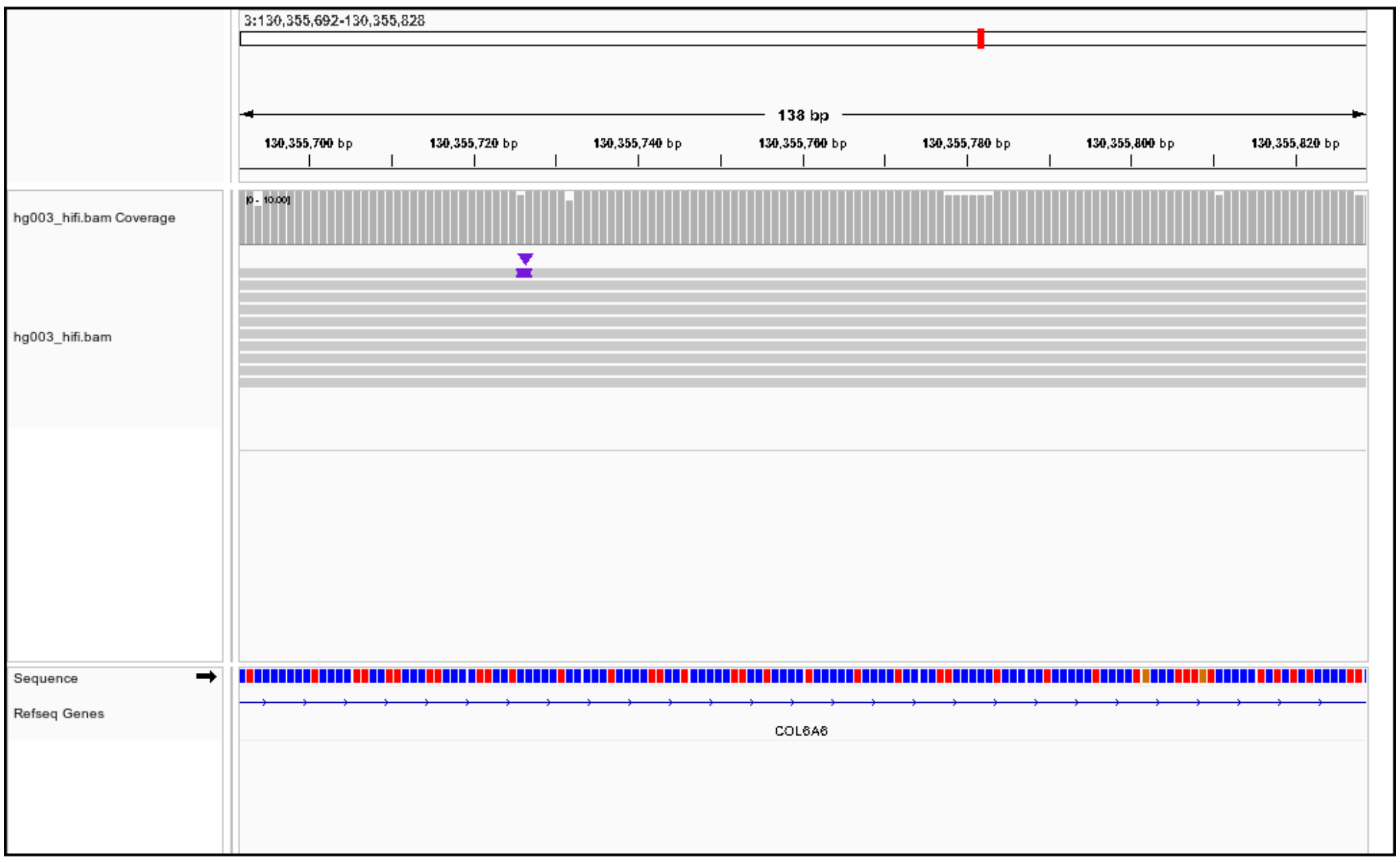

NA

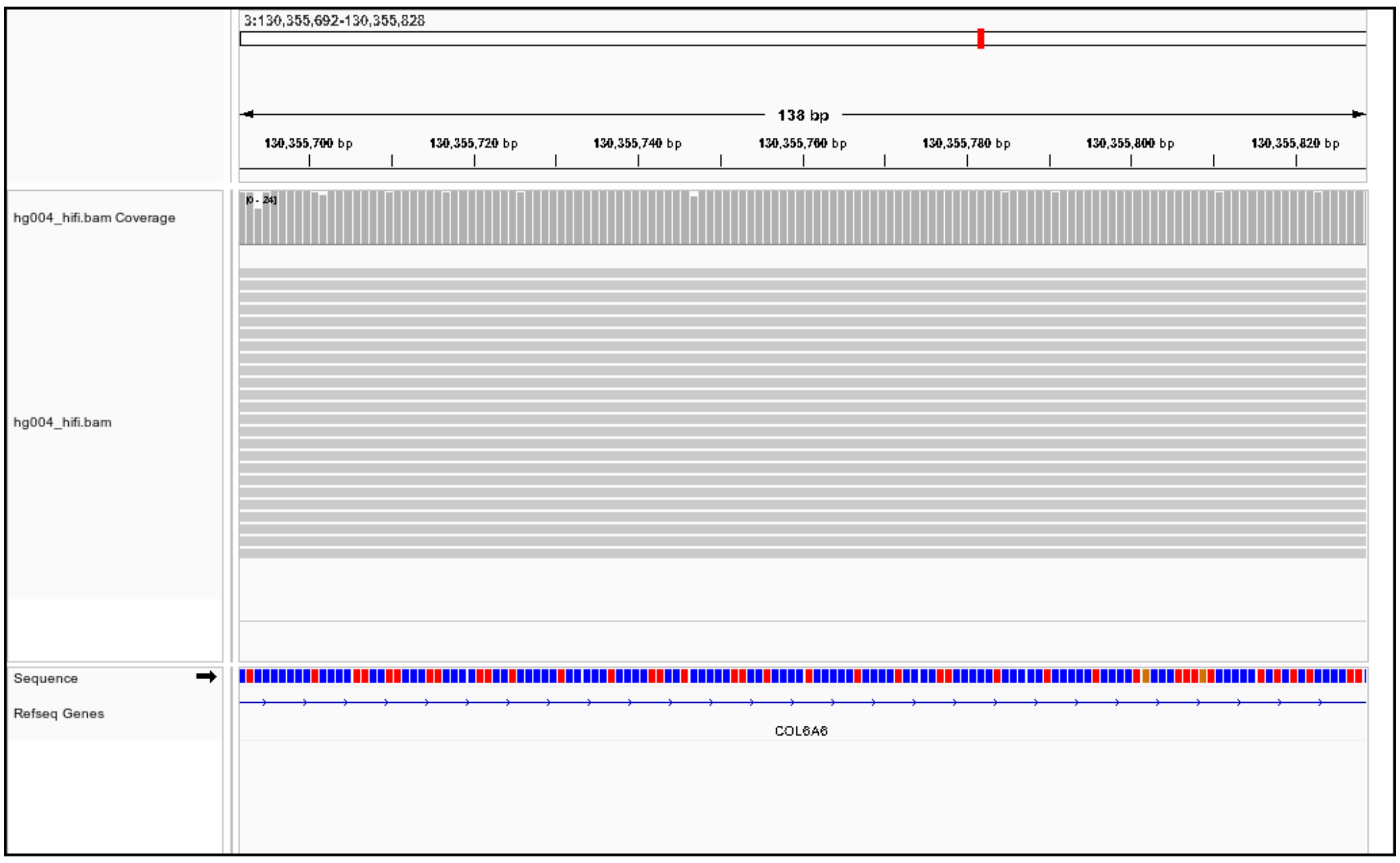

GIAB: Chr3: 196538064 3479bp INS HG002_GT= 1/1; HG003_GT= 1/1; HG004_GT=1/1

Sniffles2

Chr3: 196538072

2599bp

INS

1/1

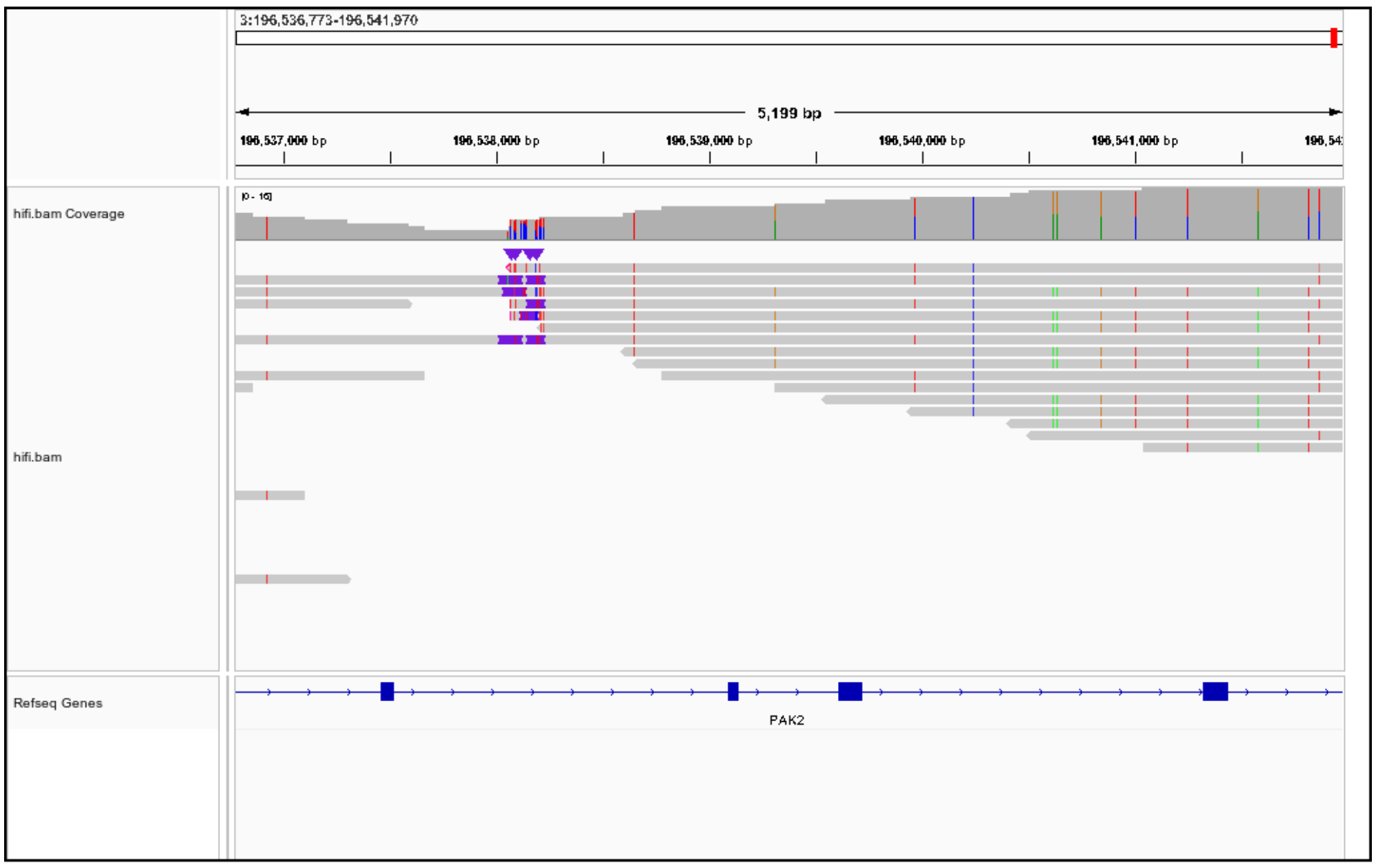

NA

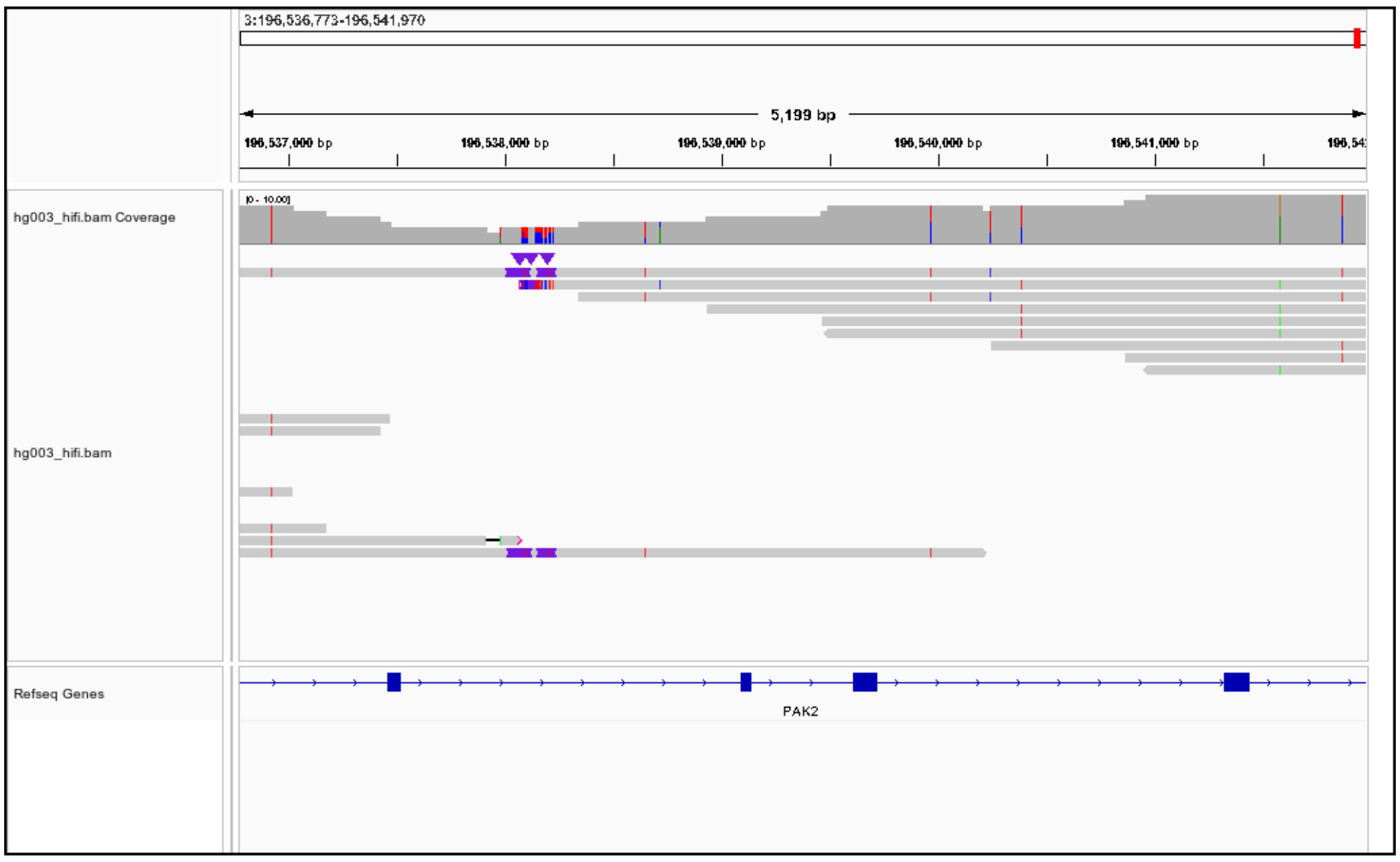

NA

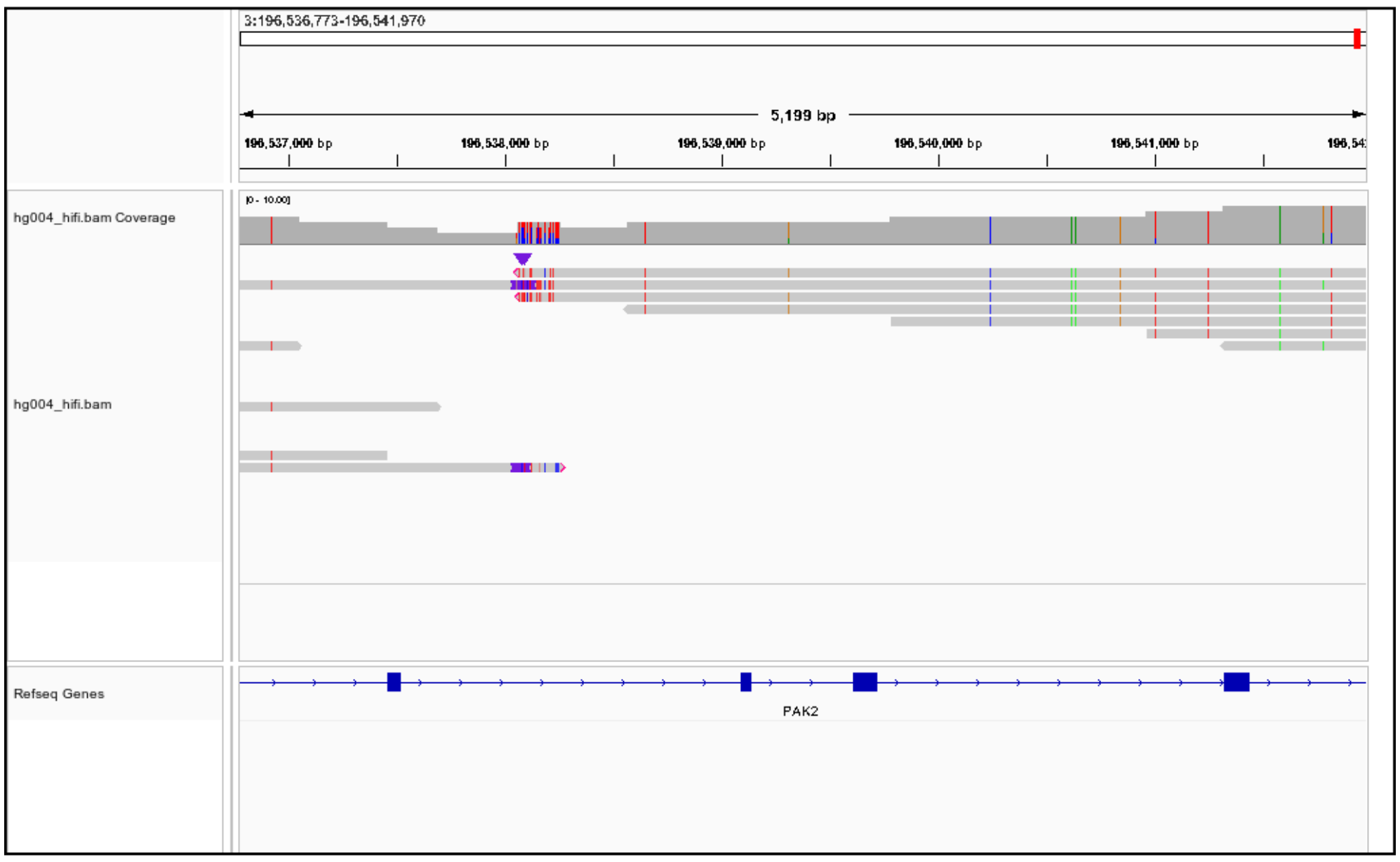

Supplementary Note 2. Validation of four SVPG-missed SVs in HG008 against the GIAB benchmark set.

Validation of seven SVs missed by SVPG based on the GIAB benchmark set, four of which are represented in the pangenome graph. The IGV screenshots support the credibility of the benchmark calls, while the Bandage visualizations illustrate the pangenome context in the corresponding regions (Supplementary Table 9 for overlap information detail).

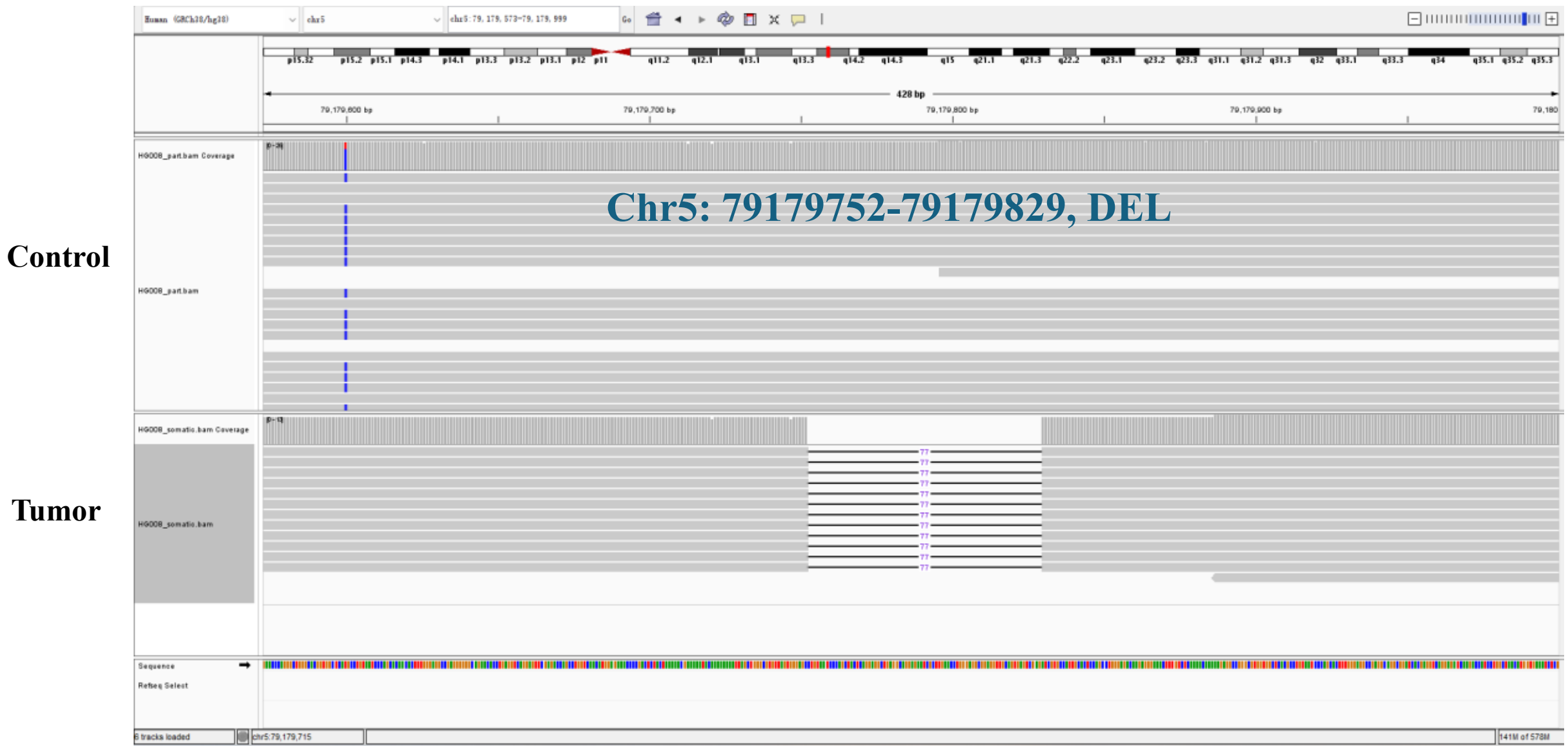

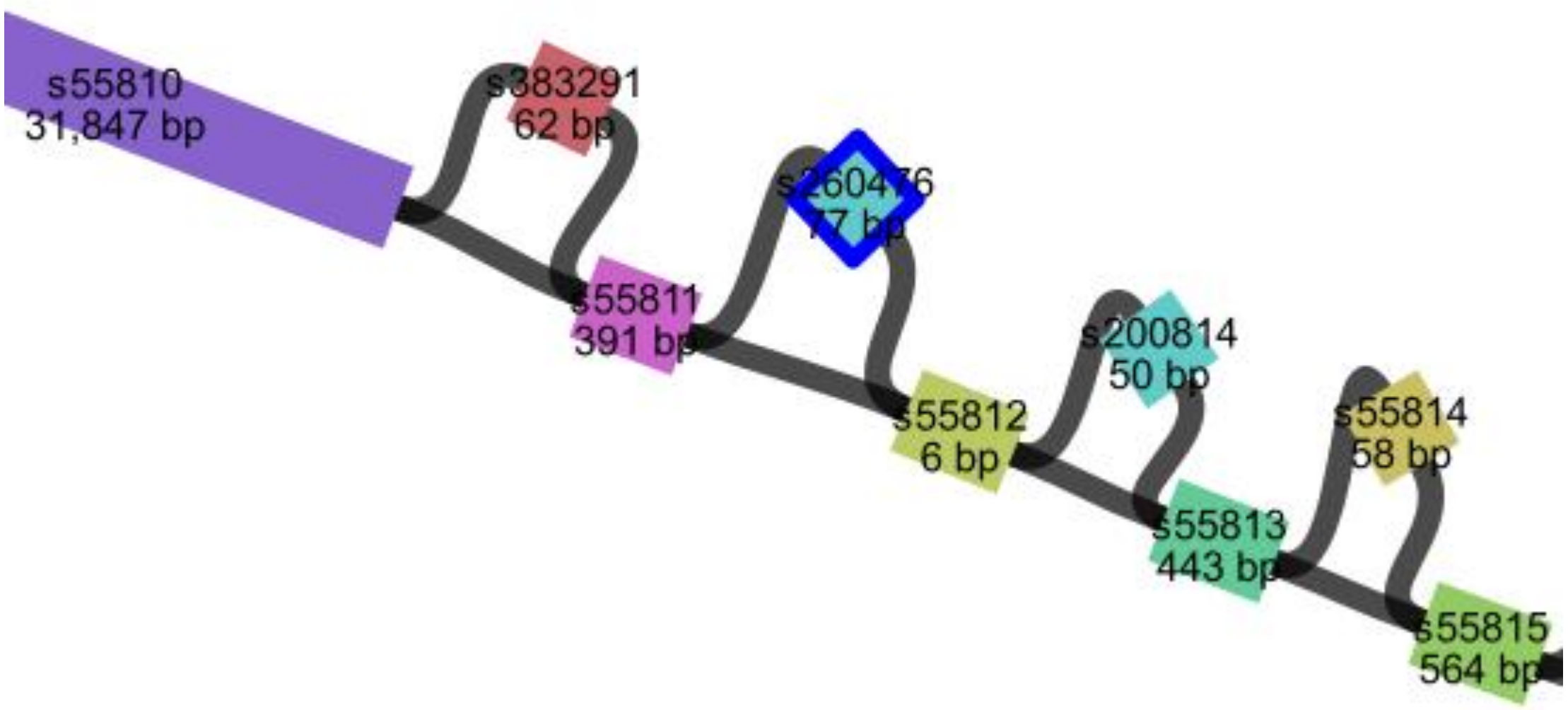

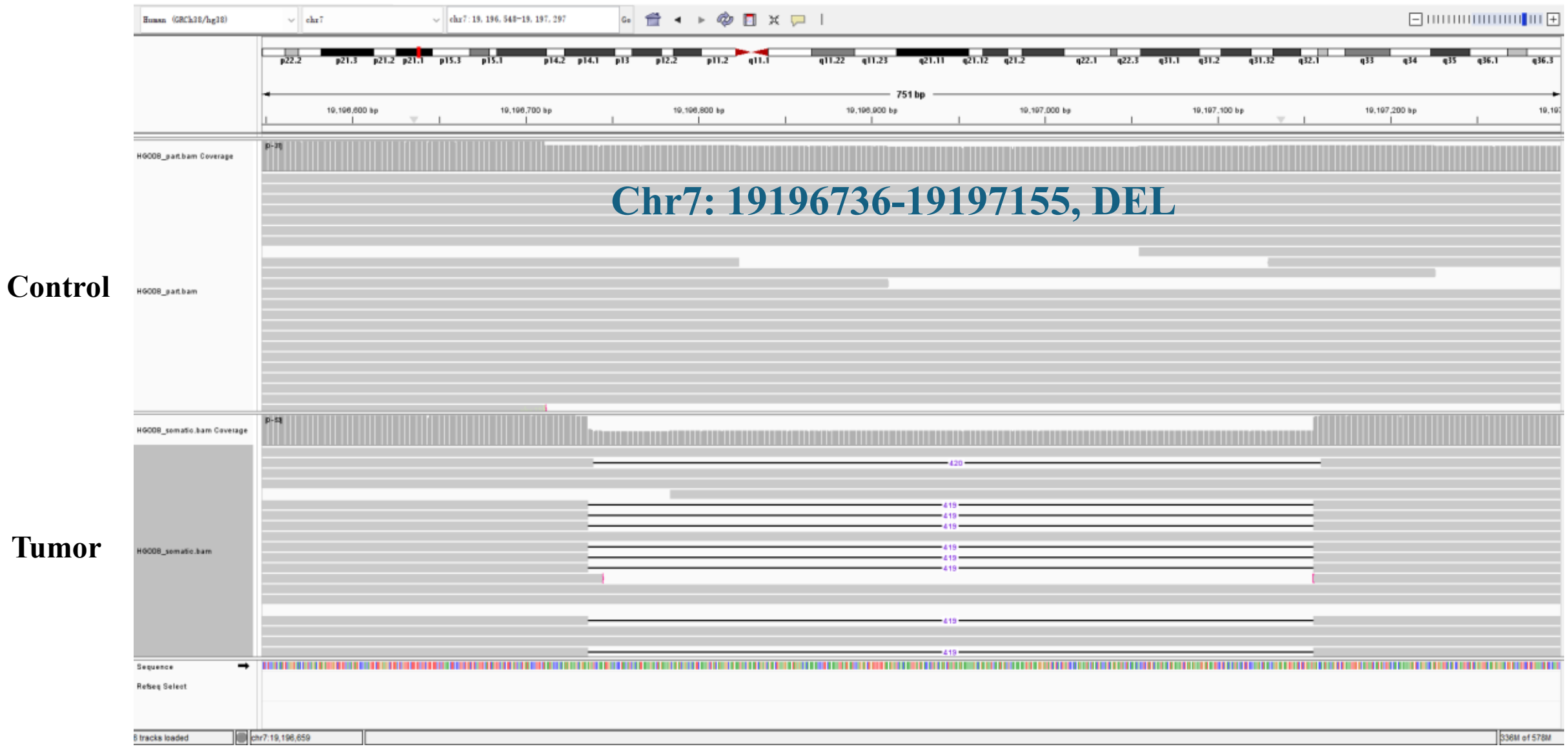
Chr7: 19196736-19197155, DEL
Control
Tumor

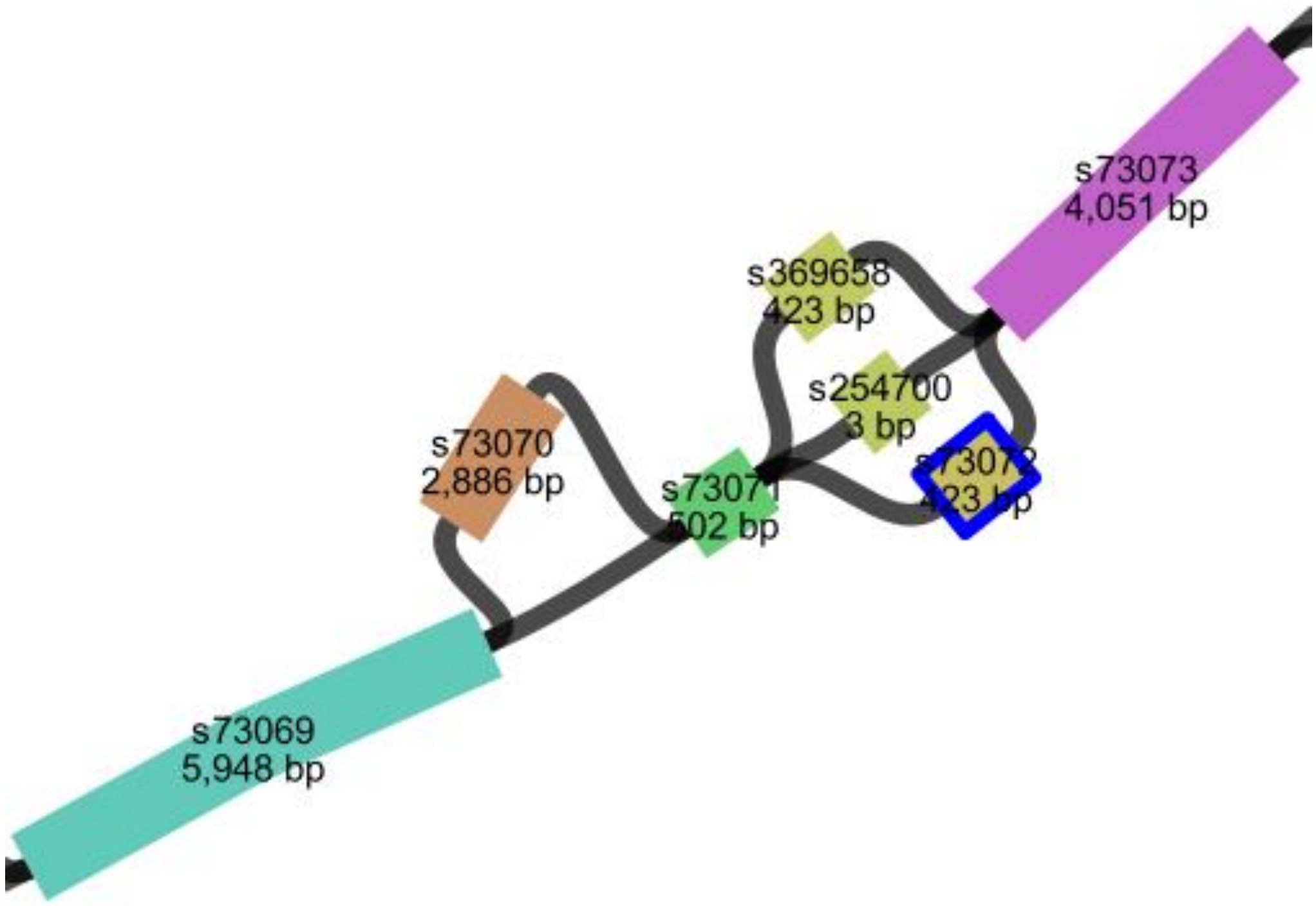
s73073
4,051 bp
s369658
423 bp
s254700
3 bp
s73070
2,886 bp
s73069
5,948 bp

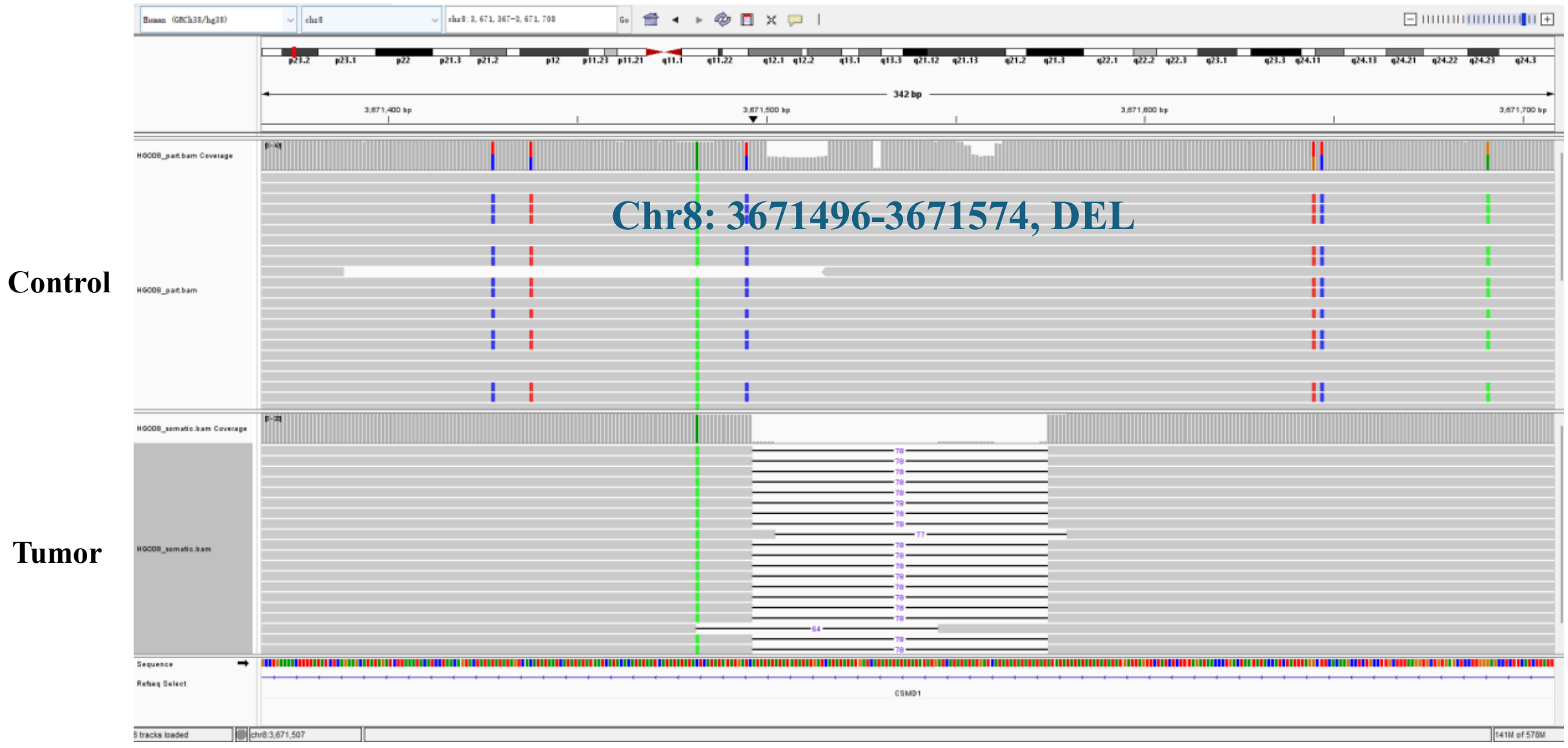
Chr8: 3671496-3671574, DEL
Control
Tumor

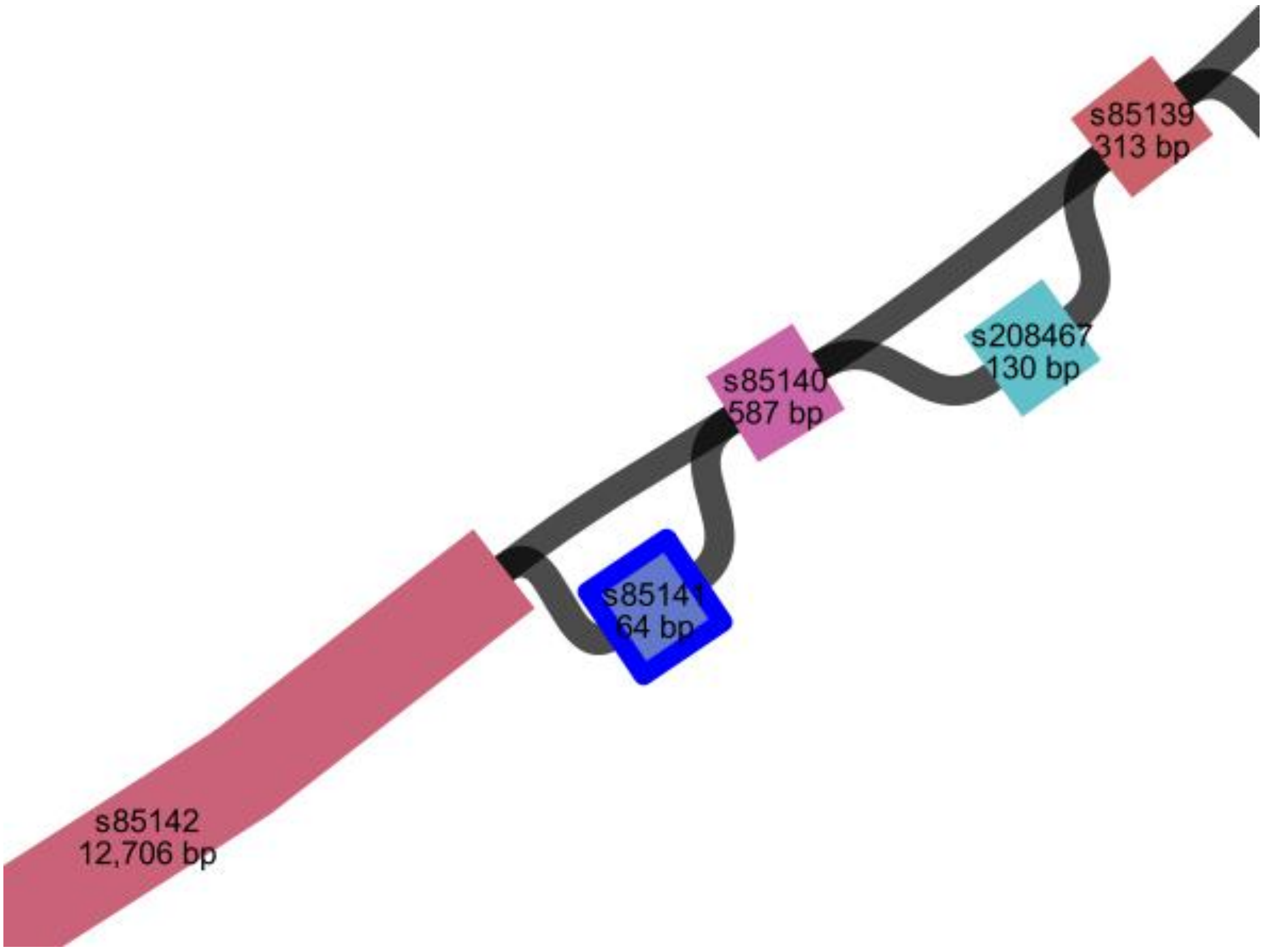
s85139
313 bp
s208467
130 bp
s85140
587 bp
s85141
64 bp
s85142
12,706 bp

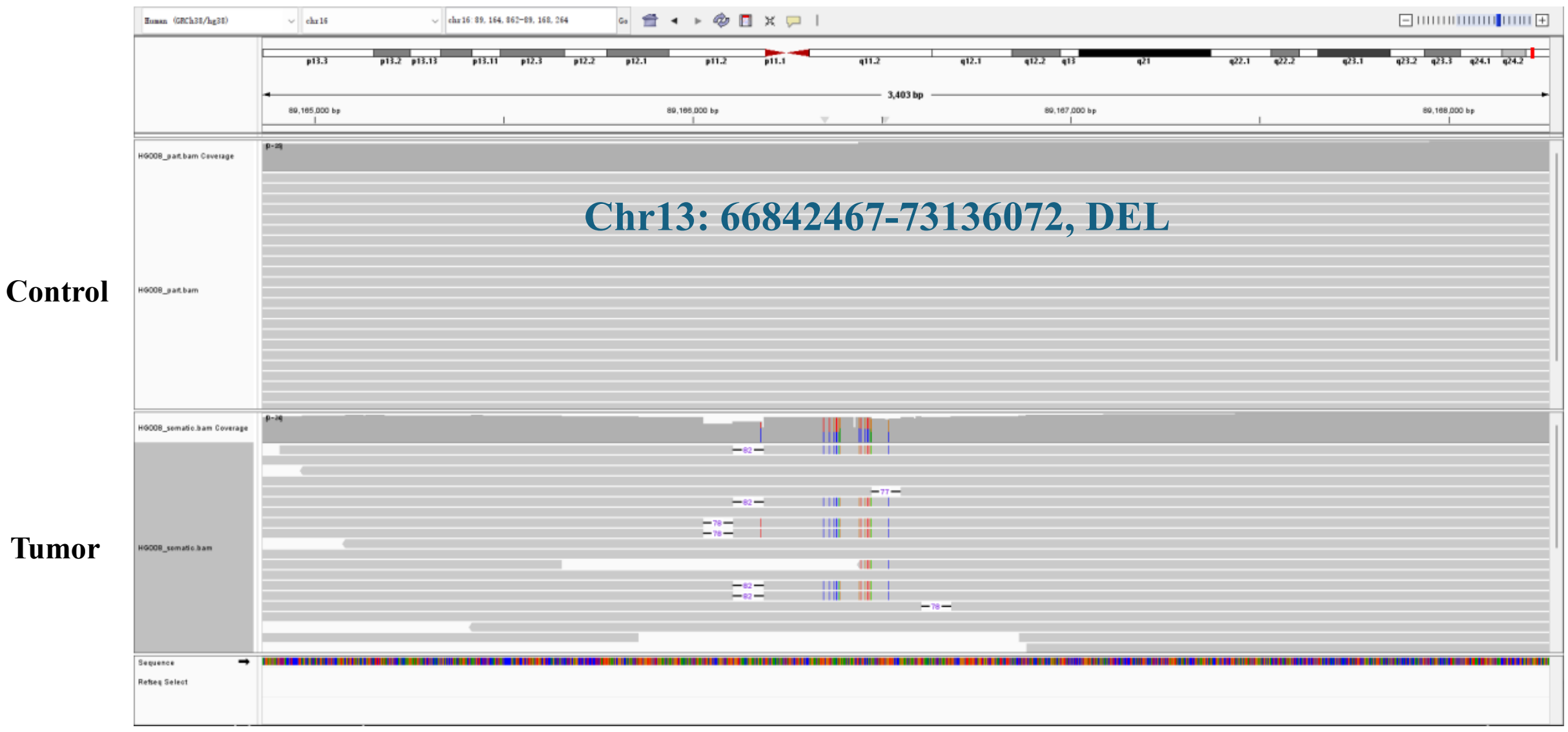
Chr13: 66842467-73136072, DEL
Control
Tumor

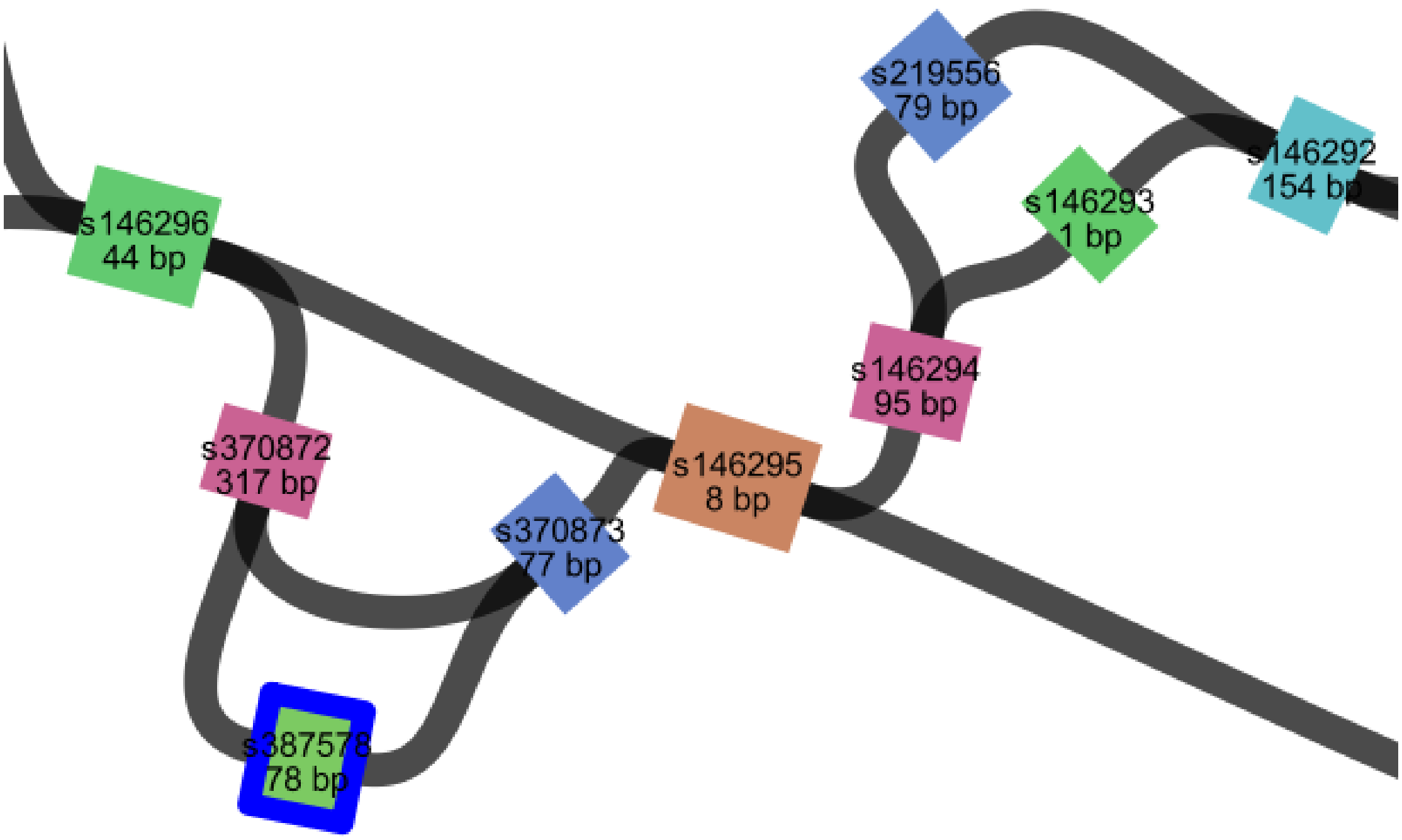
s219556
79 bp
s146292
154 bp
s146293
1 bp
s146296
44 bp
s146294
95 bp
s370872
317 bp
s146295
8 bp
s370873
77 bp
s387578
78 bp

Supplementary Note 3. Commands used

1 Command lines for each caller

SVPG (v1.3)

Pangenome-guided call mode:

SVPG call --working_dir /path/to/output --bam /path/to/sample.bam --ref /path/to/ref.fa --gfa /path/to/pangenome.gfa -s {min_support} -t 16

Pangenome-based mode (graph call mode):

SVPG graph-call --working_dir /path/to/output --ref /path/to/ref.fa --gaf /path/to/sample.gaf --gfa /path/to/pangenome.gfa -s {min_support} -t 16

Graph augment mode:

SVPG augment --working_dir /path/to/samples --ref /path/to/ref.fa --gfa /path/to/pangenome.gfa

Sniffles2 (v2.3.3)

sniffles --input /path/to/sample.bam --vcf /path/to/output.vcf -t 16

cuteSV (v2.1.1)

HiFi:

cuteSV /path/to/sample.bam /path/to/ref.fa /path/to/output/output.vcf /path/to/output -s {min-support} --genotype -t 16 --max_cluster_bias_INS 1000 --diff_ratio_merging_INS 0.9 --max_cluster_bias_DEL 1000 --diff_ratio_merging_DEL 0.5

ONT:

cuteSV /path/to/sample.bam /path/to/ref.fa /path/to/output/output.vcf /path/to/output -s {min-support} --genotype -t 16 --max_cluster_bias_INS 100 --diff_ratio_merging_INS 0.3 max_cluster_bias_DEL 100 --diff_ratio_merging_DEL 0.3

SVIM (v1.4.1)

svim alignment /path/to/output/variants.vcf /path/to/sample.bam /path/to/ref.fa

DeBreak (v1.2)

Debreak --bam /path/to/sample.bam --outpath /path/to/output --ref /path/to/ref.fa --poa --rescue_large_ins --rescue_dup -t 16

Sawfish (v2.0.3)

Discover step:

sawfish discover --threads 16 --ref /path/to/ref.fa --bam /path/to/sample.bam --output-dir /path/to/discover_dir

Joint-call step:

sawfish joint-call --threads 16 --sample /path/to/discover_dir --output-dir /path/to/output_call

miniSV (r29)

minisv.js extract -l 50 -b data/hs38.cen-mask.bed /path/to/sample.gaf > /path/to/variants.rsv;

cat /path/to/variants.rsv | sort -k1,1 -k2,2n -S4g | minisv.js merge -c {min_support} - >/path/to/variants.msv;

minisv.js genvcf /path/to/variants.msv > /path/to/ variants.vcf

SVarp (v1.0)

build/svarp -a /path/to/sample.gaf -g /path/to/pangenome.gfa --fasta /path/to/sample.fasta -o /path/to/output

manta (v1.6)

configManta.py --normalBam /path/to/normal_sample.bam --tumorBam /path/to/tumor_sample.bam --reference /path/to/ref.fa --runDir /path/to/output

nanomonsv (v0.7.2)

nanomonsv parse /path/to/tumor_sample.bam /path/to/output/test_tumor

nanomonsv parse /path/to/normal_sample.bam /path/to/output/test_normal

nanomonsv get /path/to/tumor_sample.bam /path/to/normal_sample.bam /path/to/ref.fa --control_prefix /path/to/output/test_tumor --control_bam /path/to/output/test_normal

Severus (v1.5)

python severus.py --target-bam /path/to/tumor_sample.bam --control-bam /path/to/normal_sample.bam --out-dir /path/to/output -t 16 --phasing-vcf /path/to/phased_vcf --vntr-bed vntr_bed

The "*min-support*" parameter of minimum supporting reads note: For the HG002 sample's HiFi 48×/20×/10×/5× data, "*min-support*" were set to 4/2/1. For the HG002 sample's ONT 47×/20×/10×/5× data, "*min-support*" were set to 10/4/3/2. For the HG003/HG004 sample's HiFi and ONT data, "*min-support*" were set to 4 and 10. For the HG005/HG006/HG007

sample's HiFi and ONT data, "*min-support*" were set to 3 and 10. For rare SVs simulated HiFi and ONT data, "*min-support*" were set to 4 for both. For HG008 and HCC$_{1395}$ sample's HiFi data, "min-support" were set to 3 and 4.

2 Command lines of SV call eval

bgzip /path/to/variants.vcf && tabix /path/to/variants.vcf.gz;

For germline SV benchmark: truvari bench -f /path/to/ref.fa -b /path/to/ground_truth.vcf.gz -o /path/to/tools --sizemin 50 --sizefilt 50 --passonly -p 0.00 -P 0.5 -r 1000 -c /path/to/variants.vcf.gz --includebed /path/to/include.bed

For somatic SV benchmark: truvari bench -f /path/to/ref.fa -b /path/to/ground_truth.vcf.gz -o /path/to/tools --pick multi --sizemin 50 --sizefilt 50 --sizemax -1 --passonly -p 0.00 --dup-to-ins --typeignore -c /path/to/variants.vcf.gz --includebed /path/to/include.bed

python /minda/minda.py truthset --base /path/to/ground_truth.vcf.gz --vcfs *.variants.vcf.gz --out_dir minda_multimatch --multimatch --bed /path/to/include.bed

3 Command lines for alignment

minimap2 (v2.17-r941)

minimap2 /path/to/ref.fa /path/to/sample.fasta -x map-hifi --MD -Y -o /path/to/sample.sam -R '@RG\tID:hg2' -t 64 && samtools view -@ 64 -buS /path/to/sample.sam | samtools sort -@ 64 -O bam -T ./ - > /path/to/sample.bam

pbmm2 (v1.17.0)

pbmm2 align /path/to/ref.fa /path/to/sample.fasta /path/to/sample.bam --sort --num-threads 64

minigraph (v0.21-r606)

HiFi: *minigraph -t128 -cxasm --vc --secondary yes /path/to/pangenome.gfa /path/to/sample.fasta > /path/to/sample.gaf*

ONT: *minigraph -t128 -cxlr --vc --secondary yes /path/to/pangenome.gfa /path/to/sample.fasta > /path/to/sample.gaf*

For miniSV (stable segment coordinate): *minigraph -t128 -cx asm(lr) /path/to/pangenome.gfa /path/to/sample.fasta > /path/to/sample.gaf*

4 Command lines for down-sample

HiFi-20×: *samtools view -@ 64 -bS -s 0.417 /path/to/sample.bam >*

/path/to/sample_20×.bam

HiFi-10×: *samtools view -@ 64 -bS -s 0.208 /path/to/sample.bam > /path/to/sample_10×.bam*

HiFi-5×: *samtools view -@ 64 -bS -s 0.104 /path/to/sample.bam > /path/to/sample_5×.bam*

ONT-20× and ONT-20×_replicate: *samtools view -@64 -bS -s 0.426 /path/to/sample.bam > /path/to/sample_20×.bam && samtools view -@64 -bS -s 0.426 /path/to/sample.bam –subsample-seed 99 > /path/to/sample_20×_replicate.bam*

ONT-10× and ONT-10×_replicate: *samtools view -@64 -bS -s 0.213 /path/to/sample.bam > /path/to/sample_10×.bam && samtools view -@64 -bS -s 0.213 /path/to/sample.bam –subsample-seed 99 > /path/to/sample_10×_replicate.bam*

ONT-5× and ONT-5×_replicate: *samtools view -@64 -bS -s 0.106 /path/to/sample.bam > /path/to/sample_5×.bam && samtools view -@64 -bS -s 0.106 /path/to/sample.bam –subsample-seed 99 > /path/to/sample_5×_replicate.bam*

5 Command lines for reads simulation of rare SV

PBSIM3 (v3.0.4)

ONT: *pbsim --strategy wgs --method qshmm --qshmm R103.model --depth 20 --genome /path/to/sample.fasta*

HiFi: *pbsim --strategy wgs --method sample --sample /path/to/sample.fastq --depth 20 --genome /path/to/sample.fasta*
