# Supplementary material for: SVPG: a pangenome-based structural variant detection approach and rapid augmentation of pangenome graphs with new samples
Source: Nat Methods. 2026 Sep 21;23(10):1984–93. doi: 10.1038/s41592-026-03219-2 (PMC13645633; doi:10.1038/s41592-026-03219-2)
Supplement: Supplementary file 2 [file 41592_2026_3219_MOESM2_ESM.pdf]

nature portfolio

Corresponding author(s): Guohua Wang

Last updated by author(s): Apr 27, 2026

Reporting Summary

Nature Portfolio wishes to improve the reproducibility of the work that we publish. This form provides structure for consistency and transparency in reporting. For further information on Nature Portfolio policies, see our Editorial Policies and the Editorial Policy Checklist.

Statistics

For all statistical analyses, confirm that the following items are present in the figure legend, table legend, main text, or Methods section.

n/a	Confirmed	
☐	☒	The exact sample size (n) for each experimental group/condition, given as a discrete number and unit of measurement
☐	☒	A statement on whether measurements were taken from distinct samples or whether the same sample was measured repeatedly
☐	☒	The statistical test(s) used AND whether they are one- or two-sided<br>*Only common tests should be described solely by name; describe more complex techniques in the Methods section.*
☒	☐	A description of all covariates tested
☒	☐	A description of any assumptions or corrections, such as tests of normality and adjustment for multiple comparisons
☐	☒	A full description of the statistical parameters including central tendency (e.g. means) or other basic estimates (e.g. regression coefficient) AND variation (e.g. standard deviation) or associated estimates of uncertainty (e.g. confidence intervals)
☐	☒	For null hypothesis testing, the test statistic (e.g. F, t, r) with confidence intervals, effect sizes, degrees of freedom and P value noted<br>*Give P values as exact values whenever suitable.*
☒	☐	For Bayesian analysis, information on the choice of priors and Markov chain Monte Carlo settings
☒	☐	For hierarchical and complex designs, identification of the appropriate level for tests and full reporting of outcomes
☒	☐	Estimates of effect sizes (e.g. Cohen's d, Pearson's r), indicating how they were calculated

Our web collection on statistics for biologists contains articles on many of the points above.

Software and code

Policy information about availability of computer code

Data collection	No software was used to collect the data.
Data analysis	The software stack developed for the method proposed in this work (SVPG) is available on GitHub under the MIT license at: https://github.com/coopsor/SVPG. SVPG was developed in Python3.10. In addition we used the following third-party software for (1) sequence alignment and visualization: minimap2 v2.17, pbmm2 v1.7.0, minigraph v0.21, samtools v1.9, IGV v2.9.4; (2) SV calling: sniffles v2.3.3, cuteSV v2.1.1, SVIM v1.4.1, Debreak v1.2, sawfish v2.0.3, miniSV, SVarp v.1.0, manta v1.6, nanomonsv v0.7.2, severus v1.5 (3) read simulation: pbsim3 v3.0.4; (4) benchmarking and SV comparisons: Truvari v5.3, minda, SURVIVOR 1.0.7, bcftools v1.14, bedtools v2.31.1, gfatools v0.5.

For manuscripts utilizing custom algorithms or software that are central to the research but not yet described in published literature, software must be made available to editors and reviewers. We strongly encourage code deposition in a community repository (e.g. GitHub). See the Nature Portfolio guidelines for submitting code & software for further information.

Data

Policy information about availability of data

All manuscripts must include a data availability statement. This statement should provide the following information, where applicable:

- Accession codes, unique identifiers, or web links for publicly available datasets
- A description of any restrictions on data availability
- For clinical datasets or third party data, please ensure that the statement adheres to our policy

Sequencing and alignment data:
GIAB HG002 HiFi data publicly available at https://ftp-trace.ncbi.nlm.nih.gov/giab/ftp/data/AshkenazimTrio/HG002_NA24385_son/PacBio_HiFi-Revio_20231031/. HG002 ONT: https://ftp-trace.ncbi.nlm.nih.gov/giab/ftp/data/AshkenazimTrio/HG002_NA24385_son/UCSC_Ultralong_OxfordNanopore_Promethion/. Ashkenazim trio: https://ftp-trace.ncbi.nlm.nih.gov/giab/ftp/data/AshkenazimTrio/. Chinese trio: https://ftp-trace.ncbi.nlm.nih.gov/giab/ftp/data/ChineseTrio/. HG008 HiFi: https://ftp-trace.ncbi.nlm.nih.gov/giab/ftp/data_somatic/HG008/Liss_lab/PacBio_Revio_20240125/. HG008 Illumina: https://ftp-trace.ncbi.nlm.nih.gov/giab/ftp/data_somatic/HG008/Liss_lab/BCM_Illumina-WGS_20240313/. HCC1395 and COLO829: https://www.pacb.com/connect/datasets/. 20 HPRC samples: https://s3-us-west-2.amazonaws.com/human-pangenomics/index.html?prefix=working/HPRC/.
Benchmark dataset:
HG002 genome wide: https://ftp-trace.ncbi.nlm.nih.gov/ReferenceSamples/giab/release/AshkenazimTrio/HG002_NA24385_son/NIST_SV_v0.6/ and https://ftp-trace.ncbi.nlm.nih.gov/ReferenceSamples/giab/data/AshkenazimTrio/analysis/NIST_HG002_DraftBenchmark_defrabbV0.019-20241113/. HG002 medical regions: https://ftp-trace.ncbi.nlm.nih.gov/ReferenceSamples/giab/release/AshkenazimTrio/HG002_NA24385_son/CMRG_v1.00/. Rare SV: https://github.com/hall-lab/sv_paper_042020/blob/master/Supplementary_File_1.zip. Repeats region: https://genome.ucsc.edu/cgi-bin/hgTables. HGSVC3: https://ftp.1000genomes.ebi.ac.uk/vol1/ftp/data_collections/HGSVC3/release/Variant_Calls/1.0/GRCh38/. HG008: https://ftp-trace.ncbi.nlm.nih.gov/ReferenceSamples/giab/data_somatic/HG008/Liss_lab/analysis/NIST_HG008-T_somatic-stvar_DraftBenchmark_V0.3-20250220/. Consensus benchmark set of HCC1395: https://static-content.springer.com/esm/art%3A10.1186%2Fs13059-022-02816-6/MediaObjects/13059_2022_2816_MOESM4_ESM.xlsx. Validation benchmark set of HCC1395: https://static-content.springer.com/esm/art%3A10.1186%2Fs13059-022-02816-6/MediaObjects/13059_2022_2816_MOESM5_ESM.xlsx. Validation benchmark set of COLO829: https://doi.org/10.5281/zenodo.13917379.
Reference genome:
GRCh38: http://ftp.1000genomes.ebi.ac.uk/vol1/ftp/technical/reference/GRCh38_reference_genome/. GRCh37: http://ftp-trace.ncbi.nih.gov/1000genomes/ftp/technical/reference/phase2_reference_assembly_sequence/hs37d5.fa.gz. GRCh38-90c.r518 and GRCh37-91c.r559: https://zenodo.org/records/10693675.
The VCF outputs of all tools, augment pangenome graph, and benchmark scripts are available at https://doi.org/10.5281/zenodo.18456502.

Research involving human participants, their data, or biological material

Policy information about studies with human participants or human data. See also policy information about sex, gender (identity/presentation), and sexual orientation and race, ethnicity and racism.

Reporting on sex and gender	Not relevant to our study
Reporting on race, ethnicity, or other socially relevant groupings	Not relevant to our study
Population characteristics	Not relevant to our study
Recruitment	Not relevant to our study
Ethics oversight	Not relevant to our study

Note that full information on the approval of the study protocol must also be provided in the manuscript.

Field-specific reporting

Please select the one below that is the best fit for your research. If you are not sure, read the appropriate sections before making your selection.

☒ Life sciences ☐ Behavioural & social sciences ☐ Ecological, evolutionary & environmental sciences

For a reference copy of the document with all sections, see nature.com/documents/nr-reporting-summary-flat.pdf

Life sciences study design

All studies must disclose on these points even when the disclosure is negative.

Sample size	The sample size is the one determined by the publicly available human long-read sequencing data from Genome In A Bottle (GIAB) consortium and the Human Pangenome Reference Consortium (HPRC) for this study.
Data exclusions	No data were excluded. We have used publicly available human long-read sequencing data from Genome in A Bottle (GIAB) consortium and the Human Pangenome Reference Consortium (HPRC) consortium for this study.

Replication	Not applicable since this study describes a deterministic algorithms without statistical analysis. Moreover, this study does not involve wet lab experiments.
Randomization	Not applicable since this study have no experimental or control groups. Moreover, this study introduces a method and does not include biological hypothesis analysis.
Blinding	Not applicable since this study does not involve statistical analysis and data acquisition. The method introduced in this study and all softwares used in the experimental evaluation are deterministic and do not take advantages from knowning the origin of the input data.

Reporting for specific materials, systems and methods

We require information from authors about some types of materials, experimental systems and methods used in many studies. Here, indicate whether each material, system or method listed is relevant to your study. If you are not sure if a list item applies to your research, read the appropriate section before selecting a response.

Materials & experimental systems

n/a	Involved in the study
☒	☐ Antibodies
☒	☐ Eukaryotic cell lines
☒	☐ Palaeontology and archaeology
☒	☐ Animals and other organisms
☒	☐ Clinical data
☒	☐ Dual use research of concern
☒	☐ Plants

Methods

n/a	Involved in the study
☒	☐ ChIP-seq
☒	☐ Flow cytometry
☒	☐ MRI-based neuroimaging

Plants

Seed stocks	Not relevant to our study
Novel plant genotypes	Not relevant to our study
Authentication	Not relevant to our study
