# Supplementary material for: SVPG: a pangenome-based structural variant detection approach and rapid augmentation of pangenome graphs with new samples
Source: Nat Methods. 2026 Sep 21;23(10):1984–93. doi: 10.1038/s41592-026-03219-2 (PMC13645633; doi:10.1038/s41592-026-03219-2)
Supplement: Supplementary file 3 [file 41592_2026_3219_MOESM3_ESM.pdf]

nature portfolio

Peer Review File

SVPG: A pangenome-based structural variant detection approach and rapid augmentation of pangenome graphs with new samples

Corresponding Author: Professor Guohua Wang

Version 0:

Decision Letter:

17th Jul 2025

Dear Professor Jiang,

Thank you for your inquiry about submitting your manuscript, "SVPG: A pangenome-based structural variant detection approach and rapid augmentation of pangenome graphs with new samples" to Nature Methods. The paper sounds like it may be of interest, and should fit the scope of the journal. We would be willing to read and evaluate the full manuscript.

Of course, it is very difficult to judge a paper based only on the limited information available in a presubmission inquiry. Therefore I am sure you understand that we cannot promise to send your paper out for peer review and must read it in its entirety before deciding if this would be suitable.

Please keep in mind that the journal is aimed at a large, interdisciplinary audience and places a strong emphasis on the practical value of the work presented for basic research in the life sciences. We strongly encourage you to include data to validate method performance and demonstrate its general applicability.

Manuscripts describing new algorithms and software should describe the principles underlying the algorithms in an accessible style targeted for our broad biological audience. The manuscript should demonstrate the practical relevance of the tool and any advantages and limitations it presents compared to available software. The code, and ideally a user manual and example data for testing the code, must be made available to reviewers as part of the peer review process.

You will find our Guide to Authors at http://www.nature.com/naturemethods to assist you in preparing your manuscript. However, it is not necessary at this stage to spend major effort adhering to our detailed formatting instructions.

Thank you for your interest in Nature Methods.

Sincerely,

Lin Tang, PhD
Senior Editor
Nature Methods

Version 1:

Decision Letter:

29th Aug 2025

Dear Professor Jiang,

Your Article, "SVPG: A pangenome-based structural variant detection approach and rapid augmentation of pangenome graphs with new samples", has now been seen by 2 reviewers. As you will see from their comments below, although the reviewers find your work of potential interest, they have raised a number of important concerns. We continue to be interested in potentially publishing your manuscript in Nature Methods, but would like to evaluate your response to these concerns before we reach a final decision on publication.

We therefore invite you to extensively revise your manuscript to fully address all these concerns. We hope you understand that until we have read the revised manuscript we cannot promise that it will be sent back for peer review.

We are committed to providing a fair and constructive peer-review process. Do not hesitate to contact us if there are specific requests from the reviewers that you believe are technically impossible or unlikely to yield a meaningful outcome.

When revising your paper:

* include a point-by-point response to the reviewers and to any editorial suggestions

* please underline/highlight any additions to the text or areas with other significant changes to facilitate review of the revised manuscript

* address the points listed described below to conform to our open science requirements

* ensure it complies with our general format requirements as set out in our guide to authors at www.nature.com/naturemethods

* resubmit all the necessary files by using the link below to access your home page

Link Redacted

Note: This URL links to your confidential account and associated information about manuscripts you may have submitted, or that you are reviewing for us. If you wish to forward this email to co-authors, please delete this link.

We hope to receive your revised paper within 3 months. If you are substantially delayed, please let us know. In this event, we will still be happy to reconsider your paper at a later date so long as nothing similar has been accepted for publication at Nature Methods or published elsewhere.

OPEN SCIENCE REQUIREMENTS

REPORTING SUMMARY
When revising your manuscript, please update your reporting summary.

Reporting summary: https://www.nature.com/documents/nr-reporting-summary.zip

If your paper includes custom software, we also ask you to complete a supplemental reporting summary.

Software supplement: https://www.nature.com/documents/nr-software-policy.pdf

Please submit these with your revised manuscript. They will be available to reviewers to aid in their evaluation if the paper is re-reviewed. If you have any questions about the checklist, please see http://www.nature.com/authors/policies/availability.html or contact me.

Please note that these forms are dynamic 'smart pdfs' and must therefore be downloaded and completed in Adobe Reader. We will then flatten them for ease of use by the reviewers. If you would like to reference the guidance text as you complete the template, please access these flattened versions at http://www.nature.com/authors/policies/availability.html.

For any revision that includes light microscopy data, we ask our authors to please include a completed light microscopy reporting table [https://www.nature.com/documents/Light_microscopy_reporting_table.xlsx] to ensure the methods are described thoroughly. The table will be available to reviewers and ultimately published should the manuscript be accepted at the journal.

EXTENDED DATA FIGURES
When re-submitting your manuscript, please ensure that any supplementary figures and tables that are crucial to the manuscript's conclusions are converted into Extended Data figures and tables to increase visibility of these data. Extended Data figures and tables are online-only (present in the online PDF and full-text HTML versions of the paper), peer-reviewed display items that provide essential background to the article but are not included in the main article due to space constraints. A maximum of ten Extended Data display items (figures and tables) is permitted.

DATA AVAILABILITY
We strongly encourage you to deposit all new data associated with the paper in a persistent repository where they can be freely and enduringly accessed. We recommend submitting the data to discipline-specific and community-recognized repositories; a list of repositories is provided here: http://www.nature.com/sdata/policies/repositories

All novel DNA and RNA sequencing data, protein sequences, genetic polymorphisms, linked genotype and phenotype data, gene expression data, macromolecular structures, and proteomics data must be deposited in a publicly accessible database, and accession codes and associated hyperlinks must be provided in the “Data Availability” section.

For papers containing bioimaging data, we strongly recommend depositing the data to Bioimage Archive (https://www.ebi.ac.uk/bioimage-archive/). Associated accession codes and hyperlinks should be provided in the "Data Availability" section.

Refer to our data policies here: https://www.nature.com/nature-research/editorial-policies/reporting-standards#availability-of-data

To further increase transparency, we encourage you to provide, in tabular form, the data underlying the graphical representations used in your figures. This is in addition to our data-deposition policy for specific types of experiments and large datasets. For readers, the source data will be made accessible directly from the figure legend. Spreadsheets can be submitted in .xls, .xlsx or .csv formats. Only one (1) file per figure is permitted: thus if there is a multi-paneled figure the source data for each panel should be clearly labeled in the csv/Excel file; alternately the data for a figure can be included in multiple, clearly labeled sheets in an Excel file. File sizes of up to 30 MB are permitted. When submitting source data files with your manuscript please select the Source Data file type and use the Title field in the File Description tab to indicate which figure the source data pertains to.

Please include a “Data availability” subsection in the Online Methods. This section should inform readers about the availability of the data used to support the conclusions of your study, including accession codes to public repositories, references to source data that may be published alongside the paper, unique identifiers such as URLs to data repository entries, or data set DOIs, and any other statement about data availability. At a minimum, you should include the following statement: “The data that support the findings of this study are available from the corresponding author upon request”, describing which data is available upon request and mentioning any restrictions on availability. If DOIs are provided, please include these in the Reference list (authors, title, publisher (repository name), identifier, year). For more guidance on how to write this section please see: http://www.nature.com/authors/policies/data/data-availability-statements-data-citations.pdf

CODE AVAILABILITY
Please include a “Code Availability” subsection in the Online Methods which details how your custom code is made available. Only in rare cases (where code is not central to the main conclusions of the paper) is the statement “available upon request” allowed (and reasons should be specified).

We request that you deposit code in a DOI-minting repository such as Zenodo, Gigantum or Code Ocean and cite the DOI in the Reference list. We also request that you use code versioning and provide a license.

For more information on our code sharing policy and requirements, please see: https://www.nature.com/nature-research/editorial-policies/reporting-standards#availability-of-computer-code

MATERIALS AVAILABILITY
As a condition of publication in Nature Methods, authors are required to make unique materials promptly available to others without undue qualifications.

Authors reporting new chemical compounds must provide chemical structure, synthesis and characterization details. Authors reporting mutant strains and cell lines are strongly encouraged to use established public repositories.

More details about our materials availability policy can be found at https://www.nature.com/nature-portfolio/editorial-policies/reporting-standards#availability-of-materials

ORCID
Nature Methods is committed to improving transparency in authorship. As part of our efforts in this direction, we are now requesting that all authors identified as ‘corresponding author’ on published papers create and link their Open Researcher and Contributor Identifier (ORCID) with their account on the Manuscript Tracking System (MTS), prior to acceptance. This applies to primary research papers only. ORCID helps the scientific community achieve unambiguous attribution of all scholarly contributions. You can create and link your ORCID from the home page of the MTS by clicking on ‘Modify my Springer Nature account’. For more information please visit please visit www.springernature.com/orcid.

Please do not hesitate to contact me if you have any questions or would like to discuss these revisions further. We look forward to seeing your revised manuscript and thank you for the opportunity to consider your work.

Best regards,

Lin Tang, PhD
Senior Editor
Nature Methods

Reviewers' Comments:

Reviewer #1 (Remarks to the Author):

The paper describes a new tool SVPG that was developed for long-read structural variant calling using a pangenome graph. By using the population SV structure information that is encoded in the pangenome, SVPG can improve the sensitivity and accuracy of SV detection. The authors benchmark SVPG against the single-reference SV calling methods, as well as one pagenome approach and show that SVPG results in improvement in F1 scores against HG002 benchmark, simulated data as well as somatic SV calls with cancer cell lines.

Overall, the topic of pangenome-aided variant calling is exciting, and with the recent releases of high-quality references by the HPRC consortium new tools are needed to take advantage of this resource. It is expected that using the knowledge of existing SVs in a population, one can perform better variant calling of both common and rare variants. However, the current version of benchmarks described in this paper has substantial omissions and inaccuracies, and it is therefore difficult to judge if SVPG represents a practical advance.

Somatic SVs benchmarking:
1. The authors report a FN SVPG calls in the HG008 truth set, and 800+ FN calls in the HCC1395 truth set, but they argue that these calls overlap with bubbles in the pangenome graph and therefore likely represent germline variation that was erroneously included in the truth sets. However, the vast majority of these calls (from Suppl. Tables 9 and 10) seem to overlap annotated repetitive elements, such as VNTRs, that are known SV hotspots. This explains the overlap of FN SVPG calls with the pangenome bubbles - and does not really mean that these SVs are gemline, they just happened to be in hotspot regions. In fact, on all provided IGV screenshots one can clearly see that there is no SV signal in the normal reads, a clear indication of somatic SVs.

2. Benchmarking with Truvari is not optimal, putting the tools that produce BND calls at substantial disadvantage. Truvari was initially developed for germline SV comparison, predominantly small INDELs, and with default parameters performs poorly on datasets with large events and BNDs. One should adjust parameters to better capture these variants: --sizemax 100000000 (to raise the default 50 kb size limit for large calls), -S 0 (to include BNDs, as their “size” is considered 0 by truvari). Without these adjustments, truvari removes many true SVs from both benchmarking and query VCFs. Different tools may also represent the same variant with different VCF type (e.g. INS could be DUP). To improve that, --typeignore, and --dup-to-ins should be added. In addition, VCF format requires paired representations for BNDs (A -> B and B ->A), which Truvari v5.2 cannot handle; thus, VCFs must be filtered for such entries prior to benchmarking.

3. Recall/precision should be reported for all tools on the HCC1395 and COLO829 datasets, currently it is only reported for HG008.

Other benchmarking:
4. HG002 benchmarks: It is expected that pengenome-based tools will perform better than tools relying on single reference (since pangenome contains additional information). SVPG should be compared with other pangenome tools, such as SVarp and miniSV on this benchmark.

5. Rare SVs benchmark: performance of SVPG should be compared with reference-based methods and also SVarp, currently only miniSV comparison is provided.

6. What is the overall significance of the rare SV benchmark? For reference-based methods, it does not make a difference if a variant is rare or common. Is it more difficult to detect rare SVs in the pangenome mode? Perhaps, a direct evaluation of common vs rare SV performance could be helpful here.

7. Which of two modes (pangenome-guided or pangenome based) are the authors using for HG002 and somatic benchmarking? Currently it is unclear. Can both modes be used?

8. In the pangenome augmentation section, the following statement is not accurate: “Traditional pangenome augmentation typically relies on complex, time-consuming de novo assembly processes which require high-quality sequencing data and

multiple refined assembly steps.” For example, VG can augment the graph from alignment (https://github.com/vgteam/vg).

9. For the running time benchmarking, hifiasm does not take 3 days to assemble a genome (https://github.com/chhylp123/hifiasm). It seems that the authors are comparing CPU time, but not wall clock time, which is not an entirely fair comparison, and how it is currently reported in the methods is misleading.

10. All tool outputs that are used in benchmarking should be made publicly available (e.g. via a figshare or zenodo archive).

Reviewer #2 (Remarks to the Author):

Hu et al described SVPG, a new tool for long-read SV calling. It can also augment an existing minigraph graph with new SVs called from long reads. Schloissnig et al, preprinted last year and now published in Nature, use a similar approach including graph augmentation. Although the other paper provides source code, it does not aim for enduser tooling, so this paper by Hu et al still have values. In my opinion, the main advantage of SVPG is the consistent SV representation enforced by pangenome graphs (Fig. 2f). This will help multi-sample SV analysis as is shown by Schloissnig et al. Nonetheless, the authors focused on single-sample SV calling. This is a missed opportunity.

1) For the HG002 benchmark, the authors should use the more recent Q100 ground truth, which is more accurate, doubles the number of SVs and is more representative of practical SV calling.

2) Sawfish is the state of the art for HiFi SV calling and should be evaluated. The sawfish paper uses the Q100 ground truth.

3) Am I right that the pangenome-guided mode can only genotype known SVs in the graph? Either way, this should be clearly stated. Also, is it possible to combine the two modes? We will want to call both known and novel SVs for a practical sample.

4) I do think SV consistency shown in Fig. 2f demonstrates the advantage of pangenome-based SV calling. However, inconsistency does not necessarily mean SV calls are wrong. What happens is that the other callers still call correct SVs but put the same SV at different places. The discussion in this section should be revised.

5) The authors may call known and novel SVs for the 20 HPRC samples and merge SVs across samples. Then compare to the merge of sniffles2/sawfish calls. They will probably find there are fewer SVPG-based calls at higher frequency, indicating higher SV consistency.

6) While reference-based or pangenome-based methods are fine for small SVs shorter than reads, they can't resolve longer segmental duplications or satellite. Only de novo assembly can. I know the authors found cases where assembler failed but overall assembly-based analysis will be more reliable. Schloissnig et al augmented graphs because most of their samples were sequenced with Nanopore R9 and could not be assembled.

7) If I am right, SVPG is unable to call translocations and foldback inversions. If that is the case, it is not suitable for tumor SV calling in general as for most cancers, these types of SVs are more important than long INDELs.

Version 2:

Decision Letter:

27th Jan 2026

Dear Professor Jiang,

Your Article, "SVPG: A pangenome-based structural variant detection approach and rapid augmentation of pangenome graphs with new samples", has now been seen by 2 reviewers. As you will see from their comments below, Reviewer 1 still raises a number of important concerns. We continue to be interested in potentially publishing your manuscript in Nature Methods, but would like to evaluate your response to these concerns before we reach a final decision on publication.

We will consider looking at a revised manuscript only if you are able to fully address all these criticisms of the reviewers. We hope you understand that until we have read the revised manuscript we cannot promise that it will be sent back for peer review.

We are committed to providing a fair and constructive peer-review process. Do not hesitate to contact us if there are specific requests from the reviewers that you believe are technically impossible or unlikely to yield a meaningful outcome.

When revising your paper:

* include a point-by-point response to the reviewers and to any editorial suggestions

* please underline/highlight any additions to the text or areas with other significant changes to facilitate review of the revised manuscript

* address the points listed described below to conform to our open science requirements

* ensure it complies with our general format requirements as set out in our guide to authors at www.nature.com/naturemethods

* resubmit all the necessary files by using the link below to access your home page

Link Redacted

Note: This URL links to your confidential account and associated information about manuscripts you may have submitted, or that you are reviewing for us. If you wish to forward this email to co-authors, please delete this link.

We hope to receive your revised paper within 2 months. If you are substantially delayed, please let us know. In this event, we will still be happy to reconsider your paper at a later date so long as nothing similar has been accepted for publication at Nature Methods or published elsewhere.

OPEN SCIENCE REQUIREMENTS

REPORTING SUMMARY
When revising your manuscript, please update your reporting summary.

Reporting summary: https://www.nature.com/documents/nr-reporting-summary.zip

If your paper includes custom software, we also ask you to complete a supplemental reporting summary.

Software supplement: https://www.nature.com/documents/nr-software-policy.pdf

Please submit these with your revised manuscript. They will be available to reviewers to aid in their evaluation if the paper is re-reviewed. If you have any questions about the checklist, please see http://www.nature.com/authors/policies/availability.html or contact me.

Please note that these forms are dynamic ‘smart pdfs’ and must therefore be downloaded and completed in Adobe Reader. We will then flatten them for ease of use by the reviewers. If you would like to reference the guidance text as you complete the template, please access these flattened versions at http://www.nature.com/authors/policies/availability.html.

For any revision that includes light microscopy data, we ask our authors to please include a completed light microscopy reporting table [https://www.nature.com/documents/Light_microscopy_reporting_table.xlsx] to ensure the methods are described thoroughly. The table will be available to reviewers and ultimately published should the manuscript be accepted at the journal.

EXTENDED DATA FIGURES
When re-submitting your manuscript, please ensure that any supplementary figures and tables that are crucial to the manuscript’s conclusions are converted into Extended Data figures and tables to increase visibility of these data. Extended Data figures and tables are online-only (present in the online PDF and full-text HTML versions of the paper), peer-reviewed display items that provide essential background to the article but are not included in the main article due to space constraints. A maximum of ten Extended Data display items (figures and tables) is permitted.

DATA AVAILABILITY
We strongly encourage you to deposit all new data associated with the paper in a persistent repository where they can be freely and enduringly accessed. We recommend submitting the data to discipline-specific and community-recognized repositories; a list of repositories is provided here: http://www.nature.com/sdata/policies/repositories

All novel DNA and RNA sequencing data, protein sequences, genetic polymorphisms, linked genotype and phenotype data, gene expression data, macromolecular structures, and proteomics data must be deposited in a publicly accessible database, and accession codes and associated hyperlinks must be provided in the “Data Availability” section.

For papers containing bioimaging data, we strongly recommend depositing the data to Bioimage Archive (https://www.ebi.ac.uk/bioimage-archive/). Associated accession codes and hyperlinks should be provided in the "Data Availability" section.

Refer to our data policies here: https://www.nature.com/nature-research/editorial-policies/reporting-standards#availability-of-data

To further increase transparency, we encourage you to provide, in tabular form, the data underlying the graphical representations used in your figures. This is in addition to our data-deposition policy for specific types of experiments and large datasets. For readers, the source data will be made accessible directly from the figure legend. Spreadsheets can be submitted

in .xls, .xlsx or .csv formats. Only one (1) file per figure is permitted: thus if there is a multi-paneled figure the source data for each panel should be clearly labeled in the csv/Excel file; alternately the data for a figure can be included in multiple, clearly labeled sheets in an Excel file. File sizes of up to 30 MB are permitted. When submitting source data files with your manuscript please select the Source Data file type and use the Title field in the File Description tab to indicate which figure the source data pertains to.

Please include a “Data availability” subsection in the Online Methods. This section should inform readers about the availability of the data used to support the conclusions of your study, including accession codes to public repositories, references to source data that may be published alongside the paper, unique identifiers such as URLs to data repository entries, or data set DOIs, and any other statement about data availability. At a minimum, you should include the following statement: “The data that support the findings of this study are available from the corresponding author upon request”, describing which data is available upon request and mentioning any restrictions on availability. If DOIs are provided, please include these in the Reference list (authors, title, publisher (repository name), identifier, year). For more guidance on how to write this section please see: http://www.nature.com/authors/policies/data/data-availability-statements-data-citations.pdf

CODE AVAILABILITY
Please include a “Code Availability” subsection in the Online Methods which details how your custom code is made available. Only in rare cases (where code is not central to the main conclusions of the paper) is the statement “available upon request” allowed (and reasons should be specified).

We request that you deposit code in a DOI-minting repository such as Zenodo, Gigantum or Code Ocean and cite the DOI in the Reference list. We also request that you use code versioning and provide a license.

For more information on our code sharing policy and requirements, please see: https://www.nature.com/nature-research/editorial-policies/reporting-standards#availability-of-computer-code

MATERIALS AVAILABILITY
As a condition of publication in Nature Methods, authors are required to make unique materials promptly available to others without undue qualifications.

Authors reporting new chemical compounds must provide chemical structure, synthesis and characterization details. Authors reporting mutant strains and cell lines are strongly encouraged to use established public repositories.

More details about our materials availability policy can be found at https://www.nature.com/nature-portfolio/editorial-policies/reporting-standards#availability-of-materials

ORCID
Nature Methods is committed to improving transparency in authorship. As part of our efforts in this direction, we are now requesting that all authors identified as ‘corresponding author’ on published papers create and link their Open Researcher and Contributor Identifier (ORCID) with their account on the Manuscript Tracking System (MTS), prior to acceptance. This applies to primary research papers only. ORCID helps the scientific community achieve unambiguous attribution of all scholarly contributions. You can create and link your ORCID from the home page of the MTS by clicking on ‘Modify my Springer Nature account’. For more information please visit please visit www.springernature.com/orcid.

Please do not hesitate to contact me if you have any questions or would like to discuss these revisions further. We look forward to seeing your revised manuscript and thank you for the opportunity to consider your work.

Best regards,

Lin Tang, PhD
Senior Editor
Nature Methods

Reviewers' Comments:

Reviewer #1 (Remarks to the Author):

I thank the authors for their responses. The revision now addresses my original requests 3-10. However, my original concerns about the somatic variation benchmarking (number 1 and 2) remain unaddressed. I have provided my response in the attached pdf file.

Reviewer #2 (Remarks to the Author):

The authors have done a lot of additional work in this revision which is a notable improvement over the initial version. I still have a few minor comments:

1) The old SV v0.6 benchmark contains quite a few wrong SVs. The new Q100 is of higher quality overall. Instead of using tier 2 in v0.6 (line 133), the authors may subtract v0.6 Tier 1 regions from Q100 confident regions and use the remainder as harder regions.

2) "Cancer-specific somatic SV" is a confusing terminology: by definition, somatic events are cancer specific (i.e. they are not in the sample's germline); then "non-cancer-specific somatic SV" becomes more confusing. I would remove the terminologies and say (e.g. line 306): "they are likely somatic SVs overlapping with common germline SVs".

3) Relatedly, I found the response to Reviewer 1's comment 1 is weak. The authors take the paragraph starting at line 310 as an advantage of SVPG but I see this the major limitation. Some tumor types are associated with frequent STR/VNTR expansions. These real somatic events often overlap with common germline SVs and would be missed by SVPG. It is okay to have weakness, but I hope the authors can rewrite this paragraph and properly discuss the effect of somatic SVs overlapping with common germline SVs.

Version 3:

Decision Letter:

Our ref: NMETH-A61911C

19th Mar 2026

Dear Dr. Jiang,

Thank you for submitting your revised manuscript "SVPG: A pangenome-based structural variant detection approach and rapid augmentation of pangenome graphs with new samples" (NMETH-A61911C). It has now been seen by the original referees and their comments are below. The reviewers find that the paper has improved in revision, and therefore we'll be happy in principle to publish it in Nature Methods, pending minor revisions to comply with our editorial and formatting guidelines.

We are now performing detailed checks on your paper and will send you a checklist detailing our editorial and formatting requirements within two weeks or so. Please do not upload the final materials and make any revisions until you receive this additional information from us.

TRANSPARENT PEER REVIEW
Nature Methods offers a transparent peer review option for new original research manuscripts submitted from 17th February 2021. We encourage increased transparency in peer review by publishing the reviewer comments, author rebuttal letters and editorial decision letters if the authors agree. Such peer review material is made available as a supplementary peer review file. **Please state in the cover letter 'I wish to participate in transparent peer review' if you want to opt in, or 'I do not wish to participate in transparent peer review' if you don't.** Failure to state your preference will result in delays in accepting your manuscript for publication.

Please note: we allow redactions to authors' rebuttal and reviewer comments in the interest of confidentiality. If you are concerned about the release of confidential data, please let us know specifically what information you would like to have removed. Please note that we cannot incorporate redactions for any other reasons. Reviewer names will be published in the peer review files if the reviewer signed the comments to authors, or if reviewers explicitly agree to release their name. For more information, please refer to our FAQ page.

ORCID
IMPORTANT: Non-corresponding authors do not have to link their ORCIDs but are encouraged to do so. Please note that it will not be possible to add/modify ORCIDs at proof. Thus, please let your co-authors know that if they wish to have their ORCID added to the paper they must follow the procedure described in the following link prior to acceptance: https://www.springernature.com/gp/researchers/orcid/orcid-for-nature-research

Author names using non-Roman characters
Nature Portfolio journals can support presentation of author names using non-Roman characters in the HTML version of the article. If you wish to, please include author names in parentheses after the Roman-character spelling; see example online here. Currently supported scripts are: Arabic, Chinese, Cyrillic, Devanagari, Greek, Hebrew, Hangul, Japanese and Persian. You will be asked to verify the rendering is correct at proof stage.

Thank you again for your interest in Nature Methods. Please do not hesitate to contact me if you have any questions. We will be in touch again soon.

Sincerely,

Lin Tang, PhD
Senior Editor
Nature Methods

Reviewer #1 (Remarks to the Author):

I thank the authors for addressing all my requests in detail, I have no further comments.

Reviewer #1 (Remarks on code availability):

The respository appears to be in a good shape.

Reviewer #2 (Remarks to the Author):

The authors have addressed my comments.

Version 4:

Decision Letter:

6th Aug 2026

Dear Professor Wang,

I am pleased to inform you that your Article, "SVPG: A pangenome-based structural variant detection approach and rapid augmentation of pangenome graphs with new samples", has now been accepted for publication in Nature Methods. The received and accepted dates will be 22nd Jul 2025 and 6th Aug 2026. This note is intended to let you know what to expect from us over the next month or so, and to let you know where to address any further questions.

Per your request, we have updated the affiliation information of the co-author Heng Hu. Please kindly double check at the proof stage.

Over the next few weeks, your paper will be copyedited to ensure that it conforms to Nature Methods style. Once your paper is typeset, you will receive an email with a link to choose the appropriate publishing options for your paper and our Author Services team will be in touch regarding any additional information that may be required. It is extremely important that you let us know now whether you will be difficult to contact over the next month. If this is the case, we ask that you send us the contact information (email, phone and fax) of someone who will be able to check the proofs and deal with any last-minute problems.

After the grant of rights is completed, you will receive a link to your electronic proof via email with a request to make any corrections within 48 hours. If, when you receive your proof, you cannot meet this deadline, please inform us at rjsproduction@springernature.com immediately.

Authors may need to take specific actions to achieve compliance with funder and institutional open access mandates. If your research is supported by a funder that requires immediate open access (e.g. according to Plan S principles or the NIH public access policy) then you should select the gold OA route, and we will direct you to the compliant route where possible. Because authors warrant under our subscription licensing terms that they haven’t committed to licensing any version of their article under a licence inconsistent with the terms of our agreement – including the applicable embargo period – publication under the subscription model isn’t suitable for authors whose funders require no embargo.

If you have any questions about our publishing options, costs, Open Access requirements, or our legal forms, please contact ASJournals@springernature.com

If you have posted a preprint on any preprint server, please ensure that the preprint details are updated with a publication reference, including the DOI and a URL to the published version of the article on the journal website.

You may wish to make your media relations office aware of your accepted publication, in case they consider it appropriate to organize some internal or external publicity. Once your paper has been scheduled you will receive an email confirming the publication details. This is normally 3-4 working days in advance of publication. If you need additional notice of the date and time of publication, please let the production team know when you receive the proof of your article to ensure there is sufficient time to coordinate. Further information on our embargo policies can be found here:

https://www.nature.com/authors/policies/embargo.html

To assist our authors in disseminating their research to the broader community, our SharedIt initiative provides you with a unique shareable link that will allow anyone (with or without a subscription) to read the published article. Recipients of the link with a subscription will also be able to download and print the PDF.

As soon as your article is published, you will receive an automated email with your shareable link.

You can now use a single sign-on for all your accounts, view the status of all your manuscript submissions and reviews, access usage statistics for your published articles and download a record of your refereeing activity for the Nature journals.

Please note that you and any of your coauthors will be able to order reprints and single copies of the issue containing your article through Nature Portfolio's reprint website, which is located at http://www.nature.com/reprints/author-reprints.html. If there are any questions about reprints please send an email to author-reprints@nature.com and someone will assist you.

Please feel free to contact me if you have questions about any of these points. Thank you very much for publishing your paper at Nature Methods!

Best regards,

Lin Tang, PhD
Senior Editor
Nature Methods

** Visit the Springer Nature Editorial and Publishing website at www.springernature.com/editorial-and-publishing-jobs for more information about our career opportunities. If you have any questions please click here.**

Open Access This Peer Review File is licensed under a Creative Commons Attribution 4.0 International License, which permits use, sharing, adaptation, distribution and reproduction in any medium or format, as long as you give appropriate credit to the original author(s) and the source, provide a link to the Creative Commons license, and indicate if changes were made. In cases where reviewers are anonymous, credit should be given to 'Anonymous Referee' and the source.
The images or other third party material in this Peer Review File are included in the article's Creative Commons license, unless indicated otherwise in a credit line to the material. If material is not included in the article's Creative Commons license and your intended use is not permitted by statutory regulation or exceeds the permitted use, you will need to obtain permission directly from the copyright holder.
To view a copy of this license, visit https://creativecommons.org/licenses/by/4.0/

Dear reviewers,

We would like to express our sincere gratitude for your review comments. Please find the following a summary of revisions we have made to the manuscript based on your comments and suggestions. In the point-by-point response section, new items (figures/tables) added to address review questions are named with R (e.g., Figure R1, Table R1); and changes made to the revised manuscript are highlighted in red text. In the revised manuscript, any changes made are highlighted in red text. These changes have substantially improved the manuscript's arguments and conclusions. We hope you will be satisfied with our response.

Summary of revisions

	**Major analyses and discussions**	**In response to**	**Contents added to / changed in**	**Newly Added items**
1	Substantial omissions in benchmarks	Reviewer #1 – Comments 3, 4, 5, 10; Reviewer #2 – Comments 1, 2	Results; Methods; Fig. 2a; Fig. 2c-e; Fig. 3e; Fig. 4a; Supplementary Table 1; Supplementary Table 9	Fig. 2b; Fig. 3d; Supplementary Table 8
2	Inaccuracies in benchmarks	Reviewer #1 – Comments 1, 2, 9	Results; Fig. 4a; Supplementary Table 9; Supplementary Table 13	-
3	Unclear benchmark descriptions	Reviewer #1 – Comments 6, 7; Reviewer #2 – Comments 3	Results	Fig. 3g-h Supplementary Table 8
4	Inaccurate statements	Reviewer #1 – Comments 8; Reviewer #2 – Comments 4, 6	Results; Methods; Discussion	-
5	Function requires expansion	Reviewer #2 – Comments 5, 7	Results; Methods; Fig. 4a; Supplementary Table 9	Fig. 2h; Fig. 4b; Supplementary Table 10

Reviewer #1 (Remarks to the Author):

The paper describes a new tool SVPG that was developed for long-read structural variant calling using a pangenome graph. By using the population SV structure information that is encoded in the pangenome, SVPG can improve the sensitivity and accuracy of SV detection. The authors benchmark SVPG against the single-reference SV calling methods, as well as one pagenome approach and show that SVPG results in improvement in F1 scores against HG002 benchmark, simulated data as well as somatic SV calls with cancer cell lines.

Overall, the topic of pangenome-aided variant calling is exciting, and with the recent releases of high-quality references by the HPRC consortium new tools are needed to take advantage of this resource. It is expected that using the knowledge of existing SVs in a population, one can perform better variant calling of both common and rare variants. However, the current version of benchmarks described in this paper has substantial omissions and inaccuracies, and it is therefore difficult to judge if SVPG represents a practical advance.

Response

We sincerely thank the reviewer for the positive recognition of our work and for highlighting the significance of developing new pangenome-based SV calling tools in the era of high-quality HPRC references. We fully agree that rigorous and comprehensive benchmarking is crucial to demonstrate the practical advantages of SVPG.

In the revised manuscript, we have carefully addressed the reviewer's concerns regarding omissions and inaccuracies in the benchmarks by performing additional analyses, clarifying methodological details, and providing more complete comparisons across datasets and tools. We believe these revisions significantly improve the transparency, interpretability, and robustness of our benchmarking results, and we hope they adequately address the reviewer's concerns while reinforcing the utility and reliability of SVPG.

Somatic SVs benchmarking:

1. The authors report a FN SVPG calls in the HG008 truth set, and 800+ FN calls in the HCC1395 truth set, but they argue that these calls overlap with bubbles in the pangenome graph and therefore likely represent germline variation that was erroneously included in the truth sets. However, the vast majority of these calls (from Suppl. Tables 9 and 10) seem to overlap annotated repetitive elements, such as VNTRs, that are known SV hotspots. This explains the overlap of FN SVPG calls with the pangenome bubbles - and does not really mean that these SVs are gemline, they just happened to be in hotspot regions. In fact, on all provided IGV screenshots one can clearly see that there is no SV signal in the normal reads, a clear indication of somatic SVs.

Response:

We sincerely thank the reviewer for raising this important and insightful comment.

First, we would like to clarify that the somatic SVs discussed in our study specifically refer to cancer-specific SVs—that is, variants present exclusively in tumor tissues, absent from matched adjacent normal tissues, and **also not observed in any haplotype within the human pangenome reference**. Under this definition, such SVs have the potential to serve as candidate clinical biomarkers for cancer screening, classification, and prognosis.

As the reviewer mentioned, many of the somatic SVs we identified are indeed located within regions overlapping with bubbles in the pangenome graph. We agree that these regions are often associated with repetitive sequences, such as VNTRs, which are known SV hotspots. However, in our context, this overlap does not necessarily imply that these SVs represent true germline variation. Rather, our interpretation is that these variants—although occurring in repetitive regions—are more likely basic genomic attributes shared across healthy individuals, and thus not cancer-specific.

Notably, a recent study by Ayse G. Keskus et al. (PMID: 38585974) systematically benchmarked somatic SV callers on both healthy and cancer genomes. Their results showed that short-read-based SV callers (e.g., Manta, SvABA, GRIPSS) performed poorly on healthy genomes (e.g., HG002, CHM1/CHM13) due to challenges in repetitive sequence alignment, but exhibited better performance on cancer genomes (e.g., COLO829) compared to long-read-based tools (e.g., sniffles2, SVision). This finding highlights the distinct genomic distribution patterns between tumor-specific somatic SVs and germline (or mosaic) SVs found in normal genomes.

In our study, the somatic SVs filtered out tended to be enriched in repetitive genomic regions (i.e., overlapped with bubbles in the pangenome graph), further supporting the view that these variants are unrelated to cancer-specific alterations. Therefore, we believe that our filtration strategy is both reasonable and of practical significance.

In summary, we recognize that the difference in interpretation may stem from the focus of our respective perspectives: the reviewer emphasizes the inclusion of a broader spectrum of somatic SVs, whereas we are particularly concerned with distinguishing **cancer-specific somatic SVs** from germline or matched control's background variants. To avoid such ambiguities, we have revised the manuscript text to explicitly state that the somatic SVs discussed in this work refer specifically to cancer-specific events. The detailed modifications are as follows:

"SVPG enables enhanced somatic SV detection and benchmark refinement" of Results section: We assessed SVPG, miniSV, SVarp and five linear-reference-based callers using recently GIAB-released HiFi datasets from the HG008 cancer cell line and its matched control[35], as well as the widely used COLO829[36] tumor–normal pair (see Methods for benchmark details). Following the GIAB-

recommended benchmarking, the pangenome-based mode of SVPG achieved F1 scores of 86.1% on HG008 and 74.3% on COLO829, substantially outperforming other methods (Fig. 4a-b; Supplementary Table 9-10). Compared to traditional linear-reference-based callers (such as nanomonsv[37] and Severus[38]), the pangenome-based SVPG and miniSV, both exhibited significantly lower number of false positive calls. Furthermore, a closer inspection of HG008 dataset the five INDELs missed by SVPG revealed that four were already represented in the pangenome graph, suggesting that ***they are likely non-cancer-specific somatic SV events*** *(see Supplementary Note 2 for details). Overall, we attribute the improvement and low false-positive rate results to SVPG effectively leverages pangenome characteristics to enable precise inference of various types of* ***cancer-specific somatic SV****.*

To further evaluate the ability of SVPG to ***reduce non-cancer-specific somatic SV****s within existing benchmark sets, we analyzed an earlier yet widely-studied breast cancer cell line (tumor HCC_{1395} and matched normal HCC_{1395}BL, respectively) ...*

2. Benchmarking with Truvari is not optimal, putting the tools that produce BND calls at substantial disadvantage. Truvari was initially developed for germline SV comparison, predominantly small INDELs, and with default parameters performs poorly on datasets with large events and BNDs. One should adjust parameters to better capture these variants: --sizemax 100000000 (to raise the default 50 kb size limit for large calls), -S 0 (to include BNDs, as their "size" is considered 0 by truvari). Without these adjustments, truvari removes many true SVs from both benchmarking and query VCFs. Different tools may also represent the same variant with different VCF type (e.g. INS could be DUP). To improve that, --typeignore, and --dup-to-ins should be added. In addition, VCF format requires paired representations for BNDs (A -> B and B ->A), which Truvari v5.2 cannot handle; thus, VCFs must be filtered for such entries prior to benchmarking.

Response:

We thank the reviewer for raising this important concern regarding the appropriate configuration of Truvari for benchmarking somatic SVs, particularly large events and BND-type variants.

As the reviewer correctly noted, earlier versions of Truvari (prior to v5.0) indeed had limitations in evaluating BND-type and large-scale SVs, which could influence benchmarking outcomes. Our use of Truvari in this study was guided by the fact that it is the **officially recommended benchmarking tool** in the recently released GIAB somatic SV workflow for the HG008 benchmark set (see GIAB documentation: https://ftp-trace.ncbi.nlm.nih.gov/ReferenceSamples/giab/data_somatic/HG008/Liss_lab/analysis/NIST_HG008-T_somatic-stvar_DraftBenchmark_V0.3-20250220/README.md). As stated in the GIAB guidelines:

"We currently recommend using the SV comparison tool [truvari bench] (https://github.com/acenglish/truvari/wiki/bench) (v5.0+) to benchmark somatic structural variant calls against our draft benchmark. We represent all complex variants as a series of BND calls in the benchmark, including all inversions. As of v5.0, Truvari can handle BND to BND comparisons as well as convert between BNDs and tandem DUP, DEL, and INV symbolic calls so that different representations can be compared to the benchmark."

In our updated benchmark, we used **Truvari v5.3**, which incorporates key fixes for BND handling and resolves the issues highlighted by the reviewer (including the correction related to interpreting --*sizemax*=0 for FP BNDs; see discussions at https://github.com/ACEnglish/truvari/discussions/263 and https://github.com/ACEnglish/truvari/wiki/Updates). To ensure that ultra-large somatic SVs were evaluated without truncation, we **set *--sizemax* to –1** (no size limit), thereby removing the constraints imposed by the default value. In addition, following the reviewer's recommendation, we **included the parameters *--typeignore* and *--dup-to-ins*** to improve cross-format variant matching.

Regarding the requirement that BNDs be represented as paired entries (A→B and B→A), we agree that earlier Truvari versions did not fully support the comparison of paired BND structures. To avoid this limitation, we applied a preprocessing step similar to that used in the Severus (PMID: 38585974) to **filter and harmonize paired BND entries in both the query VCFs and benchmark sets** before evaluation (the custom script available at https://doi.org/10.5281/zenodo.17838112). This ensured that all callers were compared fairly and that no method was penalized due to differences in BND representation.

In summary, we fully acknowledge the reviewer's concern about the potential sensitivity of Truvari's default parameters in cancer SV benchmarking. In response, we have now incorporated the reviewer-recommended adjustments and have updated the somatic benchmarking results in the revised manuscript.

The updated benchmarking results show in Fig. 4a and Supplementary Table 9 in revised manuscript.

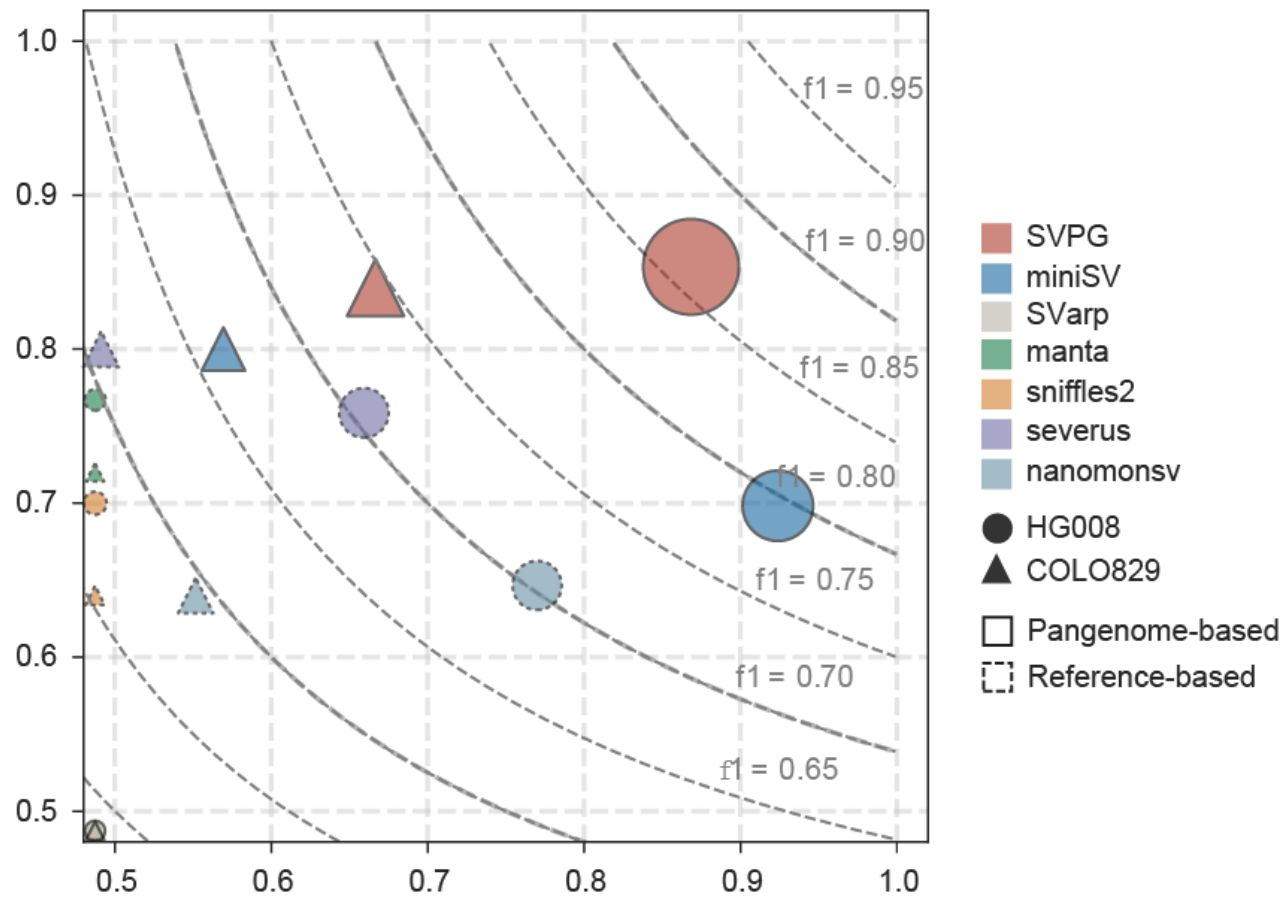

Fig.4a. Precision-Recall bubble plot of cancer-specific SVs called from HG008 and COLO829 sample. Solid lines represent pangenome-based methods and dashed lines represent linear-reference-based methods. Symbols positioned on the axis indicate the score below 0.5.

3. Recall/precision should be reported for all tools on the HCC1395 and COLO829 datasets, currently it is only reported for HG008.

Response:

We sincerely thank the reviewer for this valuable and constructive suggestion.

The HG008 dataset represents the most recent high-quality cancer genome assembly accompanied by a comprehensive and high-confidence SV benchmark set released by the GIAB. For this dataset, we reported the full set of evaluation metrics, including precision, recall, and F1-score, as it provides reliable ground truth across the entire genome.

In contrast, the benchmark sets available for the previously published HCC_{1395} and COLO829 cancer datasets were generated through limited experimental validation based on consensus SV calls. These truth sets **do not provide high-confidence SV call regions**, which makes it difficult to determine whether certain SVs should be classified as false positives. Additionally, the HCC_{1395} benchmark set also covers only part of the genome because only a selected subset of consensus SVs underwent experimental validation, and it therefore cannot fully represent the true complexity of somatic SVs in cancer genomes. As a result of these limitations, when a predicted SV does not appear in the benchmark set, it is often unclear whether this discrepancy reflects a true false positive, an event occurring within a low-confidence region, or simply a region that is not included in the truth set. **This uncertainty can lead to biased or unreliable precision estimates**.

Given these constraints, many recent studies in the field (e.g., SVision-pro and related works) have focused primarily on recall for such cancer datasets, as it more appropriately reflects the sensitivity of SV detection tools under complex tumor genomic backgrounds. Following this convention, our original manuscript mainly reported recall results for all tools on HCC_{1395} and COLO829.

In response to the reviewer's request, we **have added the complete precision and recal**l results for all evaluated tools on the HCC_{1395} and COLO829 datasets in the revised manuscript (**Supplementary Table 9**). It is important to note that, due to the inherent limitations of these benchmark sets described above, the overall low precision observed across all tools on HCC_{1395} and COLO829 should be interpreted as reflecting relative performance trends rather than absolute false-negative rates.

Other benchmarking:

4. HG002 benchmarks: It is expected that pengenome-based tools will perform better than tools relying on single reference (since pangenome contains additional information). SVPG should be compared with other pangenome tools, such as SVarp and miniSV on this benchmark.

Response:

We greatly appreciate the suggestion to include comparisons with other pangenome-based SV detection tools, such as SVarp and miniSV, on the HG002 benchmark dataset. Indeed, this was initially considered during the experimental design phase. However, several practical and methodological reasons led us to the current evaluation strategy, as detailed below.

First, the SVs in the HG002 sample are largely non-reference sequences or alleles relative to the linear reference genome (GRCh38). However, when compared against the pangenome, some of these SVs correspond to common (graph known) variants already represented within the pangenome, whereas others represent novel alleles absent from it (i.e., non-pangenome reference SVs). Our tool, SVPG, is designed to detect both categories—SVs that are already represented in the pangenome (common/known SVs) and those absent from it (rare/novel SVs). In contrast, tools such as **SVarp and miniSV are limited to detecting only non-pangenome reference (rare/novel) SVs**. Therefore, a direct comparison across all detected SVs at the HG002 **whole-genome level would be methodologically unfair**, as these tools operate on inherently different detection scopes, and we only include these tools in the benchmarks of the non-pangenome reference (rare/novel) SVs.

Second, we initially intended to include SVarp in our comparative evaluation. However, despite multiple attempts and direct communication with the original authors, **we were unable to reproduce stable results using the available implementation** (https://github.com/asylvz/SVarp/issues/9). As also noted by Reviewer #2, although SVarp provides

its source code, **it was not developed as an end-user tool**. This further highlights the current limitations of many pangenome-based SV detection frameworks in terms of usability, reproducibility, and practical deployment.

However, we believe that comparing SVPG with traditional linear-reference-based tools on the HG002 benchmark remains methodologically sound, as it highlights SVPG's performance advantage within a consistent evaluation framework. Meanwhile, **the comparison with miniSV and SVarp is more appropriately positioned within the rare/novel SV discovery context, where both tools target the same variant class.**

In response to the reviewer's request, we have **added the comparisons of SVPG with the two pangenome-based tools (miniSV and SVarp) using both the HG002-tier1 ground truth and the recently released HG002-Q100 benchmark** by GIAB (also recommended by Reviewer #2). As expected, miniSV and SVarp showed poor performance because **they only report variants outside the pangenome graph** (see Table R1 and Fig. 2a–b in the revised manuscript). To ensure a fairer evaluation, we further **stratified the GIAB-Q100 SVs using the pangenome reference**: variants not overlapping with pangenome bubbles were classified as rare SVs (n = 586), while the remaining ones were classified as common SVs (n = 27,698). We then aligned the HG002 sequencing data to the pangenome reference and performed both pangenome-based SV calling (SVPG, miniSV, and SVarp) and linear-reference-based SV calling (Sniffles2, cuteSV, SVIM, DeBreak, and Sawfish). Overall, SVPG achieved 3.0%–19.8% and 1.0%–10.8% higher F1-scores than other callers on ONT and HiFi data **from the Q100-rare SVs**, respectively (see Fig. 3d in the revised manuscript).

Table R1. Performance of miniSV and SVarp on the two GIAB ground truth

Ground truth	Caller	Platform	Precision	Recall	F1
Tier1	miniSV	HiFi	0.853211	0.019283	0.037713
	SVarp	HiFi	0.847059	0.022393	0.043632
	miniSV	ONT	0.462651	0.019905	0.038167
	SVarp	ONT	0.667488	0.028095	0.05392
Q100	miniSV	HiFi	0.789604	0.012273	0.02417
	SVarp	HiFi	0.849498	0.021621	0.04217
	miniSV	ONT	0.353695	0.014186	0.027277
	SVarp	ONT	0.613402	0.025917	0.049732

All these new analyses and results have been incorporated into the revised manuscript:

Introduction section: Recent developments such as SVarp[20] and miniSV[21] represent significant advances in de novo SV discovery from pangenome. SVarp can generate SV-related contigs through local assembly, while miniSV provides direct SV calling based on the pangenome graph.

***Both tools are limited to reporting variants outside the pangenome graph**, and their performance in diverse genomic contexts has yet to be evaluated comprehensively.*

…

"SVPG enables high performance SV calling on the GIAB ground truth set" of Results section: We first assessed pangenome-guided mode's SV calling performance on HG002 sample's Tier1_ v0.6 ground truth set[22], from the Genome in a Bottle (GIAB) consortium. We compared the SVPG with five leading long-read linear-reference-based SV callers, including Sniffles2[8], cuteSV[9], SVIM[23], DeBreak[24] and a recent published from PacBio specific HiFi SV calling tool, Sawfish[25], and with two pangenome-based callers, miniSV and SVarp. In the high-confidence SV regions, SVPG was among the top performers with F1 scores of 95.8% and 96.6% for ONT and HiFi platforms (see Fig. 2a, Supplementary Table 1 and Supplementary Fig. 1–3 for even more details).

…

Notably, miniSV and SVarp performed poorly on the HG002 benchmark set primarily because they can only detect SVs outside the pangenome. For this reason, we excluded both tools from subsequent HG002 benchmark evaluations.

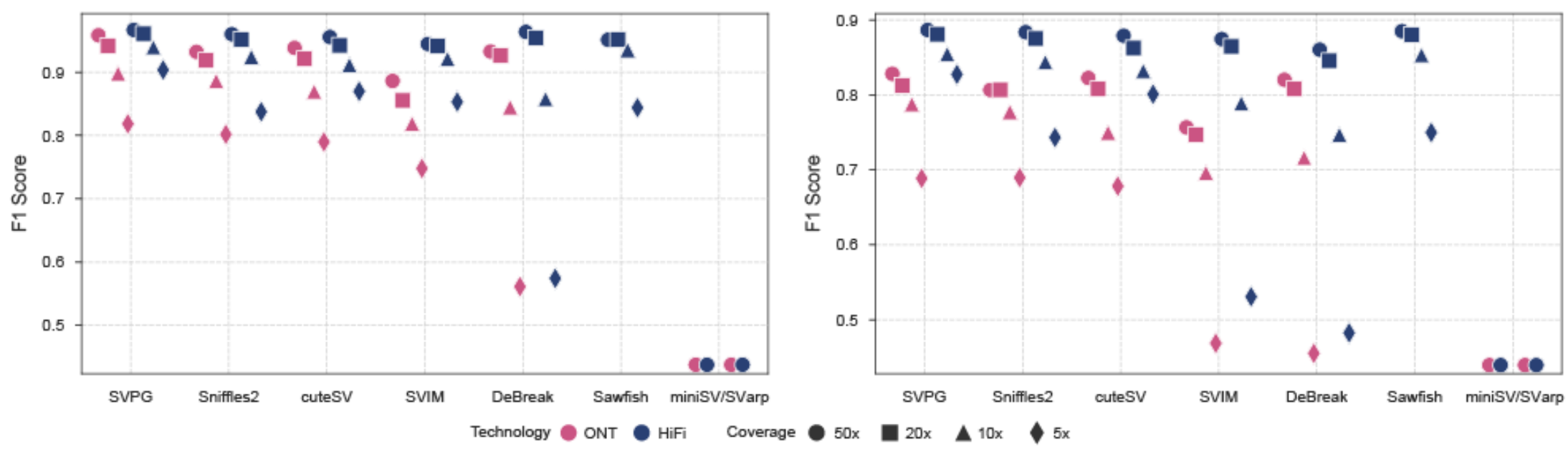

Fig. 2a. Comparison of SV calling F1 score using the GIAB-tier1 (left) and GIAB-Q100 (right) HG002 benchmark across sequencing platform (ONT and HiFi) and coverage. Symbols positioned on the x-axis indicate F1 scores below 0.5.

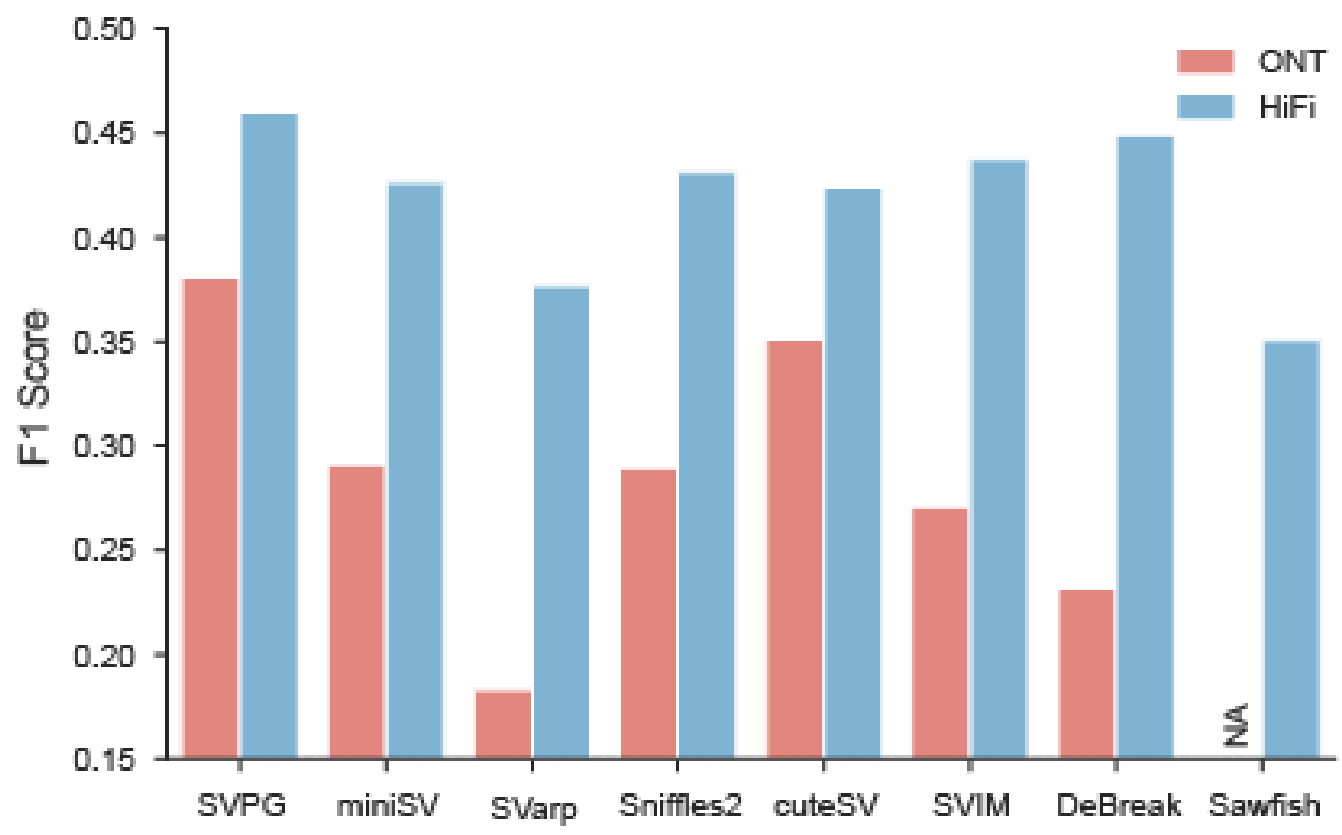

Fig.3d. Performance comparison of pangenome-based tools (top three) and linear-reference-based tools in detecting HG002-Q100 rare SVs. Sawfish is compatible only with HiFi data, and therefore was not included in the ONT platform evaluation.

5. Rare SVs benchmark: performance of SVPG should be compared with reference-based methods and also SVarp, currently only miniSV comparison is provided.

Response:

We sincerely appreciate the reviewer's insightful comment. As described in our previous response, we have expanded our evaluation on the latest HG002-Q100 benchmark by incorporating analyses of **rare SVs using both reference-based methods and SVarp** (see Fig. 3d). In these new assessments, the pangenome-based mode of SVPG achieved the best overall performance.

Notably, we initially encountered technical difficulties running SVarp (details available at [https://github.com/asylvz/SVarp/issues/9]). Following direct communication, the author recently updated the tool, enabling successful completion of the HG002 (Fig. 3d) and cancer benchmark (Fig. 4a). However, when applied to our rare SVs benchmark simulated dataset, **SVarp failed to complete the task within five days**, whereas SVPG and miniSV finished in roughly ten minutes. For this reason, we excluded SVarp from the benchmarking on simulated data. We believe this long runtime stems from the fact that, unlike normal genome data, the simulation contained a large number of rare SVs not represented in the pangenome, causing SVarp to spend excessive time performing local assemblies.

All corresponding analyses and results have been integrated into the revised manuscript:

"Pangenome-based rare SV calling performance assessment" of Results section: We input the simulated read alignments against the pangenome reference into SVPG's pangenome-based calling mode, and compared its performance with that of miniSV (SVarp was excluded because it failed to finish after five days of runtime, while SVPG and miniSV completed within several minutes).

...

We further benchmarked SVPG on HG002 to analyze the performance to detect rare SVs in real sample. Our evaluation was based on two key assumptions: (1) The existing pangenome reference already covered the most common SVs, so de novo SVs called from the pangenome graph are likely to represented individual-specific rare SVs; (2) An individual's rare SVs should be a subset of their complete SV spectrum, with benchmark-validated SVs considered as true positive. Specifically, we stratified the GIAB-Q100 SVs using the pangenome reference: variants not overlapping with pangenome bubbles were labeled as rare SV ground truth, while the remaining were labeled as common SVs. We then aligned HG002 sequencing data to the pangenome reference and performed

pangenome-based SV calling (i.e., SVPG, miniSV and SVarp) and linear-reference-based SV calling (i.e., Sniffles2, cuteSV, SVIM, DeBreak, Sawfish). Overall, SVPG achieved 3%~19.8% and 0.5~10.3% more F1 scores than other callers on ONT and HiFi data from the Q100-rare SVs (Fig. 3d), respectively. These SVs were further confirmed as genuinely rare through verification against the Human Genome Structural Variation Consortium (HGSVC) dataset of 65 individuals (Fig. 3e). Fig. 3f shows an example of a rare deletion called by SVPG, with the UCSC Genome Browser screenshot suggesting that this variant may be pathogenic.

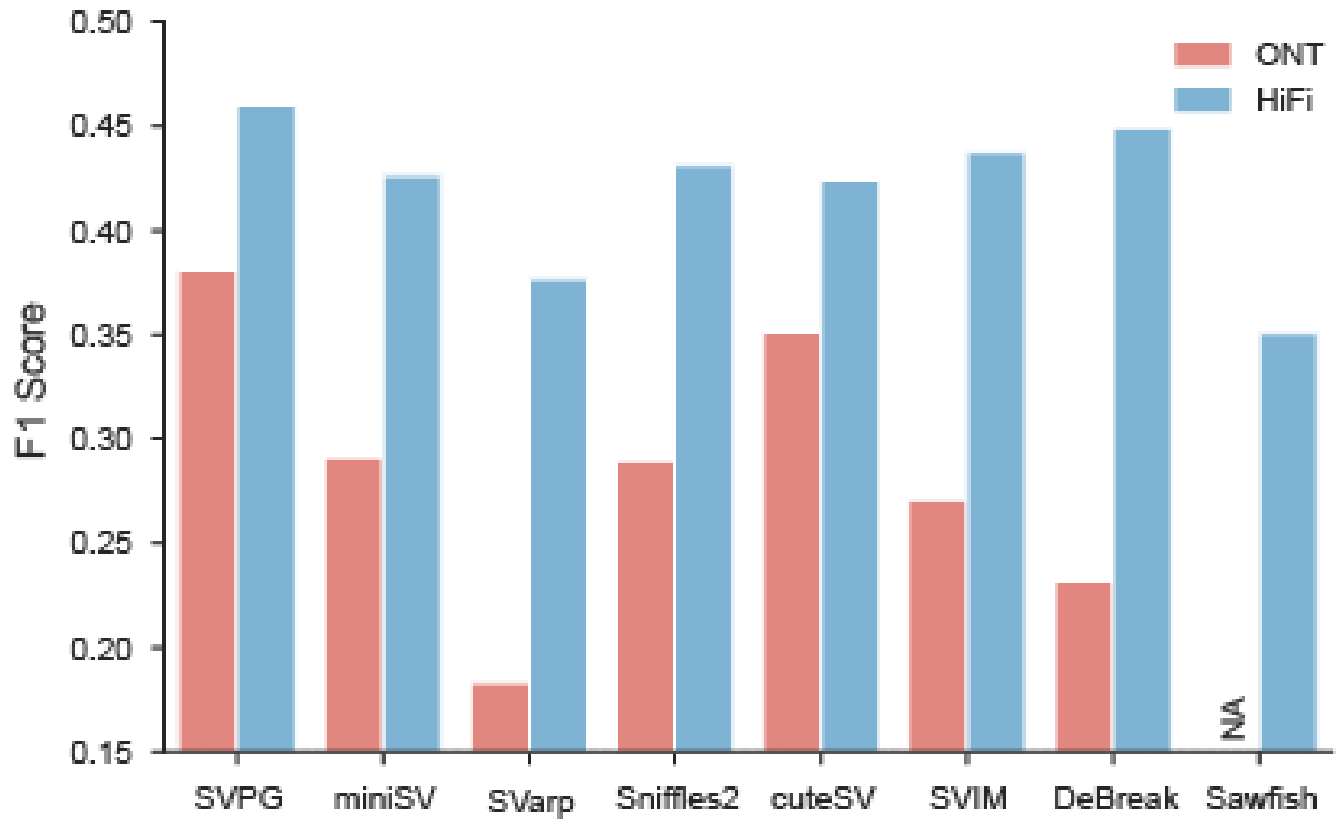

Fig.3d. Performance comparison of pangenome-based tools (top three) and linear-reference-based tools in detecting HG002-Q100 rare SVs. Sawfish is compatible only with HiFi data, and therefore was not included in the ONT platform evaluation.

6. What is the overall significance of the rare SV benchmark? For reference-based methods, it does not make a difference if a variant is rare or common. Is it more difficult to detect rare SVs in the pangenome mode? Perhaps, a direct evaluation of common vs rare SV performance could be helpful here.

Response:

We sincerely thank the reviewer for this insightful comment. The motivation for introducing a rare SV benchmark in our study is rooted in a practical and methodological distinction between pangenome-based and reference-based workflows that is often overlooked: for reference-based callers, "rare" versus "common" is chiefly a downstream annotation — variants are first discovered genome-wide against a linear reference and only subsequently classified by population frequency using cohort panels or pangenome panels; by contrast, **pangenome-based methods can expose rare, sample-specific structural differences directly at the call stage** because the graph encodes alternative haplotypes and common structural sequence that linear references do not. In cohort studies, the ability to call rare SVs directly from graph alignments removes an expensive and error-prone two-step procedure (discovery then cohort-level filtering), enabling fast, on-the-fly identification of variants that are absent from all graph haplotypes. That operational advantage is

precisely the rationale behind **SVPG's graph-augmentation** mode: by encoding common structural haplotypes directly in the graph, **it reduces reference-driven false positives and makes true sample-specific variants stand out more clearly during alignment**.

Against this background, the rare-SV benchmark we designed serves two purposes. First, it provides a **direct test of whether pangenome-based callers can robustly recover low-frequency, potentially novel events without relying on post hoc cohort subtraction**. Second, it measures **whether the structural priors encoded in a pangenome introduce bias against detecting low-frequency alleles** (for example, by over-favoring graph-encoded common paths). Conceptually, this is complementary to standard benchmarks that emphasize high-frequency or well-represented variants and therefore addresses a gap that is particularly relevant for population genomics and for studies interested in rare, possibly deleterious SVs.

We fully agree that directly comparison of performance on common versus rare SVs is informative and have therefore included such an analysis in our revision. **To address this, we have added a comprehensive evaluation using the recently released GIAB Q100 benchmark.** In this expanded analysis, we assessed all tools on the HG002 dataset.

In the updated Q100 benchmarking results (see Fig. 3g and Supplementary Table 8 in the revised manuscript) show that **rare SVs are inherently more difficult to detect** (F1 score: ~45% for rare vs. ~89% for common), not only for linear-reference–based tools but also for pangenome-based tools. Importantly, the pangenome-based mode of SVPG remains the most effective configuration in the rare SV benchmark, outperforming the linear-reference-based methods by alleviating single-reference bias and leveraging only the internal structure and variation information embedded within the pangenome graph. Moreover, while DeBreak achieved the second-best F1 score after SVPG when using PacBio HiFi reads, its performance dropped substantially on the ONT platform, limiting its broader applicability.

Overall, these results demonstrate that detecting rare SVs remains a significant challenge for all tools, but SVPG consistently achieves the best performance across sequencing platforms.

All these new analyses and results have been incorporated into the revised manuscript:

"Pangenome-based rare SV calling performance assessment" of Results section: Specifically, we stratified the GIAB-Q100 SVs using the pangenome reference: variants not overlapping with pangenome bubbles were labeled as rare SV ground truth, while the remaining were labeled as common SVs. We then aligned HG002 sequencing data to the pangenome reference and performed pangenome-based SV calling (i.e., SVPG, miniSV and SVarp) and linear-reference-based SV calling (i.e., Sniffles2, cuteSV, SVIM, DeBreak, Sawfish).

…

Moreover, we found that rare SVs are substantially more difficult to detect than common ones (Fig. 3g). Even so, the pangenome-based strategy remains highly effective: compared with the pangenome-guided mode, the full pangenome-based mode enables SVPG to detect rare SVs with markedly higher accuracy (Fig. 3h). It is owing that pangenome-based mode is more sensitive to sequences absent in the pangenome, while pangenome-guided mode inherits biases from the linear reference, limiting its ability in the fully rare SV detection.

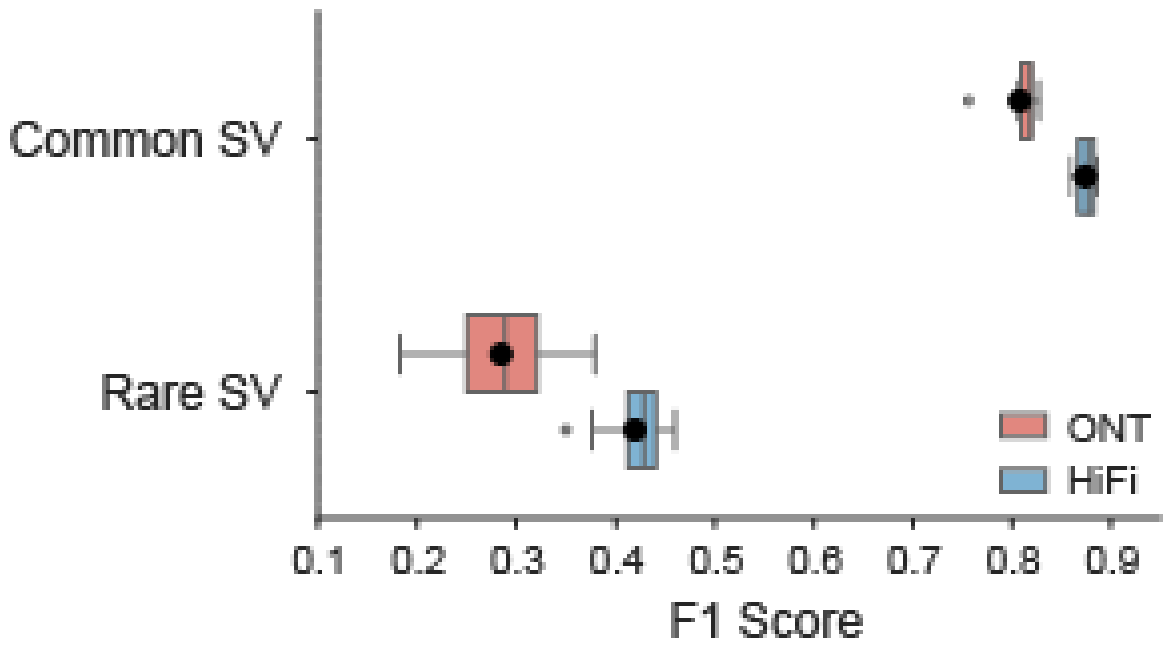

Fig.3g. Performance comparison for calling common (six tools) and rare SVs (eight tools) in the HG002-Q100 benchmark set.

7. Which of two modes (pangenome-guided or pangenome based) are the authors using for HG002 and somatic benchmarking? Currently it is unclear. Can both modes be used?

Response:

We sincerely thank the reviewer for pointing out that this section was not clearly described. In the revised manuscript, we have clarified the differences between the **pangenome-guided** and **pangenome-based** modes, as well as their specific usage in the HG002 and somatic benchmarks.

As mentioned earlier, SVs can be categorized as either common (known)—those already represented in the pangenome—or rare (novel)—those absent from it.

- For **common SVs**, we aim to leverage the pangenome to improve alignment accuracy on the single-reference. To achieve this, we designed the **pangenome-guided mode**, which performs mapping primarily against the single-reference genome but is refined using pangenome information.
- For **rare SVs**, we focus on sequences or alleles not captured by the pangenome. In such cases, direct comparison on the graph is more informative and effective, leading to the design of the **pangenome-based mode**, which eliminates single-reference bias and relies entirely on graph-based analysis.

While the pangenome-guided mode is also capable of identifying some rare SVs (detailed discussion see comment 3 of Review #2), its reliance on single-reference alignment inevitably reduces sensitivity for highly divergent sequences. For instance, as shown in Fig. 3h in the revised manuscript, the pangenome-based mode of SVPG achieved higher F1 scores for rare SV detection compared to the pangenome-guided mode (46.0% vs. 41.0% on PacBio HiFi, and 38.1% vs. 37.1% on ONT). Conversely, because the pangenome-based mode focuses solely on novel sequences, it sacrifices some ability to detect known/common SVs.

Overall, we recommend:

- **Pangenome-guided mode** — for comprehensive SV detection, especially when common SVs are of primary interest or when high sensitivity to rare SVs is not essential.
- **Pangenome-based mode** — for high-precision detection of *rare* or *somatic* SVs, where removing single-reference bias is critical.

In our benchmarking:

- For **HG002**, which includes both common and rare SVs, we primarily used the **pangenome-guided mode** to provide an overall performance evaluation. Additionally, we conducted supplementary tests using the **pangenome-based mode** to specifically assess rare SV detection.
- For **somatic benchmarks**, which focus exclusively on tumor-specific and rare SVs, we used the **pangenome-based mode**.

We have added detailed explanations and corresponding clarifications in both the main text, ensuring that readers can clearly understand the distinct purposes, advantages, and limitations of the two modes, as well as the rationale for our selection in different benchmarking scenarios.

The detailed modifications in the revised manuscript are as follows:

"Overview of SVPG" of Results section: This dual-mode design provides SVPG with broader applicability: Researchers can select the first mode conservative and highly accurate germline SV calling, capturing both common and novel variants and supporting high-precision whole-genome and population-level genotyping. In contrast, the second mode is suited for detecting sample-specific events in tumor analyses and identifies novel or rare variants that are not encoded by any haplotype in the graph.

"Pangenome-based rare SV calling performance assessment" of Results section: Moreover, we found that rare SVs are substantially more difficult to detect than common ones (Fig. 3g). Even so, the pangenome-based strategy remains highly effective: compared with the pangenome-guided mode, the full pangenome-based mode enables SVPG to detect rare SVs with markedly higher

accuracy (Fig. 3h). It is owing that pangenome-based mode is more sensitive to sequences absent in the pangenome, while pangenome-guided mode inherits biases from the linear reference, limiting its ability in the fully rare SV detection.

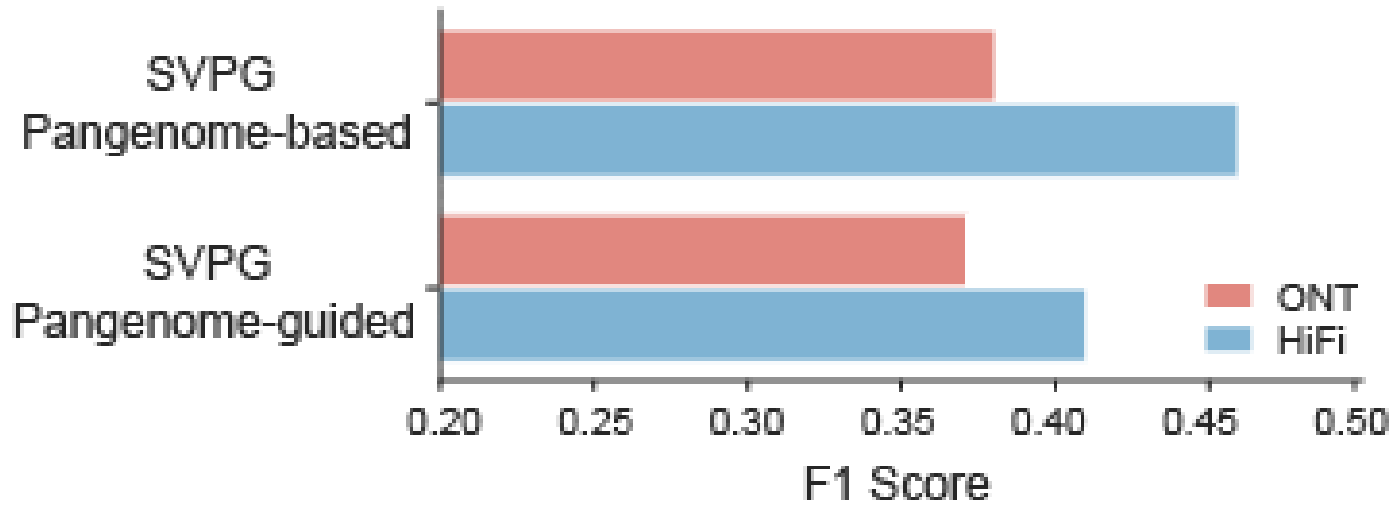

Fig.3h. Performance comparison of SVPG in pangenome-based versus pangenome-guided modes for HG002 rare SV detection

8. In the pangenome augmentation section, the following statement is not accurate: "Traditional pangenome augmentation typically relies on complex, time-consuming de novo assembly processes which require high-quality sequencing data and multiple refined assembly steps." For example, VG can augment the graph from alignment (https://github.com/vgteam/vg).

Response:

We sincerely thank the reviewer for this valuable comment. We fully agree that the VG toolkit supports graph augmentation from read alignments, as stated in its documentation: "*Variation from alignments can be embedded back into the graph.*" This means that variants detected during alignment can indeed be incorporated into the existing graph for iterative refinement and variant calling.

At the same time, we would like to clarify the specific technical context we intended to convey in our original manuscript. In the framework of large-scale pangenome projects such as those led by the human pangenome reference construction (HPRC), the mainstream and widely adopted workflow for pangenome construction and augmentation focuses on integrating high-quality *de novo* assemblies. This approach ensures that the resulting graph represents complete and accurate allele sequences, rather than primarily relying on direct incorporation of read alignments.

Moreover, regarding the practical maturity of VG's augmentation functionality, the VG documentation itself provides a clear caution:

"*Warning: Using VG augmentation for variant calling remains very experimental. It is not at all recommended for structural variant calling, and even for small variants, you will often get much more accurate results (at least on human) by projecting your alignment to BAM and running a linear variant caller such as DeepVariant.*"

Therefore, in the context of HPRC with SVs as a core focus, our original statement referred specifically to the mainstream and recommended practice—that is, augmentation through high-quality *de novo* assembly, which remains the most robust and reliable method for characterizing SVs.

To eliminate ambiguity, we have revised the relevant sentence in the manuscript to a more precise formulation:

"Rapid pangenome graph augmentation from SVPG's graph-called SVs" of Results section: Traditional pangenome augmentation, exemplified by HPRC efforts, typically relies on complex, time-consuming de novo assembly processes which require high-quality sequencing data and multiple refined assembly steps.

9. For the running time benchmarking, hifiasm does not take 3 days to assemble a genome (https://github.com/chhylp123/hifiasm). It seems that the authors are comparing CPU time, but not wall clock time, which is not an entirely fair comparison, and how it is currently reported in the methods is misleading.

Response:

We sincerely thank the reviewer for pointing out this important clarification. We would like to provide the following explanations:

1. The reported "3 days" refers to **the total running time for assembling 20 genomes** in the pangenome augmentation experiment (as shown in Supplementary Table 13), rather than the time required for a single sample. On average, **hifiasm took approximately 2.85 hours per sample**, which is consistent with the performance described in the official documentation (https://github.com/chhylp123/hifiasm).
2. All running times reported in our study represent **wall-clock time**, measured directly in a real execution environment. We acknowledge that in Supplementary Table 13, the column header was mistakenly labeled as "CPU time", which may have caused misunderstanding. **This has been corrected to "wall-clock time" in the revised version**, and the corresponding measurement approach has been explicitly clarified in the table legend.

We sincerely appreciate the reviewer's careful attention to this detail. We believe that the revised description will make our benchmarking more accurately reflect the actual computational performance.

10. All tool outputs that are used in benchmarking should be made publicly available (e.g. via a figshare or zenodo archive).

Response:

We sincerely thank the reviewer for highlighting the importance of reproducibility. To address this, we have deposited all tool outputs used in our benchmarking analyses—including VCF files, benchmarking scripts and augmented graphs into a public Zenodo repository: https://doi.org/10.5281/zenodo.17838112. Together, this archive ensures that all analyses presented in the manuscript are transparent and fully reproducible.

Reviewer #2 (Remarks to the Author):

Hu et al described SVPG, a new tool for long-read SV calling. It can also augment an existing minigraph graph with new SVs called from long reads. Schloissnig et al, preprinted last year and now published in Nature, use a similar approach including graph augmentation. Although the other paper provides source code, it does not aim for enduser tooling, so this paper by Hu et al still have values. In my opinion, the main advantage of SVPG is the consistent SV representation enforced by pangenome graphs (Fig. 2f). This will help multi-sample SV analysis as is shown by Schloissnig et al. Nonetheless, the authors focused on single-sample SV calling. This is a missed opportunity.

Response

We thank the reviewer for the clear and insightful summary of SVPG's value in achieving consistent SV representation on graph-based pangenome. SVPG was initially developed with a primary focus on single-sample SV detection, aiming to establish a robust methodological and performance baseline under single-sample conditions. We fully agree that demonstrating performance at the multi-sample or cohort level is important for readers. Although the original manuscript emphasized the application scenario of iteratively integrating new sample variants into the pangenome, it underrepresented another complementary and equally important aspect: leveraging a shared pangenome reference to enhance cross-sample calling consistency, particularly for high-frequency SVs.

In response to the reviewer's suggestion, we have added an evaluation of SVPG's performance in a multi-sample setting. In this supplementary analysis, we applied SVPG as an SV caller across a cohort and assessed its concordance and reproducibility at matched variant loci, benchmarking the results against existing cohort-level pipelines (see Response 5 for detail). The results demonstrate that the current SVPG framework is technically prepared for extension to multi-sample analysis and exhibits clear potential for improving calling consistency of high-frequency SVs at the population scale.

We would like to once again thank the reviewer's insightful comments, which have substantially contributed to refine the scope and presentation of SVPG. We have made detailed responses addressing each point and hope these revisions will adequately address the reviewer's concerns and reinforce the clarity and strength of this study. A point-to-point response to each comment is provided below.

1) For the HG002 benchmark, the authors should use the more recent Q100 ground truth, which is more accurate, doubles the number of SVs and is more representative of practical SV calling.

Response:

We sincerely thank the reviewer for bringing up this critical point. Regarding the two suggestions to evaluate SVPG using the latest HG002-Q100 ground truth and to include an assessment of sawfish, we provide a unified response and report supplementary experiments to address the reviewer's concerns.

It is important to note that, at the time of initial manuscript submission, the GIAB HG002-Q100 benchmark (https://www.biorxiv.org/content/10.1101) was still under active development and review; therefore, our early evaluations were primarily based on the more mature Tier1_v0.6 benchmark. The HG002-Q100 benchmark represents a next-generation variant reference derived from a fully gapless, whole-chromosome assembly (T2T-HG002 v1.0, QV100). Compared to the traditional Tier1_v0.6 benchmark, Q100 fills approximately 15% of previously unrepresented genomic regions, including numerous complex and repetitive loci, leading to a substantial increase in variant density and diversity, particularly within low-complexity regions (LCRs) (https://arxiv.org/html/2509.23057v1). In this revision, we adopt the Q100 benchmark as a new ground truth and re-evaluate a series of tools, including SVPG and sawfish, to provide readers and reviewers with performance comparisons under this more comprehensive benchmark.

Regarding sawfish, we note that this tool was specifically optimized by the PacBio team for the Q100 benchmark and emphasizes a local haplotype assembly strategy to achieve base-level SV detection (https://github.com/PacificBiosciences/sawfish). It is important to highlight that sawfish currently only supports PacBio HiFi data, and there is no ONT support; therefore, our evaluation of sawfish was limited to the HiFi platform.

Using the Q100 benchmark in Truvari testing, SVPG maintained the leading performance across both HiFi and ONT platforms. Specifically, SVPG achieved the highest F1 score on HiFi data (88.6%), outperforming all other tools, with sawfish ranking second (Table R2; Fig 2b and Supplementary Table 1 in the revised manuscript). On ONT data, SVPG likewise obtained the highest F1 score (82.8%). Given the known challenges of evaluating SVs in LCR-enriched regions within the Q100 benchmark, we further applied Truvari's --*refine* command (https://github.com/ACEnglish/truvari/wiki/refine) for sequence-based assessment. In this refined evaluation, SVPG achieved overall F1 scores higher than most comparative methods, ranking just below sawfish (see Table R2). We attribute sawfish's advantage in the refine scenario primarily to its built-in local assembly and precise breakpoint modeling: by generating basepair resolution variant sequences, sawfish attains higher consistency scores in sequence realignment and evaluation in high repetition and LCRs.

We further investigated the mechanisms underlying the relative challenges faced by SVPG in these regions. While pangenome graphs provide rich reference information for diverse sequences, **highly repetitive regions or LCRs often fragment into numerous short nodes, resulting in fragmented alignment paths rather than continuous haplotype mappings**. Additionally, the presence of

repeats can produce mixed representations in graph alignments (comprising both fragmented non-reference sequences and CIGAR-based local alignments), which complicates downstream SV calling. As is shown in Figure R1, minigraph often produces inconsistent alignments in certain LCR, rendering heuristic rules insufficient for identifying consistent SV breakpoints. These observations highlight the difficulty of robustly detecting continuous non-reference sequences in repetitive and LCR-enriched loci.

To address these challenges, **the latest version of SVPG introduces an optional realignment module** (*--realign*). This module collects reads from difficult regions, constructs a local consensus using abPOA, and then realigns the consensus sequence back to the reference to refine both breakpoints and variant sequences. In *--refine* tests of truvari with the Q100 benchmark, enabling *--realign* brought SVPG's performance on par with sawfish (see Table R2), demonstrating that incorporating local consensus and realignment effectively improves base-level representation of variants in LCRs. However, this strategy inevitably increases computational cost: in our tests, the baseline runtime of SVPG is approximately 20 minutes (see Supplementary Table 20), whereas enabling *--realign* adds roughly 60 minutes (totaling ~1 hour 20 minutes). This observation that substantial increase in computational time is consistent with sawfish, which relies entirely on local assembly and requires ~2.5 hours using 16 threads on the HG002 HiFi sample, far longer than SVPG with *--realign*.

In summary, SVPG maintains strong overall performance in assembly-based HG002-Q100 benchmarking. For complex regions such as LCRs, it offers an optional realignment pathway to narrow the base-level gap with local assembly approaches. We acknowledge that further optimization is possible. In particular, we are considering (1) more precise and efficient LCR identification strategies to limit unnecessary realignment, (2) tighter integration of local assembly with graph information to retain SVPG's advantages while improving breakpoint resolution, and (3) algorithmic acceleration, including parallelization and streamlined realignment procedures, to enhance base-pair resolution without substantially increasing computational burden. These optimizations are beyond the scope of the current submission but will be implemented in future releases of the framework.

Table R2. Comparison on HG002 HiFi dataset using Truvari *bench* and *refine* against the Q100 ground truth (P: precision; R: Recall)

Tools	P-bench	R-bench	F1-bench	P-refine	R-refine	F1-refine
SVPG	0.93509	0.84251	**0.88639**	0.94738	0.87341	**0.90889**
SVPG-realign	0.93440	0.85406	**0.89243**	0.94672	0.88460	**0.91461**
sniffles	0.95884	0.81902	0.88343	0.96332	0.84721	0.90154
cuteSV	0.95792	0.81162	0.87872	0.96332	0.84721	0.90154
SVIM	0.94997	0.80967	0.87422	0.96808	0.84970	0.90503
debreak	0.95465	0.78249	0.86004	0.96219	0.84414	0.89931
sawfish	0.95482	0.82436	**0.88481**	0.96285	0.87188	**0.91511**

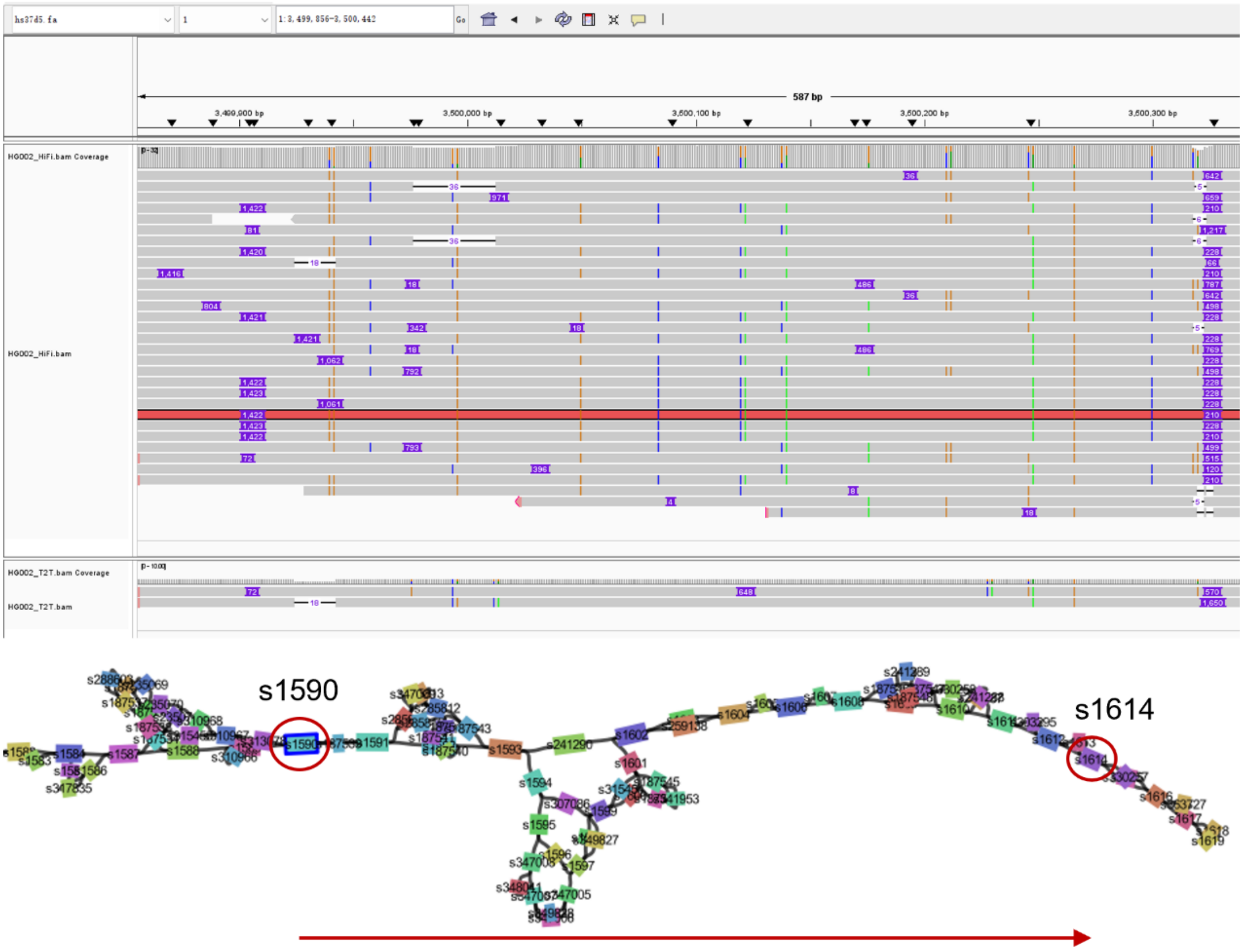

Figure R1. The Q100 benchmark reports three insertions in the region chr1:3,500,140–3,500,326, with lengths of 1,045 bp, 229 bp, and 1,669 bp (as shown at the bottom of the IGV panel). However, the bottom panel of Bandage indicates that the pangenome graph in this region is highly fragmented, comprising numerous short local nodes. Aligning HiFi read (the read as marked in IGV) from this region using minigraph produces the following node chain: >s1590>s187539>s1591<s285814 <s285813<s285812>s187543>s1593>s1594>s1595>s1596>s1597>s1598 >s1599 >s1600>s187544>s187545>s1601>s1602>s1603>s1604>s1605>s1606>s1607>s1608>s187546<s241289>s187547<s330258<s241287>s1611>s1612>s1614. This alignment path traverses multiple non-reference

nodes and repeatedly changes orientation, reflecting highly noisy signals in complex repetitive regions and fail to produce a consistent local structural representation.

All these new analyses and results have been incorporated into the revised manuscript:

*"SVPG enables high performance SV calling on the GIAB ground truth set" of results section: We compared the SVPG with five leading long-read linear-reference-based SV callers, including Sniffles2[8], cuteSV[9], SVIM[23], DeBreak[24] and a **recent published from PacBio specific HiFi SV calling tool, Sawfish**[25], and with two pangenome-based callers, miniSV and SVarp. In the high-confidence SV regions, SVPG was among the top performers with F1 scores of 95.8% and 96.6% for ONT and HiFi platforms (see Fig. 2a, Supplementary Table 1 and Supplementary Fig. 1–3 for even more details). In subsequent downsampling datasets, SVPG maintained F1 scores above 90.0% even at low-coverage conditions (ONT-10× and HiFi-5× coverage), consistently demonstrating superior detection robustness.*

***We also evaluated all tools using the newly released GIAB HG002-Q100 benchmark set**, which provides a next-generation variant reference derived from a fully gapless, telomere-to-telomere genome assembly. On HiFi data, SVPG delivered a leading F1 score of 88.6%, performing on par with Sawfish and outperforming all other methods (Fig. 2b). In contrast, all tools exhibited a marked drop in performance on ONT data, likely reflecting the substantial number of newly added low-complexity regions in the Q100 benchmark, which pose challenges for technologies with higher base-error rates. Despite this, SVPG achieved the highest F1 score at 82.8%. Analyses of downsampled datasets from both platforms further support above observations. Notably, miniSV and SVarp performed poorly on the HG002 benchmark set primarily because they can only detect SVs outside the pangenome. For this reason, we excluded both tools from subsequent HG002 benchmark evaluations.*

2) Sawfish is the state of the art for HiFi SV calling and should be evaluated. The sawfish paper uses the Q100 ground truth.

Response:

We once again thank the reviewer for this constructive comment. Given the strong relevance of this suggestion to the previous comment, we have incorporated a comprehensive explanation and analysis on the HG002-Q100 benchmark into the response. Thus, it will not be reiterated here.

Additionally, in the revised manuscript we have included **sawfish for comparison** in the original HG002 benchmarking, including the challenging-region benchmark, Mendelian discordance

evaluation, and replicate experiments; details are provided in Fig. 2a-d (see Supplementary Table 1-6 for detail) and the *Results* section in the revised manuscript.

3) Am I right that the pangenome-guided mode can only genotype known SVs in the graph? Either way, this should be clearly stated. Also, is it possible to combine the two modes? We will want to call both known and novel SVs for a practical sample.

Response:

We sincerely thank the reviewer for bringing up this critical point. In the revised manuscript, we have clarified the design rationale of the two modes, their mechanistic differences in handling known (graph-encoded/common) versus novel SVs, and how both known and novel SVs can be simultaneously captured in real samples.

First, we clarify that the pangenome-guided mode is not a simple genotyping of SVs already encoded in the graph. Although the core purpose of this mode is to leverage SV information embedded in the pangenome graph to reduce reference bias and reinforce breakpoint consistency, it can also detect SVs that are not fully represented by graph nodes (e.g., from CIGARs and local node offsets in graph alignments). The pangenome-guided mode enhances SV calling precision via **"constraint-guided genotyping"** based on graph paths: for common SVs already represented in the graph, this mode effectively corrects noise and false positives often associated with linear alignment; for potential novel SVs derived from the pangenome, it employs a read-level consistency filter based on BAM evidence. Those with consistent support are reported as novel SVs, rather than being ignored. Therefore, **this mode emphasizes conservative, highly accurate calling of germline SVs (common + novel SVs)**, making it suitable for scenarios requiring high-precision whole-genome genotyping (e.g., accurate detection of population-common variants).

In contrast, the pangenome-based mode is specifically designed to discover novel SVs directly from the pangenome graph. **It does not rely on linear reference alignments** and fully parses graph alignments file (GAF) to identify structural differences. As such, this mode is effective for **detecting sample-specific SVs in tumor analyses and can capture novel/rare SVs not encoded by any haplotype in the graph** in population-level analyses. It also facilitates rapid updates or augmentation of the pangenome.

From a **methodological perspective (see *Methods* section for detail)**, the two modes differ in handling **novel SVs**: in pangenome-guided mode, novel structural differences observed **in reference-nodes** (composed solely of reference nodes in the graph) are reported as novel SVs only if there is consistent support in the BAM file. Structural differences **aligned to non-reference nodes** (representing non-reference haplotypes) are mapped to corresponding common SVs based on SV

type and reported using unified reference coordinates, avoiding over-splitting, or false positive inflation caused by repetitive structures. In contrast, pangenome-based mode records any structural gap observed in the graph alignment as a novel SV, regardless of whether it originates from reference-node or non-reference-node paths. Because it lacks BAM-based consistency filtering, graph-based calling relies more heavily on internal heuristic filters to control noise.

We also fully agree with the reviewer regarding the importance of capturing both known and novel SVs for a practical sample. As described above, **the pangenome-guided mode inherently allows simultaneous reporting of common SVs and novel SVs** (with BAM-consistent support) in a single run. To facilitate clear distinction between these two categories, we have **introduced a new *PAN_KNOWN* flag annotation field** in the VCF output of the pangenome-guided mode in the latest SVPG release, which identifies the variant's status relative to the pangenome graph. Researchers can filter variants based on this field to distinguish between known/common variants and novel/rare SVs by bcftools.

The detailed modifications in the revised manuscript are as follows:

"Overview of SVPG" of Results section: SVPG integrates two SV detection modes: pangenome-guided and pangenome-based SV detection (Fig. 1). ***In the pangenome-guided mode****, SVPG first converts collected SV signals from a BAM file into signature reads and realigns these reads to the pangenome reference. By thoroughly analyzing the topological features and path transition patterns of SV signature reads in the pangenome graph, SVPG achieves high-precision SV breakpoint localization while optimizing the quality of original feature signals.* ***In the pangenome-based mode****, SVPG directly analyzes the alignment characteristics and patterns of raw read sequences within the pangenome graph structure without relying on the linear reference genome, accurately detecting de novo SVs within haplotype paths of graph (see Methods for details).* ***This dual-mode design provides SVPG with broader applicability****: Researchers can select the first mode conservative and highly accurate germline SV calling, capturing both common and novel variants and supporting high-precision whole-genome and population-level genotyping. In contrast, the second mode is suited for detecting sample-specific events in tumor analyses and identifies novel or rare variants that are not encoded by any haplotype in the graph.*

…

Discussion section: Unlike traditional the current long-read SV calling methods that rely on linear reference, SVPG leverages pangenome-guided priors to ***force-call and annotate widespread common SVs while retaining high sensitivity for sample-specific events****. We showed that SVPG consistently outperforms existing state-of-the-art methods, and both unbiased replicate experiments and cross-sample analyses further highlighted its substantially enhanced call consistency in the population scale and markedly reduced false negatives.*

…

Methods: Additionally, gaps in path CIGAR strings further localize SV breakpoints based on path-start nodes and alignment-start coordinates in the path, ***thereby capturing variants that are not explicitly encoded in the graph****.*

4) I do think SV consistency shown in Fig. 2f demonstrates the advantage of pangenome-based SV calling. However, inconsistency does not necessarily mean SV calls are wrong. What happens is that the other callers still call correct SVs but put the same SV at different places. The discussion in this section should be revised.

Response:

We sincerely thank the reviewer for bringing up this critical point. We fully concur with the reviewer's point that "inconsistency does not necessarily equate to error.": in regions characterized by repetitive sequences, microhomology, or complex breakpoints, different SV callers may place the same genuine variant at slightly different positions (i.e., breakpoint shifts), which can manifest as "inconsistency" during evaluation.

In fact, we recognized and accounted for this potential issue in our original manuscript. Consequently, we did not employ a strict "exact-match" criterion for our consistency evaluation. Specifically, to distinguish legitimate "breakpoint shifts/representation differences" from "technical false positives," we adopted two **tolerance criteria** inspired by Truvari:

1. **Breakpoint Tolerance:** We consider two candidate variants as potentially the same event if their breakpoint distance is ≤ 500 bp, thereby allowing for minor positional shifts.
2. **Size Concordance:** Given that the breakpoint distance criterion is met, we only classify the variants as consistent if the ratio of their lengths (min_len / max_len) is ≥ 0.7 (indicating size similarity).

These criteria aim to strike a balance: preventing the misclassification of genuine variants with minor breakpoint shifts as false positives, while maintaining sensitivity to actual single-sample artifacts arising from technical noise or library/sequencing discrepancies.

To ensure reproducibility, we have made the scripts used for this inconsistency assessment available at https://doi.org/10.5281/zenodo.17838112. We have also updated the *Methods* section of the revised manuscript to include a detailed explanation of this logic and the default thresholds used. Furthermore, we have revised the related text to avoid simplistically equating "inconsistency" with "error".

The detailed modifications in the revised manuscript are as follows:

Methods section: It is important to note that inconsistency does not always imply incorrect SV calls. Different callers may report the same true SV with slightly shifted breakpoints or alternative representations, especially in repetitive or microhomology regions. To reduce the risk of misclassifying such cases as false positives, we allowed a breakpoint tolerance of 500 bp and required a size concordance ratio (min/max) of at least 0.7 when comparing SVs across replicates. Only when both conditions were met did we classify events as consistent, otherwise as inconsistent.

5) The authors may call known and novel SVs for the 20 HPRC samples and merge SVs across samples. Then compare to the merge of sniffles2/sawfish calls. They will probably find there are fewer SVPG-based calls at higher frequency, indicating higher SV consistency.

Response:

We sincerely thank the reviewer for the insightful and constructive suggestions. Following this proposal, **we conducted an additional analysis on 20 HPRC samples to examine whether SVPG tends to produce highly consistent and more recurrent SV calls at the population scale**. Specifically, all samples were aligned to the GRCh38 reference for SV calling using SVPG and sniffles2. For sawfish, we utilized pbmm2 alignments, as the tool strictly requires pbmm2 result as input. Subsequently, we merged the call results across the 20 samples using the merging tool SURVIVOR with a 500 bp distance threshold to obtain SV sets with varying levels of "SUPP" support (i.e., the number of occurrences across samples). The "SUPP" value serves as a direct measure of SV recurrence across samples and acts as a good approximation for allele frequency (AF), thereby reflecting the recurrence and stability of an SV within the population.

Given the differences in total SV call counts driven by the distinct false-positive filtering strategies and thresholds among across tools, we adopted the *SUPP-call proportion* (calculated as *SUPP_call_count / total_call_count*) for normalization. This allows for a more objective comparison of relative proportions of SV calls across different support levels. As shown in Figure R2 (Fig. 2h in the revised manuscript), as SUPP increases (indicating the SV is consistently detected in more samples), the *SUPP-call proportion* for SVPG rises steadily and consistently exceeds that of sniffles2 and sawfish. Notably, at SUPP $\geq$ 7 (corresponding to AF $\geq$ 7/20), the *SUPP-call proportion* for SVPG is significantly higher than that of the other tools. This indicates that **SVPG tends to retain SVs that appear consistently across the sample cohort and possess strong recurrence**, rather than rare or unstable calls driven by single-sample noise. This demonstrates that the structural priors provided by the pangenome effectively enhance the stability and reproducibility of SV calling across the multi-sample dimension.

To ensure these results were not biased by parameter selection or merging strategy, we performed independent merging experiments using SURVIVOR with a 1000 bp threshold and the tool Jasmine.

Both strategies yielded highly consistent trends and conclusions, further excluding the possibility of merging-induced bias.

We further assessed the Hardy–Weinberg equilibrium (HWE) of the 20 HPRC samples, the results revealed that SVPG produced the highest proportion of loci (98.1%; HWE test scores >= 0.01 were considered pass) conforming to expected allele-frequency distributions. This closer agreement with the theoretical HWE model highlights SVPG's improved genotyping consistency and reduced bias.

In summary, these new population-scale analyses fully support the reviewer's judgment: **SVPG is more inclined to output stable, high-frequency SV calls present across multiple samples, thereby improving consistency and credibility at the population level**. These findings not only reinforce our understanding of SVPG's methodological advantages but also further illustrate the significant value of the pangenome framework in enhancing the cross-sample reproducibility of SV calling.

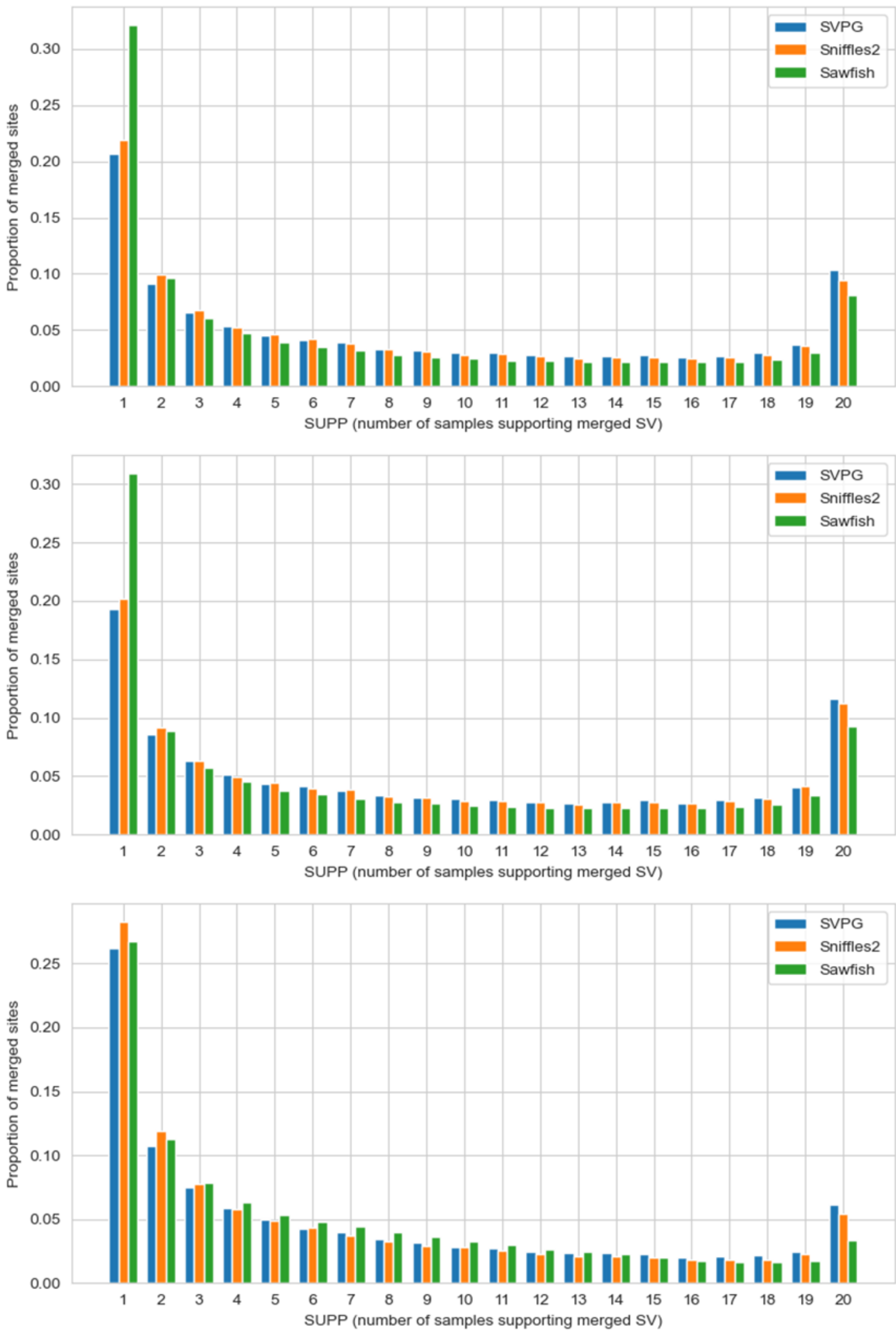

Figure R2. Multi-sample merging results of SVPG, sniffles2, and sawfish across 20 HPRC samples. The x-axis represents the sample support count (SUPP) of the merged SVs, and the y-axis shows the proportion of SVs at each SUPP level relative to the total number of calls produced by each caller. From top to bottom, the panels correspond to merging performed with SURVIVOR at 500 bp and 1000 bp thresholds, followed by Jasmine using its default parameters.

All these new analyses and results have been incorporated into the revised manuscript:

"Benchmarking SV call consistency using replicate experiments and cross sample analyses" of Results section: To validate SVPG's cross-sample consistency at the population level, ***we next performed SVPG calling on each of the 20 HPRC samples and merged the results****, alongside parallel workflows using Sniffles2 and Sawfish. We used the number of supporting samples per merged site as a proxy for population frequency and quantified the proportion of SVs falling into different support intervals for each method. SVPG exhibited a markedly higher fraction of high-support (high-frequency) variants (Fig. 2h), indicating superior sensitivity to recurrent, reliable SVs shared across individuals. Consistently, Hardy–Weinberg equilibrium (HWE) analysis further revealed that SVPG produced the highest proportion of loci (98.1%; HWE test scores >= 0.01 were considered pass) conforming to expected allele-frequency distributions. This closer agreement with the theoretical HWE model highlights SVPG's improved genotyping consistency and reduced bias. Overall, these results highlight SVPG's advantage in population-scale analyses: leveraging the pangenome framework, SVPG enforces unified representations of the same SV across samples, thereby suppressing sample-specific noise and enhancing cross-sample consistency and comparability.*

Discussion section: Unlike traditional the current long-read SV calling methods that rely on linear reference, SVPG leverages pangenome-guided priors to ***force-call and annotate widespread common SVs while retaining high sensitivity for sample-specific events****. We showed that SVPG consistently outperforms existing state-of-the-art methods, and both unbiased replicate experiments and cross-sample analyses further highlighted its substantially enhanced call consistency in the population scale and markedly reduced false negatives.*

Methods section: HPRC has released raw sequencing data for over 300 samples. From this dataset, we selected 20 samples that were not included in the draft pangenome reference. The HiFi raw reads were aligned to the GRCh38 reference genome using minimap2 and then processed with SVPG in pangenome-guided mode and Sniffles for SV calling; for Sawfish, we utilized pbmm2 alignments to meet the tool's specific requirements. SV call results across the 20 samples were subsequently merged using Jasmine[48] . For each merged site, the number of supporting samples was extracted from the "SUPP" field of the merged VCF, and the proportion of calls for each "SUPP" value (SUPP_call_count / total_call_count) was calculated. As for the proportion of HWE pass rate, it was calculated using the fill-tags module in bcftools.

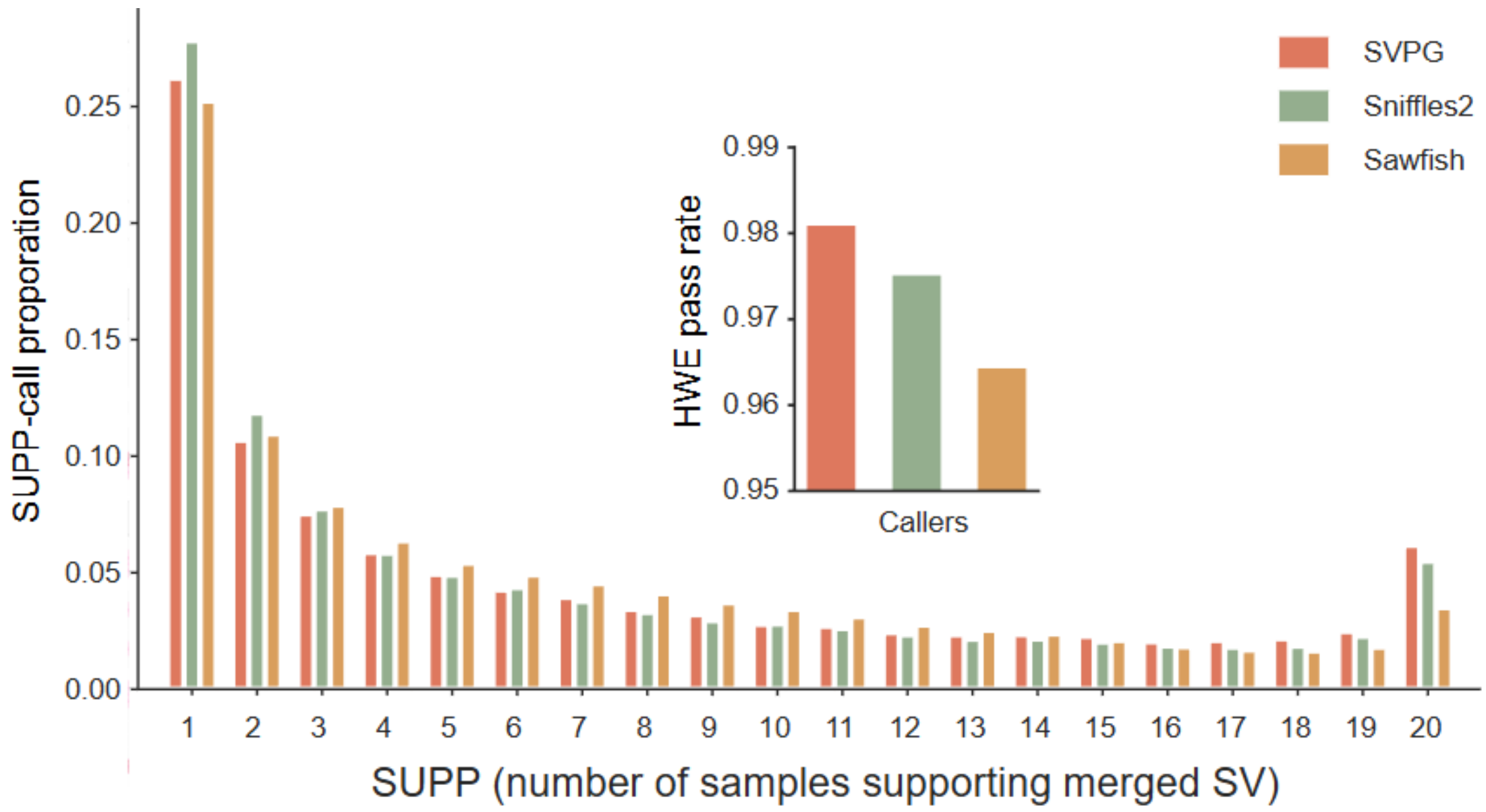

Fig. 2h. Multi-sample merging results of SVPG, Sniffles2, and Sawfish across 20 HPRC samples. The x-axis represents the sample support count (SUPP) of the merged SV loci, and the y-axis shows the proportion of SVs at each SUPP level relative to the total number of calls produced by each caller. The inset figure shows comparison of the proportion of SVs that pass the Hardy–Weinberg equilibrium (HWE) test of different tools. The SVs with HWE test scores >= 0.01 were considered pass the test.

6) While reference-based or pangenome-based methods are fine for small SVs shorter than reads, they can't resolve longer segmental duplications or satellite. Only de novo assembly can. I know the authors found cases where assembler failed but overall assembly-based analysis will be more reliable. Schloissnig et al augmented graphs because most of their samples were sequenced with Nanopore R9 and could not be assembled.

Response:

We sincerely thank the reviewer for highlighting this critical issue. We fully agree that the reviewer's perspective that ***de novo* assembly possesses superior capabilities** in resolving segmental duplications (SDs) and satellite sequences. As the reviewer noted, the decision by Schloissnig et al. to adopt a graph-augmentation strategy when dealing with data predominantly from early Nanopore R9 chemistry—due to assembly challenges—underscores the critical impact of **data quality and platform limitations** on the feasibility of successful assembly. When high-coverage, high-fidelity long reads are available (such as PacBio HiFi or ultra-long ONT combined with robust polishing), modern assemblers including hifiasm and Verkko can indeed reconstruct complex repeat structures and multi-allelic architectures, even within centromeres and other challenging genomic contexts, often providing more direct and reliable evidence than alignment-based methods.

At the same time, we acknowledge that although SVPG utilizes the pangenome reference, it shares similarities with traditional reference-based methods and **faces challenges regarding alignment accuracy in complex regions**. Previous work by Heng Li et al. (PMID: 33066802) has shown that multi-allelic VNTRs pose particular challenges for current graph construction frameworks, because different structural haplotypes may differ by only a few base pairs, preventing an optimal graph representation. We envision that future versions of SVPG could incorporate more advanced graph-building and graph-alignment engines to mitigate these limitations.

In practice, we recommend using high-quality *de novo* assemblies whenever feasible to resolve SDs, satellites, and other highly repetitive regions. However, in scenarios where generating stable and high-quality assemblies is not practical, such as projects with limited coverage, single-platform data, or large population cohorts (as exemplified by Schloissnig et al. and the American All of Us Research Program)—SVPG offers a cost-effective and scalable alternative approach. In such contexts, SVPG can complement partial assemblies or targeted validation when needed, providing a more economical solution for population-scale pangenome analyses.

In the revised manuscript, we have softened and clarified earlier statements that may have sounded overly absolute regarding "assembly failures." We now provide technical context and quantitative evidence for these limitations, and we have replaced the previous wording with a more balanced discussion. **A new explanatory text has been added to the *Discussion*** section, ensuring that reviewers and readers can clearly understand the technical background underlying these assembly challenges.

The detailed modifications are as follows:

Discussion section: Our exploration of SVPG application in pangenome graph augmentation also yielded promising results. Using graph SVs called from 20 human samples, we implemented a graph-alignment-based augmentation strategy that integrates SVPG-called graph SVs directly into existing pangenomes. The resulting augmented graphs showed high concordance with those generated from assembly–based approaches, while reducing the computational time required for graph construction by nearly ten-fold across 20 samples, and this efficiency is expected to increase further with larger sample sizes. Notably, while we present several assembly-failure cases, these examples are based on preliminary native assemblies, we acknowledge that with sufficiently accurate and high-coverage reads, together with appropriate post-processing (e.g., trio-binning, polishing, scaffolding), de novo assembly can resolve most complex regions, particularly longer segmental duplications or satellite. Nevertheless, when data quality or cost constraints limit assembly success, SVPG provides a cost-effective and scalable alternative for augmenting pangenome graphs, offering substantial practical advantages for large-scale, population-level analyses.

7) If I am right, SVPG is unable to call translocations and foldback inversions. If that is the case, it is not suitable for tumor SV calling in general as for most cancers, these types of SVs are more important than long INDELs.

Response:

We sincerely thank the reviewer for raising this important point. As correctly noted, earlier versions of SVPG were limited in their ability to detect complex SVs such as translocations and foldback inversions. To address this limitation, we have incorporated **support for graph split alignment in the latest release of SVPG, enabling the calling of three types of complex SVs: translocations, foldback inversions, and tandem duplications**. Specifically, events categorized as inversions and translocations are uniformly represented using the *SVTYPE=BND*, while a dedicated *EVENT_TYPE* field is added to further distinguish translocations, simple inversions, and foldback inversions.

We first benchmarked SVPG using the newly GIAB-released HG008 dataset and the widely used COLO829 dataset, following the official recommended Truvari tool and commands (https://ftp-trace.ncbi.nlm.nih.gov/ReferenceSamples/giab/data_somatic). The results demonstrate that SVPG achieved the highest F1 score of 86.1% on HG008 and 72.6% on COLO829, substantially outperforming other methods (Fig. 4a and Supplementary Table 9 in the revised manuscript). Notably, **graph-based methods continued to outperform traditional linear-reference-based approaches in terms of precision**, fully consistent with the INDELs evaluation in the original submission. These results further support the potential of pangenome frameworks for enhancing the refinement of cancer benchmarking datasets.

Currently, Truvari reports only overall SV performance and does not provide per–SV-type evaluation. In addition, both the somatic benchmark sets and the callsets may represent complex events using breakpoint-level annotations rather than canonical SVTYPE labels (GRIDSS has a more in-depth explanation for their decision to do this here: https://github.com/PapenfussLab/gridss#why-are-all-calls-bnd), making it difficult to distinguish inversions from translocations solely from SVTYPE. To overcome these limitations, we developed a custom evaluation script (available on https://doi.org/10.5281/zenodo.17838112) that extends Truvari's logic to more accurately assess SVs of different complexity. SVs were categorized into two classes: (i) INDEL-type events, including insertions (tandem duplications treated as insertions) and deletions, and (ii) BND-type events, including inversions, translocations. For INDELs, both breakpoint positions and variant lengths were used for matching; for BND-type events, evaluation was based on breakpoint distance and orientation. These refinements allow a more faithful assessment of complex SV detection performance. To additionally validate our evaluation script, we used Truvari to perform the same benchmarking on HG008 and COLO829,

and the overall F1 scores were highly correlated (see Figure R3). As shown in Fig. 4b and Supplementary Table 10 in the revised manuscript, SVPG achieves superior precision while maintaining high recall for complex events such as inversions and translocations.

We also conducted additional evaluations on the previously published HCC1395 cancer datasets. As noted in our response to Reviewer #1 (comment 3), the dataset didn’t provide defined high-confidence regions and not fully experimental validated. Therefore, recall was treated as the primary evaluation metric. Under Truvari, SVPG achieved the highest recall and ranked second in F1 score. Under our custom evaluation framework, SVPG showed a similar performance.

It is important to clarify that SVPG is not specifically designed for a tumor SV caller; this work represents our first attempt to apply a pangenome-based strategy to cancer genome. Accurately capturing inversion polarity and the complex topological structure surrounding repetitive regions generally requires high-fidelity graph alignment signals, highlighting the need for improved graph-based methods specifically tailored to complex tumor SVs. As emphasized in the revised manuscript, SVPG maintains high recall while offering substantial potential for **panel filtering of germline and population SVs** (particularly INDELs). This enables SVPG to serve as an effective layer for validation and refinement in cancer benchmarking studies, substantially reducing experimental validation costs and improving the reliability of variant interpretation. For ultra-complex somatic events, complementary identification via specialized somatic callers remains essential. In future work, we aim to develop new methods within the pangenome alignment framework to enhance the detection of complex tumor SVs and expand SVPG’s applicability in cancer genomics.

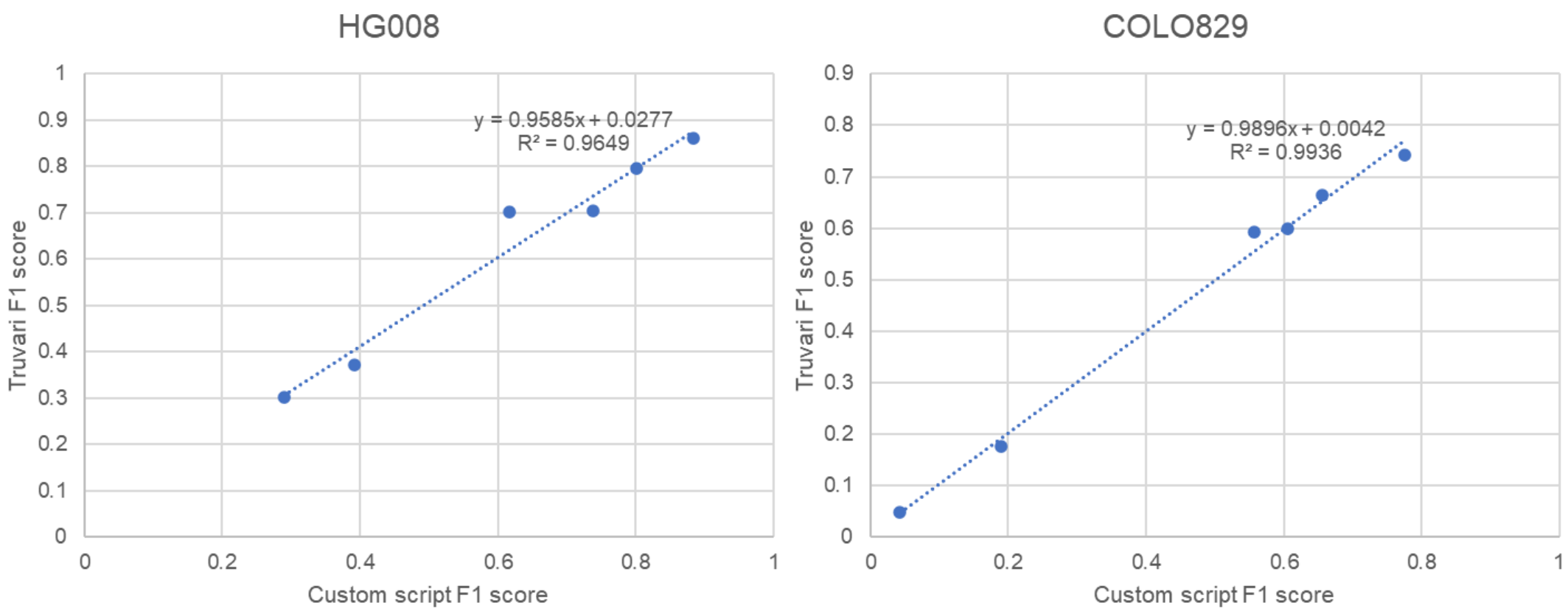

Figure R3. Consistency between the Truvari and custom benchmarking script on HG008 and COLO829 dataset. The corresponding Pearson correlation coefficients are shown.

All these new analyses and results have been incorporated into the revised manuscript:

“SVPG enables enhanced somatic SV detection and benchmark refinement” of Result section: We assessed SVPG, miniSV, SVarp and five linear-reference-based callers using recently GIAB-released

HiFi datasets from the HG008 cancer cell line and its matched control[35], as well as the widely used COLO829[36] tumor–normal pair (see Methods for benchmark details). Following the GIAB-recommended benchmarking, the pangenome-based mode of SVPG achieved F1 scores of 86.1% on HG008 and 74.3% on COLO829, substantially outperforming other methods (Fig. 4a-b; Supplementary Table 9-10). Compared to traditional linear-reference-based callers (such as nanomonsv[37] and Severus[38]), the pangenome-based SVPG and miniSV, both exhibited significantly lower number of false positive calls. Furthermore, a closer inspection of HG008 dataset the five INDELs missed by SVPG revealed that four were already represented in the pangenome graph, suggesting that they are likely non-cancer-specific somatic SV events (see Supplementary Note 2 for details). Overall, we attribute the improvement and low false-positive rate results to SVPG effectively leverages pangenome characteristics to enable precise inference of various types of cancer-specific somatic SV.

Methods section: Given the lack of explicit supplementary alignment markers in the GAF format compared to BAM format, SVPG sorts multiple alignment records from the same read by genomic position, designating the upstream alignment as primary, and determines SV breakpoint coordinates through primary-supplementary alignment relationships. Subsequently, SVPG infers the SV types (include larger INDELs, inversions, duplications, and translocations) based on the orientation and clipping location of the two segments involved.

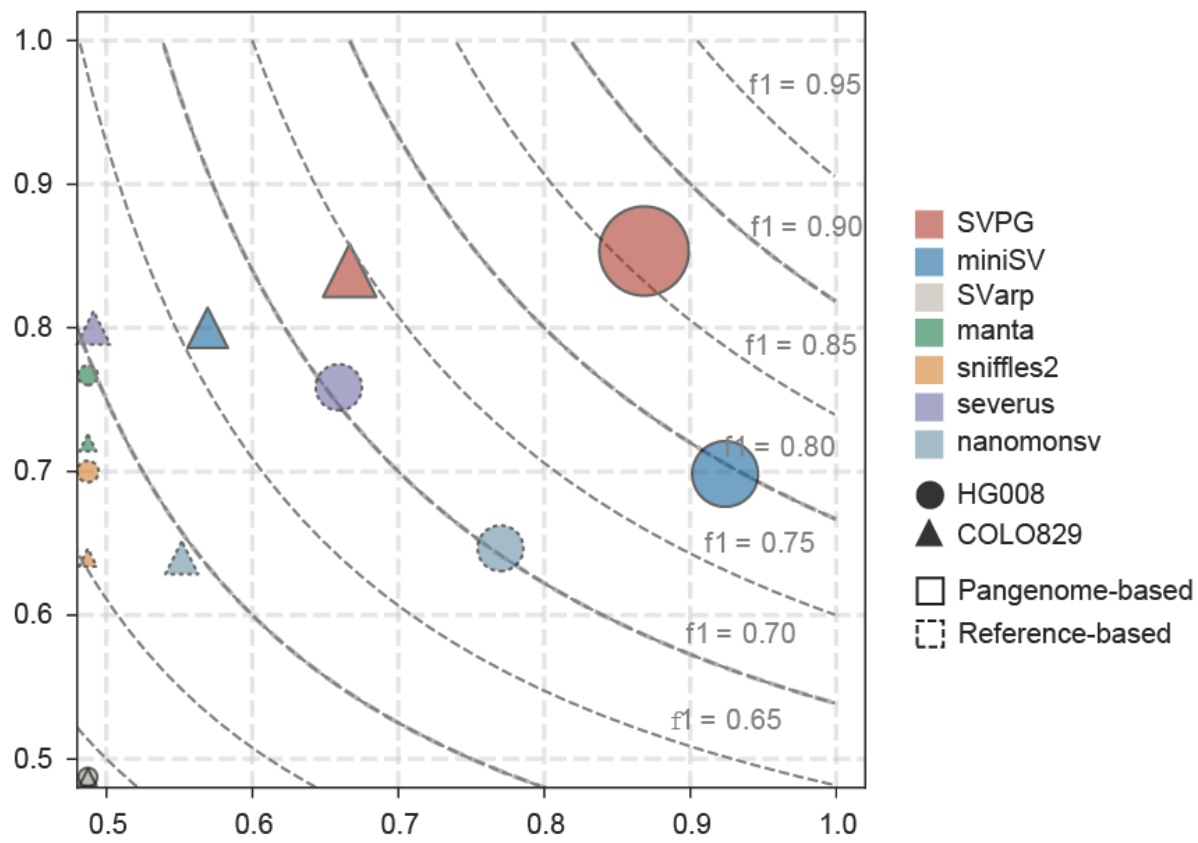

Fig.4a. Precision-Recall bubble plot of cancer-specific SVs called from HG008 and COLO829 sample. Solid lines represent pangenome-based methods and dashed lines represent linear-reference-based methods. Symbols positioned on the axis indicate the score below 0.5.

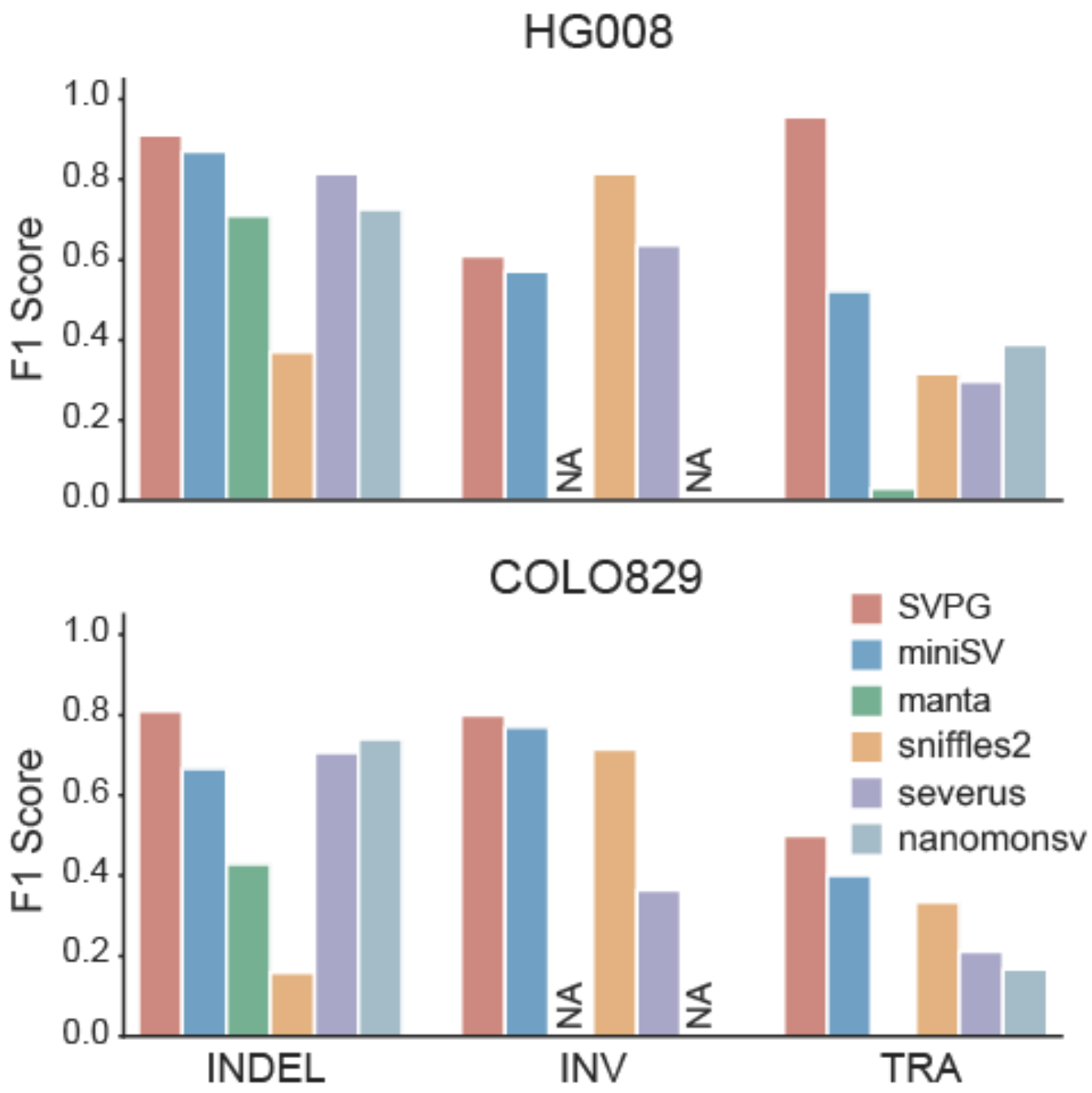

Fig. 4b. F1 score comparison across SV types. Duplications are treated as insertions and included within the INDEL category.

Dear reviewers,

We would like to express our sincere gratitude for your review comments. Please find the following a summary of revisions we have made to the manuscript based on your comments and suggestions. In the point-by-point response section, new items (figures/tables) added to address review questions are named with R (e.g., Figure R1, Table R1); and changes made to the revised manuscript are highlighted in red text. In the revised manuscript, any changes made are highlighted in red text. These changes have substantially improved the manuscript's arguments and conclusions. We hope you will be satisfied with our response.

Summary of revisions

	**Major analyses and discussions**	**In response to**	**Contents added to / changed in**	**Newly Added items**
1	Inaccuracies in somatic benchmarks	Reviewer #1 – Comments 2; Reviewer #2 – Comments 2,3	Results; Methods; Fig. 4b-e; Supplementary Table 9-10	Fig. 4b, Fig 4e; Supplementary Table 9
2	Unclear descriptions in somatic benchmark	Reviewer #1 – Comments 2,3	Results; Methods; Supplementary Table 9-10	-
3	Improvements to GIAB benchmarking	Reviewer #2 – Comments 1	Results; Methods; Supplementary Table 2-3	Supplementary Table 2

Reviewer #1 (Remarks to the Author):

I thank the authors for their responses. The revision now addresses my original requests 3-10. However, my original concerns about the somatic variation benchmarking (number 1 and 2) remain unaddressed.

Specifically, for the first point, the author's request does not address the possibility of having multiple independent alleles in highly polymorphic regions (e.g. VNTRs). The current evaluation framework matches any two non-reference alleles: for example a somatic repeat expansion in a tumor sample A is matched against an *independent* non-reference allele in an unrelated sample B from the pangenome. This leads to a missing somatic call by SVPG, and such calls seem to be highly prevalent in the HCC1395 benchmark. Furthermore, the authors count somatic calls made by other tools in VNTR regions as false positives without sufficient evidence.

Secondly, the authors have now updated the benchmarking approach with truvari and provided the output of tools used in evaluations. However, as we attempted to reproduce HG008 and COLO829 benchmarks using the author provided instructions, but were not able to do so.

Please see more details about those specific points below. Overall, given that the key somatic benchmark presentation remains problematic, I don't see sufficient evidence for a claim that SVPG improves somatic SV calling, as compared to the current methods.

Response

We thank the reviewer for the careful follow-up and for acknowledging that points 3–10 have now been addressed. We also appreciate the reviewer's continued emphasis on the somatic SV benchmarking issues (points 1 and 2), which we agree are central to a fair interpretation of the results.

Regarding the first concern, we acknowledge that our original evaluation framework did not sufficiently account for the presence of multiple independent non-reference alleles in highly polymorphic regions, such as VNTRs. As the reviewer correctly points out, in such regions a true somatic repeat expansion in the tumor sample may be spuriously matched to an independent non-reference allele observed in an unrelated pangenome sample, leading to an apparent missing somatic call by SVPG. We agree that this phenomenon is likely prevalent in the HCC1395 consensus benchmark set, and reflects a fundamental limitation of allele-agnostic matching strategies in highly repetitive regions, rather than a failure specific to SVPG.

In response, our revised benchmarking of the HCC1395 sample now focuses primarily on recall measured against biologically validated benchmark sets (Fig .4e and Supplementary Table 10). For the 953 SVs present in the consensus benchmark but not reported by SVPG, we performed a separate analysis and found that the vast majority of these SVs were likewise not detected by other tools

specifically designed for somatic SV calling (**see specific point 1 for details**). We explicitly state that these regions represent a known limitation of current somatic SV evaluation frameworks, where distinguishing true somatic events from overlapping common germline variation remains challenging. Importantly, we no longer interpret somatic calls in highly polymorphic regions as false positives in the absence of orthogonal supporting evidence.

More broadly, we emphasize that this issue highlights the need for stratified somatic SV benchmarking, consistent with prior work from the GIAB (PMID: 39424793) and pangenome-based analyses by Heng Li (PMID: 40905373), which have shown that unstratified consensus benchmarks can lead to misleading performance interpretations in highly polymorphic or uncertain genomic regions. We have revised the Results section accordingly to avoid over-claiming improvements in these regions and to clearly delineate the scope in which our benchmarking conclusions are valid.

Regarding the second concern on reproducibility, we acknowledge that, although we updated the benchmarking pipeline using Truvari and provided tool outputs, reproduction of the HG008 and COLO829 benchmarks by external users was hindered by insufficient documentation and availability of preprocessed benchmark inputs. Upon re-examination, we found that certain preprocessing steps applied to the benchmark datasets, as well as the specific benchmark versions used, were not fully made publicly available, which led to discrepancies between reproduced results and those reported in the manuscript. To address this issue, we have therefore released the exact preprocessed benchmark files and updated the Zenodo repository (https://zenodo.org/records/18456502) to enable faithful reproduction of our results.

Finally, we agree with both reviewers that, without these clarifications, the original presentation of the somatic benchmarking could give an overly strong impression of improved performance. We have therefore revised the manuscript to adopt a more conservative interpretation, explicitly limiting our claims to genomic regions where the benchmarking assumptions are valid, and clearly stating the remaining challenges in highly polymorphic regions.

Detailed, point-by-point responses to the reviewer's specific comments are provided below.

Specific points:

1 - In their response, the authors provided a clarification, but did not change their evaluation approach, which remains problematic. To reiterate, in the "SVPG enables enhanced somatic SV detection and benchmark refinement" section, the authors report 935 SVs in HCC1395 datasets that were called by 2+ other methods, but not SVPG. These are potential FNs for SVPG. However, the authors claim that 839 of these are not truly somatic SVs because they overlap with bubbles in the pangenome graph. I originally pointed out that the vast majority of these SVs overlap with annotated VNTR regions, which are highly polymorphic. Therefore - it is not surprising to see bubbles in the

pangenome graph in the same regions, as other genomes may have other unrelated polymorphisms in the same regions. and it does *not* prove that SVs uncalled by PGSV are germline.

The authors provided the following response: “We agree that these regions are often associated with repetitive sequences, such as VNTRs, which are known SV hotspots. [...] our interpretation is that these variants—although occurring in repetitive regions—are more likely basic genomic attributes shared across healthy individuals, and thus not cancer-specific.”

According to this statement, the authors consider any non-reference allele inside highly polymorphic regions as likely-germline. However, it is well known that somatic repeat expansions and contractions are common in cancer. Thus, labeling repetitive regions as “likely-germline” is (i) not ideal for the original tool, and (ii) not fair for evaluation of other methods.

Furthermore, IGV screenshots from supplementary note 2 with SVPG-missed SVs in HG008, clearly show somatic, not germline SVs, as there is clear breakpoint signal present in tumor, and absent in normal reads.

Although the authors originally answered my question by giving an alternative definition of “somatic variant”, this is highly misleading. The authors should either provide convincing proof that the 839 SVPG SVs in question should not be considered FNs, or if no such proof is provided, these variants should be clearly acknowledged as FNs.

Response:

We thank the reviewer for raising this important and well-considered concern. We fully agree that, in highly polymorphic regions such as VNTRs, **the mere presence of non-reference alleles or bubbles in a pangenome graph is insufficient to infer that an SV is germline**. Somatic repeat expansions and contractions are well documented in cancer and may occur in regions that also harbor common germline SVs. In the original manuscript, such overlaps were interpreted as indicative of shared polymorphisms, an assumption that was overly simplistic and may have led to an overestimation of SVPG’s apparent performance. In particular, pangenome overlap alone cannot reliably distinguish somatic from germline SVs in VNTR-rich contexts in the absence of biological or orthogonal validation.

To better understand the nature of the 951 SVs (updated using Truvari *--dup-to-ins* and *--typeignore*) present in the HCC1395 consensus benchmark but not reported by SVPG, we revised our analysis rather than relying on pangenome overlap alone. Specifically, we compared these SVs against call sets generated by two of the most widely used state-of-the-art **somatic SV callers**, **nanomonsv (v0.7.2) and Severus (v1.5)**, which employ substantially different detection strategies. Using Truvari v5.3 (*truvari -b fn.vcf.gz -c call.vcf.gz*), we found that the vast majority of these SVs were

also missed by nanomonsv (**949/951**) and Severus (**899/951**), respectively, suggesting that these events are intrinsically difficult to detect rather than being specific failures of SVPG.

We further note that, as discussed in the Severus study (PMID: 40185952), the HCC1395 (PMID: 36514120) consensus benchmark was constructed using earlier sequencing protocols and relied heavily on SV calls generated by traditional, largely **germline-oriented callers**, including Sniffles1, PBSV, and NanoSV. In the absence of biological or orthogonal experimental validation, consensus cancer SV benchmarks constructed primarily from germline SV callers are **inherently prone to introducing bias in highly polymorphic regions.** This observation further underscores the urgent need for higher-quality, stratified cancer SV benchmarks.

In additionally, SVPG is designed to prioritize the detection of SVs that are more likely to be cancer-specific and biologically relevant, by favoring pangenome graph paths that are not supported by common population variation. While this design may inevitably sacrifice sensitivity in highly repetitive regions enriched for STRs and VNTRs, it improves specificity and robustness across most high-confidence genomic contexts (Supplementary Tables 9 and 10), which is particularly desirable in precision oncology settings.

In summary, while SVPG's conservative design may miss some somatic SVs in highly polymorphic regions, our additional analyses indicate that the majority of these consensus benchmark candidate false negatives are not recovered by other advanced somatic SV callers and are likely affected by ambiguity or errors in the consensus benchmark itself. We have revised the evaluation and manuscript accordingly to avoiding the classification of pangenome-overlapping SVs as non-somatic or non–cancer-specific. The detailed modifications are as follows:

"Pangenome-based somatic SV calling performance assessment" of Results section: We additionally analyzed an earlier yet widely-studied HCC1395 breast cancer cell line, and its matched normal HCC1395BL. This benchmark was established through the biological validation of a curated subset of SVs derived from consensus germline callsets. Because the dataset lacks rigorously defined high-confidence genomic regions necessary for an accurate assessment of false positives, we prioritized recall as the primary metric for our performance evaluation. As shown Fig. 4e, SVPG achieved the highest sensitivity, identifying 155 out of 179 validated SVs (Supplementary Tables 10), followed closely by the pangenome-based method miniSV. We further performed the same evaluation on the consensus benchmark set comprising 1,268 SVs identified by at least two germline SV callers. We found that 951 SVs were not reported by SVPG. Notably, the majority of these SVs were also missed by nanomonsv (949) and Severus (899), indicating that these events may be broadly challenging for current somatic SV callers. Among the 951 SVs not detected by SVPG, cross-validation using bedtools intersect showed that 829 overlap with node regions in the GRCh38-90c.r518-based pangenome reference, indicating that these hard-to-call somatic SVs occur in regions enriched for common germline SV (Supplementary Table 11). In contrast, only 25 of the

285 SVs detected by SVPG overlap with pangenome nodes, which is significantly lower than the overlap observed in the unrecalled group (Fisher's exact test, p < 0.001). These regions expose a fundamental limitation of current somatic SV evaluation frameworks, where independent somatic events may overlap common germline variation in highly polymorphic regions. This ambiguity affects SVPG as well as other state-of-the-art callers, underscoring the need for more refined benchmarking strategies that explicitly account for overlapping germline and somatic variation.

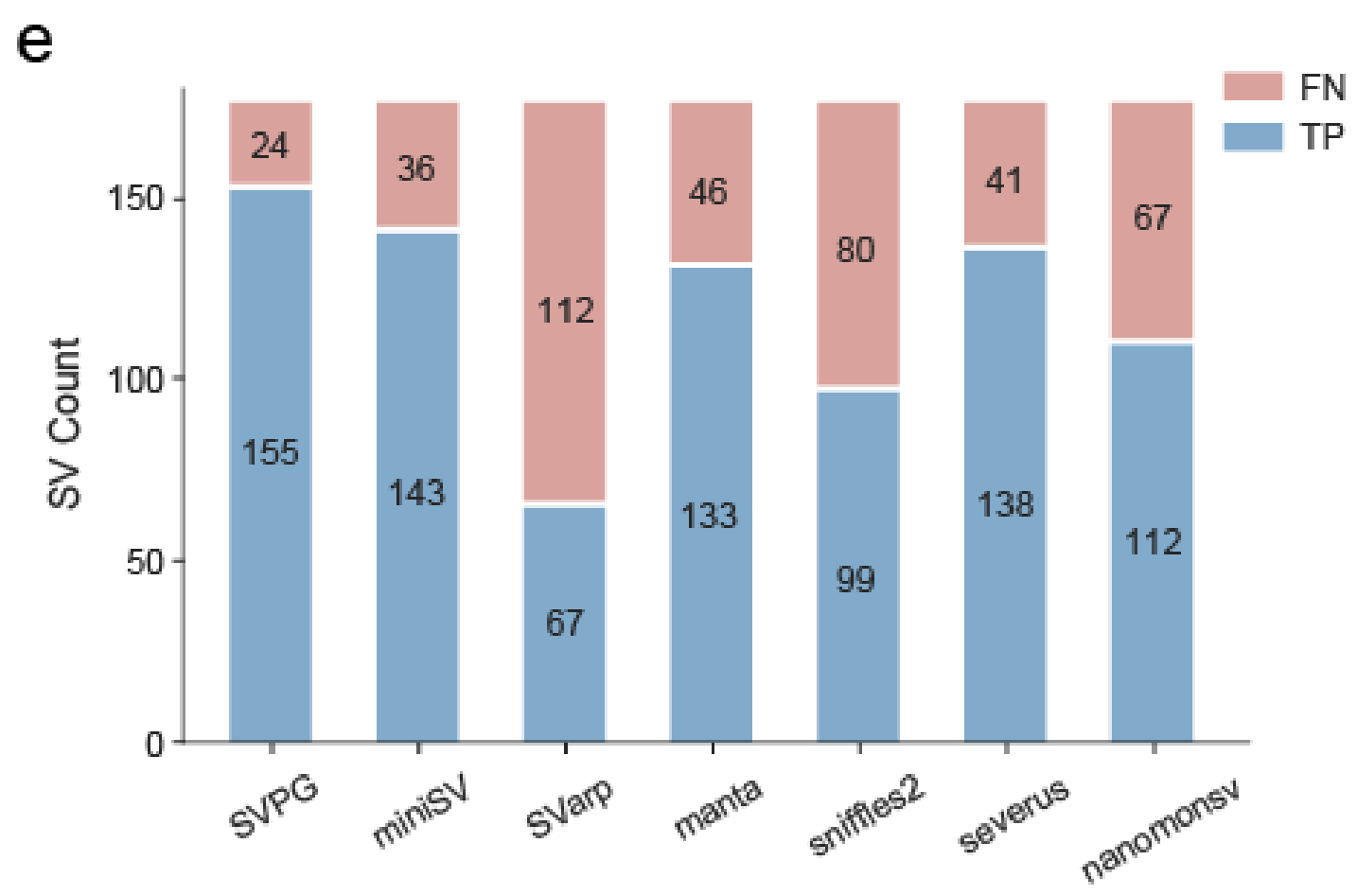

Fig.4e True positive (TP) and false positive (FP) counts of somatic SV calling on the HCC1395 biologically validated benchmark.

2 - In the revised version, the authors have provided the postprocessed outputs of all SV calling tools that were used for key evaluations. We attempted to reproduce the benchmarking statistics for the HG008 and COLO829 datasets. However, none of our strategies returned the numbers that are reported in supplementary tables 8 and 9. This needs to be clarified, and authors need to make sure that the reported results are accurate and reproducible.

Specifically, we performed the following experiments:

1. Downloaded filtered vcf files from COLO829 and HG008 runs from the zenodo archive

2. Downloaded HG008 vcf and bed file from GIAB website (GRCh38_HG008-T-V0.3_somatic-stvar_PASS.draftbenchmark) and COLO829 truthset from Valle-Inclan et al., 2022 (truthset_somaticSVs_COLO829_hg38lifted)

3. Generated single-BND representation of benchmarking vcf using the code provided in truvari_preprocess.py from the zenodo archive.

4. Ran truvari 5.3 using the parameters that the authors provided in their response (truvari bench -b "$VCF" --pick multi --sizemin 50 --sizefilt 50 --sizemax -1 --passonly -p 0.00 -o "truvari_filt/${SAMPLE}" -c "${SAMPLE}") [--includebed $BED]

5. In addition we tested the author's evaluation script (python eval_somatic.py $REF_VCF *.filter.vcf $BED >eval_somatic_filt.tsv)

6. We also ran minda (described in Keskus et al., 2025; commit 182fbcf) as an alternative benchmarking tool (minda.py truthset --base $REF_VCF --vcfs *filter.vcf.gz --out_dir minda_out --multimatch --bed $BED)

Response:

We sincerely thank the reviewer for carefully attempting to reproduce our benchmarking results and for pointing out this important reproducibility concern. We apologize for any confusion caused during the reproduction of the somatic benchmarking results.

We would like to first clarify that, based on the context and the issue raised by the reviewer, the reference to Supplementary Tables 8 and 9 appears to correspond to Supplementary Tables 9 and 10 in the revised version of the manuscript. After thorough re-examination, we confirm that the differences arise from **mismatches in benchmark versions and evaluation commands**, rather than from inconsistencies in the reported results themselves. Specifically, the discrepancies can be attributed to the following three factors:

(1) HG008 benchmark preprocessing for paired BND representation.

As noted in our first revision response, earlier versions of Truvari do not fully support the comparison of paired BND representations (A→B and B→A). To address this limitation, we applied a preprocessing step similar to that used in the Severus (PMID: 40185952) to filter and harmonize paired BND entries in both the query VCFs and benchmark sets before evaluation

We note that, although the reviewer attempted to reproduce this preprocessing using the script we previously provided, earlier versions of the script did not fully expose all preprocessing steps in a standardized, function-level manner, which may have led to minor discrepancies in the resulting benchmark files. To eliminate any remaining ambiguity, we now recommend either (i) directly using the fully preprocessed HG008 benchmark files provided in the updated zenodo archive (https://zenodo.org/records/18456502), or (ii) regenerating the benchmark using the updated and standardized version of *truvari_preprocess.py* (the processing logic not changed) included in the scripts/ directory of the repository (e.g., *python truvari_preprocess.py <input_vcf> <output_vcf> hg008*).

(2) Use of the updated COLO829 benchmark.

For COLO829, our evaluation was performed using an updated somatic SV benchmark refined with the CHM13-T2T reference genome (PMID: 40097200; specified in the Data Availability section of the revised manuscript), rather than the earlier COLO829 truth set referenced by the reviewer. This updated benchmark largely preserves the core somatic SV landscape of the original COLO829 set, with refinements that reduce the impact of likely false positives and unstable, replicate-specific

events. In addition, the updated COLO829 benchmark represents BNDs as single A→B entries rather than paired records, making additional BND preprocessing unnecessary.

(3) Updated Truvari parameters not reflected in the original supplementary material.

Finally, in response to reviewer recommendations from the first revision, we used Truvari with the additional parameters *--dup-to-ins* and *--typeignore*. We acknowledge that the Truvari command reported in the supplementary materials of the revised manuscript was not updated accordingly, which may further hinder exact reproduction.

To address these issues, we have updated both the Zenodo repository (https://zenodo.org/records/18456502) and the supplementary materials. Using the provided benchmark versions of our Zenodo archive, and updated Truvari commands, all benchmarking results reported in the revised manuscript can now be reproduced exactly (**see Table R1-6**).

We sincerely apologize for the confusion and thank the reviewer for highlighting this issue. We believe that these clarifications and updates substantially improve the transparency and reproducibility of our evaluation. Detailed, point-by-point responses to the reviewer's specific comments are provided below.

Table R1. Comparison on HG008 dataset using Truvari

Truvari	truvari bench -b "$VCF" --pick multi --sizemin 50 --sizefilt 50 --sizemax -1 --passonly -p 0.00 --dup-to-ins --typeignore -o "truvari_filt/${SAMPLE}" -c "${SAMPLE}" –includebed GRCh38_HG008-T-V0.3_somatic-stvar.draftbenchmark.bed								
	Tools	TP	FP	FN	Precision	Recall	F1	TP+FP	TP+FN
	manta	89	386	27	18.74%	76.72%	30.12%	475	116
	minisv	81	7	35	92.39%	69.83%	79.54%	88	116
	nanomonsv	75	23	41	77.00%	64.66%	70.29%	98	116
	severus	88	46	28	65.93%	75.86%	70.55%	134	116
	sniffles	91	238	35	25.39%	69.83%	37.24%	319	116
	svarp	43	235	73	17.54%	37.07%	23.82%	278	116
	svpg	99	15	17	86.84%	85.34%	**86.09%**	114	116

Table R2. Comparison on HG008 dataset using script *eval_somatic.py*

eval_somatic.py	python ../eval_somatic.py $VCF *.filter.vcf $BED >eval_somatic_filt.tsv								
	Tools	TP	FP	FN	Precision	Recall	F1	TP+FP	TP+FN
	manta	81	367	31	18.08%	72.32%	28.93%	448	112
	minisv	85	15	27	85.00%	75.89%	80.19%	100	112
	nanomonsv	61	25	51	70.93%	54.46%	61.62%	86	112
	severus	86	35	26	71.07%	76.79%	73.82%	121	112
	sniffles	86	240	26	26.38%	76.79%	39.27%	326	112
	svarp	42	235	70	0.1516	0.3750	21.59%	278	112
	svpg	99	13	13	88.39%	88.39%	**88.39%**	112	112

Table R3. Comparison on HG008 dataset using minda

minda	/minda/minda.py truthset --base $VCF --vcfs *.filter.vcf.gz --out_dir minda_multimatch --multimatch –bed $BED								
	Tools	TP	FP	FN	Precision	Recall	F1	TP+FP	TP+FN
	manta	101	15	399	20.20%	87.07%	32.79%	500	118
	minisv	97	23	7	93.27%	80.83%	86.61%	104	118
	nanomonsv	83	36	53	61.03%	69.75%	65.10%	136	118
	severus	113	4	25	81.88%	96.58%	88.63%	138	118
	sniffles	83	33	216	27.76%	71.55%	40.00%	299	118
	svarp	49	76	269	15.41%	39.20%	22.12%	318	118
	svpg	103	14	11	90.35%	88.03%	**89.18%**	114	118

Table R4. Comparison on COLO829 dataset using Truvari

Truvari	truvari bench -b "$VCF" --pick multi --sizemin 50 --sizefilt 50 --sizemax -1 --passonly -p 0.00 --dup-to-ins --typeignore -o "truvari_filt/${SAMPLE}" -c "${SAMPLE}" –includedebed GRCh38_HG008-T-V0.3_somatic-stvar.draftbenchmark.bed								
	Tools	TP	FP	FN	Precision	Recall	F1	TP+FP	TP+FN
	manta	37	1443	13	2.50%	74.00%	4.84%	1480	50
	minisv	40	31	10	56.94%	80.00%	66.53%	71	50
	nanomonsv	32	26	18	55.17%	64.00%	59.26%	58	50
	severus	41	47	9	47.19%	82.00%	59.91%	88	50
	sniffles	33	302	17	10.12%	66.00%	17.55%	335	50
	svarp	16	209	34	7.52%	32.00%	12.18%	225	50
	svpg	42	21	8	66.67%	84.00%	**74.34%**	63	50

Table R5. Comparison on COLO829 dataset using script *eval_somatic.py*

eval_somatic.py	python ../eval_somatic.py $VCF *.filter.vcf $BED >eval_somatic_filt.tsv								
	Tools	TP	FP	FN	Precision	Recall	F1	TP+FP	TP+FN
	manta	32	1401	22	2.23%	59.26%	4.30%	1723	54
	minisv	41	30	13	57.75%	75.93%	65.60%	71	54
	nanomonsv	29	21	25	58.00%	53.70%	55.77%	50	54
	severus	40	38	14	51.28%	74.07%	60.61%	78	54
	sniffles	36	289	18	11.08%	66.67%	19.00%	325	54
	svarp	18	207	36	0.0800	0.3333	12.90%	225	54
	svpg	45	17	9	72.58%	83.33%	**77.59%**	62	54

Table R6. Comparison on COLO829 dataset using minda

minda	/minda/minda.py truthset --base $VCF --vcfs *.filter.vcf.gz --out_dir minda_multimatch --multimatch –bed $BED								
	Tools	TP	FP	FN	Precision	Recall	F1	TP+FP	TP+FN
	manta	35	15	1492	2.29%	70.00%	4.44%	1527	50
	minisv	41	10	44	48.24%	80.39%	60.29%	85	50
	nanomonsv	33	17	32	50.77%	66.00%	57.39%	65	50

	severus	45	6	47	48.91%	88.24%	62.94%	92	50
	sniffles	34	17	472	6.72%	66.67%	12.21%	506	50
	svarp	17	34	430	3.80%	33.33%	6.83%	447	50
	svpg	43	7	29	59.72%	86.00%	**70.49%**	72	50

Specific points that need to be addressed for these benchmarks:

1. The authors should make sure their statistics are accurate and provide transparent instructions how to reproduce the reported statistics.

Response:

We thank the reviewer for carefully attempting to reproduce our benchmarking results and for highlighting this important issue. We agree that ensuring accuracy and full reproducibility of the reported statistics is essential. As mentioned above, the discrepancies arise from differences in the benchmark set versions and evaluation procedures used during reproduction, rather than from inconsistencies in the underlying SV call sets.

To resolve these issues, we have now:

1. Updated the Zenodo repository (https://zenodo.org/records/18456502) to include the exact original benchmark VCFs and high-confidence-BEDs used, the original tool outputs, and all preprocessed benchmark files.
2. Updated the supplementary materials to list the exact Truvari commands and parameters used for somatic benchmark.

Using the provided preprocessed benchmark sets and call VCFs, together with the updated evaluation commands, all statistics reported in Supplementary Tables 9 and 10 can now be reproduced exactly. We have verified this internally and appreciate the reviewer's feedback, which helped improve the clarity and reproducibility of our evaluation.

2. It is currently unclear which evaluation results are reported in the supplementary tables-those produced by truvari or by eval_somatic.py. Moreover, eval_somatic.py is not described or referenced in the manuscript. It is currently unclear which evaluation results are reported in the supplementary tables-those produced by truvari or by eval_somatic.py. Moreover, eval_somatic.py is not described or referenced in the manuscript.

Response:

We thank the reviewer for pointing out this lack of clarity. **Supplementary Table 9** reports the benchmarking results generated using **Truvari and minda**, while **Supplementary Table 10** reports the results produced by the script of '**eval_somatic.py**'. We have now explicitly clarified this distinction in the main text and revised the manuscript to clearly describe the role of eval_somatic.py

and how its results differ from the Truvari-based evaluation. The detailed modifications are as follows:

"Pangenome-based somatic SV calling performance assessment" of Results section: We further evaluated all methods using minda (an independent somatic SV assessment tool developed for Severus), as well as our custom evaluation script specifically designed for SV-type–based assessment of cancer-associated SVs. Fig. 4b shows that SVPG exhibited highly consistent performance across these distinct evaluation tools. In addition, SVPG maintains strong and well-balanced performance across multiple SV types using evaluation scrip (Fig. 4e and Supplementary Table 10).

"Somatic SV detection benchmarking" of Methods section: Overall performance assessment employed Truvari and minda (commit 182fbcf), while SV-type–based evaluation was performed using a custom evaluation script. The script categorizes variants according to the SVTYPE field in the VCF and currently evaluates three primary SV classes: INDELs (with duplications mapped to insertions), inversions, and translocations. Matching criteria and thresholds are defined to be consistent with those used by Truvari. Supplementary Table 10 shows the custom script show high concordance with Truvari-based evaluations. Before assessment, VCFs with double-entry BNDs were preprocessed to convert them to single representation.

3. In the original VCFs, BNDs are represented as paired records in both A B and B A orientations. It seems that neither truvari nor eval_somatic.py can process such duplicate entries. The authors thus generated .filter.vcf files to remove these duplicates; however, the choice of which orientation (A->B or B->A) to retain appears to be arbitrary, leading to false mismatches during evaluation. The authors should address this, or alternatively can use Minda which automatically supports both single- or double-BND representations.

Response:

We thank the reviewer for raising this important point regarding paired BND representations and their handling during evaluation. For all SV callsets, BND preprocessing was performed per VCF using a unified, deterministic strategy, rather than an arbitrary choice of breakpoint orientation. This strategy is consistent with the preprocessing logic adopted in the Severus benchmarking framework.

All input VCFs are required to be properly sorted by genomic coordinates. Under this assumption, the overall principle of the preprocessing is to retain a single canonical representation per BND event, preferentially keeping the orientation in which the upstream chromosome appears first. The preprocessing script (truvari_preprocess.py) applies one of the following rules depending on how paired BNDs are encoded:

1. **VCFs with an explicit MATEID field in the INFO column** (e.g. the HG008 benchmark

and SVPG callsets):

In these files, the ordering of BND record IDs is consistent with the ordering of breakpoint chromosomes. For each paired BND entry, only the record with the **lexicographically smaller ID** (VCF column 3) is retained. For example, given a paired representation SV_1 → SV_10 (MATEID) and SV_10 → SV_1 (MATEID), the script retains SV_1 → SV_10 and removes the reciprocal entry. This rule is deterministic, reproducible, and independent of strand orientation.

2. **VCFs without MATEID, where paired relationships are encoded directly in the ID field** (e.g. Severus, Manta, miniSV, nanomonsv):
   In these cases, the record corresponding to the downstream chromosome is explicitly marked by a tool-specific suffix in the ID field. The preprocessing script removes duplicate entries based on these well-defined patterns (e.g. Severus paired IDs ending with _2, Manta paired IDs ending with :1). This approach follows the same logic as the BND preprocessing scripts distributed with Severus and ensures consistent canonicalization across tools.

3. **VCFs without explicit or implicit pairing annotations** (e.g. SVIM-ASM callset derived from SVarp-svtig):
   When no pairing information is available, the script retains the BND whose first breakpoint chromosome is lexicographically smaller. For example, given chr1 → chr5 and chr5 → chr1, only chr1 → chr5 is retained. This rule is deterministic given sorted input and avoids arbitrary selection.

For tools such as **Sniffles** and for the **updated COLO829 benchmark**, no preprocessing was required, as these datasets already represent BNDs as single A→B entries by default.

Finally, following the reviewer's suggestion, we additionally evaluated the benchmarks using **Minda (**commit 182fbcf), which natively supports both single- and double-BND representations. The corresponding results (Table R3 and 6) have now been added to the main text (Fig.4b and Supplementary Table 9) and are consistent with the conclusions reported using Truvari and eval_somatic.py. The detailed modifications are as follows:

"Pangenome-based somatic SV calling performance assessment" of Results section: We further evaluated all methods using minda (an independent somatic SV assessment tool developed for Severus), as well as our custom evaluation script specifically designed for SV-type–based assessment of cancer-associated SVs. Fig. 4b shows that SVPG exhibited highly consistent performance across these distinct evaluation tools. In addition, SVPG maintains strong and well-balanced performance across multiple SV types using evaluation scrip (Fig. 4e and Supplementary Table 10).

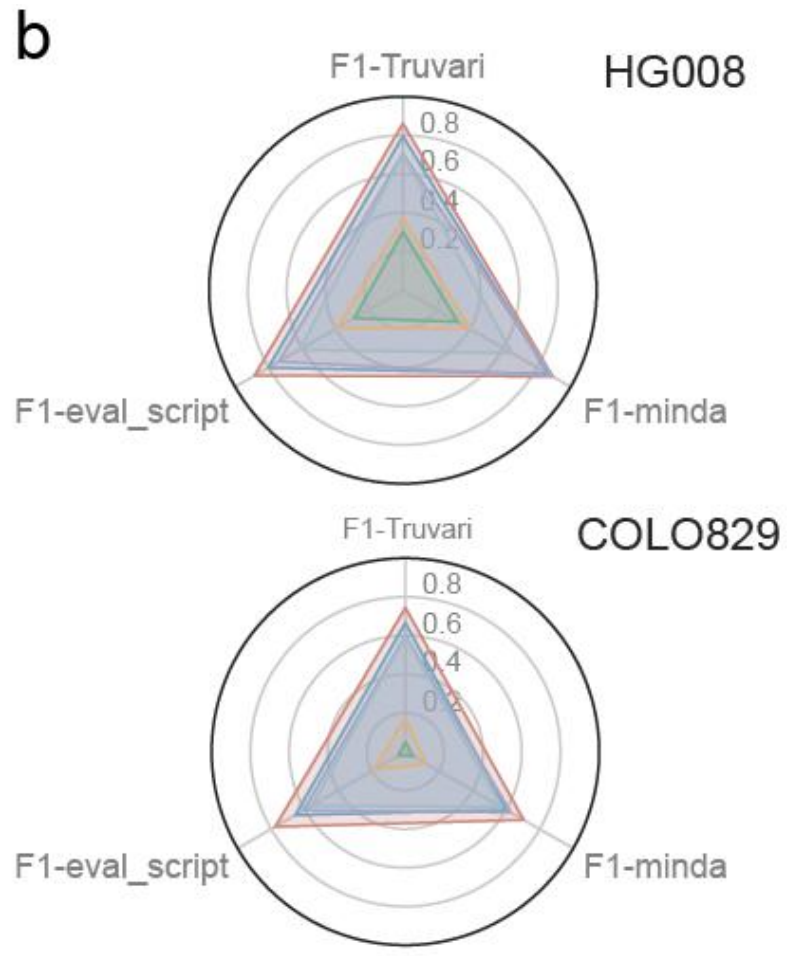

Fig. 4b *F1 scores of somatic SV calling on the HG008 and COLO829 assessed using Truvari, minda, and custom evaluation scripts.*

4. Comparison of the number of structural variants in the input VCFs with those reported in the final results table reveals discrepancies. Specifically, 118 SVs were expected in the HG008 truth (within BED intervals), yet only 112 calls were evaluated (TP + FN column); similarly for COLO829 the number decreased from 63 (truth vcf) to 54 (TP + FN). These differences indicate that some calls were excluded from the analysis. The criteria for excluding these SVs are not described.

Response:

We carefully examined the original truth VCFs and the VCFs after preprocessing and evaluation, and confirmed that the observed discrepancies in the number of evaluated SVs arise from two well-defined and previously insufficiently documented causes, rather than from arbitrary exclusion.

(1) Complex SVs represented as BNDs in the HG008 benchmark

Our custom evaluation script categorizes variants based on SVTYPE field of VCF and currently evaluates three primary classes—INDELs (duplications mapped to INS), inversions, and translocations. In the HG008 benchmark, however, a small number of SVs are reported exclusively as BND records that represent *complex events* (e.g. ultra-large tandem duplications, ultra-large deletions, or chromoplexy), spanning very large genomic distances and lacking well-defined SVLEN annotations.

During evaluation, these complex SVs were filtered out because (i) they cannot be reliably mapped to a single canonical indel category, and (ii) the absence of SVLEN prevents length-based normalization and comparison. Upon inspection, we confirmed that exactly five such complex SVs account for the discrepancy, and are listed below for full transparency:

*chr17 32305445 SV_130 A [chr17:32673231[AA . PASS SVTYPE=BND;EVENTTYPE=DUP:TANDEM;MATEID=SV_200;EVENT=cluster_12;SUBCLONAL=n;COMPLEX=y GT:PSL:PSO:CN 0|1:.,chr17*32305445*1:.,1:.*
*chr19 44719441 SV_147 T]chr19:44916907]T . PASS SVTYPE=BND;EVENTTYPE=DUP;MATEID=SV_181;EVENT=cluster_6;CIPOS=-1,1;CIEND=-1,1;HOMLEN=2;HOMSEQ=AT;SUBCLONAL=n;COMPLEX=y GT:PSL:PSO:CN 0|1|1:.,chr19*38022545*1,chr19*38022545*1:.,3,5:.*
*chr19 39706584 SV_145 C CT[chr19:50554251[. PASS SVTYPE=BND;EVENTTYPE=DEL;MATEID=SV_184;EVENT=cluster_6;SUBCLONAL=n;COMPLEX=y GT:PSL:PSO:CN 0|0|0|1:.,.,.,chr19*38022545*1:.,.,.,8:.*
*chr19 40470024 SV_146 G G[chr19:44908989[. PASS SVTYPE=BND;EVENTTYPE=DEL;MATEID=SV_180;EVENT=cluster_6;CIPOS=0,2;CIEND=0,2;HOMLEN=2;HOMSEQ=GG;SUBCLONAL=n;COMPLEX=y GT:PSL:PSO:CN 0|1|1:.,chr19*38022545*1,chr19*38022545*1:.,2,6:.*
*chr3 139998694 SV_21 C [chr3:193903984[C . PASS SVTYPE=BND;EVENTTYPE=CHROMOPLEXY;MATEID=SV_174;EVENT=cluster_3;SUBCLONAL=n;COMPLEX=y GT:PSL:PSO:CN 1|0:chr13*114353244*1,.:3,.:.*

In addition, our script removed SVs that are less than 50bp. As a result, the number of HG008 benchmark SVs changed as follows: **BND-filtered & BED: 118 → SVLEN < 50 filtered: 117 → complex BND SVs filtered: 112.**

To directly address the reviewer's concern, we revised the evaluation script to explicitly include these five complex SVs as a separate category in the assessment. As shown in **Table R7**, the total number of evaluated HG008 benchmark variants (TP + FN) now equals **117**, **matching the number of benchmark SVs passing size filtering.** Importantly, the overall performance trends remain unchanged: SVPG continues to achieve the highest F1 score, demonstrating that the original conclusions are robust to this correction.

Table R7. Comparison on HG008 dataset using script *eval_somatic_update.py*

	python ../eval_somatic_update.py $VCF *.filter.vcf $BED >eval_somatic_filt.tsv								
	Tools	TP	FP	FN	Precision	Recall	F1	TP+FP	TP+FN
eval_somatic_update.py	manta	82	413	35	16.57%	70.09%	26.80%	448	117
	minisv	85	15	32	85.00%	72.65%	78.34%	100	117
	nanomonsv	63	40	54	61.17%	53.85%	57.27%	86	117
	severus	89	49	28	64.49%	76.07%	69.80%	121	117
	sniffles	86	240	31	26.38%	73.50%	38.83%	326	117
	svarp	42	235	75	15.16%	35.90%	21.32%	278	117
	svpg	99	13	18	88.39%	84.62%	**86.46%**	112	117

(2) Size filtering in the original COLO829 benchmark

For COLO829, the reduction from 63 benchmark SVs to 54 evaluated SVs is explained by the presence of **nine SVs with SVLEN < 50 bp** in the original truth set. These small events were excluded by the size filter applied in the evaluation script, which was designed to focus on canonical SVs.

Notably, this issue does not arise when using the updated COLO829 benchmark refined with the CHM13-T2T reference genome, as this updated benchmark already removes SVs shorter than 50 bp. Accordingly, all benchmark SVs in the updated COLO829 set are consistently evaluated, and no discrepancy between truth set size and TP + FN counts is observed.

In summary, the discrepancies in SV counts are fully explained by (i) the initial exclusion of 5 complex BND SVs in HG008, and (ii) the presence of short (<50 bp) variants in the original COLO829 benchmark. We have updated the evaluation scripts and supplementary tables to clearly describe these criteria and ensure that all reported statistics are accurate, transparent, and reproducible.

5. Additionally, the --sizemin 50 and --sizefilt 50 parameters in the truvari version 5.3 do not appear to function as expected, leading to an increased false-negative rate.

Response:

We sincerely thank the reviewer for this valuable and constructive suggestion. We carefully investigated the behavior of the *--sizemin 50* and *--sizefilt 50* parameters in Truvari (v5.3), which are intended to filter out SVs smaller than 50 bp during benchmarking (see https://github.com/ACEnglish/truvari/wiki/bench#size-filtering).

Upon detailed inspection, we found that these parameters behave as expected in our evaluations. Specifically, in the original HG008 filtered.vcf (restricted to BED intervals), there is exactly one SV with *SVLEN = -47*, shown below:

chr9 73758583 SV_77 AGAAAAAAAAAAGAGTTAAAGATGTTTTATGTTTTTTTTTTTTTTGCAT TAAGTGGTCAGATGAAGGCAGCACTATTGGTTCATTTATGGGCTAT . PASS SVTYPE=DEL;EVENTTYPE=DEL;SVLEN=-47;END=73758653;SUBCLONAL=n;COMPLEX=n GT:PSL:PSO:CN 0/1:.:.:.

After manually examining all Truvari output files (including TP, FN, and FP VCFs), we confirmed that this variant does not appear in any of the evaluation outputs. Consistently, the Truvari's log reports *{'__filtered': 1}*, indicating that this variant was filtered as intended.

To further validate this behavior independently of Truvari's internal filtering, we manually applied size filtering using bcftools:

bcftools view -i 'abs(SVLEN)>=50 || SVTYPE="BND"' \ GRCh38_HG008-T-V0.3_somatic-stvar_PASS.draftbenchmark.filtered.vcf -Oz -o filtered50.vcf.gz

We then reran the benchmarking using *filtered50.vcf.gz* as the benchmark set and obtained results that were identical to those produced by Truvari with *--sizemin 50 and --sizefilt 50*.

Together, these checks indicate that the size-filtering parameters in Truvari v5.3 function as expected in our evaluation and do not introduce an increased false-negative rate in this context.

6. The description of the methodology (versions, command line parameters, inputs etc) should be synchronized across all manuscript materials, as currently the reviewer's response document, main paper text, supplementary information and supplementary script archive are not always consistent, for example in describing truvari parameters and inputs.

Response:

We thank the reviewer for highlighting this issue. We have thoroughly cross-checked all manuscript materials and synchronized the methodological descriptions across the main text, supplementary information, reviewer response document, and the supplementary script archive. In particular, all Truvari versions, command-line parameters, and input specifications are now fully consistent. These revisions ensure a clear, unambiguous, and reproducible description of the evaluation workflow.

7. The authors need to provide truthset vcf files that were used to generate the final statistics, as well as unmodified outputs of all tools before preprocessing.

Response:

We sincerely thank the reviewer for this valuable and constructive suggestion. To address this point, we have now made all truth-set VCF files used to generate the final statistics publicly available via the updated Zenodo repository (https://zenodo.org/records/18456502). In addition, we have uploaded the complete, unmodified output VCFs produced by all evaluated SV callers prior to any preprocessing steps.

This update ensures full transparency of the evaluation pipeline and allows reviewers and readers to independently verify the impact of preprocessing and reproduce the final benchmarking statistics exactly.

Reviewer #2 (Remarks to the Author):

The authors have done a lot of additional work in this revision which is a notable improvement over the initial version. I still have a few minor comments:

1) The old SV v0.6 benchmark contains quite a few wrong SVs. The new Q100 is of higher quality overall. Instead of using tier 2 in v0.6 (line 133), the authors may subtract v0.6 Tier 1 regions from Q100 confident regions and use the remainder as harder regions.

Response:

We sincerely thank the reviewer for this valuable and constructive suggestion. Following the reviewer's recommendation, we constructed a more challenging evaluation set by subtracting the SV v0.6 Tier 1 regions from the Q100 confident regions. Specifically, we used *bedtools subtract* command to exclude genomic intervals overlapping v0.6 Tier 1 from the Q100 benchmark, and used the remaining regions as a set of harder evaluation regions. Using this revised benchmark, we re-evaluated all SV callers across both sequencing platforms. The results show that SVPG consistently achieves the highest average F1 score on both HiFi and ONT data (Figure R1, Supplementary Table 2). Compared to the original v0.6 benchmark, the Q100-based evaluation exhibits a stronger dependence on sequencing platform, with HiFi consistently outperforming ONT across methods.

We note that the observed discrepancies between evaluations using v0.6 and Q100 benchmarks are partly attributable to differences in how ground truth SVs are constructed and how difficult regions are defined during benchmarking. Importantly, **SVPG's relative performance remains robust across benchmarks with differing definitions of confident and difficult regions**.

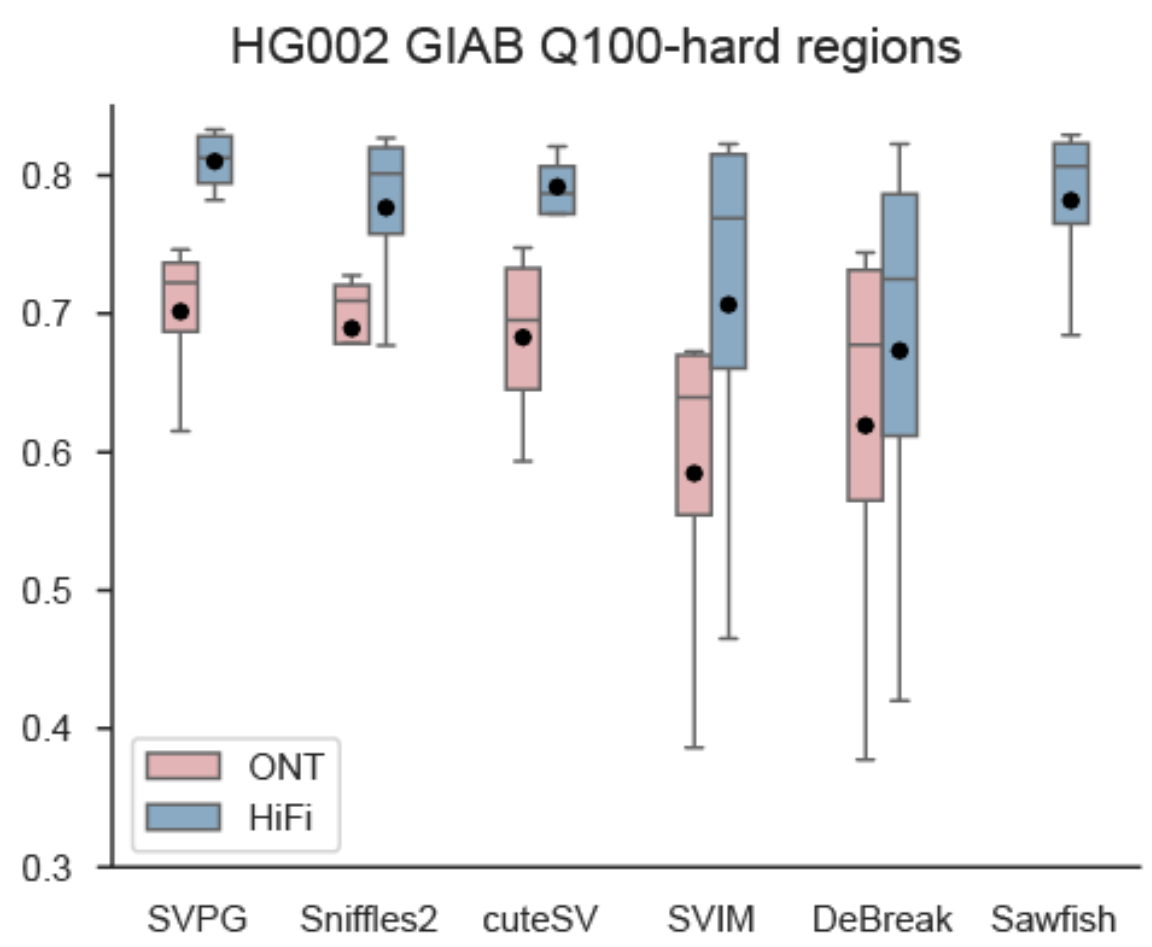

Fig. R1 Comparison of SV calling F1 score at multiple coverages levels (5-50×) across Q100-hard genomic regions on HG002.

The detailed modifications are as follows:

"SVPG enables high performance SV calling on the GIAB ground truth set" of Results section: In addition, we applied all callsets to the Q100-defined hard regions to assess the consistency of SVPG's performance (see Methods for details on region construction). Under this evaluation, SVPG outperformed all other approaches, achieving average F1 scores of 70.2% and 81.0% on ONT and HiFi data, respectively (Supplementary Table 2). Overall, SVPG consistently maintained stable, high-precision performance under various testing conditions, facilitating the reliable discovery of SVs in complex regions.

"Benchmarking SV callers on GIAB ground truths and complex regions" of Methods section: Q100-hard regions were constructed by subtracting the SV v0.6 Tier 1 regions from the Q100 confident regions using the "bedtools subtract" command. The remaining genomic intervals, which are not covered by v0.6 Tier 1 but still within the Q100 confident regions, were designated as harder evaluation regions.

2"Cancer-specific somatic SV" is a confusing terminology: by definition, somatic events are cancer specific (i.e. they are not in the sample's germline); then "non-cancer-specific somatic SV" becomes more confusing. I would remove the terminologies and say (e.g. line 306): "they are likely somatic SVs overlapping with common germline SVs".

Response:

We thank the reviewer for pointing out this important terminology issue. We agree that the phrase "cancer-specific somatic SV" is potentially confusing, as somatic variants are, by definition, cancer-specific, and that the corresponding term "non–cancer-specific somatic SV" is ambiguous.

Following the reviewer's suggestion, we have removed the term "cancer-specific somatic SV" throughout the manuscript. We now use either "somatic SV" or "cancer-specific SV" where appropriate. In addition, we describe "non-cancer-specific somatic SV" more precisely as somatic SVs overlapping with common germline SVs.

"Pangenome-based somatic SV calling performance assessment" of Results section: Furthermore, a closer inspection of HG008 dataset the five INDELs missed by SVPG revealed that four were already represented in the pangenome graph, suggesting that they are likely somatic SVs overlapping with common germline SVs (Figs. 4d show one case; see Supplementary Note 2 for all). Overall, we attribute the improvement and low false-positive rate results to SVPG effectively leverages pangenome characteristics to enable precise inference cancer-specific SV.

3) Relatedly, I found the response to Reviewer 1's comment 1 is weak. The authors take the paragraph starting at line 310 as an advantage of SVPG but I see this the major limitation. Some tumor types are associated with frequent STR/VNTR expansions. These real somatic events often overlap with common germline SVs and would be missed by SVPG. It is okay to have weakness,

but I hope the authors can rewrite this paragraph and properly discuss the effect of somatic SVs overlapping with common germline SVs.

Response:

We thank the reviewer for this insightful comment and fully agree that the behavior described in the paragraph starting at line 310 represents a limitation rather than an advantage of SVPG.

SVPG is designed to prioritize somatic SVs that introduce novel structural paths relative to common population variation represented in the pangenome. As a result, somatic repeat expansions or contractions occurring in STR/VNTR-rich regions—where common germline SVs are prevalent—may overlap existing pangenome structures and therefore be missed by SVPG. This limitation is particularly relevant for tumor types characterized by frequent repeat-length alterations.

Following the reviewer's suggestion, we have rewritten the paragraph starting at line 310 to explicitly describe this behavior as a limitation and to discuss its potential impact on somatic SV detection. We also clarify that this challenge is not unique to SVPG, but reflects a broader difficulty in distinguishing true somatic events from overlapping germline SV in highly polymorphic regions using current evaluation frameworks.

"Pangenome-based somatic SV calling performance assessment" of Results section: We additionally analyzed an earlier yet widely-studied HCC1395 breast cancer cell line[39] , and its matched normal HCC1395BL. This benchmark was established through the biological validation of a curated subset of SVs derived from consensus germline callsets. Because the dataset lacks rigorously defined high-confidence genomic regions necessary for an accurate assessment of false positives, we prioritized recall as the primary metric for our performance evaluation. As shown Fig. 4e, SVPG achieved the highest sensitivity, identifying 155 out of 179 validated SVs (Supplementary Tables 10), followed closely by the pangenome-based method miniSV. We further performed the same evaluation on the consensus benchmark set comprising 1,268 SVs identified by at least two germline SV callers. We found that 951 SVs were not reported by SVPG. Notably, the majority of these SVs were also missed by nanomonsv (949) and Severus (899), indicating that these events may be broadly challenging for current somatic SV callers. Among the 951 SVs not detected by SVPG, cross-validation using bedtools intersect showed that 829 overlap with node regions in the GRCh38-90c.r518-based pangenome reference, indicating that these hard-to-call somatic SVs occur in regions enriched for common germline SV (Supplementary Table 11). In contrast, only 25 of the 285 SVs detected by SVPG overlap with pangenome nodes, which is significantly lower than the overlap observed in the unrecalled group (Fisher's exact test, $p < 0.001$). These regions expose a fundamental limitation of current somatic SV evaluation frameworks, where independent somatic events may overlap common germline variation in highly polymorphic regions. This ambiguity affects

SVPG as well as other state-of-the-art callers, underscoring the need for more refined benchmarking strategies that explicitly account for overlapping germline and somatic variation.

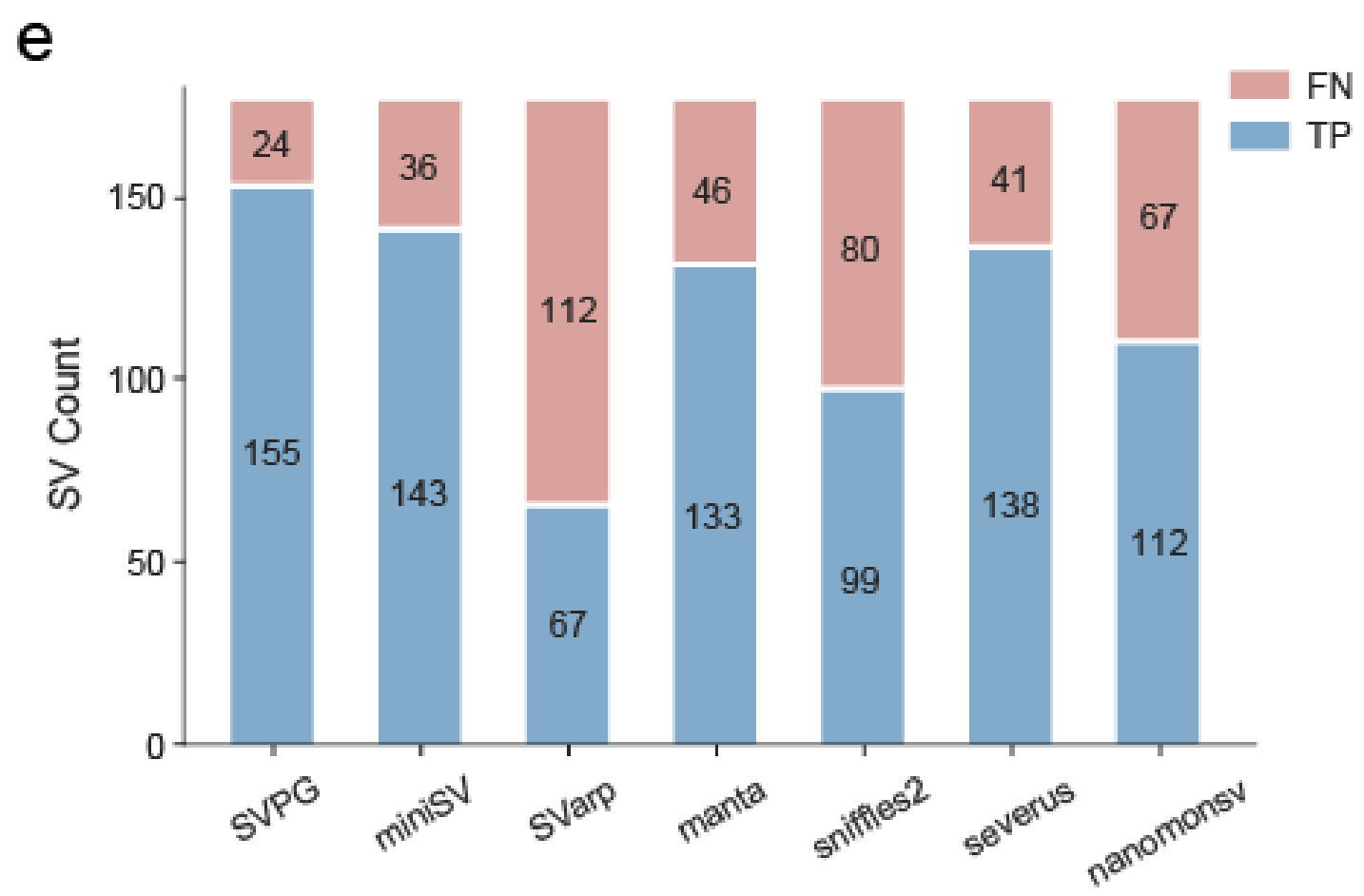

Fig.4e True positive (TP) and false positive (FP) counts of somatic SV calling on the HCC1395 biologically validated benchmark.
